# Unlocking photochemical tunability in functionalised bridged-isoindigo molecular motors

**DOI:** 10.1039/d5sc08776g

**Published:** 2026-01-19

**Authors:** Carlijn L. F. van Beek, Ainoa Guinart, Yusuf Qutbuddin, Ben L. Feringa

**Affiliations:** a Stratingh Institute for Chemistry, University of Groningen Groningen 9747AG The Netherlands b.l.feringa@rug.nl; b Cellular and Molecular Biophysics, Max Planck Institute of Biochemistry 82152 Martinsried Germany

## Abstract

Artificial molecular machines enable precise control over motion on the molecular scale. Dual-rotor molecular motors offer unique opportunities for the development of responsive functional systems and molecular machines, yet remain considerably underexplored compared to single-rotor motors. Here, we report six new light-driven bridged-isoindigo-based dual motors, developed through strategic rotor substitution, to investigate the tunability of their rotational behaviour. While thermal processes were largely unaffected by rotor substitution, the photochemical properties were significantly influenced. All functionalised motors retained visible-light addressability, with substitution enabling additional modulation of their absorption wavelengths. Rotor functionalisation also impacted the photostationary state composition and the photochemical accessibility of specific intermediates. Notably, we made the unique observation of a photochemical generated double metastable state in light-driven molecular motors, highlighting the potential for advanced control over dual motor function. The synthetic versatility of the bridged-isoindigo scaffold was further demonstrated by the successful post-functionalisation and membrane incorporation of a representative motor, underscoring its promise for future applications in adaptive molecular systems.

## Abstract 2

Rotor-functionalised bridged-isoindigo motors exhibit tuneable absorption and photochemical behaviour without altering thermal kinetics. Incorporation into lipid bilayers enables light-driven membrane perturbation.

## Graphical abstract

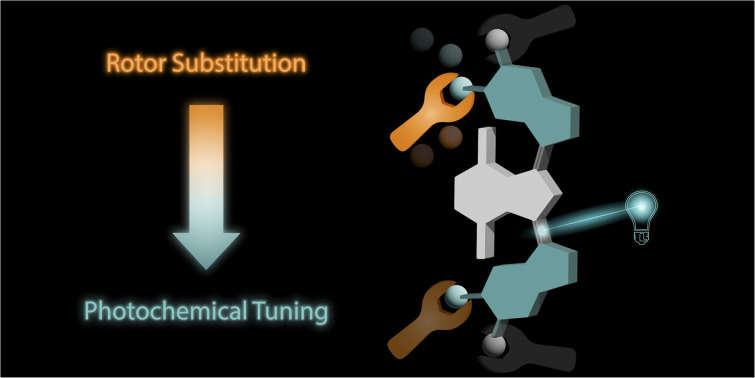

## Introduction

The field of artificial molecular machines aims to emulate the efficiency, precision, and complexity of biological molecular machinery present in nature.^1–5^ A wide array of synthetic machine analogues have been developed that are capable of performing mechanical tasks at the nanoscale,^6–10^ powered by external energy inputs such as redox processes,^11–13^ chemical fuels,^14–17^ or light.^18–20^ These molecular machines have found applications across diverse fields, including biomedical systems,^21,22^ responsive smart materials,^23–26^ and catalysis.^27^ Light-driven molecular motors have garnered particular attention due to their ability to harness light energy and convert it into continuous directional rotation with high temporal and spatial control.^28^ Since the development of the first light-driven unidirectional rotary motor by our group,^29^ extensive progress has been made in tuning the properties of overcrowded alkene-based systems, including their absorption wavelengths, quantum yields, and rotational speeds.^30–40^ Moreover, the emergence of alternative scaffolds, such as hemothioindigo-based^41–43^ and imine-based^44,45^ motors introduced by the groups of Dube and Lehn, respectively, has expanded the repertoire of light-driven molecular motors. This progress has established an understanding of how molecular structure governs motor function, paving the way for innovative designs that extend beyond classical concepts.

**Fig. 1:**
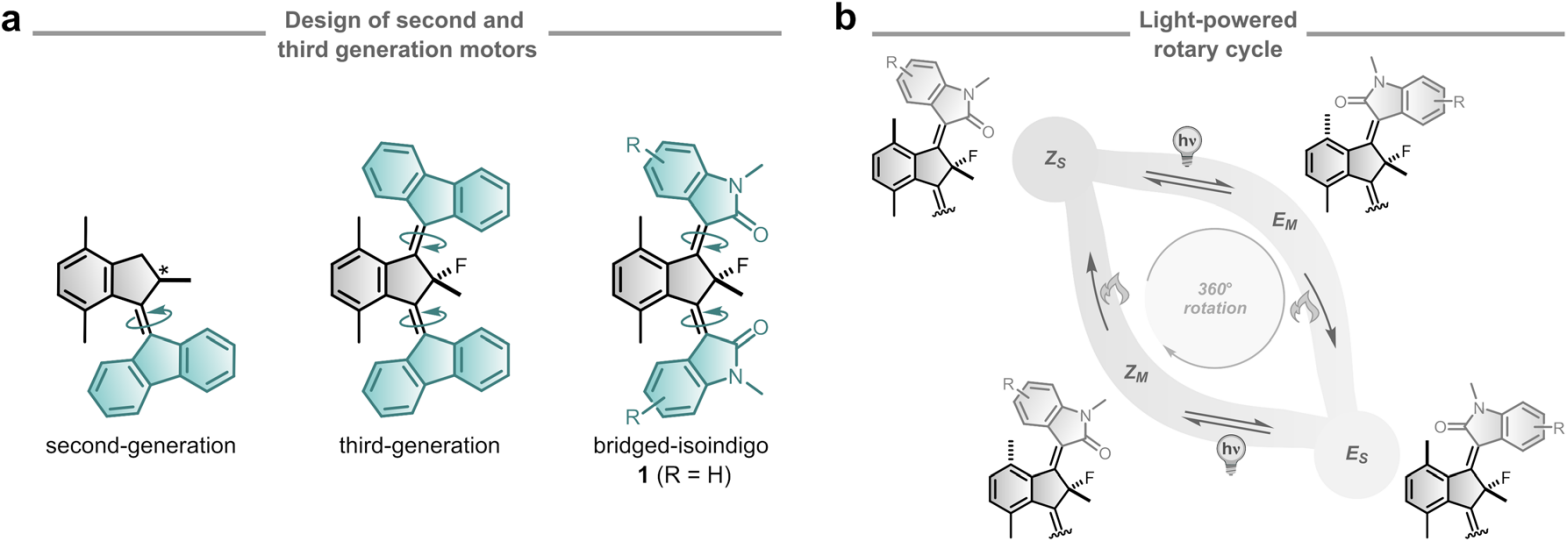
Design and typical rotational behaviour of overcrowded alkene-based molecular motors. (a) Achiral third-generation motors were realised from merging two second-generation motors and subsequently developed into bridged-isoindigo motors. (b) Typical four-step light-driven unidirectional rotation cycle of overcrowded alkene-based molecular motors comprised of two photochemical *E*/*Z* (PEZ) isomerisations and each followed by a thermal helix inversion (THI) process.

Overcrowded alkene-based molecular motors rely on chirality to enforce unidirectional rotation.^29,46^ A unique subclass is represented by third-generation molecular motors, which no longer possess an asymmetric centre – an essential feature in first- and second-generation designs.^29,30^ Instead, these motors consist of *meso* structures containing only a pseudo-asymmetric centre located at the bridgehead position of the core (Fig. 1a). Third-generation motors can be regarded as a fusion of the two enantiomers of a second-generation motor into a single, achiral architecture.

Previous studies on third-generation molecular motors focused on fluorene-based rotors established the principle of a pseudo-asymmetric centre to drive unidirectional rotation and modulation of this motion through structural modifications of the core motif.^47–49^ Although the entire system is symmetric, the opposite helicities around the central C=C bonds (axles of rotation) proved to be sufficient to induce the unidirectional rotation of the rotors.^47^ Each rotor follows the characteristic four-step rotation cycle comprising of alternating photochemical *E*/*Z* (PEZ) isomerisation and thermal helix inversion (THI) steps (Fig. 1b). Notably, both rotors exhibit coordinated motion, rotating in the same direction relative to the aromatic core – analogous to two wheels on an axle, with one rotating clockwise and the other counterclockwise. The substituents at the indane bridgehead position were shown to control the degree of unidirectionality, while alterations to the aromatic core allowed tuning of the rotational speed by adjusting the steric demand in the fjord region.^48^ Additionally, distal substituents were introduced at the aromatic core to enhance solubility and enable post-functionalisation.^49^ Compared to earlier single-rotor designs, third-generation motors combine dual, coordinated rotors with enhanced mechanical output and visible-light activation. While third-generation molecular motors present highly interesting properties, for example, for cargo transport or locomotion,^49,50^ their cumbersome synthesis and limited solubility have hampered further investigations and applications.

**Fig. 2:**
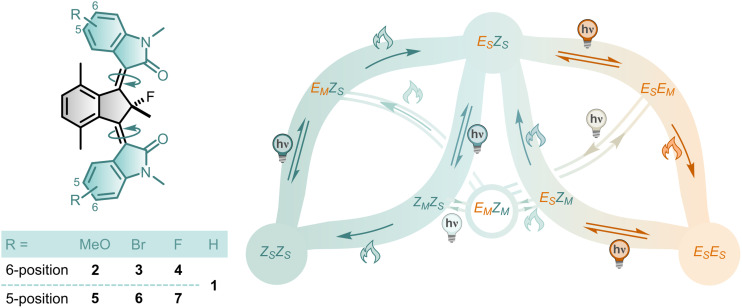
Functionalised bridged-isoindigo motors and their rotation mechanism. The studied functionalised bridged-isoindigo molecular motors 2–7 and the corresponding unidirectional rotational mechanism. The rotational cycle connects stable, single metastable, and double metastable isomers *via* repetitive photochemical *E*/*Z* (PEZ) and thermal helix inversion (THI) steps. During PEZ isomerisation, one of the alkenes undergoes an *E*/*Z* isomerisation, accompanied by interconversion between stable and metastable geometries of the attached rotor. THI corresponds to the thermal relaxation of a rotor from a metastable to a stable configuration. The two four-step leafs illustrate a complete 360° unidirectional rotation of one of the rotors, while off-leaf pathways show the connectivity of the double metastable state (*E*_M_*Z*_M_) to all single metastable states, enabled by additional PEZ isomerisation and THI steps. For the structures of the intermediates see Fig. S23.

More recently, we revised the third-generation motor design by using oxindole-based instead of fluorene-based rotors.^51^ The resulting bridged-isoindigo scaffold offers many advantages over traditional third-generation molecular motors, including a more easily accessible synthesis, greatly improved solubility in organic solvents, and additional functionalisation handles. For future applications, the ability to tune motor properties (such as absorption profile, quantum yield, and rotational frequency) to suit specific functional requirements is essential, for instance in light-driven cargo transport, responsive materials, or biological systems. One strategy involves substitution of the motor, which not only allows fine-tuning of the motor's behaviour but also introduces additional sites amenable to further modification. In this work, we explored the use of functionalised rotors in a bridged-isoindigo motor motif to assess their impact on the motor's photochemical and thermal properties (Fig. 2). Structural modifications focused on introducing substituents in conjugation with either the central C=C bonds (6-position) or the oxindolic amides (5-position).

The unidirectional rotation mechanism of unfunctionalised motor 1 was previously elucidated in detail, enabled by the unique combination of desymmetrised oxindole rotors and a fluorine spectroscopic handle at the pseudo-asymmetric centre within the bridged-isoindigo motif.^51^ This design allowed for precise monitoring of the individual isomerisation steps and revealed unexpected additional mechanistic complexity in the form of coupled rotor motion. The same rotational mechanism applies to the new functionalised motors 2–7 (Fig. 2). For bridged-isoindigo motors, four different stable isomers exist: *Z*_S_*Z*_S_, *E*_S_*Z*_S_, *Z*_S_*E*_S_, and *E*_S_*E*_S_, denoting the *E*/*Z* configuration of each alkene and the corresponding stable (S) or metastable (M) rotor geometries. When both rotors are functionalised identically, *E*_S_*Z*_S_ and *Z*_S_*E*_S_ form an enantiomeric pair, resulting in two mirror-image rotational cycles that converge at the *meso* isomers *Z*_S_*Z*_S_ and *E*_S_*E*_S_. For clarity, a simplified mechanism featuring a single rotational cycle *via E*_S_*Z*_S_ is used throughout this work. Each four-step leaf (Fig. 2) represents a full 360° unidirectional rotation of one of the rotors *via* repetitive PEZ isomerisation and THI steps. Coupled motion between the rotors (CO-flip) provides access to the double metastable state isomer *E*_M_*Z*_M_, which interconnects to all single metastable state through additional PEZ isomerisation and THI steps.^51^

## Results and discussion

### Synthesis

**Scheme 1:**
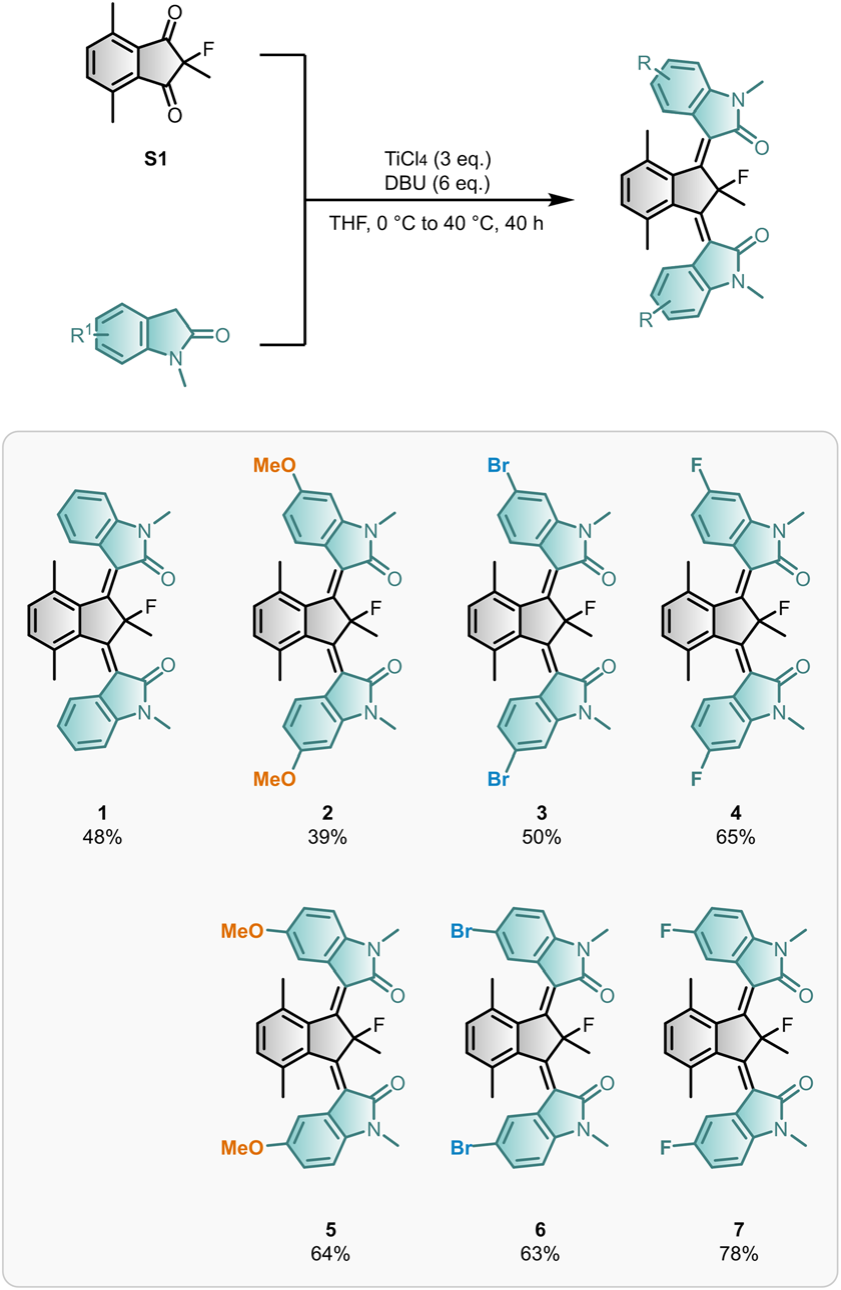
| Synthesis of bridged-isoindigo motors. The synthesis of functionalised motors 1–7 (obtained as mixtures of *Z*_S_*Z*_S_, *E*_S_*Z*_S_, and *E*_S_*E*_S_ isomers; only *Z*_S_*Z*_S_ isomers are depicted) *via* the optimised double Knoevenagel reaction.

We build on our reported synthesis of motor 1 which used a TiCl_4_-mediated one-pot double Knoevenagel condensation of diketone S1 and *N*-methyl oxindole.^51^ Since mono-rotor motors and β-hydroxyamide intermediates were present in the reaction mixture after heating overnight, the influence of additional base and longer reaction times was investigated. To our delight, doubling the equivalents of base (DBU) and extending the reaction time improved the isolated yield of dual motor 1 from 30% to 48% (Scheme 1). Motors 2–7 were prepared *via* the optimised double Knoevenagel procedure in good yields (up to 78%), which is comparable to the best yields reported for installing a single rotor.^52^ Motors 1–7 were obtained as mixtures of their stable isomers (*Z*_S_*Z*_S_, *E*_S_*Z*_S_, and *E*_S_*E*_S_), which could be separated by flash column chromatography. An exception occurred for (*Z*_S_*Z*_S_)-6 and (*Z*_S_*Z*_S_)-7, for which only enriched samples, rather than pure isomers, were isolated.

Functionalised rotors are well tolerated in the synthesis of bridged-isoindigo motors, though their compatibility depends on the specific substitution pattern. Notably, motor 2 suffered from considerable defluorination of the final motor under the reaction conditions, reflected in the isolated yield of defluorinated motor 2DeF (18%) (Section S2). F-substituted rotors displayed the highest yields and a lower degree of defluorination at the quaternary position. Defluorination is hypothesised to occur *via* Lewis acid-mediated fluoride ion abstraction of the central core. Likely, electron-rich substituents in conjugation with the central C=C bonds, such as the 6-methoxy substituents present in motor 2, can provide additional stabilisation for the associated delocalised carbocation motor intermediate, favouring the undesirable defluorination pathway.

### Photochemical and thermal behaviour

**Fig. 3:**
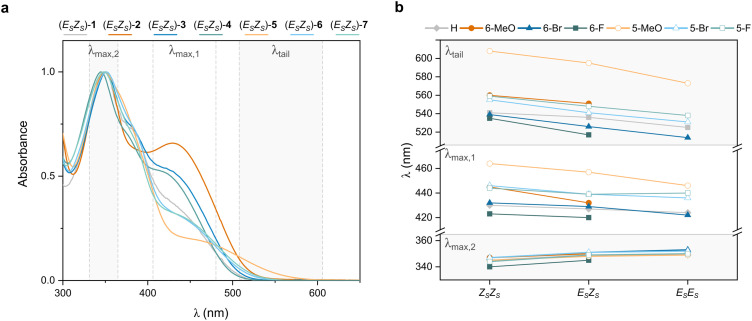
UV-Vis absorption data of motors 1–7. (a) UV-Vis absorption spectra of (*E*_S_*Z*_S_)-1–7 in CH_2_Cl_2_ at rt. The spectra are normalised for the second absorption band. (b) Key parameters in UV-Vis absorption spectra of the isomers of motors 1–7. The visible light and UV absorption bands are defined by *λ*_max,1_ and *λ*_max,2,_ respectively, and *λ*_tail_ is the highest wavelength with a non-zero absorption.

The photochemical behaviour of motors 2–7 was first studied by ultraviolet-visible (UV-Vis) absorption spectroscopy. All motors show two main absorption bands, one in the visible light region (*λ*_1_ = 420–465 nm) and one in the ultraviolet (UV) region (*λ*_2_ = ∼350 nm) (Fig. 3a), in accordance with our previous study.^51^ Since the two absorption bands overlap, the highest wavelength with a non-zero absorption (*λ*_tail_) is also reported to aid evaluation of the changes in the visible light absorption band. The absorption spectra of *Z*_S_*Z*_S_ isomers are red-shifted relative to those of the corresponding *E*_S_*Z*_S_ isomers, with an even greater bathochromic shift compared to the *E*_S_*E*_S_ isomers (Fig. 3b, *λ*_max,1_ and *λ*_tail_). The UV absorption band *λ*_max,2_ follows the opposite trend and is slightly blue-shifted from *E*_S_*E*_S_ to *E*_S_*Z*_S_ to *Z*_S_*Z*_S_.

Functionalisation of the rotors can be used to achieve large bathochromic shifts of the maxima of the first absorption band (Δ*λ*_max,1_ up to 41 nm and Δ*λ*_tail_ up to 78 nm). Methoxy substitution on the rotors induces a bathochromic shift, although the extent of the shift depends on the position of substituent. Substitution at the 6-position of the rotor leads to a pronounced effect on the first absorption band (*λ*_1_), while minimally affecting the second absorption band (*λ*_max,2_ is within 6 nm for the *E*_S_*Z*_S_ isomers of all motors, Fig. 3b). The contribution of the first absorption band is increased for motors 2–4 compared to motor 1 (Fig. 3a). For 5-subsititution, being a position not directly conjugated to the central C=C bonds, the effects are highly dependent on the nature of the substituent. The UV-Vis absorption spectra of motors 6–7 are comparable to motor 1, showing only minor red shifts. Motor 5 behaves differently from all the other motors, exhibiting a markedly red-shifted and broadened absorption band in the visible region.

Irradiation of the stable isomers of each motor with 455 nm light at room temperature was monitored by UV-Vis absorption spectroscopy (Section S3). Metastable states of motors 1–7 cannot be observed at room temperature, as expected based on the low thermal barriers for THI steps in bridged-isoindigo motors.^51^ The spectral changes observed upon irradiation reflect the interconversion between stable states, progressing until the photostationary state (PSS) is established. The resulting PSS is independent of initial stable isomer (*Z*_S_*Z*_S_, *E*_S_*Z*_S_, or *E*_S_*E*_S_) used (Fig. S6–S11).

**Fig. 4:**
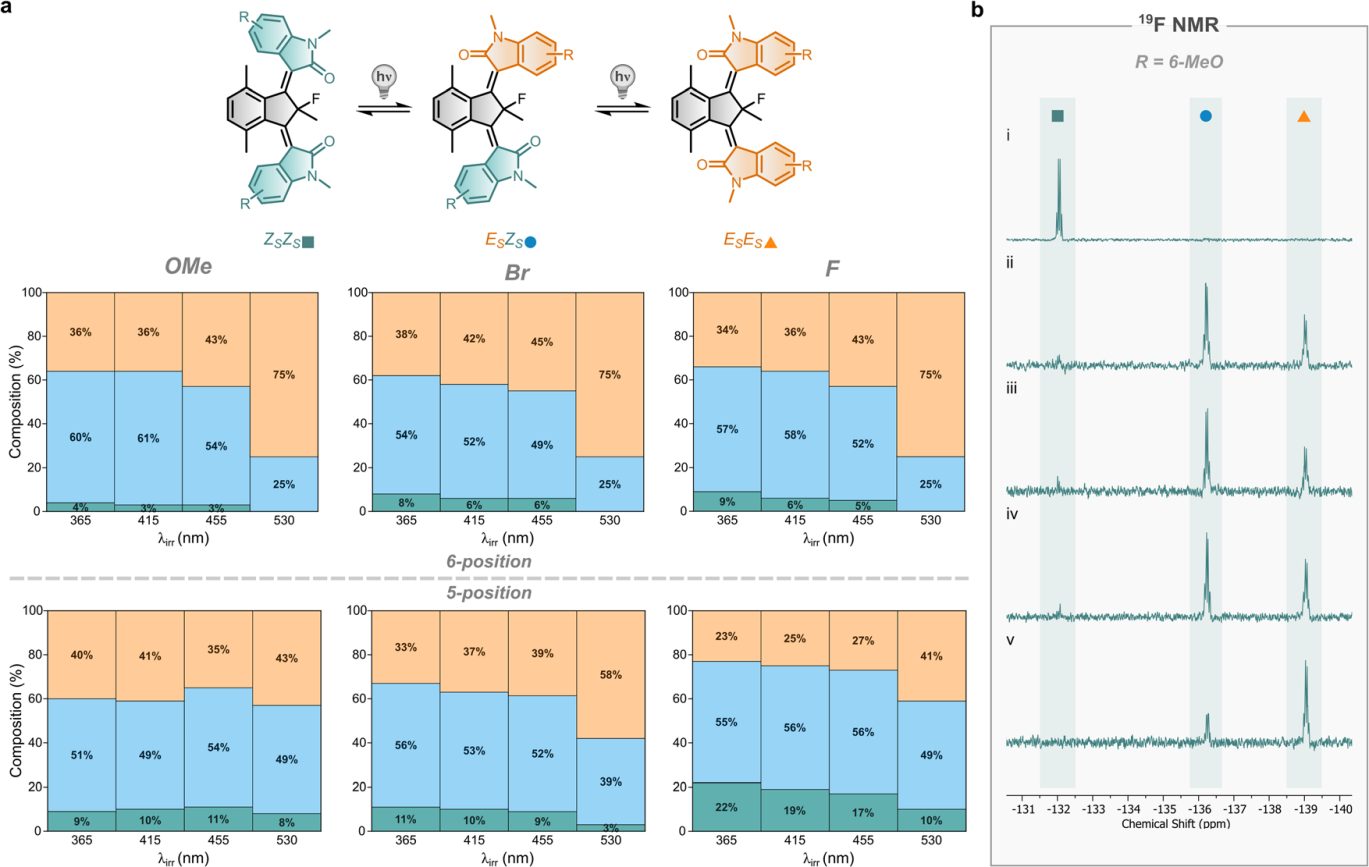
PSS ratios for motors 1–7. (a) PSSs of motors 1–7 at room temperature for four different irradiation wavelengths (365, 415, 455 and 530 nm). Under these conditions, the PSS mixture contains only stable state isomers (*Z*_S_*Z*_S_, *E*_S_*Z*_S_, and *E*_S_*E*_S_; indicated by green, blue and orange areas, respectively) as determined by NMR spectroscopy. (b) Representative ^19^F NMR spectra of 6-MeO substituted motor 2: prior to irradiation (i) and PSSs reached after *ex situ* irradiation with 365 nm (ii), 415 nm (iii), 455 nm (iv), or 530 nm (v).

*Ex situ* irradiation experiments were performed to quantify the PSS ratios at various irradiation wavelengths (*λ*_irr_) using nuclear magnetic resonance (NMR) spectroscopy (Fig. 4a). The distinct signals of the core fluorine atom of the stable states (*Z*_S_*Z*_S_, *E*_S_*Z*_S_, and *E*_S_*E*_S_) are key to their differentiation by ^19^F NMR spectroscopy (Fig. 4b). In motors 4 and 7, the presence of F-substituted rotors gives rise to additional signals that further support the isomer assignment. The determined PSS ratios consist solely of stable state isomers under the experimental conditions (25 °C) and are identical regardless of the starting isomer (Fig. S16–S21), consistent with the UV-Vis studies.

5-Bromo functionalised motor 6 exhibits PSS ratios very similar to those of unfunctionalised motor 1 across all tested irradiation wavelengths. The ratios for the other functionalised motors vary, but the same wavelength-dependent trends are observed (Fig. 4a). Lowering *λ*_irr_ slightly increases the population of the *Z*_S_*Z*_S_ isomer, while increasing *λ*_irr_ favours the *E*_S_*E*_S_ isomer, with the largest increase observed when switching from 455 nm to 530 nm. 5-Methoxy functionalised motor 5 does not follow this trend and continues to display unique behaviour. Although modulation of the PSS of motor 5 is still possible by selecting different irradiation wavelengths, the extent of modulation is minimal compared to the other motors.

Most motors reached the PSS within minutes of irradiation, whereas 5-methoxy functionalised motor 5 required prolonged irradiation (12 h) to achieve the PSS, as observed in both NMR and UV-Vis experiments (Section S3 and Fig. S14). The low photochemical efficiency of motor 5 likely stems from a low quantum yield, similar to that observed in 2,7-dimethoxy-fluorene rotors,^34,53^ which also exhibit low isomerisation quantum yields and share a similar substitution pattern to the 5-MeO group in motor 5. For applications requiring functionalisation at the 5-position, it is therefore recommended to avoid 5-methoxy functionalised motor 5 and related 5-alkoxy substitutions. Instead, 5-bromo substituted motor 6 could be a more suitable alternative, offering the potential for further modification *via* direct coupling of the desired linker. Although the linker identity will likely affect the motor's photochemical behaviour, this substitution strategy is anticipated to offer more favourable properties than 5-alkoxy functionalisation.

**Fig. 5:**
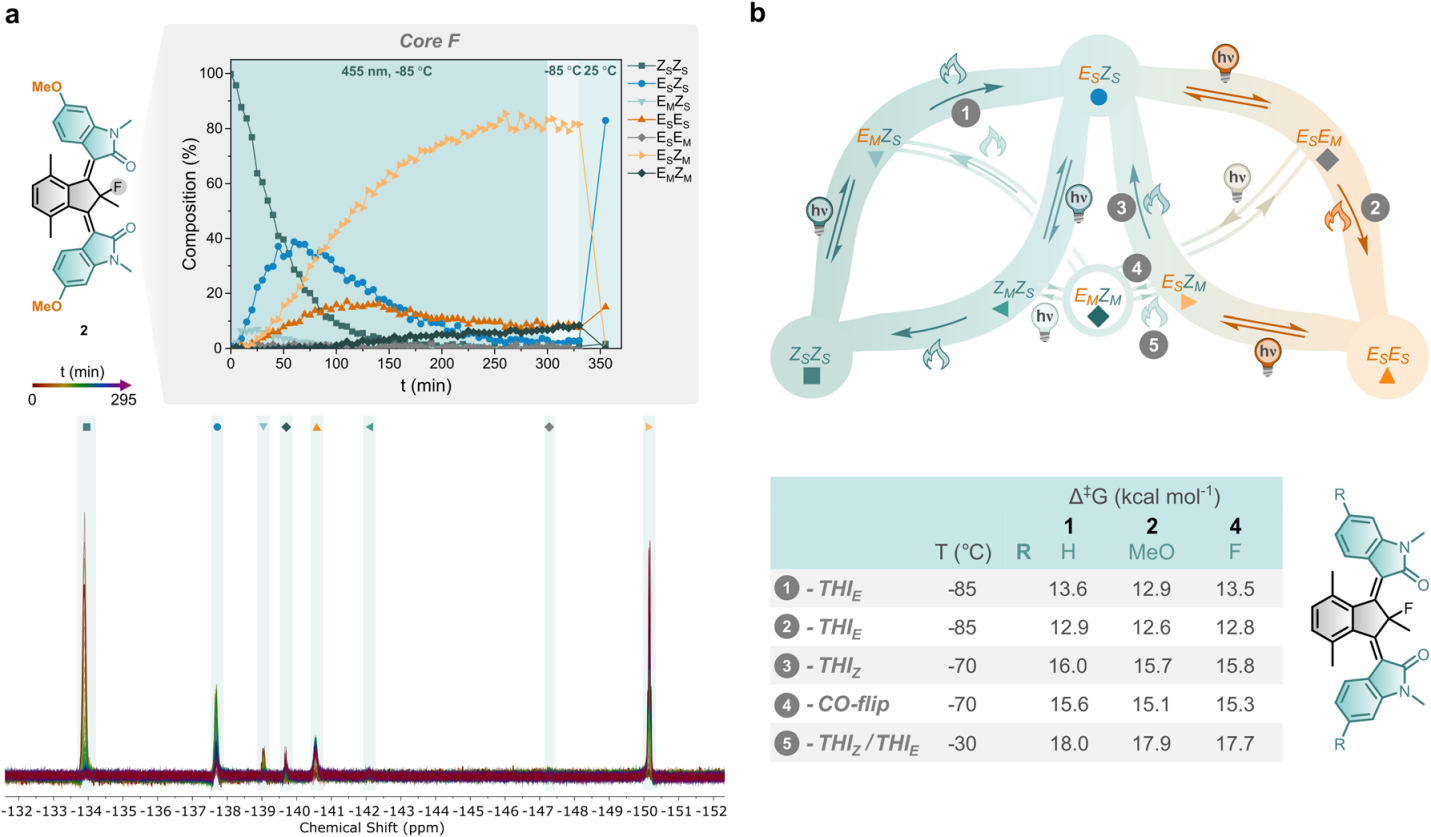
Photochemical isomerisation and thermal relaxation of substituted bridged-isoindigo motors. (a) Compositional changes of a sample of (*Z*_S_*Z*_S_)-2 in CD_2_Cl_2_ irradiated to PSS with 455 nm at −85 °C, subsequently kept at −85 °C for 35 min, and warmed up to room temperature (top). The corresponding superimposed ^19^F spectra (470 MHz) of (*Z*_S_*Z*_S_)-2 upon irradiation at −85 °C with isomer assignment (bottom). (b) Overview of the experimentally determined activation barriers of the thermal processes (steps 1–5) of motors 2 and 4, and compared to those of reported motor 1. For the structures of the involved intermediates see Fig. S23.

To investigate the individual steps in the rotation mechanism, motors 2 and 4 were studied using low-temperature *in situ* NMR irradiation experiments. Irradiation to PSS at −85 °C followed by step-wise thermal relaxations of motors 2 and 4 revealed the involvement of the anticipated eight intermediates (Section S4). The observed compositional changes over time were consistent with a sequence-specific mechanism, supporting the retention of unidirectional rotation. Three new observations emerged from the kinetic data. Motor 4 exhibited behaviour similar to that previously reported for motor 1,^51^ but with detectable formation of the *Z*_M_*Z*_S_ isomer (Fig. S28 and S29). In motor 1, a strong photochemical bias of the *E*_S_*Z*_S_ isomer toward formation of *E*_S_*E*_M_ exists, and the *Z*_M_*Z*_S_ isomer could only be populated indirectly, *via* the partially relaxed *E*_M_*Z*_M_ state.^51^ While this bias – favouring photochemical activation of its *Z* rotor over *E* rotor – remains present in motor 4, it is slightly reduced, thereby allowing direct population of *Z*_M_*Z*_S_ from *E*_S_*Z*_S_. For motor 2, the formation of *Z*_M_*Z*_S_ was also minimal; however, unexpectedly, substantial formation of the double metastable *E*_M_*Z*_M_ intermediate occurred (Fig. 5a, with its key signal observed at −139.7 ppm). As thermal pathways to the *E*_M_*Z*_M_ isomer are inaccessible under the experimental conditions (−85 °C), its formation must occur *via* a photochemical process, from either *Z*_S_*Z*_M_ or *E*_M_*E*_S_. While the formation of *E*_M_*Z*_M_*via* thermal pathways is established, this represents the first example of photochemical generation of a motor's double metastable state isomer. Upon closer inspection of the kinetic data of motor 4, *E*_M_*Z*_M_ was also formed, albeit at levels near the detection limit of NMR spectroscopy (Fig. S28 and S29). The two other conceivable double metastable-state isomers (*Z*_M_*Z*_M_ and *E*_M_*E*_M_) were not detected in the current study, in line with our previous findings.^51^ Lastly, all tested motors showed the *E*_M_*Z*_S_ isomer as the major component in the achieved PSS composition at −85 °C, but the PSS ratios depended on the substitution of the motor. Introducing 6-fluorine substituents shifted the PSS more toward the *E*_M_*Z*_S_ isomer under these conditions (PSS : *E*_M_*Z*_S_ : *E*_S_*E*_S_ : *E*_S_*Z*_S_ = 74 : 20 : 6 for 4 and 65 : 35 : 0 for 1). The presence of 6-methoxy substitution in motor 2 further enriched the population of the *E*_M_*Z*_S_ isomer and introduced the *E*_M_*Z*_M_ isomer into the PSS (*E*_M_*Z*_S_ : *E*_S_*E*_S_ : *E*_S_*Z*_S_ : *E*_M_*Z*_M_ = 82 : 8 : 2 : 8 for 2). These findings indicate that the photochemical processes are highly dependent on both the *E*/*Z* configurations of the alkenes and the nature of the rotor's substituent.

Since substitution of bridged-isoindigo motors affected their photochemical behaviour, we proceeded to investigate whether it also influenced the thermal processes. The activation barriers, extracted from the low-temperature *in situ* irradiation experiments (Table S1), are summarised in Fig. 5b. The activation barriers for the THI processes are very comparable for motors 2 and 4. THI_*E*_ processes, steps 1 and 2, proceed rapidly even at low temperature due to low activation barriers (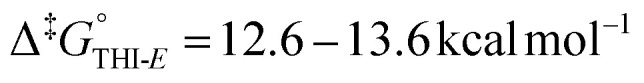, corresponding to half-lives *t*_1/2_ of several minutes at −85 °C). At −70 °C, partial relaxation *via* coupled rotor motion (step 4, CO-flip is a formal THI_*E*_) is slightly favoured over direct full relaxation *via* THI from *E*_M_*Z*_S_ to *E*_S_*Z*_S_ (step 3). Full relaxation of *E*_M_*Z*_M_ to *E*_S_*Z*_S_ (step 5) at −30 °C is associated with a considerably higher activation barrier (Δ^‡^*G*° ∼18.0 kcal mol^−1^). This relaxation process occurs without the re-population *E*_M_*Z*_S_, preventing elucidation of the origin of its increased barrier of activation compared to steps 3 and 4 – an observation also previously reported for motor 1. The observed thermal behaviour and activation barriers associated with the various relaxation processes of motors 2 and 4 are consistent with those reported for motor 1. Substitution thus exerts little to no influence on the THI activation barriers, presumably due to the outward-facing orientation of tested substituents, which leads to comparable steric congestion in the fjord region and, consequently, similar activation barriers. These findings are consistent with prior reports on substitution effects in related single-rotor oxindole-based motors.^54,55^

### Application of a functionalised bridged-isoindigo motor in lipid membranes

**Fig. 6:**
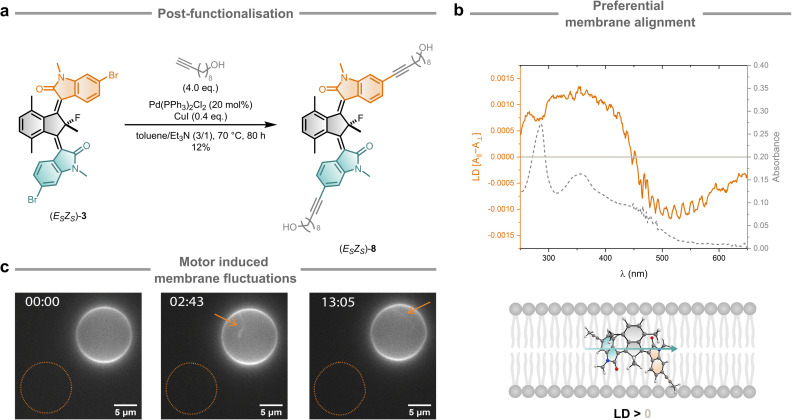
Bridged-isoindigo motor embedded in lipid membranes. (a) Synthesis of membrane motor 8*via* post-functionalisation of bromo-functionalised motor 3 through Sonogashira coupling. (b) UV/Vis absorption (grey) and linear dichroism spectra of motor (*E*_S_*Z*_S_)-8 in a lipid environment (10 mol%, SUVs 100 nm *∅*, MilliQ, 5 °C (UV-Vis) and 20 °C (LD)). (c) Representative time-lapse images of motorised POPC GUVs inducing lipid membrane area expansion under 405 nm light exposure (exposure time in minutes). Arrows indicate tubulations and the equatorial plane trace (dotted orange line) shows membrane fluctuations.

To illustrate potential applications, motor 8 was designed for incorporation into the membrane of synthetic unilamellar vesicles (Fig. 6). For these studies, 1-palmitoyl-2-oleoyl-*sn*-glycero-3-phosphocholine (POPC) was used, a lipid previously employed for embedding second-generation molecular motors into reconstructed membranes.^56,57^ The *E*_S_*Z*_S_ isomer of the motor was selected, as it features linkers on opposite sides, which can facilitate successful motor incorporation and positioning in the lipid bilayer. Linkers of appropriate length, matching to POPC bilayer thickness (around 4 nm), were introduced to bromo-functionalised motor 3*via* a Sonogashira coupling (Fig. 6a). The moderate yield of the desired product 8 can be attributed, at least in part, to the small reaction scale, undesired photoisomerisation during the coupling, and incomplete conversion – mono-linked motor being the major by-product – even after extended reaction times.

Small unilamellar vesicles (SUVs) prepared with 10 mol% (*E*_S_*Z*_S_)-8 showed UV-Vis absorption bands in the 280–520 nm range, attributable to the motor unit, confirming successful motor incorporation into the SUVs (Fig. 6b, grey dotted line). Upon irradiation, the observed spectral changes of the motorised SUVs closely matched those of other functionalised bridged-isoindigo motors in organic solvents, demonstrating that motor functioning is retained in the membrane environment (Section S5). Linear dichroism (LD) and time-dependent density functional theory (TD-DFT) studies indicate that (*E*_S_*Z*_S_)-8 preferentially aligns with its central C=C bonds oriented perpendicular to the membrane normal (Fig. 6b and S45). Full incorporation of 8 into the hydrophobic section of the lipid bilayer is confirmed due to the stability of the absorbance spectra and the absence of aggregates observed under cryo-transmission electron microscopy (Fig. S46). We employed focal correlation spectroscopy (FCS) to analyse the change in membrane fluidity upon incorporation of 8. Compared with pure POPC membranes, SUVs embedding 8 showed an increase membrane fluidity by 295% (Fig. S42). This observed change in membrane fluidity is in line with other studies using second-generation molecular motors,^56^ and can likely be attributed to the introduction of defects in the hydrophobic section of the lipid packing.

Next, the impact of *in situ* irradiation of molecular motors under fluorescence microscopy using giant unilamellar vesicles (GUVs) was examined. For GUV preparation, we employed PVA-assisted swelling in a 300 mM sucrose solution, followed by 30× dilution in equimolar glucose solution (Section S5). Once diluted and stabilized in a temperature-controlled chamber, samples were irradiated with the respective laser lines in epifluorescence mode using confocal microscopy (300 mW cm^−2^). The samples were irradiated every second and lasted an average of 30 min. Molecular motors are known to form small aggregates inside artificial lipid bilayers and induce membrane transformations due to a process of area expansion.^56,57^ We confirmed a similar behaviour for 8 which initiated membrane perturbations after an average of 15 s of irradiation (Fig. 6c, S47 and Video S1). Remarkably, our results show the fastest reaction time reported for a molecular motor inducing area expansion in lipid bilayers and correlates with the fact that motors orientating perpendicular to the membrane normal make vesicles react faster (Fig. S48). Here, we are only considering the starting time of visible fluctuations and not their magnitude. Incorporating a saturation concentration of 8, increased the observed effects (Video S2). These results highlight the feasibility of integrating light-responsive molecular motors into lipid bilayers, paving the way for their use in constructing dynamic membrane systems for synthetic cells. Compared to second-generation overcrowded alkene-based motors, the bridged-isoindigo design confers several advantages for membrane integration. Its red-shifted absorption enables visible-light activation that is more biologically compatible, while the increased planarity and π-conjugation can promote the formation of larger or more ordered aggregates within the bilayer. The bridged-isoindigo motor scaffold also provides a larger number of well-defined conformational states, expanding the range of accessible geometries within these aggregates and potentially amplifying membrane area changes under illumination. In addition, its amphiphilic substituent pattern enhances membrane compatibility and orientation control. Together, these features support a tighter coupling between rotary motion and local lipid packing, highlighting the potential of bridged-isoindigo motors for sophisticated light-driven membrane modulation.

## Conclusions

In this work, the scope of bridged-isoindigo molecular motors has been expanded to include variants bearing functionalised rotors, specifically with substituents at the 5- and 6-positions. The new functionalised derivatives were readily accessed *via* an optimised Knoevenagel condensation, with visible light-driven unidirectional rotary motion fully retained. Rotor substitution enabled modulation of key motor properties such as the absorption spectrum and PSS composition, which can be further adjusted by selecting the irradiation wavelength. Notably, 5-MeO substitution induced the largest red-shift in the absorption band, albeit accompanied with a reduced photochemical efficiency. Other substitutions, however, did not compromise motor performance. Detailed investigation of 6-substituted motors (MeO and F) also revealed, for the first time, photochemical generation of a double metastable state isomer – previously only produced *via* thermally coupled rotor motion – uncovering mechanistic pathways that had remained experimentally elusive. Full kinetic analysis further showed that the thermal processes were unaffected by rotor substitution, in sharp contrast to the pronounced influence on the motor's photochemical behaviour. This aspect is particularly useful for future applications, as it enables the attachment of relevant functional groups without altering the motor's rotational frequency. Finally, the successful integration of a post-functionalised bridged-isoindigo molecular motor into lipid systems, where it preferentially aligns through intercalation with the lipid bilayer and results in light-induced membrane perturbations, highlights the versatility and application potential of this scaffold. Together, these findings provide a valuable foundation for the selection and design of light-driven dual motors tailored for specific tasks in future nanoscale systems.

## Author contributions

C. L. F. B. and A. G. designed the study. C. L. F. B. conducted the synthesis and characterisation, and performed the NMR and UV-Vis irradiation studies, data analysis, LD measurements and computational study. A. G. prepared the vesicles and carried out all measurements and analyses on molecular motors embedded in lipid systems. Y. Q. performed the diffusion coefficient experiment. B. L. F. supervised the research and acquired funding. C. L. F. B. and A. G. prepared the manuscript; and all the authors discussed the results and commented on the manuscript.

## Conflicts of interest

There are no conflicts to declare.

## Supplementary Material

SC-OLF-D5SC08776G-s001

SC-OLF-D5SC08776G-s002

SC-OLF-D5SC08776G-s003

## Data Availability

The data supporting this article have been included as part of the supplementary information (SI) and as two SI video files. Supplementary information is available. See DOI: https://doi.org/10.1039/d5sc08776g.
