# Supplementary material for: Unlocking photochemical tunability in functionalised bridged-isoindigo molecular motors
Source: Chem Sci. 2026 Jan 19;17(10):5063–71. doi: 10.1039/d5sc08776g (PMC12813872; doi:10.1039/d5sc08776g)
Supplement: SC-017-D5SC08776G-s003 [file SC-017-D5SC08776G-s003.pdf]

Supplementary Information (SI) for Chemical Science.
This journal is © The Royal Society of Chemistry 2026

aSupporting Information

Unlocking Photochemical Tunability in Functionalised Bridged-Isoindigo Molecular Motors

Carlijn L. F. van Beek[1], Ainoa Guinart[1], Yusuf Qutbuddin[2], Ben L. Feringa[1]*

[1]Stratingh Institute for Chemistry, University of Groningen; Groningen, 9747AG, the Netherlands
[2]Cellular and Molecular Biophysics, Max Planck Institute of Biochemistry, 82152 Martinsried, Germany

*Corresponding author. Email: b.l.feringa@rug.nl

Contents

1. General Information 3
2. Synthetic Procedures 4
   - Synthesis Overview 4
   - Rotor syntheses 5
   - Motor syntheses 9
   - Post-functionalisation 22
3. UV/Vis Absorption Spectra 24
   - Irradiation studies 26
4. NMR Studies and Kinetic Experiments 30
   - NMR Irradiation Experiments 30
     - Room temperature experiments 30
     - Unidirectional Rotation Mechanism 42
     - Low-temperature experiments 43
   - Kinetic analysis 53
     - Motor 2 55
     - Motor 4 58
5. Membrane-spanning Molecular Motor 62
   - Vescile preparation 62
   - Measurements on Membrane Fluidity (Diffusion coefficient) 62
     - Small Unilamellar Vesicles (SUVs): 62
     - Fluorescence Correlation Spectroscopy (FCS): 63
   - UV-Vis Absorption Studies 64
   - Linear Dichroism 65
   - Cryo-TEM 67
   - Measurements of Membrane Fluctuations 68
     - Giant Unilamellar Vesicles (GUVs): 68
     - Fluorescence Microscopy: 68
     - Spinning Disk Microscopy: 68
6. Computational Details 70
7. NMR Spectra 72
   - Rotors 72
   - Motor 2 81
   - Motor 3 93
   - Motor 4 104
   - Motor 5 110
   - Motor 6 119
   - Motor 7 128
   - Motor 8 137
8. HRMS Spectra 141
9. References 152

1. General Information

All reagents were obtained from commercial sources and used as received without further purification. Dry solvents were obtained from a MBraun solvent purification system. Progress of the reactions was determined by UPLCMS (Waters Acquity Ultra Performance LC system with Acquity UPLC BEH C18, 2.1 x 50 mm 1.7 μm particles column) and TLC: silica gel 60, Merck, 0.25 mm. The TLC plates were visualized with ultraviolet (UV) light (λ = 254 nm or 355 nm). Column chromatography was performed on a Biotage Selekt System. High Resolution Mass Spectra (HMRS) were obtained using an LTQ Orbitrap XL. NMR spectra were recorded on a Varian Mercury Plus (^{1}H: 400 MHz, ^{13}C: 100 MHz, ^{19}F: 376 MHz), an Agilent MR (^{1}H: 400 MHz, ^{19}F: 376 MHz), a Varian Innova (^{1}H: 500 MHz, ^{19}F: 470 MHz) or a Bruker Avance Neo with Cryoprobe Prodigy BBO (^{1}H: 600 MHz, ^{13}C: 150 MHz, ^{19}F: 565 MHz) instrument. Chemical shifts (δ) are in parts per million (ppm) relative to TMS. Chemical shifts are reported in δ-units (ppm) relative to the residual solvent peak of $CDCl_3$ (^{1}H NMR, δ = 7.26 ppm; ^{13}C NMR, δ = 77.16 ppm) or CD_2Cl_2 (^{1}H NMR, δ = 5.32 ppm; ^{13}C NMR, δ = 53.84 ppm). For ^{1}H-NMR spectroscopy, the splitting pattern of peaks is designated as follows: s (singlet), d (doublet), t (triplet), q (quartet), m (multiplet), dd (doublet of doublets), td (triplet of doublets), dq (quartet of doublets), and qt (quartet of triplets). Single-crystal X-ray diffraction measurements were performed on a Bruker-AXS D8 Venture diffractometer. UV/Vis absorption spectra were recorded on a Agilent Cary 8454 spectrophotometer in a 1 cm quartz cuvette. The LEDs were attached via a 1500 μm optical fiber (M93L01). Solvents used for spectroscopic studies were of spectroscopic grade (UVASOL, Merck). Dichloromethane and Et_2O were degassed with and stored under argon. Irradiation experiments were performed using Thorlabs LEDs (M365D2, M415L4, M455L4, M530L4). NMR irradiation experiments were performed at the indicated temperature with a fiber-coupled Thorlabs LED and a 1500 μm optical fiber (FT1500UMT) to guide the light into the NMR tube inside the NMR spectrometer. Samples were equilibrated in a pre-cooled Varian Innova 500 spectrometer for 30 min or until the lock signal stabilised.

Compound **S1** was synthesised according to a literature procedure.[1]

2. Synthetic Procedures

Synthesis Overview

A previously reported $TiCl_4$-mediated double Knoevenagel condensation[1] was optimised to convert core **S1** with *N*-methyl oxindole or functionalised *N*-methyl oxindoles **S2-7** into motors **1-7** (Figure S1). These motors were isolated as mixtures of the stable Z_SZ_S, E_SZ_S and/or E_SE_S isomers.

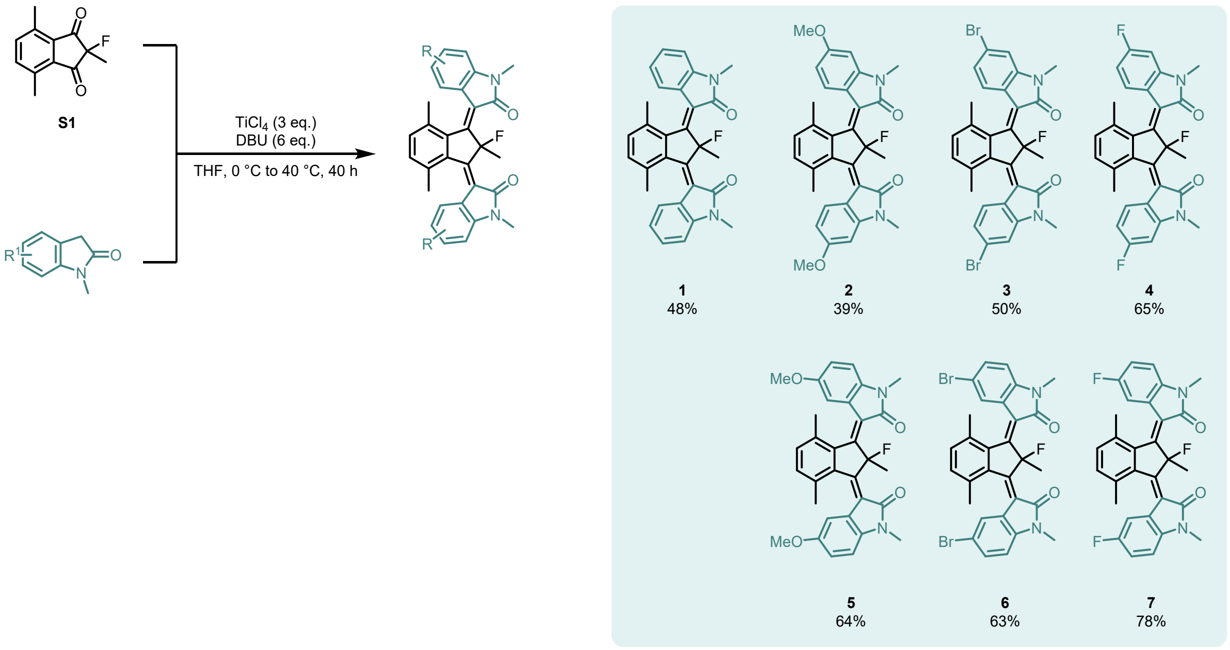

Figure S1. *Synthesis of functionalised bridged isoindigo motors* ***1-7*** *(obtained as mixtures of Z_SZ_S, E_SZ_S, and E_SE_S isomers; only Z_SZ_S isomers are depicted).*

The functionalised oxindole-based rotors **S2**-**S7** were synthesised via three different strategies (Figure S2): 1. *N*-methylation of the isatin analogue followed by a Wolff-Kishner reduction (**S2**, **S3**); 2. *N*-methylation of the corresponding 2-oxindole using Me_2SO_4 (**S3**, **S4**) or TMAF (**S7**); 3. Selective bromination of *N*-methyl oxindole with NBS (**S6**).

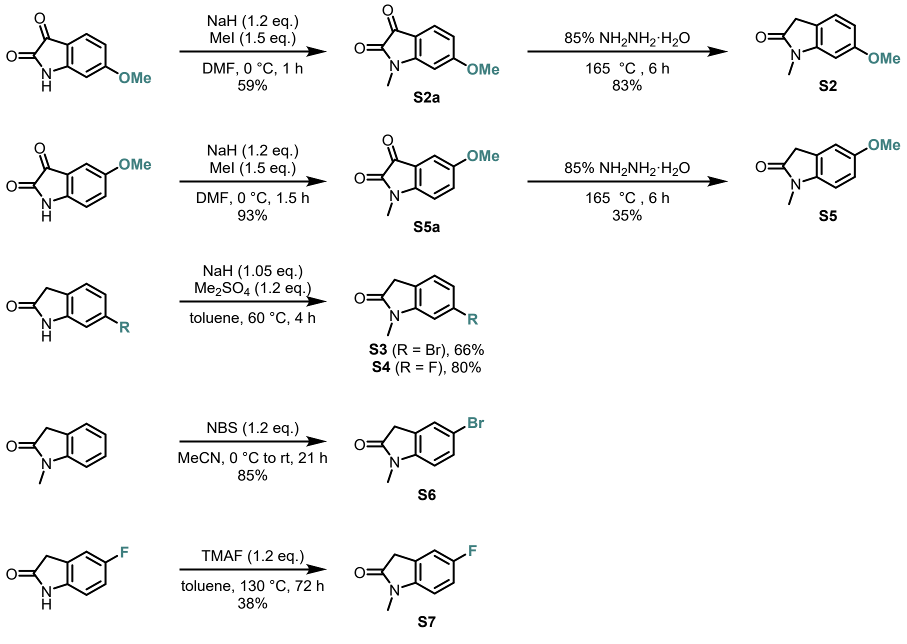

Figure S2. *Synthesis of functionalised rotors* ***S2-S7***.

Rotor syntheses

6-methoxy-1-methylindoline-2,3-dione (S2a).

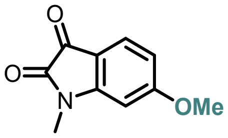

S2a

A modified literature procedure[2] was used for the synthesis of **S2a**. An oven-dried 100 mL round-bottom flask was charged with 6-methoxyisatin (2.66 g, 15.0 mmol, 1.00 eq.) and anhydrous DMF (60 mL). The resulting solution was cooled down to 0 °C and NaH (720 mg, 18.0 mmol, 1.2 eq., 60 wt% dispersion in mineral oil) was added in batches. After stirring for 5 min. at 0 °C, MeI (1.4 mL, 23 mmol, 1.5 eq.) was added. The reaction mixture was stirred for 1 h at 0 °C and then poured into ice-water and extracted with EtOAc (3 x 50 mL) and CH_2Cl_2 (3 x 70 mL). The combined organic layers were washed with brine (75 mL), dried over anhydrous Na_2SO_4, and concentrated *in vacuo*. The residue was purified by recrystallisation from EtOAc (100 mL). The product was obtained as orange crystals (1.68 g, 8.79 mmol, 59%). Spectroscopic data according to literature[3].

TLC: R_f (pentane/EtOAc 50:50) = 0.45

^{1}H NMR (600 MHz, $CDCl_3$) δ 7.58 (d, *J* = 8.4 Hz, 1H), 6.56 (dd, *J* = 8.4, 2.1 Hz, 1H), 6.37 (d, *J* = 2.1 Hz, 1H), 3.93 (s, 3H), 3.22 (s, 3H).

^{13}C NMR (151 MHz, $CDCl_3$) δ 180.9, 168.5, 159.8, 154.2, 128.1, 111.4, 108.0, 97.3, 56.3, 26.1.

6-methoxy-1-methylindoline-2,3-dione (S5a).[2]

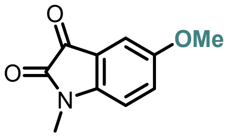

S5a

A modified literature procedure[2] was used for the synthesis of **S5a**. An oven-dried 100 mL round-bottom flask was charged with 5-methoxyisatin (2.44 g, 13.7 mmol, 1.00 eq.) and anhydrous DMF (55 mL). The resulting solution was cooled down to 0 °C and NaH (661 mg, 16.5 mmol, 1.2 eq., 60 wt% dispersion in mineral oil) was added in batches. After stirring for 5 min. at 0 °C, MeI (1.3 mL, 21 mmol, 1.5 eq.) was added. The reaction mixture was stirred for 1.5 h at 0 °C and then poured into ice-water and extracted with EtOAc (3 x 50 mL). The combined organic layers were washed with brine (50 mL), dried over anhydrous Na_2SO_4, and concentrated *in vacuo*. The residue was purified by recrystallisation from EtOAc (100 mL). The product was obtained as a dark red solid (2.44 g, 12.8 mmol, 93%). Spectroscopic data according to literature[4].

TLC: R_f (pentane/EtOAc 75:25) = 0.11

^{1}H NMR (400 MHz, $CDCl_3$) δ 7.19 – 7.12 (m, 2H), 6.81 (d, *J* = 9.3 Hz, 1H), 3.81 (s, 3H), 3.23 (s, 3H).

^{13}C NMR (151 MHz, $CDCl_3$) δ 183.9, 158.5, 156.7, 145.5, 124.8, 118.0, 111.0, 109.7, 56.1, 26.4.

HRMS (ESI$^+$) calcd for $C_{10}H_{10}NO_3^+$ [M+H]$^+$ 192.0655 found 192.0654.

6-methoxy-1-methylindolin-2-one – (S2).

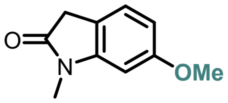

S2

Based on a literature procedure.[2] A 100 mL round-bottom flask was charged with **S2a** (1.68 g, 8.79 mmol, 1.00 eq.), water (9.9 mL) and hydrazine monohydrate (50-60%, 12.7 mL). The resulting mixture was heated to an external temperature of 165 °C for 6 h. After cooling down the reaction mixture to room temperature, the pH was adjusted to 2 with an aqueous HCl solution (4M) and the resulting mixture was stirred for 16 h at room temperature. Filtration provided the crude product as a red solid, which was purified by column chromatography (SiO_2, pentane/EtOAc 70:30). The product was obtained as off-white crystals (1.30 g, 7.33 mmol, 83%). Spectroscopic data according to literature[5].

TLC: R_f (pentane/EtOAc 70:30) = 0.34

^{1}H NMR (600 MHz, $CDCl_3$) δ 7.12 (td, J = 8.1, 1.2 Hz, 1H), 6.54 (dd, J = 8.1, 2.3 Hz, 1H), 6.41 (d, J = 2.3 Hz, 1H), 3.83 (s, 3H), 3.46 (s, 2H), 3.19 (s, 3H).

^{13}C NMR (151 MHz, $CDCl_3$) δ 176.0, 160.2, 146.6, 124.9, 116.4, 106.3, 96.4, 55.7, 35.3, 26.3.

HRMS (ESI$^+$) calcd for $C_{10}H_{12}NO_2^+$ [M+H]$^+$ 178.0863 found 178.0862.

5-methoxy-1-methylindolin-2-one – (S5).[2]

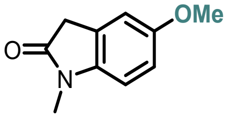

S5

Based on a literature procedure.[2] A 100 mL round-bottom flask was charged with **S5a** (2.20 g, 11.5 mmol, 1.00 eq.), water (12.9 mL) and hydrazine monohydrate (50-60%, 16.7 mL). The resulting mixture was heated to an external temperature of 165 °C for 6 h. After cooling down the reaction mixture to room temperature, the pH was adjusted to 2 with an aqueous HCl solution (4M) and the resulting mixture was stirred for 16 h at room temperature. Filtration provided the crude product as a dark red solid, which was purified by column chromatography (SiO_2, pentane/EtOAc 90:10 to 65:35). The product was obtained as a yellow solid (714 mg, 4.03 mmol, 35%). Spectroscopic data according to literature[6].

TLC: R_f (pentane/EtOAc 50:50) = 0.52
R_f (pentane/EtOAc 70:30) = 0.26

^{1}H NMR (400 MHz, $CDCl_3$) δ 6.88 (d, J = 2.5 Hz, 1H), 6.81 (dd, J = 8.4, 2.6 Hz, 1H), 6.71 (d, J = 8.4 Hz, 1H), 3.79 (s, 3H), 3.50 (s, 2H), 3.19 (s, 3H).

^{13}C NMR (151 MHz, $CDCl_3$) δ 174.8, 156.0, 139.0, 125.9, 112.3, 112.1, 108.4, 56.0, 36.3, 26.4.

6-bromo-1-methylindolin-2-one – (S3).

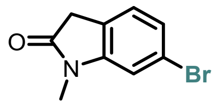

S3

A modified literature procedure[7] was used for the synthesis of **S3**. 6-Bromoindolin-2-one (4.24 g, 20.0 mmol, 1.00 eq.) was dissolved in dry toluene (50 mL). NaH (840 mg, 21.0 mmol, 1.05 eq., 60 wt% dispersion in mineral oil) was added and the resulting mixture was heated to 60 °C for 40 min. Dimethylsulfate (2.3 mL, 24 mmol, 1.2 eq.) was added dropwise. The reaction mixture was heated at 60 C for 3 h. After cooling down to room temperature, the reaction mixture was washed with brine (50 mL) and extracted with EtOAc (3 x 25 mL). The combined organic layers were dried over anhydrous $MgSO_4$ and concentrated *in vacuo*. The crude product was purified by column chromatography (SiO_2, pentane/EtOAc 98:2 to 75:25). The product was obtained as off-white crystals (2.98 g, 13.2 mmol, 66%). Spectroscopic data according to literature[8].

TLC: R_f (pentane/EtOAc 75:25) = 0.31

^{1}H NMR (600 MHz, $CDCl_3$) δ 7.17 (dd, *J* = 7.8, 1.7 Hz, 1H), 7.09 (dt, *J* = 7.9, 1.1 Hz, 1H), 6.96 (d, *J* = 1.7 Hz, 1H), 3.47 (d, *J* = 1.1 Hz, 2H), 3.19 (s, 3H).

^{13}C NMR (151 MHz, $CDCl_3$) δ 175.0, 146.7, 125.7, 125.2, 123.4, 121.6, 111.7, 35.5, 26.4.

6-fluoro-1-methylindolin-2-one – (S4).

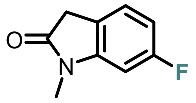

S4

A modified literature procedure[7] was used for the synthesis of **S4**. 6-Fluoroindolin-2-one (3.00 g, 19.8 mmol, 1.00 eq.) was dissolved in dry toluene (50 mL). NaH (834 mg, 20.8 mmol, 1.05 eq., 60 wt% dispersion in mineral oil) was added and the resulting mixture was heated to 60 °C for 25 min. Dimethylsulfate (2.3 mL, 24 mmol, 1.2 eq.) was added dropwise. The reaction mixture was heated at 60 C for 3 h. After cooling down to room temperature, the reaction mixture was washed with brine (50 mL) and extracted with EtOAc (3 x 25 mL). The combined organic layers were dried over anhydrous $MgSO_4$ and concentrated *in vacuo*. The crude product was purified by column chromatography (SiO_2, pentane/EtOAc 95:5 to 75:25). The product was obtained as a pink solid (2.64 g, 16.0 mmol, 80%). Spectroscopic data according to literature[9].

TLC: R_f (pentane/EtOAc 75:25) = 0.32

^{1}H NMR (400 MHz, $CDCl_3$) δ 7.16 (ddt, *J* = 8.0, 5.4, 1.2 Hz, 1H), 6.72 (ddd, *J* = 9.7, 8.0, 2.4 Hz, 1H), 6.56 (dd, *J* = 8.9, 2.4 Hz, 1H), 3.48 (s, 2H), 3.19 (s, 3H).

^{13}C NMR (101 MHz, $CDCl_3$) δ 175.6, 163.2 (d, $J_{C\text{-}F}$ = 244.3 Hz), 146.7 (d, $J_{C\text{-}F}$ = 11.8 Hz), 125.3 (d, $J_{C\text{-}F}$ = 9.5 Hz), 119.7 (d, $J_{C\text{-}F}$ = 2.9 Hz), 108.5 (d, $J_{C\text{-}F}$ = 22.4 Hz), 97.1 (d, $J_{C\text{-}F}$ = 27.5 Hz), 35.3, 26.5.

^{19}F NMR (376 MHz, $CDCl_3$) δ -112.76 – -112.85 (m).

5-bromo-1-methylindolin-2-one – (S6).[10]

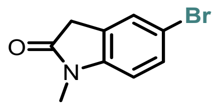

S6

Based on a literature procedure.[10] NBS (2.18 g, 12.2 mmol, 1.2 eq.) was added to a solution of *N*-methyl oxindole (1.50 g, 10.1 mmol, 1.00 eq.) in MeCN (50 mL) at 0 °C. The reaction mixture was warmed up to room temperature and stirred for 20 h. The solution was diluted with Et_2O and washed with water. The layers were separated and the aqueous phase was extracted with Et_2O (3 x 25 mL). The combined organic layers were dried over anhydrous Na_2SO_4 and concentrated *in vacuo*. Column chromatography (SiO_2, pentane/EtOAc 95:5 to 70:30) afforded the product was a slightly pink solid (1.95 g, 8.62 mmol, 85%). Spectroscopic data according to literature[10].

TLC: R_f (pentane/EtOAc 75:25) = 0.29

^1H NMR (400 MHz, $CDCl_3$) δ 7.41 (dd, J = 8.2, 2.0 Hz, 1H), 7.36 (d, J = 2.0 Hz, 1H), 6.69 (d, J = 8.2 Hz, 1H), 3.52 (s, 2H), 3.19 (s, 3H).

^{13}C NMR (101 MHz, $CDCl_3$) δ 174.5, 144.4, 130.9, 127.7, 126.6, 115.1, 109.5, 35.7, 26.4.

5-fluoro-1-methylindolin-2-one – (S7).

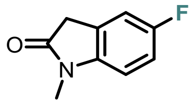

S7

Modified literature procedures[11,12] were used for the synthesis of **S7**. An oven-dried crimp top reaction vial was charged with 5-fluoroindolin-2-one (756 mg, 5.00 mmol, 1.00 eq.), anhydrous toluene (10 mL), and tetramethylammonium fluoride (TMAF) (2.12 g, 12.5 mmol, 2.50 eq.) The resulting suspension was heated at 120 °C for 6.5 h and then at 130 °C for 65 h. The reaction mixture was cooled down to room temperature and filtered through a celite plug and washed with EtOAc. The volatiles were removed under reduced pressure. Column chromatography (SiO_2, pentane/EtOAc 98:2 to 75:25) afforded the product was a yellow solid (314 mg, 1.90 mmol, 38%). Spectroscopic data according to literature[11].

TLC: R_f (pentane/EtOAc 85:15) = 0.69

^1H NMR (400 MHz, $CDCl_3$) δ 7.08 – 6.92 (m, 2H), 6.73 (dd, J = 8.3, 4.2 Hz, 1H), 3.53 (d, *J* = 1.3 Hz, 2H), 3.20 (s, 3H).

^{13}C NMR (101 MHz, $CDCl_3$) δ 174.8, 159.2 (d, $J_{C\text{-}F}$ = 239.9 Hz), 141.3 ($J_{C\text{-}F}$ = 1.4 Hz), 126.1 (d, $J_{C\text{-}F}$ = 9.0 Hz), 114.2 (d, $J_{C\text{-}F}$ = 23.4 Hz), 112.7 (d, $J_{C\text{-}F}$ = 24.9 Hz), 108.5 (d, $J_{C\text{-}F}$ = 8.5 Hz), 36.1 (d, $J_{C\text{-}F}$ = 1.9 Hz), 26.4.

^{19}F NMR (376 MHz, $CDCl_3$) -121.19 (td, *J* = 8.5, 4.1 Hz).

Motor syntheses

Double Knoevenagel Condensation - General procedure

A modified literature procedure[1] was used for the synthesis of **1**-**7**.

An oven-dried crimp top reaction vial under N_2 atmosphere was charged with **S1** (102 mg, 0.495 mmol, 1.0 equiv.) and dry THF (1.5 mL, 0.33 M), and cooled to 0 °C in an ice bath. $TiCl_4$ (0.16 mL, 1.48 mmol, 3.0 equiv.) was added dropwise and the resulting yellow suspension was stirred for 5 min at this temperature. A solution of *N*-methyl oxindole or functionalised *N*-methyl oxindole **S2-S7** (1.48 mmol, 3.0 equiv.) in dry THF (1.0-2.0 mL) was added to the reaction mixture, which was then stirred for 30 min at 0 °C. Subsequently, DBU (0.22 mL, 1.48 mmol, 3.0 equiv.) was added dropwise, and the resulting dark reaction mixture was stirred and heated at 40 °C for 16 h. Additional DBU (0.22 mL, 1.48 mmol, 3.0 equiv.) was added and the reaction mixture was heated at 40 °C for 24 h. The reaction was quenched with 1 M aqueous HCl solution and the mixture was extracted with EtOAc (3 x 10 mL). Note: as part of the work-up, sonication was used to extract all the material from the reaction vial. The combined organic layers were washed with brine (15 mL) and dried over $MgSO_4$. The volatiles were removed in vacuo to give a brown oil. The crude product was purified by flash column chromatography (SiO_2, pentane/EtOAc mixtures).

3,3'-((1,3)-2-Fluoro-2,4,7-trimethyl-1*H*-indene-1,3(2*H*)-diylidene)bis(1-methylindolin-2-one) – (1).

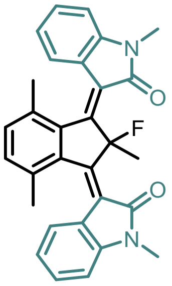

1

The title compound was obtained according to the general procedure from **S1** (102 mg, 0.495 mmol, 1.00 eq.) and *N*-methyl oxindole (218 mg, 1.48 mmol, 3.00 eq.). Purification by flash column chromatography (SiO_2, pentane/EtOAc 98:2 to 50:50) afforded **1** as pure (Z_SZ_S)-**1** and (E_SZ_S)-**1** isomers (Z_SZ_S:E_SZ_S = 54:46, 111 mg, 0.239 mmol, 48%). Spectroscopic data according to literature[1].

TLC: R_f (pentane/EtOAc 50:50) = 0.67 ((E_SZ_S)-**1**), 0.49 ((E_SE_S)-**1**), 0.31 ((Z_SZ_S)-**1**).

(Z_SZ_S)-**1**

^{1}H NMR (400 MHz, $CDCl_3$) δ 7.39 (s, 2H), 7.23 (td, *J* = 7.7, 1.2 Hz, 2H), 7.01 (dd, *J* = 7.7, 1.2 Hz, 2H), 6.87 (td, *J* = 7.6, 1.0 Hz, 2H), 6.78 (d, *J* = 7.8 Hz, 2H), 3.25 (s, 6H), 2.31 (d, *J* = 18.1 Hz, 3H), 2.26 (s, 6H).

^{19}F NMR (376 MHz, $CDCl_3$) δ -131.2 (q, *J* = 18.0 Hz)

(E_SZ_S)-**1**

^{1}H NMR (400 MHz, $CDCl_3$) δ 7.89 (dd, *J* = 7.7, 5.3 Hz, 1H), 7.39 (d, *J* = 8.0 Hz, 1H), 7.32 (d, *J* = 8.0 Hz, 1H), 7.26 (td, *J* = 7.7, 1.1 Hz, 1H), 7.23 (dd, *J* = 7.7, 1.2 Hz, 1H), 7.05 (dd, J = 7.8, 1.2 Hz, 1H), 7.03 (td, *J* = 7.7, 1.1 Hz, 1H), 6.89 (td, *J* = 7.6, 1.0 Hz, 1H), 6.79 (d, *J* = 5.6 Hz, 1H), 6.77 (d, *J* = 5.6 Hz, 1H), 3.27 (s, 3H), 3.24 (s, 3H), 2.38 (s, 3H), 2.22 (s, 3H), 2.18 (d, *J* = 18.0 Hz, 3H).

^{19}F NMR (376 MHz, $CDCl_3$) δ -135.4 (qd, *J* = 18.0, 5.3 Hz).

3,3'-((1,3)-2-Fluoro-2,4,7-trimethyl-1H-indene-1,3(2H)-diylidene)bis(6-methoxy-1-methylindolin-2-one) – (2).

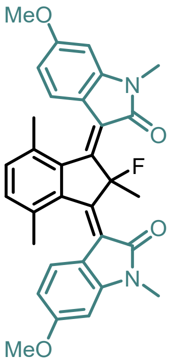

2

The title compound was obtained according to the general procedure from **S1** (102 mg, 0.495 mmol, 1.00 eq.) and **S2** (263 mg, 1.48 mmol, 3.00 eq.). Purification by flash column chromatography (SiO_2, pentane/EtOAc 98:2 to 50:50) afforded **2** as pure (Z_SZ_S)-**2** and (E_SZ_S)-**2** isomers (Z_SZ_S:E_SZ_S 65:54, 102 mg, 0.194 mmol, 39%). Defluorinated side product **2DeF** was also obtained as pure (Z_SZ_S)-**2DeF,** (E_SZ_S)-**2DeF** and (E_SE_S)-**2DeF** isomers (Z_SZ_S:E_SZ_S:E_SE_S 44:42:4, 45 mg, 89 µmol, 18%)

TLC: R_f (pentane/EtOAc 50:50) = 0.63 ((E_SZ_S)-**2DeF**), 0.56 ((E_SZ_S)-**2**), 0.44 ((E_SE_S)-**2DeF**), 0.29 ((Z_SZ_S)-**2DeF**), 0.19 ((Z_SZ_S)-**2**).

(Z_SZ_S)-**2**

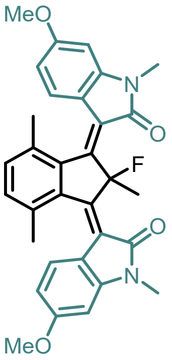

(Z_SZ_S)-**2**

^{1}H NMR (600 MHz, $CDCl_3$) δ 7.32 (s, 2H), 6.93 (d, *J* = 8.5 Hz, 2H), 6.39 (dd, *J* = 8.5, 2.3 Hz, 2H), 6.33 (d, *J* = 2.3 Hz, 2H), 3.83 (s, 6H), 3.23 (s, 6H), 2.29 (d, *J* = 18.1 Hz, 3H), 2.24 (s, 6H).

^{13}C NMR (151 MHz, $CDCl_3$) δ 166.7, 161.4, 152.2 (d, *J* = 18.7 Hz), 145.6, 141.4 (d, *J* = 4.3 Hz), 135.2 (d, *J* = 1.8 Hz), 134.2, 124.0 123.5, 116.6, 109.8 (d, *J* = 208.1 Hz), 105.8, 95.0, 55.7, 26.1, 21.6, 20.5 (d, *J* = 26.5 Hz).

^{19}F NMR (565 MHz, $CDCl_3$) δ -131.5 (q, J = 18.1 Hz).

HRMS (ESI$^+$) calcd for $C_{32}H_{30}FN_2O_4^+$ [M+H]$^+$ 525.2184 found 525.2187.

($E_S Z_S$)-**2**

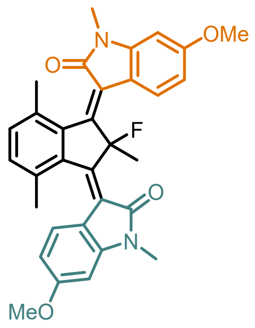

($E_S Z_S$)-**2**

^{1}H NMR (600 MHz, $CDCl_3$) δ 7.80 (dd, J = 8.6 , 5.1 Hz, 1H), 7.33 (d, J = 8.0 Hz, 1H), 7.27 (d, J = 8.0 Hz, 1H), 6.97 (d, J = 8.1 Hz, 1H), 6.54 (dd, J = 8.6, 2.3 Hz, 1H), 6.40 (dd, J = 8.6, 2.2 Hz, 1H), 6.36 (d, J = 2.2 Hz, 1H), 6.34 (d, J = 2.3 Hz, 1H), 3.86 (s, 3H), 3.83 (s, 3H), 3.24 (s, 3H), 3.22 (s, 3H), 2.35 (s, 3H), 2.20 (s, 3H), 2.14 (d, J = 18.2 Hz, 3H).

^{13}C NMR (151 MHz, $CDCl_3$) δ 167.2, 167.1, 161.4, 161.1, 151.1 (d, J = 18.1 Hz), 151.1 (d, J = 19.5 Hz), 145.2, 145.2, 140.9, 140.7, 137.9 (d, J = 1.7 Hz), 134.1, 133.9, 133.7, 128.3 (d, J = 13.8 Hz), 124.5, 122.9, 121.8, 116.5, 115.0, 108.6 (d, J = 207.1 Hz), 106.3, 105.9, 95.3, 95.3, 55.7, 55.7, 26.3, 26.1, 21.7, 21.5 (d, J = 26.0 Hz), 21.4.

^{19}F NMR (565 MHz, $CDCl_3$) δ -136.2 (td, J = 18.1, 5.0 Hz).

HRMS (ESI$^+$) calcd for $C_{32}H_{30}FN_2O_4^+$ [M+H]$^+$ 525.2184 found 525.2191.

($Z_S Z_S$)-**2DeF**

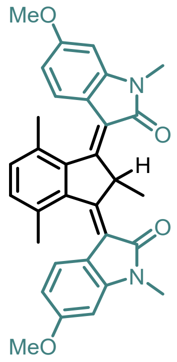

($Z_S Z_S$)-**2DeF**

^{1}H NMR (600 MHz, $CDCl_3$) δ 7.29 (s, 2H), 7.01 (d, J = 8.5 Hz, 2H), 6.43 (dd, J = 8.5, 2.3 Hz, 2H), 6.36 (d, J = 2.3 Hz, 2H), 6.02 (q, J = 6.5 Hz, 1H), 3.82 (s, 6H), 3.25 (s, 6H), 2.30 (s, 6H), 1.45 (d, J = 6.5 Hz, 3H).

^{13}C NMR (151 MHz, $CDCl_3$) δ 168.7, 160.8, 156.4, 144.6, 144.6, 134.7, 133.0, 124.3, 120.7, 115.9, 105.6, 95.0, 57.4, 55.6, 26.0, 21.3, 17.3.

HRMS (ESI$^+$) calcd for $C_{32}H_{31}N_2O_4^+$ [M+H]$^+$ 507.2278 found 507.2278.

(E_SZ_S)-**2DeF**

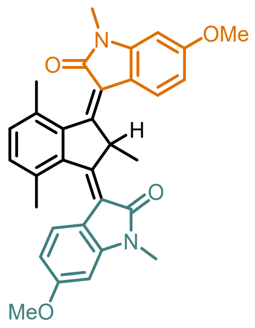

(E_SZ_S)-**2DeF**

^{1}H NMR (600 MHz, $CDCl_3$) δ 7.63 (d, J = 8.3 Hz, 1H), 7.32 (d, J = 8.0 Hz, 1H), 7.24 (d, J = 8.1 Hz, 1H), 7.01 (d, J = 8.5 Hz, 1H), 6.63 (dd, J = 8.4, 2.3 Hz, 1H), 6.44 (dd, J = 8.5, 2.3 Hz, 1H), 6.40 (d, J = 2.4 Hz, 1H), 6.35 (d, J = 2.3 Hz, 1H), 5.55 (q, J = 6.6 Hz, 1H), 3.86 (s, 3H), 3.82 (s, 3H), 3.24 (s, 3H), 3.22 (s, 3H), 2.41 (s, 3H), 2.26 (s, 3H), 1.52 (d, J = 6.6 Hz, 3H).

^{13}C NMR (151 MHz, $CDCl_3$) δ 169.4, 166.4, 160.8, 160.6, 155.6, 155.5, 144.6, 144.3, 143.6, 143.5, 138.0, 133.7, 133.1, 132.6, 124.9, 124.1, 120.4, 120.3, 116.8, 115.8, 106.3, 105.6, 95.7, 95.2, 58.0, 55.7, 55.6, 26.2, 26.0, 21.5, 21.4, 17.4.

HRMS (ESI$^+$) calcd for $C_{32}H_{31}N_2O_4^+$ [M+H]$^+$ 507.2278 found 507.2280.

3,3'-((1,3)-2-Fluoro-2,4,7-trimethyl-1H-indene-1,3(2H)-diylidene)bis(6-bromo-1-methylindolin-2-one) – (3).

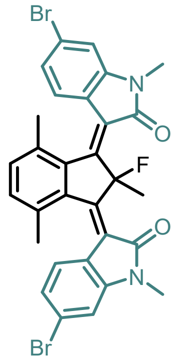

3

The title compound was obtained according to the general procedure from **S1** (102 mg, 0.495 mmol, 1.00 eq.) and **S3** (336 mg, 1.48 mmol, 3.00 eq.). Purification by flash column chromatography (SiO_2, pentane/EtOAc 98:2 to 50:50) afforded **3** as mixtures of (Z_SZ_S)-**3**, (E_SZ_S)-**3** and (E_SE_S)-**3** isomers (Z_SZ_S:E_SZ_S:E_SE_S 26:59:15, 154 mg, 0.247 mmol, 50%). Further purification of a mixed fraction by flash column chromatography (SiO_2, pentane/EtOAc 98:2 to 85:15) provided pure (E_SZ_S)-**3** and (E_SE_S)-**3** isomers (85 mg and 7 mg, respectively). Further purification of another mixed fraction by flash column chromatography (SiO_2, pentane/EtOAc 90:10 to 65:35) provided pure (Z_SZ_S)-**3** and (E_SZ_S)-**3** isomers (20 mg and 6 mg, respectively).

TLC: R_f (pentane/EtOAc 50:50) = 0.87 ((E_SZ_S)-**3**), 0.77 ((E_SE_S)-**3**), 0.63 ((Z_SZ_S)-**3**).
R_f (pentane/EtOAc 65:35) = 0.75 ((E_SZ_S)-**3**), 0.47 ((E_SE_S)-**3**), 0.38 ((Z_SZ_S)-**3**).
R_f (pentane/EtOAc 75:25) = 0.47 ((E_SZ_S)-**3**), 0.17 ((E_SE_S)-**3**), 0.08 ((Z_SZ_S)-**3**).
R_f (pentane/EtOAc 85:15) = 0.38 ((E_SZ_S)-**3**), 0.13 ((E_SE_S)-**3**), 0.03 ((Z_SZ_S)-**3**).

(Z_SZ_S)-**3**

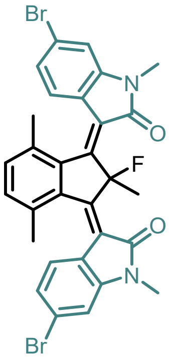

(Z_SZ_S)-**3**

^{1}H NMR (400 MHz, $CDCl_3$) δ 7.41 (s, 2H), 7.01 (dd, *J* = 8.2, 1.8 Hz, 2H), 6.94 (d, *J* = 1.8 Hz, 2H), 6.84 (d, *J* = 8.2 Hz, 2H), 3.24 (s, 6H), 2.28 (d, *J* = 18.1 Hz, 3H), 2.23 (s, 6H).

^{13}C NMR (151 MHz, $CDCl_3$) δ 165.7, 155.2 (d, *J* = 19.0 Hz), 144.9, 141.2 (d, *J* = 4.1 Hz), 135.7 (d, *J* = 1.7 Hz), 135.2, 124.1, 123.7, 123.2, 123.2, 122.2, 111.4, 109.8 (d, *J* = 209.9 Hz), 26.2, 21.7, 20.2 (d, *J* = 26.0 Hz).

^{19}F NMR (376 MHz, $CDCl_3$) δ -131.5 (q, *J* = 18.3 Hz).

HRMS (ESI$^+$) calcd for $C_{30}H_{24}Br_2FN_2O_2^+$ [M+H]$^+$ 621.0183 found 621.0191.

(E_SZ_S)-**3**

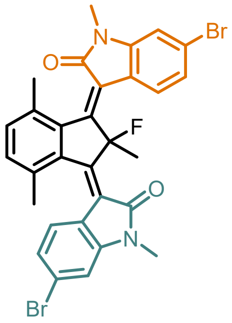

(E_SZ_S)-**3**

^{1}H NMR (400 MHz, $CDCl_3$) δ 7.71 (dd, *J* = 8.3, 5.0 Hz, 1H), 7.41 (d, *J* = 8.0 Hz, 1H), 7.34 (d, *J* = 8.0 Hz, 1H), 7.16 (dd, *J* = 8.3, 1.8 Hz, 1H), 7.03 (dd, *J* = 8.2, 1.8 Hz, 1H), 6.95 (d, *J* = 1.8 Hz, 1H), 6.94 (d, *J* = 1.8 Hz, 1H), 6.88 (d, *J* = 8.2 Hz, 1H), 3.25 (s, 3H), 3.23 (s, 3H), 2.36 (s, 3H), 2.18 (s, 3H), 2.14 (d, *J* = 17.9 Hz, 3H).

^{13}C NMR (101 MHz, $CDCl_3$) δ 166.1, 166.0, 154.1 (d, *J* = 18.6 Hz), 153.9 (d, *J* = 19.5 Hz), 144.6, 144.5, 140.8 (d, *J* = 4.6 Hz), 140.4 (d, *J* = 4.1 Hz), 138.7 (d, *J* = 1.9 Hz), 134.7, 134.6 (d, *J* = 1.8 Hz), 134.5, 128.1 (d, *J* = 14.5 Hz), 124.9 (d, *J* = 2.3 Hz), 124.4, 124.2, 123.1, 123.1, 122.5 (d, *J* = 0.8 Hz), 122.1 (d, *J* = 0.7 Hz), 121.5, 120.6 (d, *J* = 1.2 Hz), 111.4, 111.3, 108.5 (d, *J* = 208.7 Hz), 26.4, 26.2, 21.8, 21.5 (d, *J* = 25.2 Hz), 21.4.

^{19}F NMR (376 MHz, $CDCl_3$) δ -135.4 (qd, *J* = 18.0, 5.0 Hz).

HRMS (ESI$^+$) calcd for $C_{30}H_{24}Br_2FN_2O_2^+$ [M+H]$^+$ 621.0183 found 621.0186.

(E_SE_S)-**3**

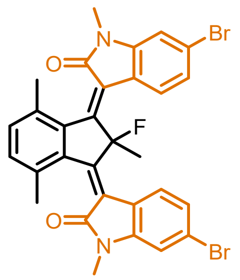

(E_SZ_S)-**3**

^{1}H NMR (600 MHz, $CDCl_3$) δ 7.58 (dd, *J* = 8.3, 5.4 Hz, 2H), 7.34 (s, 2H), 7.18 (dd, *J* = 8.3, 1.8 Hz, 2H), 6.95 (d, *J* = 1.8 Hz, 2H), 3.21 (s, 6H), 2.33 (s, 6H), 2.01 (d, *J* = 18.1 Hz, 6H).

^{13}C NMR (151 MHz, $CDCl_3$) δ 165.6, 152.7 (d, *J* = 19.8 Hz), 144.7, 140.2 (d, *J* = 4.7 Hz), 137.4 (d, *J* = 1.7 Hz), 133.9, 127.4 (d, *J* = 14.3 Hz), 124.5 (d, *J* = 2.1 Hz), 122.9, 120.8, 120.6 (d, *J* = 1.6 Hz), 111.3, 107.5 (d, *J* = 207.4 Hz), 26.4, 23.0 (d, *J* = 24.6 Hz), 21.3.

^{19}F NMR (565 MHz, $CDCl_3$) δ -137.8 (qt, *J* = 18.1, 5.4 Hz).

HRMS (ESI$^+$) calcd for $C_{30}H_{24}Br_2FN_2O_2^+$ $[M+H]^+$ 621.0183 found 621.0190.

3,3'-((1,3)-2-Fluoro-2,4,7-trimethyl-1H-indene-1,3(2H)-diylidene)bis(6-fluoro-1-methylindolin-2-one) – (4).

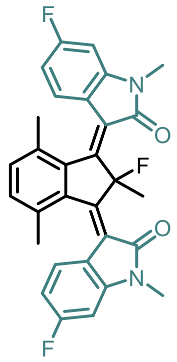

4

The title compound was obtained according to the general procedure from **S1** (102 mg, 0.495 mmol, 1.00 eq.) and **S4** (245 mg, 1.48 mmol, 3.00 eq.). Purification by flash column chromatography (SiO_2, pentane/EtOAc 98:2 to 65:35) afforded **4** as pure (Z_SZ_S)-**4** and (E_SZ_S)-**4** isomers (Z_SZ_S:E_SZ_S 31:69, 160 mg, 0.320 mmol, 65%)

TLC: R_f (pentane/EtOAc 75:25) = 0.59 ((E_SZ_S)-**4**), 0.13 ((Z_SZ_S)-**4**).

(Z_SZ_S)-**4**

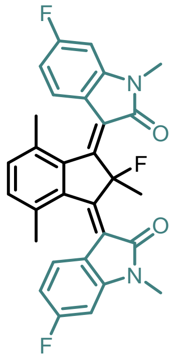

(Z_SZ_S)-**4**

^{1}H NMR (400 MHz, $CDCl_3$) δ 7.39 (s, 2H), 6.95 (dd, *J* = 8.4, 5.4 Hz, 2H), 6.56 (ddd, *J* = 9.3, 8.5, 2.4 Hz, 2H), 6.51 (dd, *J* = 8.9, 2.4 Hz, 2H), 3.24 (s, 6H), 2.29 (d, *J* = 18.2 Hz, 3H), 2.24 (s, 6H).

^{13}C NMR (151 MHz, $CDCl_3$) δ 166.1, 164.0 (d, *J* = 248.6 Hz), 154.2 (dd, *J* = 18.9, 2.7 Hz), 145.6 (d, *J* = 11.5 Hz), 141.2 (d, *J* = 4.2 Hz), 135.5 (d, *J* = 2.1 Hz), 134.9, 124.0 (d, *J* = 9.7 Hz), 123.0 (d, *J* = 2.5 Hz), 119.3 (d, *J* = 3.2 Hz), 109.7 (d, *J* = 209.2 Hz), 107.6 (d, *J* = 22.9 Hz), 96.5 (d, *J* = 27.4 Hz), 26.2, 21.7, 20.3 (d, *J* = 25.9 Hz).

^{19}F NMR (376 MHz, $CDCl_3$) δ -108.8 (td, *J* = 9.2, 5.5 Hz), -131.3 (q, *J* = 18.0 Hz).

HRMS (ESI$^+$) calcd for $C_{30}H_{24}F_3N_2O_2^+$ [M+H]$^+$ 501.1784 found 501.1782.

(E_SZ_S)-**4**

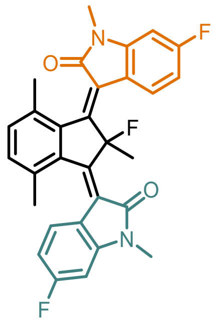

(E_SZ_S)-**4**

^{1}H NMR (400 MHz, $CDCl_3$) δ 7.82 (dt, *J* = 8.6, 5.3 Hz, 1H), 7.39 (d, *J* = 8.0 Hz, 1H), 7.32 (d, *J* = 8.0 Hz, 1H), 6.99 (dd, *J* = 8.5, 5.4 Hz, 1H), 6.72 (ddd, *J* = 9.5, 8.6, 2.5 Hz, 1H), 6.58 (ddd, *J* = 9.4, 8.5, 2.4 Hz, 1H), 6.53 (dd, *J* = 8.8, 2.5 Hz, 1H), 6.52 (dd, *J* = 8.8, 2.4 Hz, 1H), 3.25 (s, 3H), 3.22 (s, 3H), 2.37 (s, 3H), 2.20 (s, 3H), 2.14 (d, *J* = 18.1 Hz, 3H).

^{13}C NMR (101 MHz, $CDCl_3$) δ 166.6, 166.6, 163.9 (d, *J* = 248.4 Hz), 163.7 (d, *J* = 248.8 Hz), 153.2 – 152.7 (m, 2C), 145.2 (d, *J* = 11.7 Hz), 145.1 (d, *J* = 11.3 Hz), 140.8 (d, *J* = 5.0 Hz), 140.4 (d, *J* = 4.3 Hz), 138.5 (d, *J* = 1.5 Hz), 134.4, 134.4, 134.2, 128.5 (dd, *J* = 14.7, 9.1 Hz), 124.5 (d, *J* = 9.4 Hz), 122.4, 121.3, 119.2, 117.7 (d, *J* = 2.4 Hz), 108.5 (d, *J* = 207.3 Hz), 108.3 (dd, *J* = 22.6, 1.8 Hz), 108.0 (d, *J* = 23.0 Hz), 96.6 (d, *J* = 27.5 Hz), 96.3 (d, *J* = 27.3 Hz), 26.5, 26.2, 21.7, 21.5 (d, *J* = 25.3 Hz), 21.4.

^{19}F NMR (376 MHz, $CDCl_3$) δ -109.0 (td, *J* = 9.0, 5.4 Hz), -110.0 (td, *J* = 9.0, 5.3 Hz), -135.4 (qd, *J* = 18.1, 4.9 Hz).

HRMS (ESI$^+$) calcd for $C_{30}H_{24}F_3N_2O_2^+$ [M+H]$^+$ 501.1784 found 501.1782.

3,3'-((1,3)-2-Fluoro-2,4,7-trimethyl-1H-indene-1,3(2H)-diylidene)bis(5-methoxy-1-methylindolin-2-one) – (5).

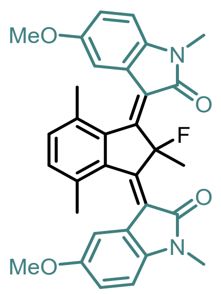

5

The title compound was obtained according to the general procedure from **S1** (102 mg, 0.495 mmol, 1.00 eq.) and **S5** (263 mg, 1.48 mmol, 3.00 eq.). Purification by flash column chromatography (SiO_2, pentane/EtOAc 98:2 to 40:60) afforded **5** as pure (Z_SZ_S)-**5**, (E_SZ_S)-**5** and (E_SE_S)-**5** isomers (Z_SZ_S:E_SZ_S:E_SE_S 16:76:8, 165 mg, 0.315 mmol, 64%).

TLC: R_f (pentane/EtOAc 50:50) = 0.49 ((E_SZ_S)-**5**), 0.35 ((E_SE_S)-**5**), 0.17 ((Z_SZ_S)-**5**).

(Z_SZ_S)-**5**

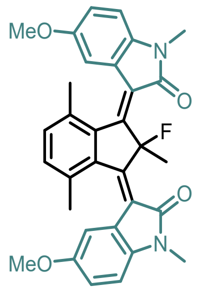

(Z_SZ_S)-**5**

^{1}H NMR (600 MHz, $CDCl_3$) δ 7.38 (s, 2H), 6.80 (dd, *J* = 8.4, 2.5 Hz, 2H), 6.67 (d, *J* = 8.4 Hz, 2H), 6.63 (d, *J* = 2.4 Hz, 2H), 3.70 (s, 6H), 3.22 (s, 6H), 2.30 (d, *J* = 18.0 Hz, 3H), 2.29 (s, 6H).

^{13}C NMR (151 MHz, $CDCl_3$) δ 166.0, 155.0, 155.0 (d, *J* = 19.0 Hz), 141.3 (d, *J* = 3.9 Hz), 138.2, 135.7 (d, *J* = 1.2 Hz), 134.9, 124.5, 124.4, 113.9, 110.4, 109.8 (d, *J* = 209.8 Hz), 107.9, 56.1, 26.1, 21.8, 20.4 (d, *J* = 26.0 Hz).

^{19}F NMR (565 MHz, $CDCl_3$) δ -130.9 (q, *J* = 18.1 Hz).

HRMS (ESI$^+$) calcd for $C_{32}H_{30}FN_2O_4^+$ [M+H]$^+$ 525.2184 found 525.2191.

(E_SZ_S)-**5**

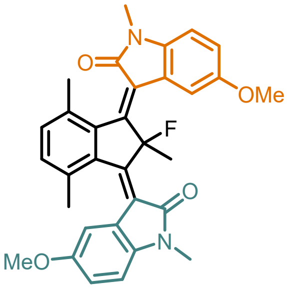

(E_SZ_S)-**5**

^{1}H NMR (600 MHz, CDCl$_3$) δ 7.50 (dd, J = 4.7, 2.5 Hz, 1H), 7.38 (d, J = 8.0 Hz, 1H), 7.33 (d, J = 8.0 Hz, 1H), 6.83 (dd, J = 8.4, 2.5 Hz, 1H), 6.80 (dd, J = 8.4, 2.5 Hz, 1H), 6.69 (d, J = 8.5 Hz, 1H), 6.67 (d, J = 8.3 Hz, 1H), 6.67 (d, J = 2.7 Hz, 1H), 3.83 (s, 3H), 3.66 (s, 3H), 3.24 (s, 3H), 3.22 (s, 3H), 2.38 (s, 3H), 2.25 (s, 3H), 2.17 (d, J = 18.0 Hz, 3H).

^{13}C NMR (151 MHz, CDCl$_3$) δ 166.3, 166.2, 155.4 (d, J = 1.1 Hz), 155.3, 153.8 (d, J = 19.1 Hz), 153.8 (d, J = 19.5 Hz), 141.0 (d, J = 4.9 Hz), 140.6 (d, J = 4.3 Hz), 138.5, 138.5, 137.7, 134.5 (d, J = 1.7 Hz), 134.5, 134.2, 124.2, 123.9, 122.9, 122.8 (d, J = 1.1 Hz), 114.9, 114.4 (d, J = 14.2 Hz), 114.1, 110.0, 108.4 (d, J = 208.9 Hz), 108.1, 107.8, 56.2, 56.2, 26.4, 26.2, 21.8, 21.4 (d, J = 25.4 Hz), 21.4.

^{19}F NMR (565 MHz, CDCl$_3$) δ -135.0 (qd, J = 18.0, 4.9 Hz).

HRMS (ESI$^+$) calcd for $C_{32}H_{30}FN_2O_4^+$ [M+H]$^+$ 525.2184 found 525.2188.

(E_SE_S)-**5**

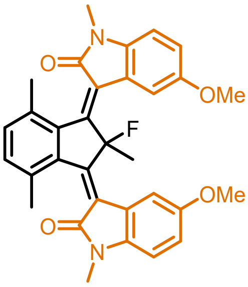

(E_SE_S)-**5**

^{1}H NMR (600 MHz, CDCl$_3$) δ 7.39 (dd, J = 5.2, 2.5 Hz, 2H), 7.31 (s, 2H), 6.83 (dd, J = 8.5, 2.5 Hz, 2H), 6.68 (d, J = 8.5 Hz, 2H), 3.85 (s, 2H), 3.20 (s, 2H), 2.34 (s, 2H), 2.03 (d, J = 18.0 Hz, 1H).

^{13}C NMR (151 MHz, CDCl$_3$) δ 165.8, 155.0, 152.4 (d, J = 19.2 Hz), 140.4 (d, J = 4.4 Hz), 138.0, 137.2 (d, J = 1.7 Hz), 133.6, 122.8 (d, J = 0.8 Hz), 122.2, 114.1 (d, J = 14.3 Hz), 113.4, 107.8, 107.3 (d, J = 208.3 Hz), 56.1, 26.4, 22.8 (d, J = 24.7 Hz), 21.3.

^{19}F NMR (565 MHz, CDCl$_3$) δ -137.0 (qt, J = 18.0, 5.4 Hz).

HRMS (ESI$^+$) calcd for $C_{32}H_{30}FN_2O_4^+$ [M+H]$^+$ 525.2184 found 525.2179.

3,3'-((1,3)-2-Fluoro-2,4,7-trimethyl-1H-indene-1,3(2H)-diylidene)bis(5-bromo-1-methylindolin-2-one) – (6).

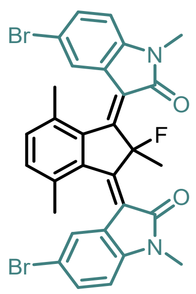

6

The title compound was obtained according to the general procedure from **S1** (102 mg, 0.495 mmol, 1.00 eq.) and **S6** (336 mg, 1.48 mmol, 3.00 eq.). Purification by flash column chromatography (SiO_2, pentane/EtOAc 100:0 to 55:45) afforded **6** as (Z_SZ_S)-**6**, (E_SZ_S)-**6** and (E_SE_S)-**6** isomers (Z_SZ_S:E_SZ_S:E_SE_S 11:46:43, 193 mg, 0.307 mmol, 63%).

TLC: R_f (pentane/EtOAc 75:25) = 0.42 ((E_SZ_S)-**6**), 0.19 ((E_SE_S)-**6**), 0.07 ((Z_SZ_S)-**6**).

(Z_SZ_S)-**6**

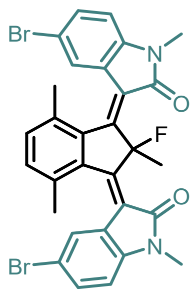

(Z_SZ_S)-**6**

(Z_SZ_S)-**6** was characterised from a mixture of (Z_SZ_S)-**6** (64%), (E_SZ_S)-**6** (29%) and (E_SE_S)-**6** (7%).

^{1}H NMR (600 MHz, $CDCl_3$) δ 7.47 (s, 2H), 7.36 (dd, *J* = 8.3, 1.9 Hz, 2H), 7.11 (*d*, J = 1.9 Hz, 2H), 6.67 (d, *J* = 8.3 Hz, 2H), 3.24 (s, 6H), 2.28 (d, *J* = 18.2 Hz, 3H), 2.27 (s, 6H).

^{13}C NMR (151 MHz, $CDCl_3$) δ 165.4, 156.0 (d, *J* = 19.0 Hz), 142.7, 141.0 (d, *J* = 4.2 Hz), 135.9 (d, *J* = 1.8 Hz), 135.5, 132.1, 125.6, 124.9, 123.0, 113.9, 109.5 (d, *J* = 210.2 Hz), 109.3, 26.2, 21.9, 20.2 (d, *J* = 25.5 Hz).

^{19}F NMR (565 MHz, $CDCl_3$) δ -131.4 (q, *J* = 18.0 Hz).

HRMS (ESI$^+$) calcd for $C_{30}H_{24}Br_2FN_2O_2^+$ [M+H]$^+$ 621.0183 found 621.0190.

(E_SZ_S)-**6**

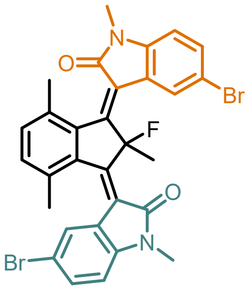

(E_SZ_S)-**6**

^{1}H NMR (400 MHz, $CDCl_3$) δ 7.92 (dd, J = 4.6, 1.9 Hz, 1H), 7.44 (d, J = 8.1 Hz, 1H), 7.39 (d, J = 8.5 Hz, 1H), 7.39 (dd, J = 8.3, 1.9 Hz, 1H), 7.35 (dd, J = 8.3, 1.9 Hz, 1H), 7.11 (d, J = 1.9 Hz, 1H), 6.69 (d, J = 8.3 Hz, 1H), 6.67 (d, J = 8.3 Hz, 1H), 3.26 (s, 3H), 3.24 (s, 3H), 2.37 (s, 3H), 2.22 (s, 3H), 2.15 (d, J = 18.1 Hz, 3H).

^{13}C NMR (151 MHz, $CDCl_3$) δ 165.8, 165.8, 154.9 (d, J = 18.9 Hz), 154.9 (d, J = 18.7 Hz), 142.4, 142.3, 140.8 (d, J = 2.3 Hz), 140.2 (dd, J = 5.3, 2.4 Hz), 138.8, 134.9, 134.8, 134.7, 132.0, 131.8, 129.5 (d, J = 13.9 Hz), 125.8, 125.0, 123.5, 122.4, 121.5, 114.8, 114.3, 109.3, 109.2, 108.4 (d, J = 209.5 Hz), 26.4, 26.2, 21.8, 21.7 (d, J = 27.0 Hz), 21.3.

^{19}F NMR (376 MHz, $CDCl_3$) δ -135.2 (qd, J = 18.2, 4.6 Hz).

HRMS (ESI$^+$) calcd for $C_{30}H_{24}Br_2FN_2O_2^+$ $[M+H]^+$ 621.0183 found 621.0191.

(E_SE_S)-**6**

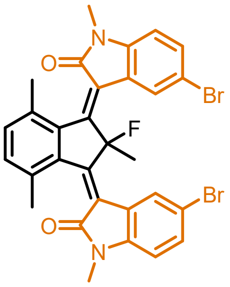

(E_SE_S)-**6**

^{1}H NMR (400 MHz, $CDCl_3$) δ 7.83 (dd, J = 5.1, 1.9 Hz, 2H), 7.40 (dd, J = 8.3, 1.9 Hz, 2H), 7.35 (s, 2H), 6.68 (d, J = 8.3 Hz, 2H), 3.22 (s, 6H), 2.33 (s, 6H), 2.04 (d, J = 18.2 Hz, 3H).

^{13}C NMR (151 MHz, $CDCl_3$) δ 165.4, 153.4 (d, J = 19.2 Hz), 142.5, 140.2 (d, J = 4.8 Hz), 137.5 (d, J = 1.9 Hz), 134.0, 131.6, 129.1 (d, J = 14.8 Hz), 123.4, 120.8, 114.5 (d, J = 2.1 Hz), 109.2, 107.5 (d, J = 208.4 Hz), 26.4, 23.3 (d, J = 24.4 Hz), 21.3.

^{19}F NMR (376 MHz, $CDCl_3$) δ -137.1 (qt, J = 18.1, 5.0 Hz).

HRMS (ESI$^+$) calcd for $C_{30}H_{24}Br_2FN_2O_2^+$ $[M+H]^+$ 621.0183 found 621.0185.

3,3'-((1,3)-2-Fluoro-2,4,7-trimethyl-1H-indene-1,3(2H)-diylidene)bis(5-fluoro-1-methylindolin-2-one) – (7).

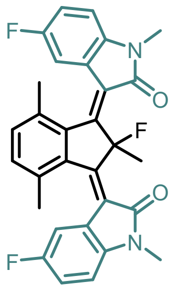

7

The title compound was obtained according to the general procedure from **S1** (102 mg, 0.495 mmol, 1.00 eq.) and **S7** (245 mg, 1.48 mmol, 3.00 eq.). Purification by flash column chromatography (SiO_2, pentane/EtOAc 95:5 to 50:50) afforded **7** as (Z_SZ_S)-**7**, (E_SZ_S)-**7** and (E_SE_S)-**7** isomers (Z_SZ_S:E_SZ_S:E_SE_S 7:50:43, 192 mg, 0.384 mmol, 78%).

TLC: R_f (pentane/EtOAc 75:25) = 0.55 ((E_SZ_S)-**7**), 0.16 ((E_SE_S)-**7**), 0.03 ((Z_SZ_S)-**7**).

(Z_SZ_S)-**7**

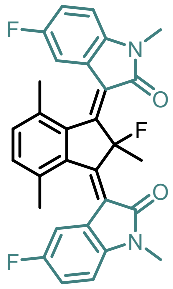

(Z_SZ_S)-**7**

(Z_SZ_S)-**7** was characterised from a mixture of (Z_SZ_S)-**7** (72%) and (E_SZ_S)-**7** (28%).

^{1}H NMR (400 MHz, $CDCl_3$) δ 7.45 (s, 2H), 6.96 (td, *J* = 8.7, 2.5 Hz, 2H), 6.74 (dd, *J* = 9.0, 2.6 Hz, 2H), 6.69 (dd, *J* = 8.6, 4.4 Hz, 2H), 3.24 (s, 6H), 2.30 (d, *J* = 17.9 Hz, 3H), 2.29 (s, 6H).

^{13}C NMR (151 MHz, $CDCl_3$) δ 165.8, 158.6 (d, *J* = 237.7 Hz), 155.8 (d, *J* = 19.3 Hz), 141.0 (d, *J* = 4.2 Hz), 140.1 (d, *J* = 1.4 Hz), 135.8 (d, *J* = 1.6 Hz), 135.4, 124.2 (d, *J* = 9.3 Hz), 124.0 (d, *J* = 2.9 Hz), 115.8 (d, *J* = 23.9 Hz), 110.1 (d, *J* = 26.0 Hz, 1H), 109.7 (d, *J* = 210.2 Hz), 108.2 (d, *J* = 8.4 Hz), 26.2, 21.6, 20.4 (d, *J* = 26.1 Hz).

^{19}F NMR (376 MHz, $CDCl_3$) δ -122.3 (td, *J* = 9.0, 4.3 Hz), -131.0 (q, *J* = 18.1 Hz).

HRMS (ESI^+) calcd for $C_{30}H_{24}F_3N_2O_2^+$ $[M+H]^+$ 501.1784 found 501.1783.

(E_SZ_S)-**7**

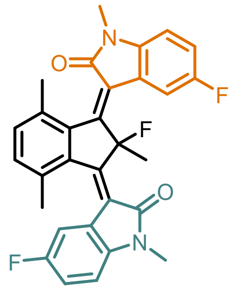

(E_SZ_S)-**7**

^{1}H NMR (600 MHz, $CDCl_3$) δ 7.60 (ddd, *J* = 9.3, 4.7, 2.5 Hz, 1H), 7.43 (d, *J* = 8.0 Hz, 1H), 7.37 (d, *J* = 8.0 Hz, 1H), 7.02 – 6.92 (m, 2H), 6.77 (dd, *J* = 8.9, 2.6 Hz, 1H), 6.73 – 6.68 (m, 2H), 3.26 (s, 3H), 3.23 (s, 3H), 2.38 (s, 3H), 2.24 (s, 3H), 2.16 (d, *J* = 18.3 Hz, 3H).

^{13}C NMR (151 MHz, $CDCl_3$) δ 166.2, 166.0, 158.7 (d, *J* = 238.5 Hz), 158.7 (dd, *J* = 238.0, 1.8 Hz), 154.7 (d, *J* = 18.6 Hz), 154.5 (d, *J* = 19.3 Hz), 140.8 (d, *J* = 4.3 Hz), 140.2 (d, *J* = 3.9 Hz), 139.7 (dd, *J* = 5.3, 1.1 Hz), 138.8 (d, *J* = 1.5 Hz), 134.8, 134.7, 134.7, 134.6 (d, *J* = 1.7 Hz), 124.2 (d, *J* = 9.4 Hz), 123.2 (d, *J* = 2.8 Hz), 122.7 (d, *J* = 9.3 Hz), 122.2 (d, *J* = 2.9 Hz), 115.8 (d, *J* = 24.0 Hz), 115.5 (d, *J* = 24.0 Hz), 114.6 (dd, *J* = 26.2, 14.6 Hz), 110.5 (d, *J* = 25.9 Hz), 108.5 (d, *J* = 208.8 Hz), 108.2 (d, *J* = 8.1 Hz), 108.0 (d, *J* = 8.4 Hz), 26.4, 26.2, 21.7, 21.6 (d, *J* = 25.5 Hz), 21.4.

^{19}F NMR (376 MHz, $CDCl_3$) δ -121.4 (td, *J* = 9.2, 4.7 Hz), -121.9 (td, *J* = 8.9, 4.2 Hz), -134.8 (qd, *J* = 18.2, 4.7 Hz).

HRMS (ESI$^+$) calcd for $C_{30}H_{24}F_3N_2O_2^+$ $[M+H]^+$ 501.1784 found 501.1780.

(E_SE_S)-**7**

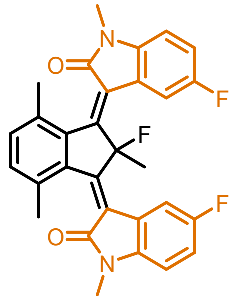

(E_SE_S)-**7**

^{1}H NMR (400 MHz, $CDCl_3$) δ 7.50 (ddd, *J* = 9.5, 5.0, 2.4 Hz, 2H), 7.34 (s, 2H), 7.00 (td, *J* = 8.8, 2.5 Hz, 2H), 6.71 (dd, *J* = 8.5, 4.4 Hz, 2H), 3.22 (s, 6H), 2.34 (s, 6H), 2.02 (d, *J* = 18.2 Hz, 3H).

^{13}C NMR (151 MHz, $CDCl_3$) δ 165.7, 158.6 (d, *J* = 239.2 Hz), 153.1 (d, *J* = 19.5 Hz), 140.1 (d, *J* = 4.4 Hz), 139.8, 137.5, 134.0, 122.6 (d, *J* = 8.7 Hz), 121.5, 115.3 (d, *J* = 24.3 Hz), 113.9 (dd, *J* = 26.2, 15.0 Hz), 108.0 (d, *J* = 8.4 Hz), 107.5 (d, *J* = 207.9 Hz), 26.5, 23.0 (d, *J* = 24.6 Hz), 21.3.

^{19}F NMR (376 MHz, $CDCl_3$) δ -122.0 (td, *J* = 9.2, 4.4 Hz), -136.8 (qt, *J* = 18.0, 5.1 Hz).

HRMS (ESI$^+$) calcd for $C_{30}H_{23}F_3N_2O_2Na^+$ $[M+Na]^+$ 523.1604 found 523.1608.

Post-functionalisation

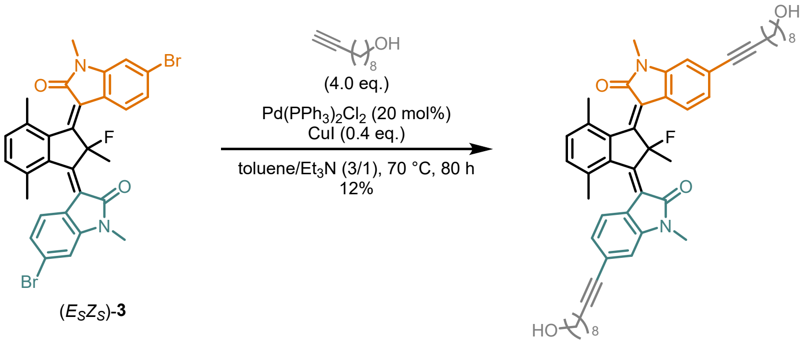

***Figure S3**. Synthesis of membrane motor (E_SZ_S)-**8** via a Sonogashira reaction of (E_SZ_S)-**3** and dec-9-yn-1-ol.*

3,3'-((1*Z*,3*E*)-2-fluoro-2,4,7-trimethyl-1H-indene-1,3(2H)-diylidene)bis(6-(10-hydroxydec-1-yn-1-yl)-1-methylindolin-2-one) – (8).

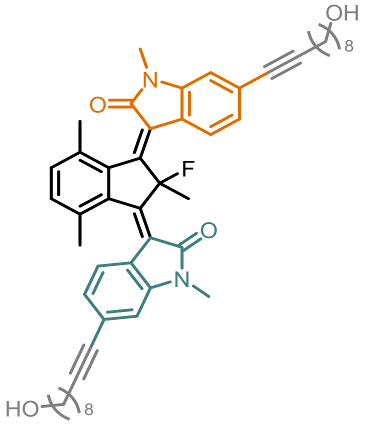

(E_SZ_S)-**8**

A oven-dried Schlenk flask was charged with (E_SZ_S)-**3** (20 mg, 32 µmol, 1.0 eq.), dec-9-yn-1-ol (20 mg, 0.13 mmol, 4.0 eq.), $Pd(PPh)_3Cl_2$ (4.5 mg, 6.4 µmol, 0.2 eq.) and CuI (2.1 mg, 13 µmol, 0.4 eq.). Toluene (0.75 mL) and Et_3N (0.25 mL) were added and the resulting reaction mixture was subjected to three freeze-pump-thaw cycles. The reaction mixture was stirred at 70 °C for 80 h (in the dark to prevent photoisomerisation of the motor unit). After cooling down to r.t., the reaction mixture was diluted with EtOAc and filtered through celite. The organic layer was washed with brine (3 x 5 mL) and dried over anhydrous Na_2SO_4. The crude product was concentrated *in vacuo* and purification by flash column chromatography (SiO_2, CH_2Cl_2/MeOH 100:0 to 90:10) provided the product (E_SZ_S)-**8** (3 mg, 3.9 µmol, 12%), the mono-functionalised intermediates (as a mixture of *E*- and *Z*-functionalised motors, 7 mg, 10 µmol, 31%) and remaining starting material (2 mg, 3.2 µmol, 10%).

TLC: R_f (CH_2Cl_2/MeOH 98:2) = 0.94 ((E_SZ_S)-**3**), 0.54 (mono *E* and mono *Z*), 0.30 ((E_SZ_S)-**8**).

^{1}H NMR (400 MHz, $CDCl_3$) δ 7.78 (dd, *J* = 8.1, 5.1 Hz, 1H), 7.38 (d, *J* = 8.0 Hz, 1H), 7.32 (d, *J* = 7.9 Hz, 1H), 7.06 (dd, *J* = 8.1, 1.5 Hz, 1H), 6.93 (app. s, 2H), 6.81 (app. d, J = 3.6 Hz, 2H), 3.65 (t, *J* = 6.6 Hz, 2H), 3.64 (t, *J* = 6.6 Hz, 2H), 3.25 (s, 3H), 3.22 (s, 3H), 2.43 (t, *J* =, 7.1 Hz, 2H), 2.41 (t, *J* =, 7.1 Hz, 2H), 2.37 (s, 3H), 2.18 (s, 4H), 2.15 (d, J = 17.9 Hz, 3H), 1.75 – 1.29 (m, 24H).

^{13}C NMR (151 MHz, $CDCl_3$) δ 166.5, 166.3, 153.9, 153.6, 143.4, 143.3, 141.0, 140.6 (d, J = 3.3 Hz), 138.5, 134.6, 134.5, 134.3, 125.6, 125.2, 124.9, 124.6, 123.0, 122.9, 122.7, 122.0, 121.1, 110.7, 110.6,

108.5 (d, *J* = 209.0 Hz), 92.4, 92.4, 81.2, 81.2, 63.2, 63.2, 32.9, 32.9, 29.9, 29.8, 29.4, 29.3, 29.0, 28.8, 26.3, 26.1, 25.9, 21.7, 21.5 (d, *J* = 25.2 Hz), 21.4, 19.7, 19.7.

^{19}F NMR (376 MHz, $CDCl_3$) δ -135.6 (qd, *J* = 18.1, 5.1 Hz).

HRMS (ESI$^+$) calcd for $C_{50}H_{58}FN_2O_4^+$ [M+H]$^+$ 769.4375 found 769.4359.

3. UV/Vis Absorption Spectra

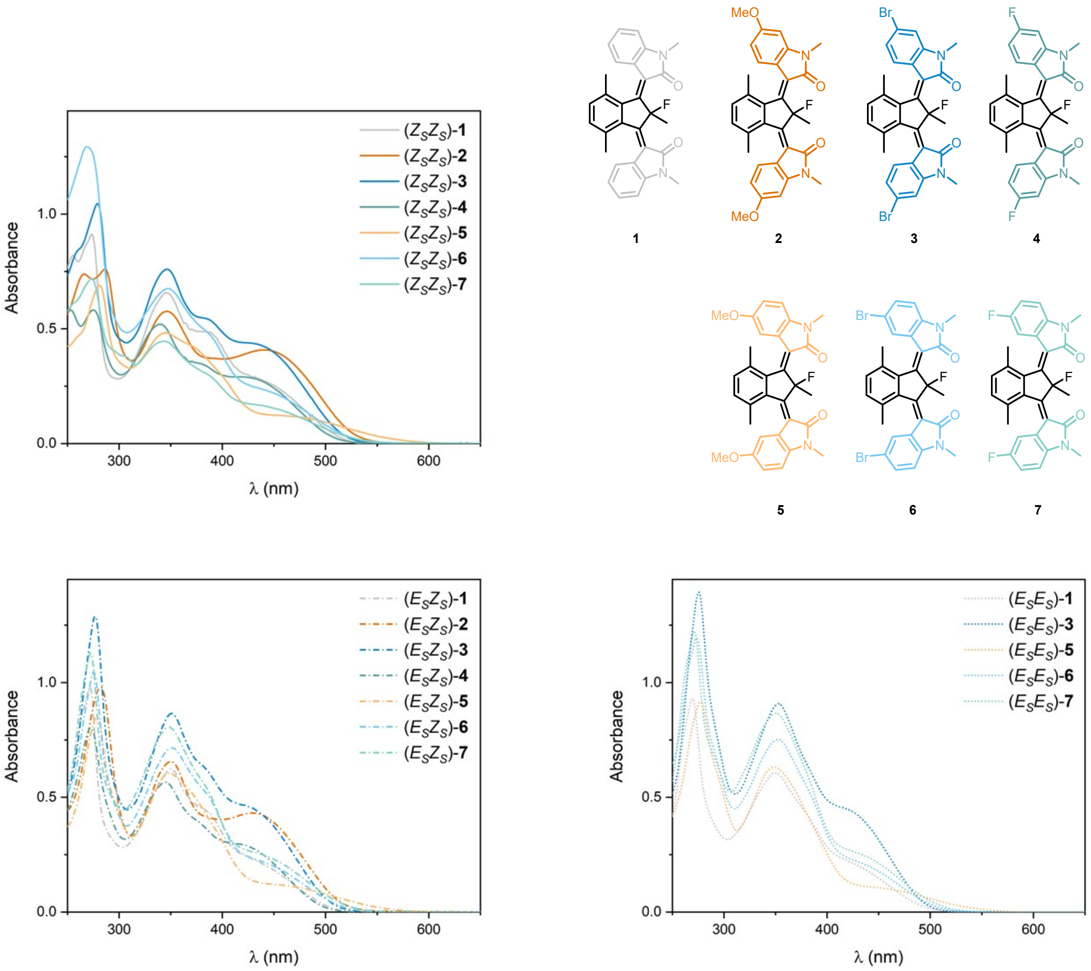

Figure S4. *UV/Vis absorption spectra of motors* ***1-7*** *in CH_2Cl_2 (~3 × 10^{-5} M, 20 °C). The spectra are sorted by isomers: Z_SZ_S, E_SZ_S and E_SE_S.*

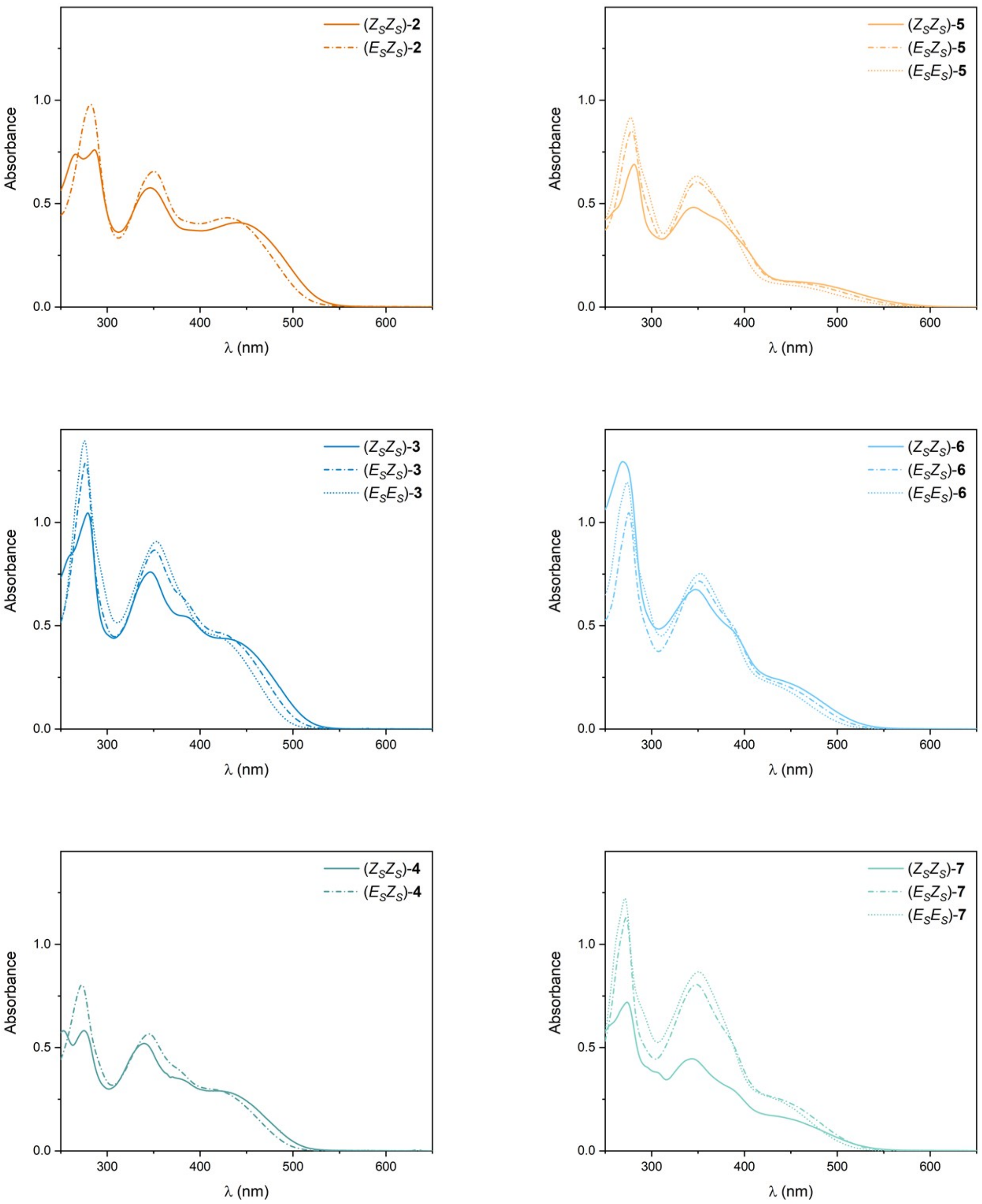

***Figure S5**. UV/Vis absorption spectra of motors **2-7** in CH_2Cl_2 (~3 × 10^{-5} M, 20 °C).*

Irradiation studies

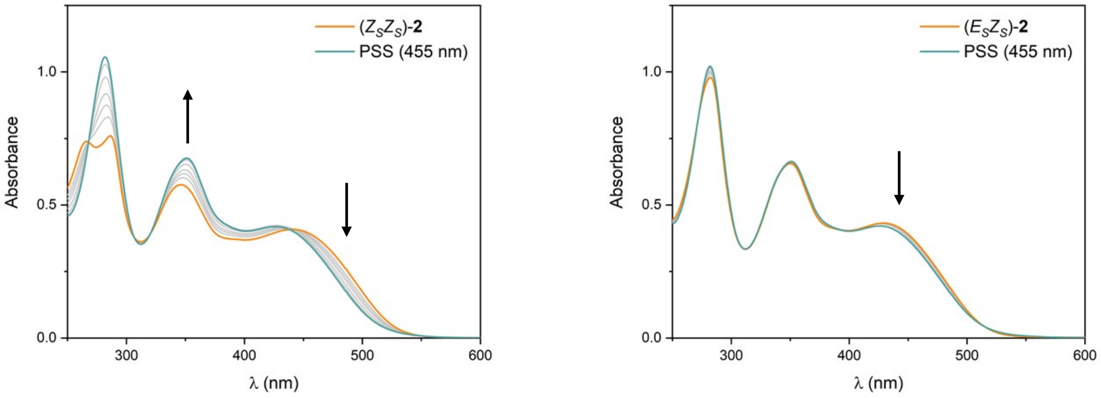

Figure S6. *UV/Vis absorption spectra of (ZSZS)-**2** (left) and (ESZS)-**2** (right) in CH_2Cl_2 (~3 × 10^{-5} M, 20 °C) upon irradiation with 455 nm light (6 min). The pure isomer is shown in orange and the obtained PSS in blue.*

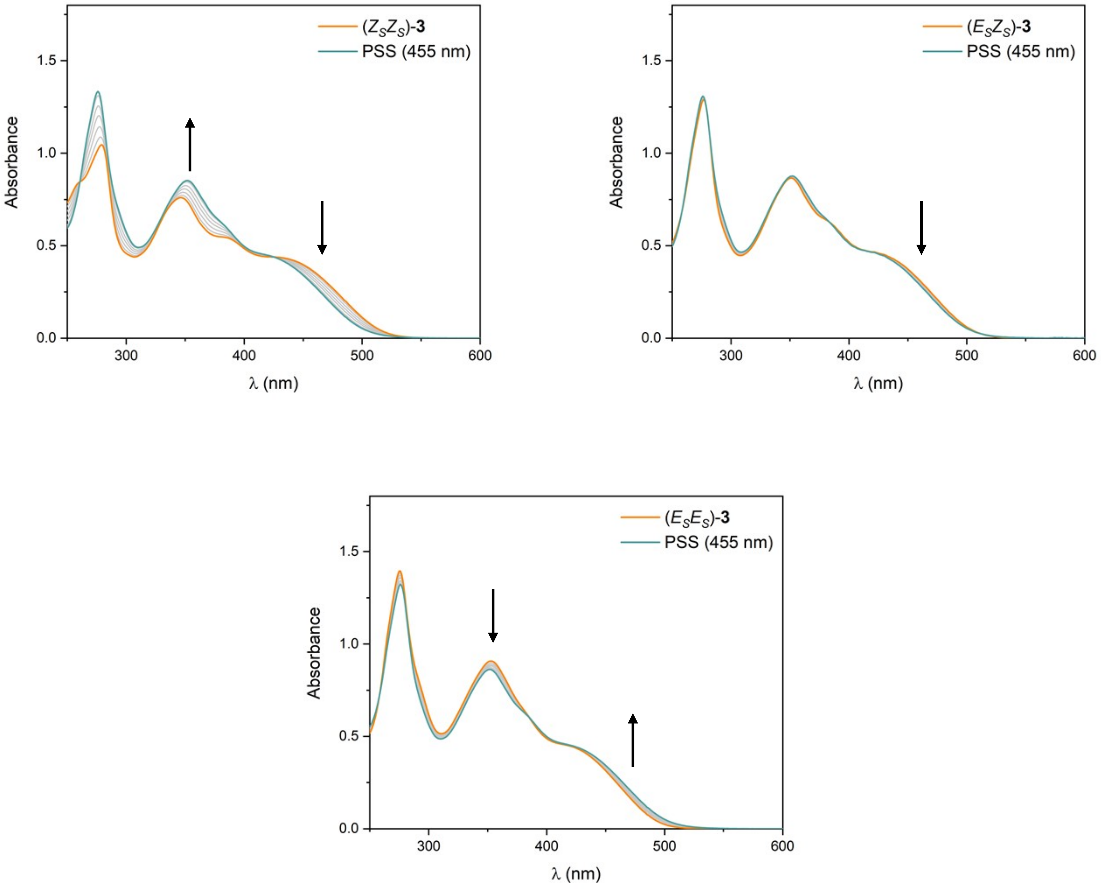

Figure S7. *UV/Vis absorption spectra of (ZSZS)-**3** (top left), (ESZS)-**3** (top right) and (ESES)-**3** (bottom) in CH_2Cl_2 (~3 × 10^{-5} M, 20 °C) upon irradiation with 455 nm light (5 min). The pure isomer is shown in orange and the obtained PSS in blue.*

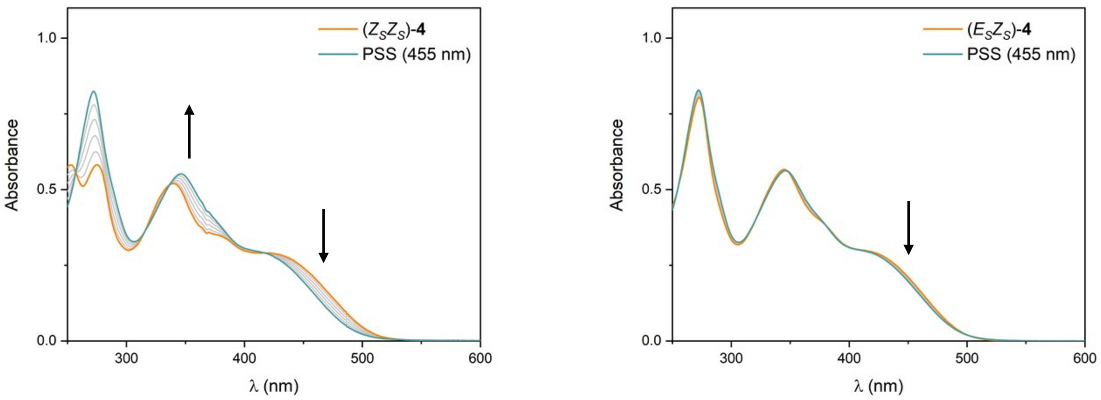

Figure S8. *UV/Vis absorption spectra of (Z_SZ_S)-**4** (left) and (E_SZ_S)-**4** (right) in CH_2Cl_2 (~3 × 10^{-5} M, 20 °C) upon irradiation with 455 nm light (8 min). The pure isomer is shown in orange and the obtained PSS in blue.*

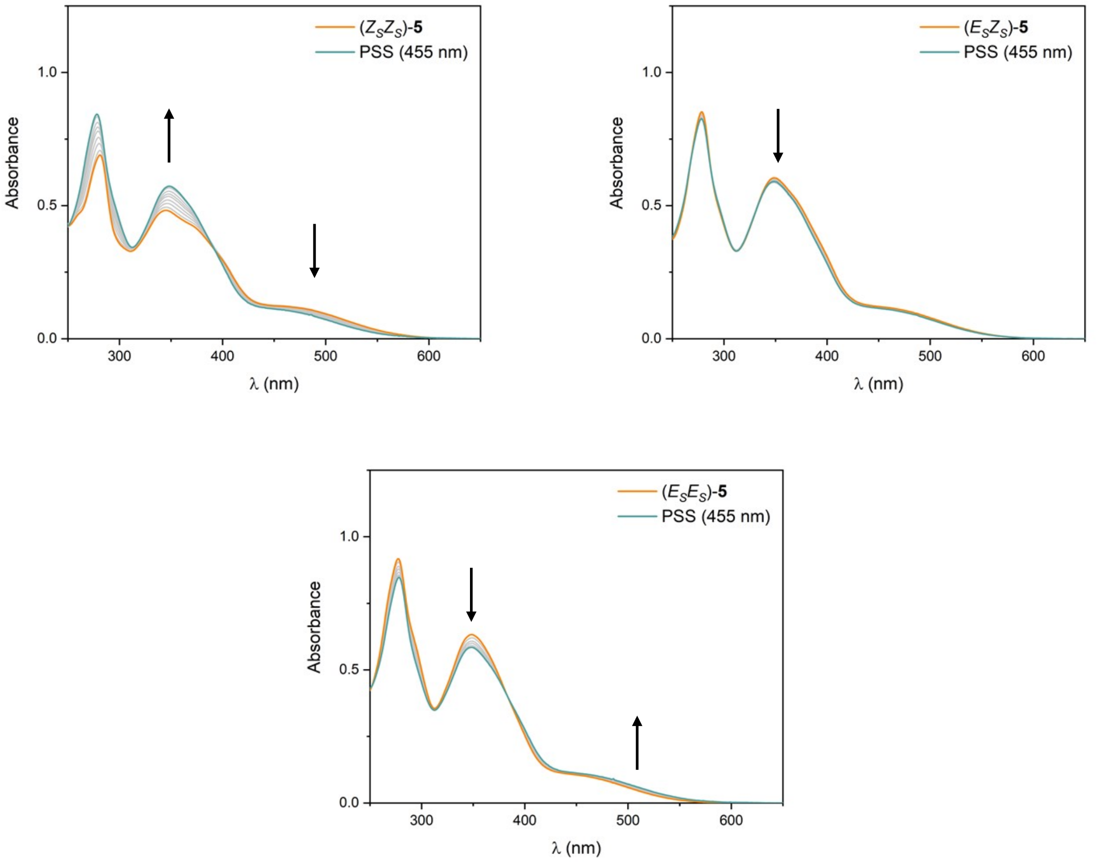

Figure S9. *UV/Vis absorption spectra of (Z_SZ_S)-**5** (top left), (E_SZ_S)-**5** (top right) and (E_SE_S)-**5** (bottom) in CH_2Cl_2 (~3 × 10^{-5} M, 20 °C) upon irradiation with 455 nm light (12 h). The pure isomer is shown in orange and the obtained PSS in blue.*

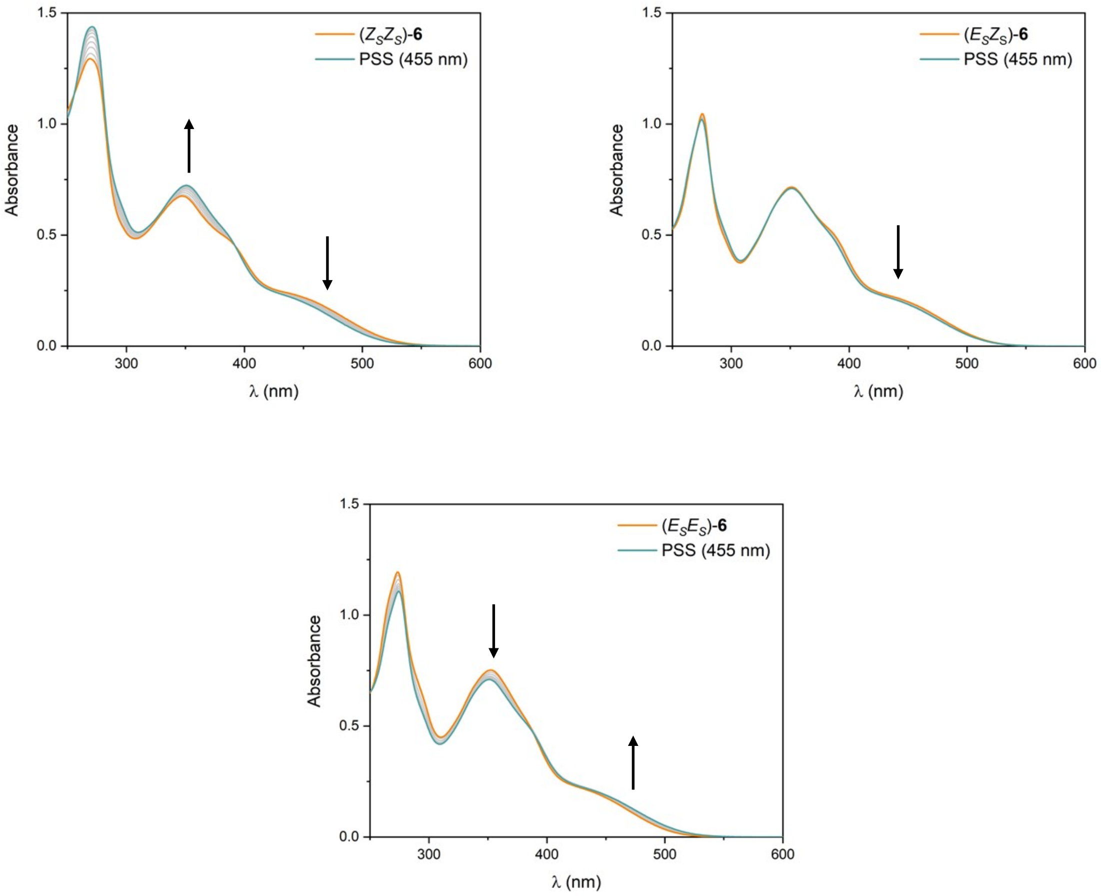

Figure S10. *UV/Vis absorption spectra of (Z_SZ_S)-**6** (top left), (E_SZ_S)-**6** (top right) and (E_SE_S)-**6** (bottom) in CH_2Cl_2 (~3 × 10^{-5} M, 20 °C) upon irradiation with 455 nm light (18 min). The pure isomer is shown in orange and the obtained PSS in blue.*

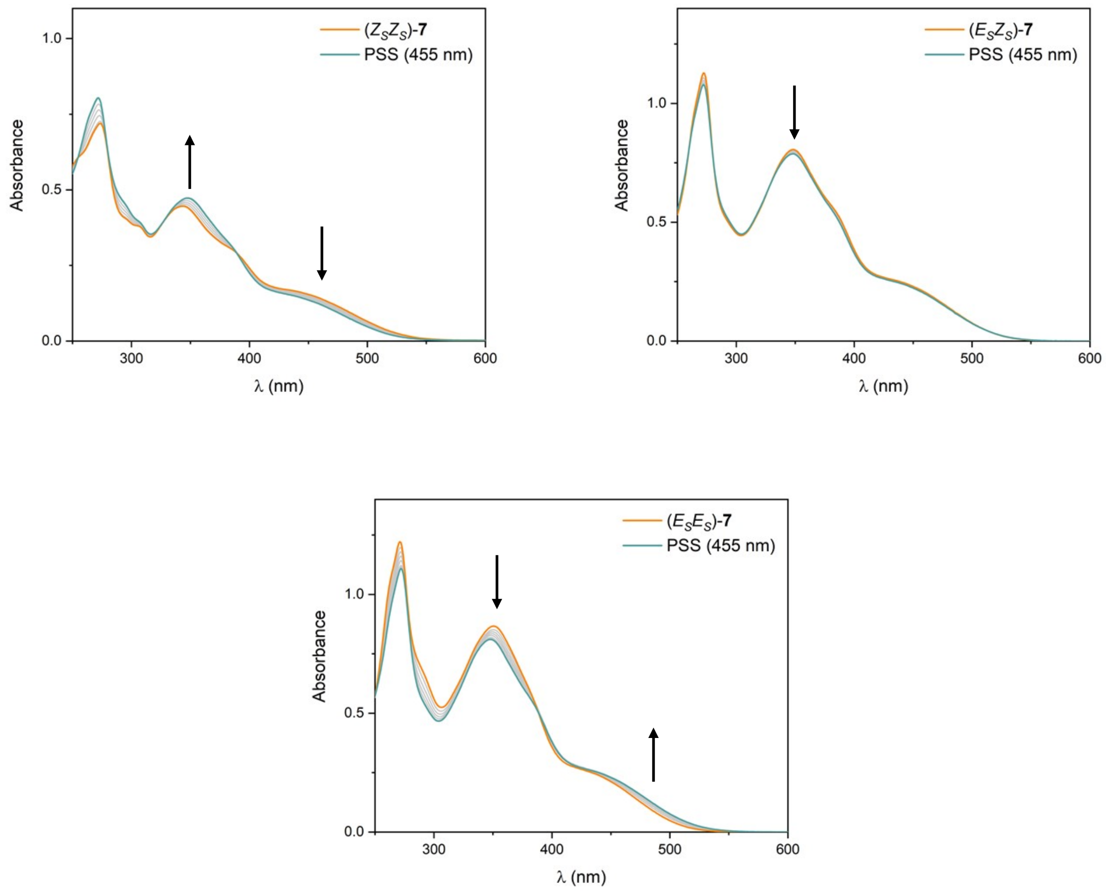

Figure S11. *UV/Vis absorption spectra of (Z_SZ_S)-**7** (top left), (E_SZ_S)-**7** (top right) and (E_SE_S)-**7** (bottom) in CH_2Cl_2 (~3 × 10^{-5} M, 20 °C) upon irradiation with 455 nm light (14 min). The pure isomer is shown in orange and the obtained PSS in blue.*

4. NMR Studies and Kinetic Experiments

NMR Irradiation Experiments

Room temperature experiments

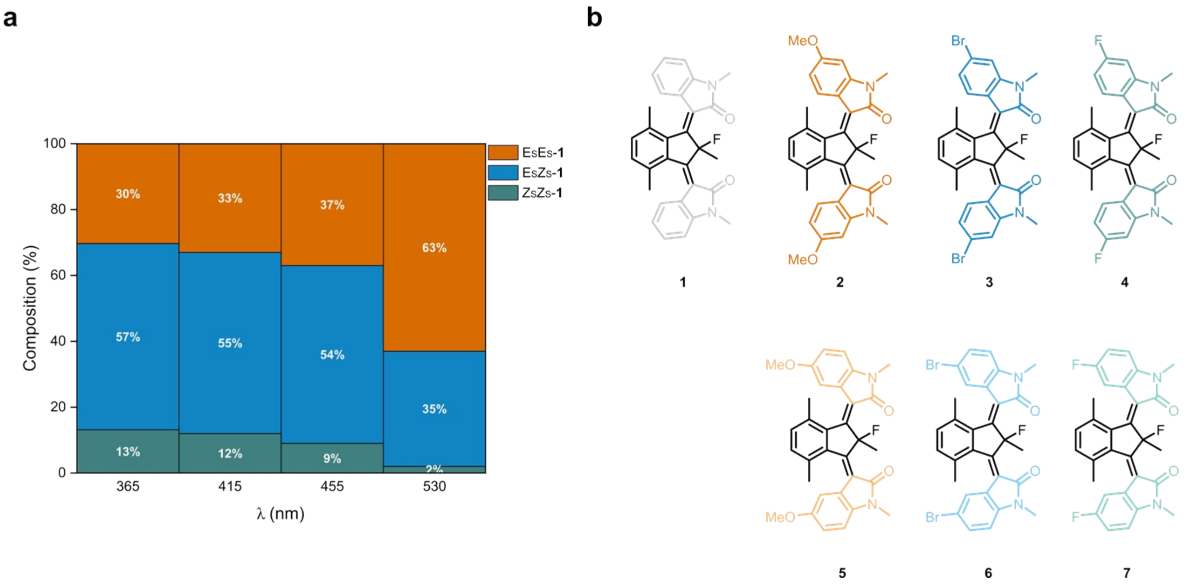

Figure S12. *a) Determination of the PSS ratios achieved upon irradiation with 455 nm, 415 nm and 365 nm of functionalised motors **1** in CD_2Cl_2 (20 °C) using ^{19}F NMR spectroscopy (565 MHz). The same PSS ratios were obtained when starting from the different stable isomers: (Z_SZ_S), (E_SZ_S) and (E_SE_S). b) Overview of the studied bridged-isoindigo molecular motors.*

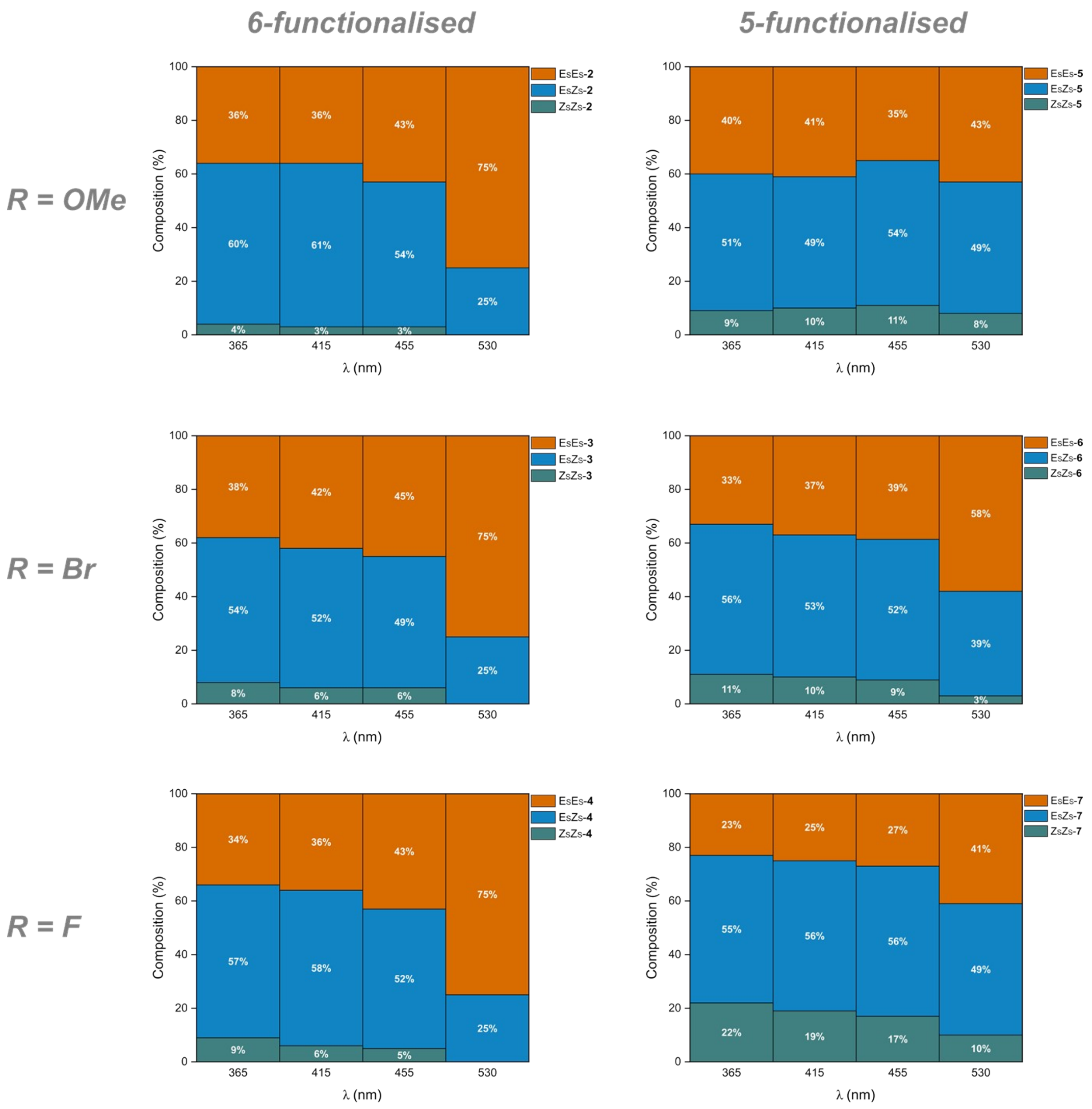

Figure S13. *Determination of the PSS ratios achieved upon irradiation with 455 nm, 415 nm and 365 nm of functionalised motors **2-7** in CD_2Cl_2 (20 °C) using ^{19}F NMR spectroscopy (565 MHz). The same PSS ratios were obtained when starting from the different stable isomers: (Z_SZ_S), (E_SZ_S) and (E_SE_S). All isolated stable isomers were measured for the respective motor.*

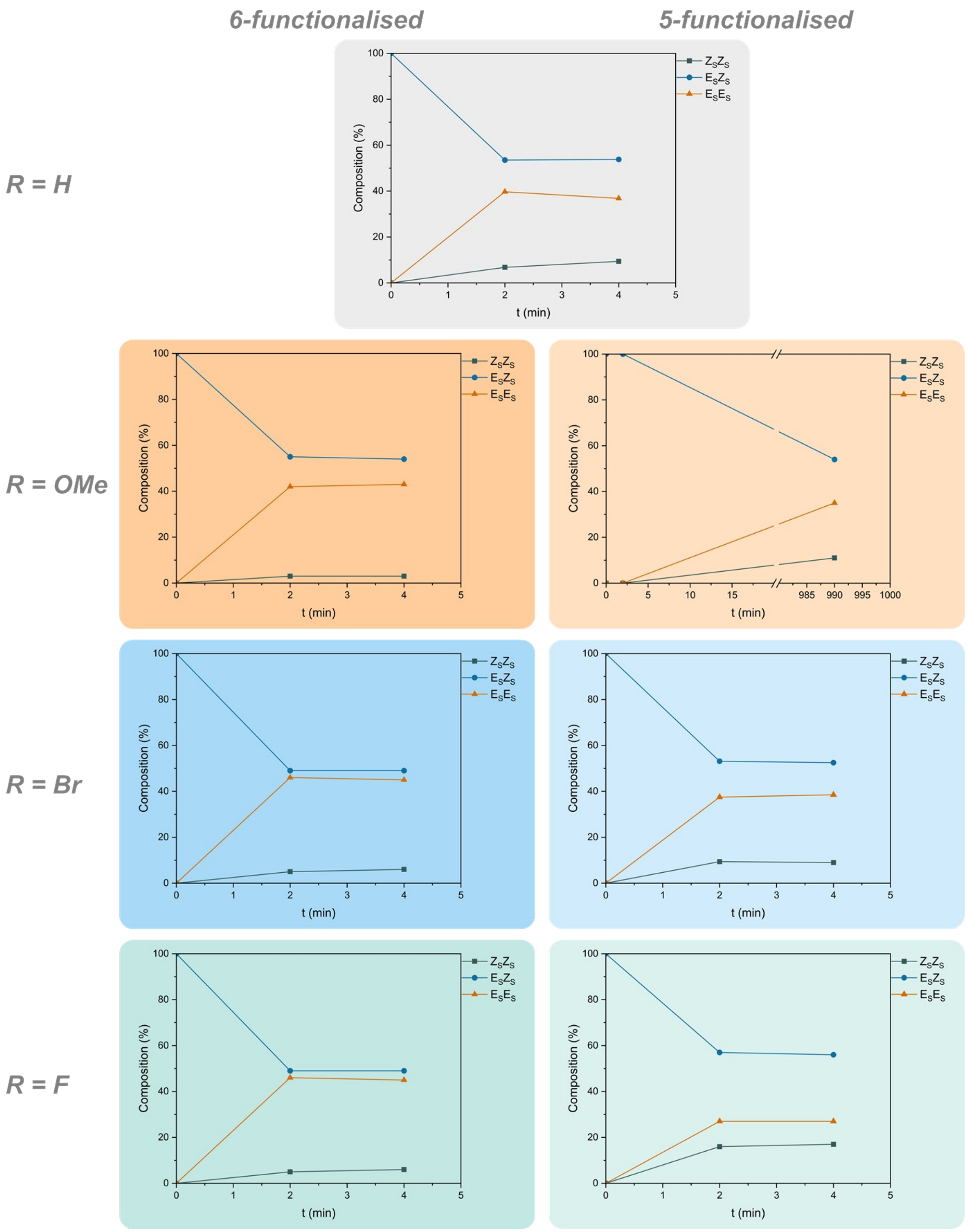

Figure S14. *Kinetics of reaching PSS (λ = 455 nm) of motors* ***1-7*** *in CD_2Cl_2 (20 °C) using ^{19}F NMR spectroscopy (565 MHz).*

The low photochemical efficiency of motor **5** likely stems from a low quantum yield. The estimated extinction coefficients for motors **1**–**7** at 455 nm (ϵ_{455}) and 365 nm (ϵ_{365}) are summarised in Table S1. The estimated differences in molar extinction coefficients at a particular wavelength across the series of motors with varying rotor substitution are on the order of 4–5-fold (i.e., less than one order of magnitude). Motor **5** indeed shows the lowest ϵ_{455} values among the series, while its ϵ_{365} values is comparable to the other motors. However, the large difference in observed photochemical efficiency – requiring irradiation times of several hours for motor **5** versus minutes for the other motors (Figure S14) – cannot be explained solely by these modest variations in extinction coefficients. We therefore conclude that a low quantum yield is the most likely primary contributor to the reduced photochemical efficiency of motor **5**, although differences in extinction coefficient may play a minor role, particularly under irradiation with, for example, 455 nm light.

Table S1. *Roughly estimated extinction coefficients at 455 nm and 365 nm of motors* ***1-7*** *(based on UV/Vis data in* CH_2Cl_2*, ~3 × 10^{-5} M, 20 °C).*

	ϵ_{455} (10^3 M^{-1} cm^{-1})			ϵ_{365} (10^3 M^{-1} cm^{-1})		
motor	Z_SZ_S	E_SZ_S	E_SE_S	Z_SZ_S	E_SZ_S	E_SE_S
**1**	8	6	4	17	17	17
**2**	13	12		16	18	
**3**	13	11	9	20	25	27
**4**	8	6		12	45	
**5**	4	4	3	15	18	19
**6**		6	5		21	23
**7**		7	7		23	26

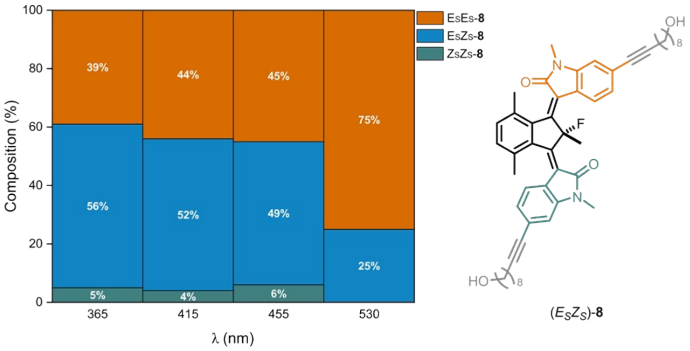

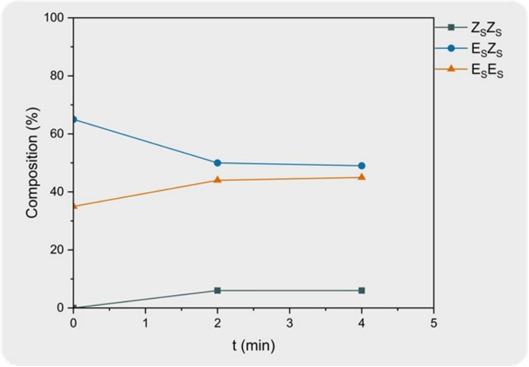

***Figure S15**. Determination of the PSS ratios achieved upon irradiation with 455 nm, 415 nm and 365 nm of motor **8** in CD_2Cl_2 (20 °C) using ^{19}F NMR spectroscopy (565 MHz) (top). Kinetics of reaching PSS (λ = 455 nm) of motor **8** (starting from a mixture of (E_SZ_S)-**8** and (E_SE_S)-**8** (65:35)) in CD_2Cl_2 (20 °C) using ^{19}F NMR spectroscopy (565 MHz) (bottom).*

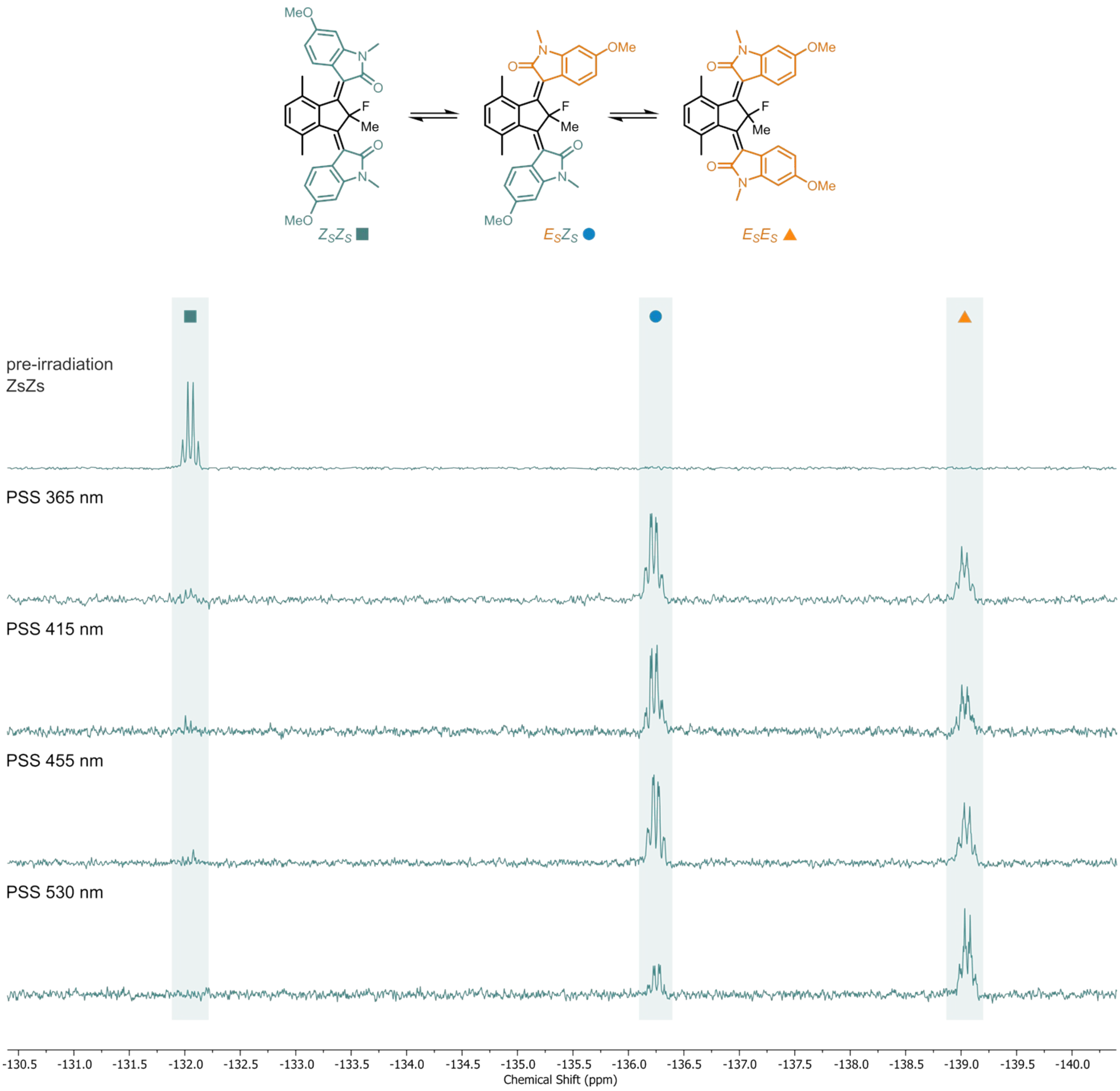

***Figure S16**. Determination of the PSS ratios achieved upon irradiation with 530 nm, 455 nm, 415 nm and 365 nm starting from of (Z_SZ_S)-**2** in CD_2Cl_2 (20 °C) using ^{19}F NMR spectroscopy (565 MHz). The same PSS ratios were obtained when starting from (E_SZ_S)-**2** instead of (Z_SZ_S)-**2**.*

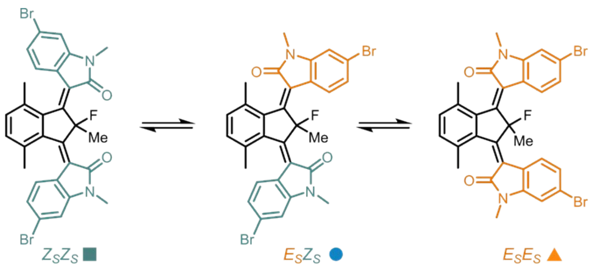

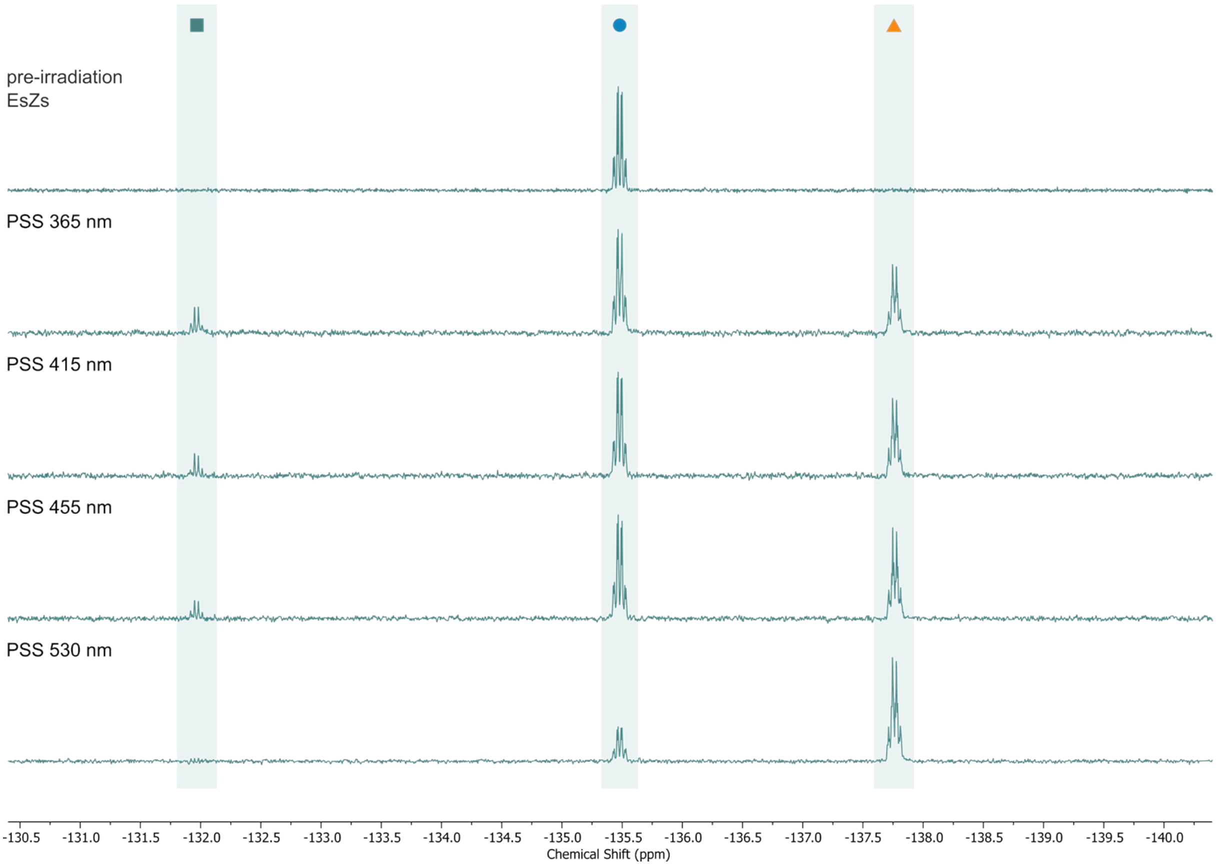

Figure S17. *Determination of the PSS ratios achieved upon irradiation with 530 nm, 455 nm, 415 nm and 365 nm starting from of* (E_SZ_S)*-**3** in* CD_2Cl_2 *(20 °C) using* ^{19}F *NMR spectroscopy (565 MHz). The same PSS ratios were obtained when starting from* (Z_SZ_S)*-**3** or* (E_SE_S)*-**3** instead of* (E_SZ_S)*-**3**.*

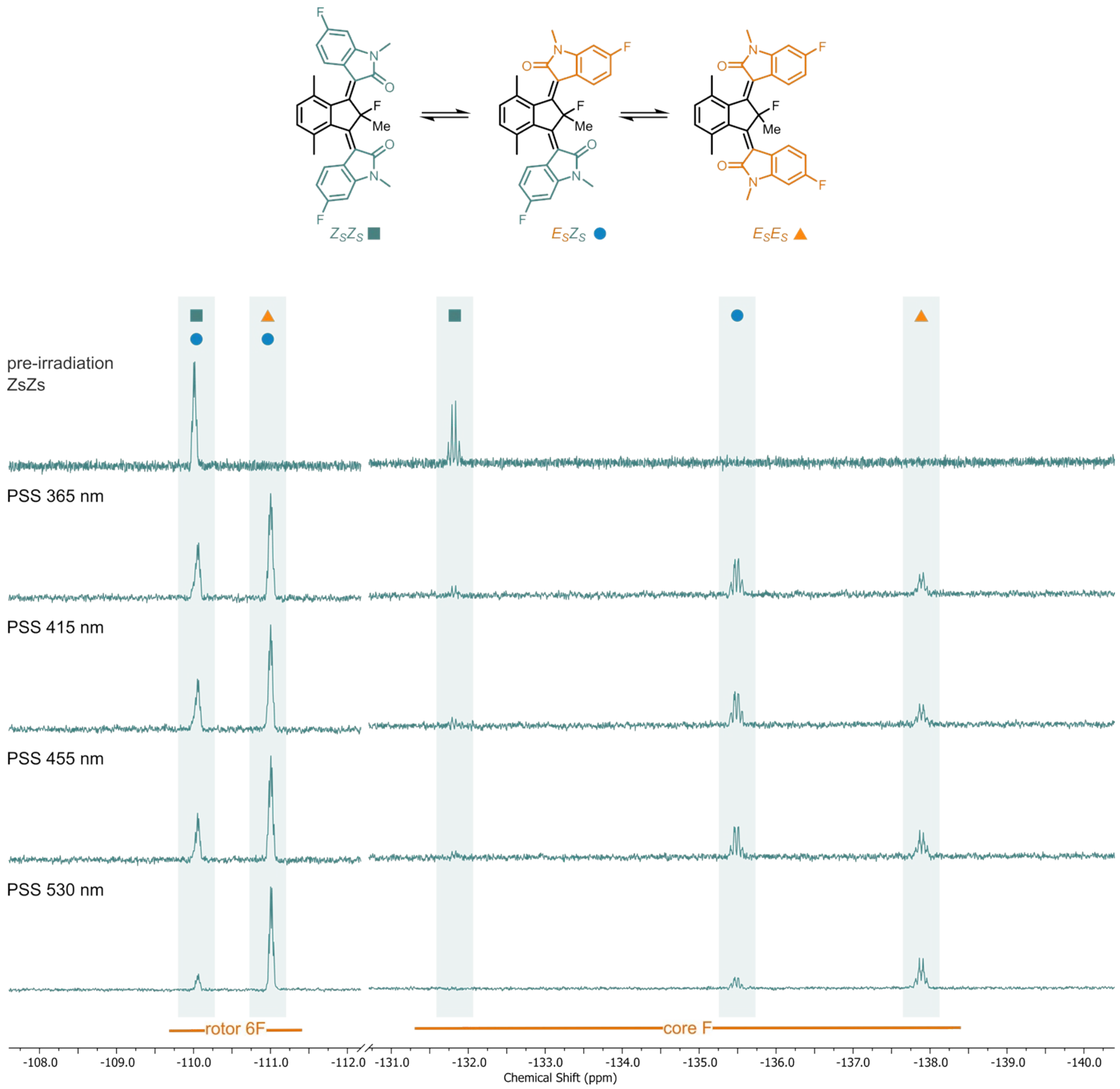

Figure S18. *Determination of the PSS ratios achieved upon irradiation with 530 nm, 455 nm, 415 nm and 365 nm starting from of (Z_SZ_S)-**4** in CD_2Cl_2 (20 °C) using ^{19}F NMR spectroscopy (565 MHz). The same PSS ratios were obtained when starting from (E_SZ_S)-**4** instead of (Z_SZ_S)-**4**.*

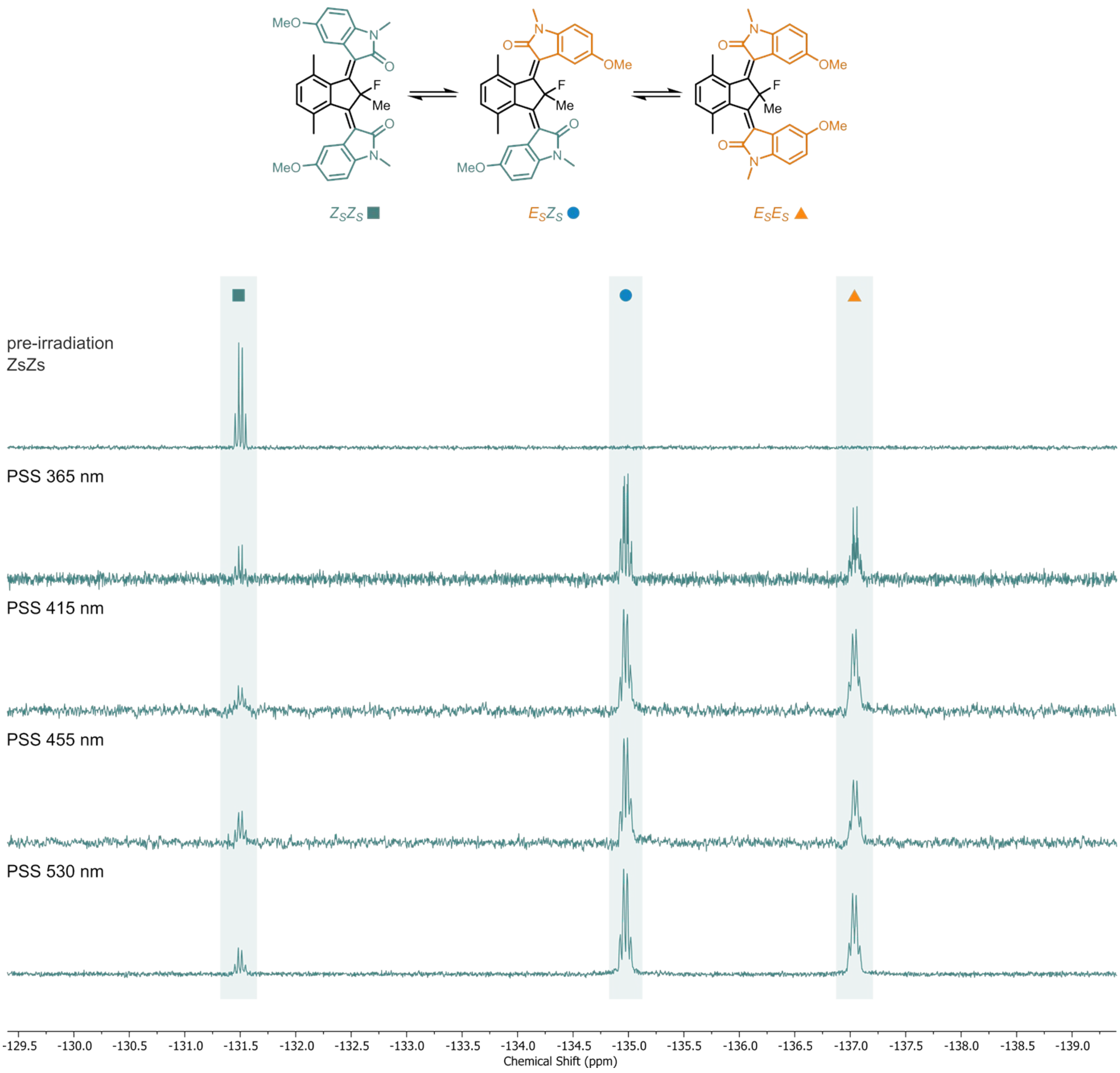

***Figure S19**. Determination of the PSS ratios achieved upon irradiation with 530 nm, 455 nm, 415 nm and 365 nm starting from of (Z_SZ_S)-**5** in CD_2Cl_2 (20 °C) using ^{19}F NMR spectroscopy (565 MHz). The same PSS ratios were obtained when starting from (E_SZ_S)-**5** or (E_SE_S)-**5** instead of (Z_SZ_S)-**5**.*

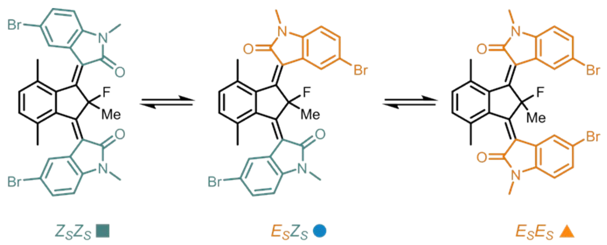

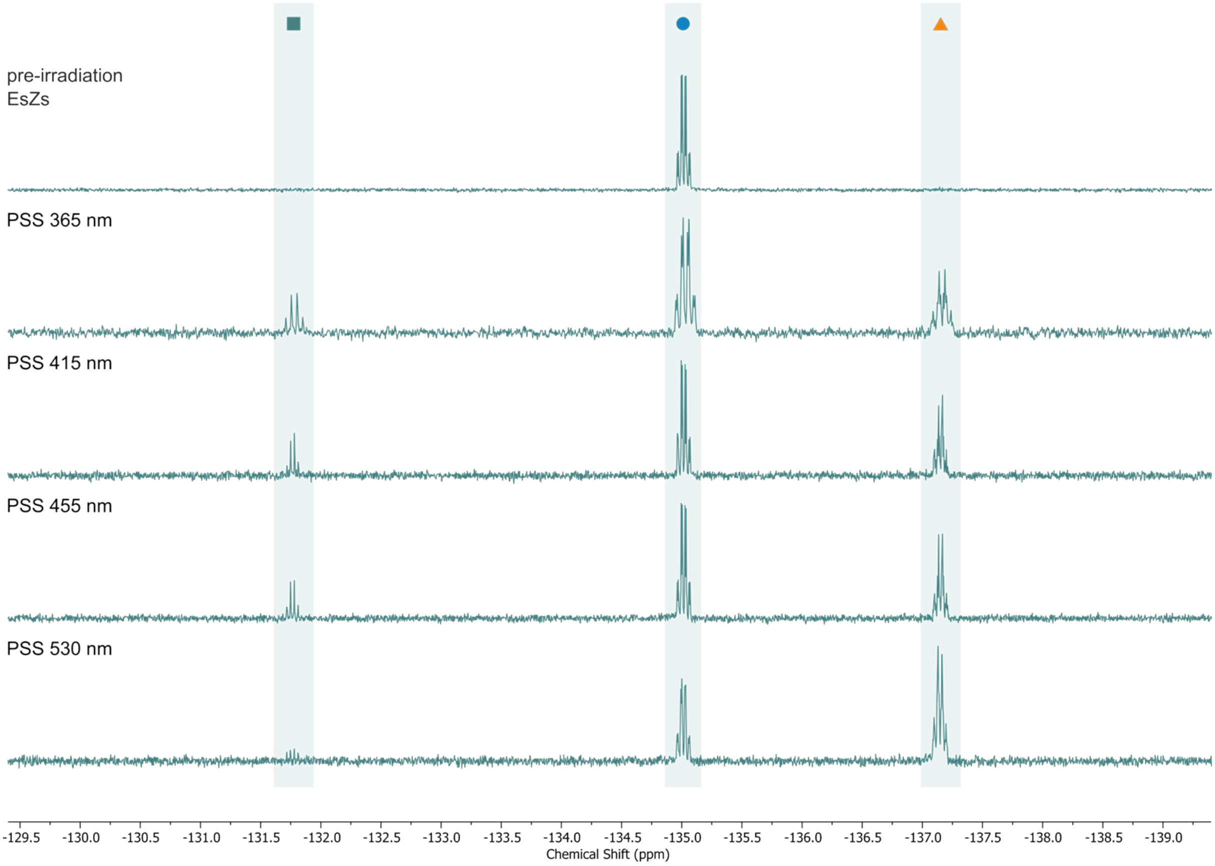

Figure S20. *Determination of the PSS ratios achieved upon irradiation with 530 nm, 455 nm, 415 nm and 365 nm starting from of (E_SZ_S)-**6** in CD_2Cl_2 (20 °C) using ^{19}F NMR spectroscopy (565 MHz). The same PSS ratios were obtained when starting from (Z_SZ_S)-**6** or (E_SE_S)-**6** instead of (E_SZ_S)-**6**.*

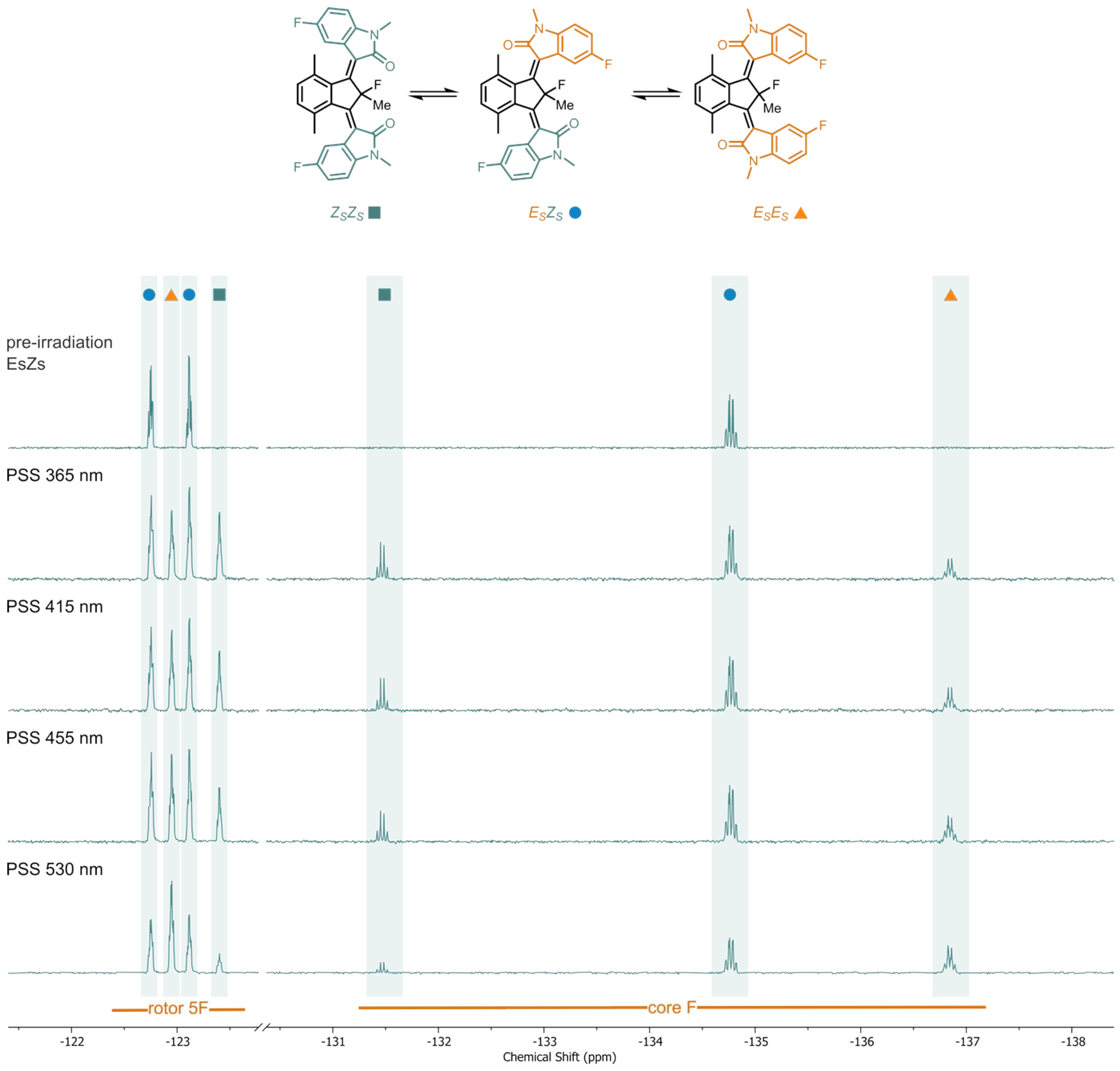

Figure S21. *Determination of the PSS ratios achieved upon irradiation with 530 nm, 455 nm, 415 nm and 365 nm starting from of (E_SZ_S)-**7** in CD_2Cl_2 (20 °C) using ^{19}F NMR spectroscopy (565 MHz). The same PSS ratios were obtained when starting from (Z_SZ_S)-**7** or (E_SE_S)-**7** instead of (E_SZ_S)-**7**.*

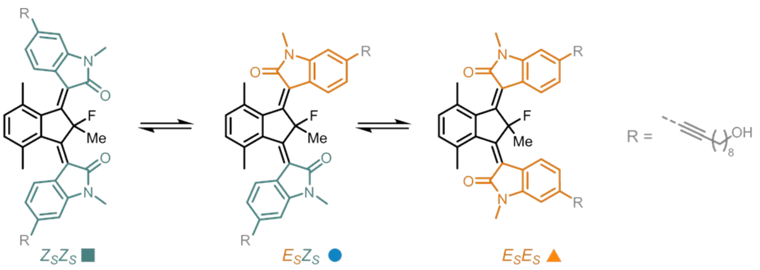

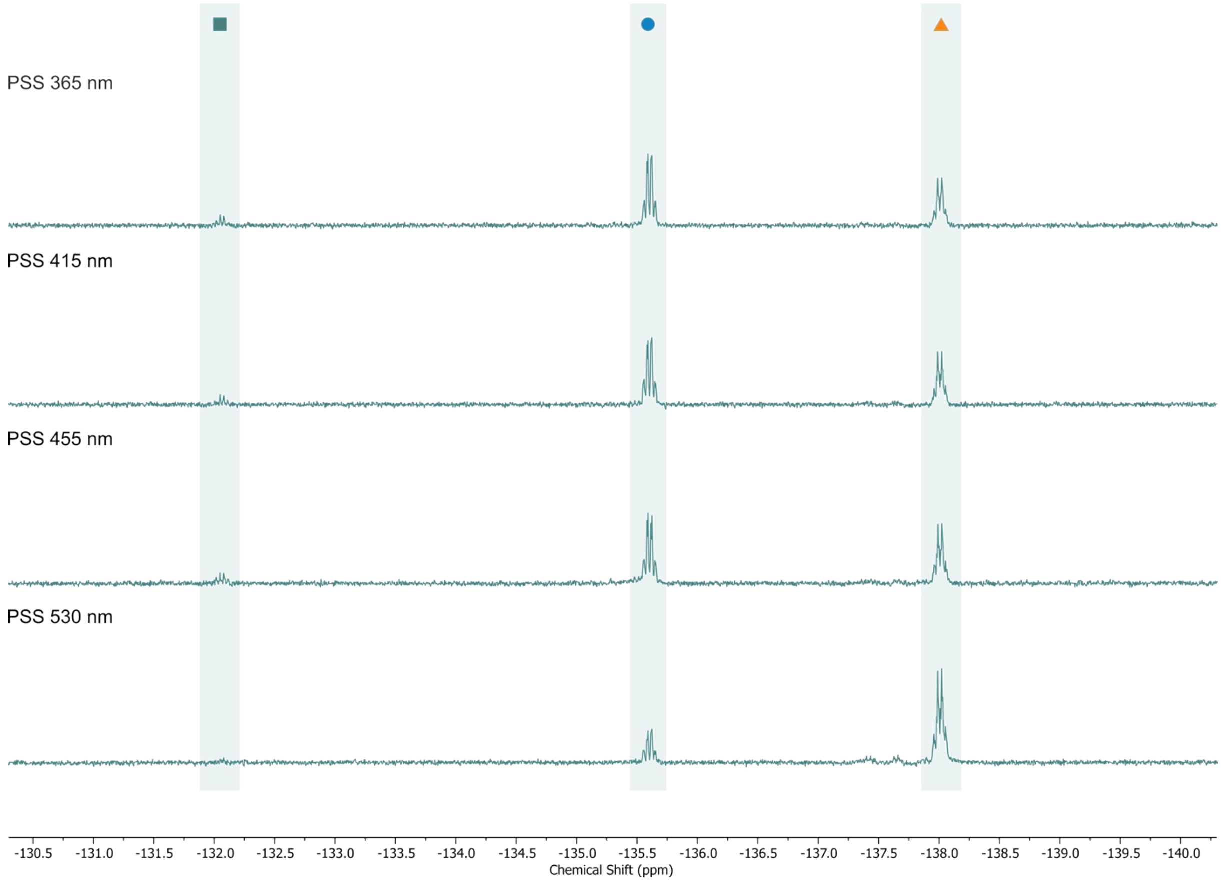

***Figure S22**. Determination of the PSS ratios achieved upon irradiation with 530 nm, 455 nm, 415 nm and 365 nm starting from a mixture of (E_SZ_S)-**8** and (E_SE_S)-**8** (65:35) in CD_2Cl_2 (20 °C) using ^{19}F NMR spectroscopy (565 MHz).*

Unidirectional Rotation Mechanism

The rotational cycle shown in Figure S23 presents all intermediates involved in the operation of functionalised bridged-isoindigo molecular motors, as experimentally confirmed in low-temperature studies

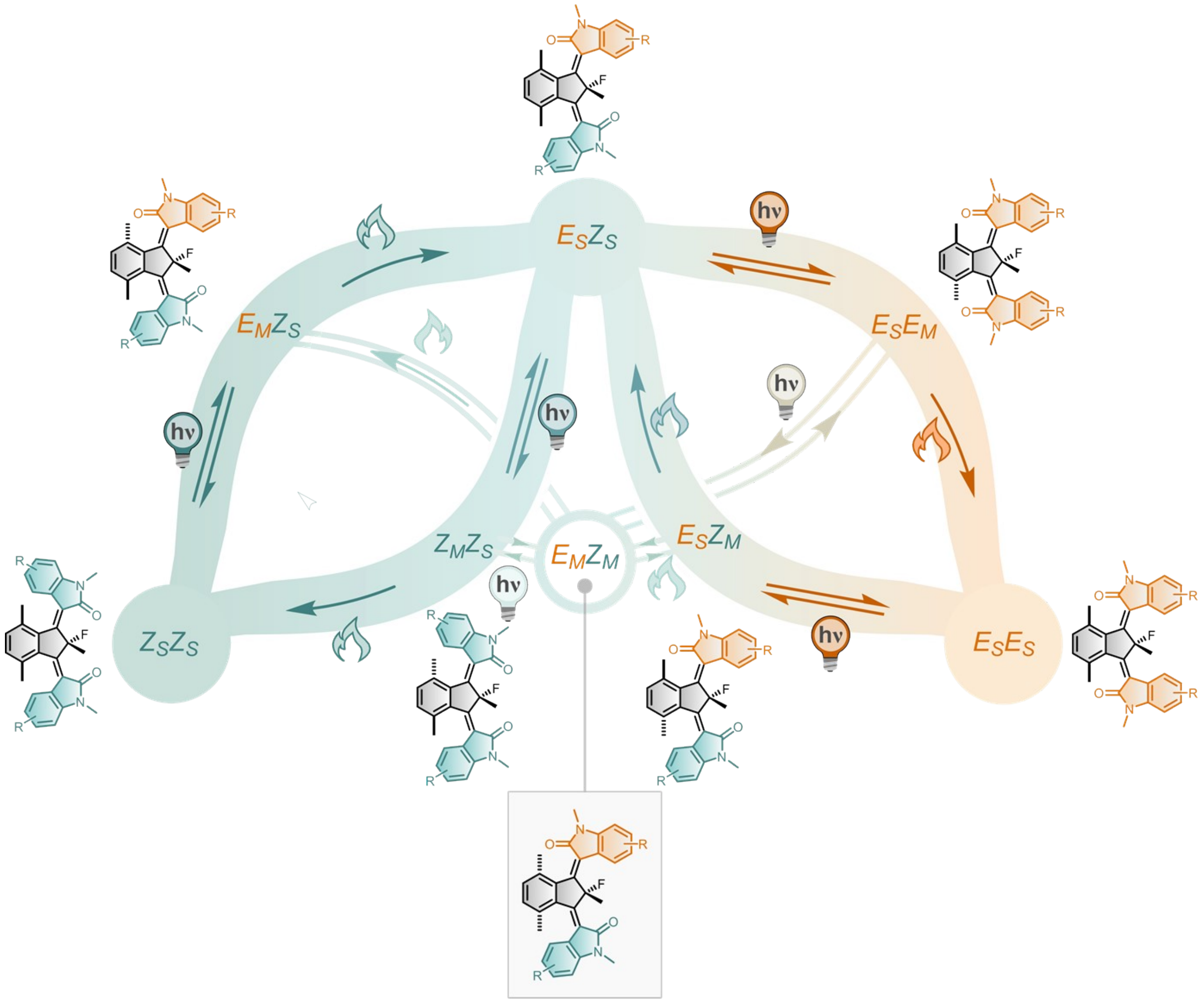

***Figure S23**. Unidirectional rotational cycle of functionalised bridged-isoindigo molecular motors showing the structures of the involved intermediates. The cycle connects stable, single metastable, and double metastable isomers through successive photochemical E/Z (PEZ) and thermal helix inversion (THI) steps. During PEZ isomerisation, one alkene undergoes an E/Z isomerisation accompanied by interconversion between stable and metastable geometries of the attached rotor. THI corresponds to the thermal relaxation of a rotor from a metastable to a stable configuration. The two four-step leafs depict a complete 360° unidirectional rotation of one of the rotors, while the off-leaf pathways illustrate the connectivity of the double metastable state (E_MZ_M) to all single metastable states via additional PEZ isomerisation and THI steps. For clarity, a simplified mechanism displaying a single rotational cycle via E_SZ_S (rather than also the enantiomeric pathway through Z_SE_S) is displayed.*

Low-temperature experiments

*Motor **2***

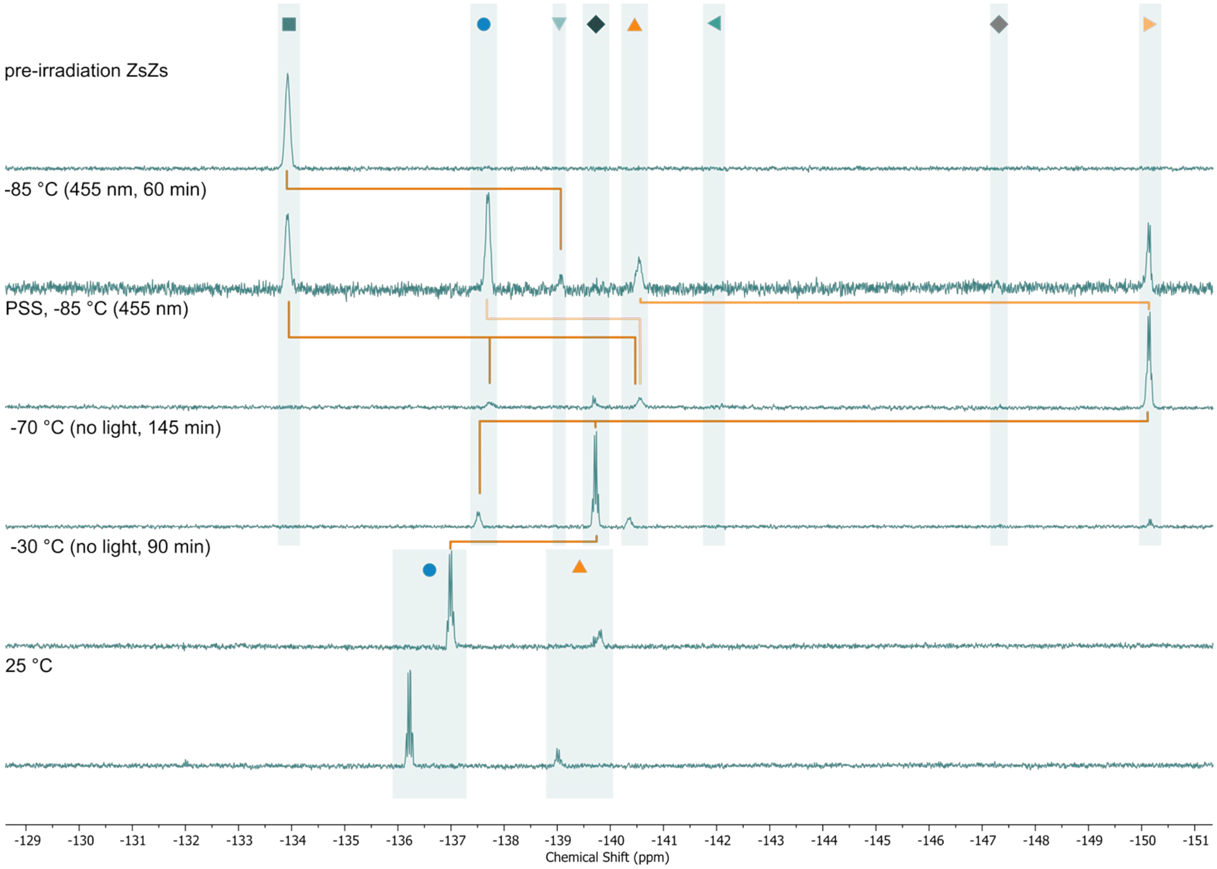

Figure S24. *The* [19]*F NMR spectra (470 MHz) of* (Z_SZ_S)-**2** *(4.2 mM in* CD_2Cl_2*) upon irradiation at -85 °C, partial and full relaxation in the dark, zoomed in on the core fluorine region. The spectra were recorded at the labelled temperatures.*

Low-temperature irradiation of Z_SZ_S-**2**

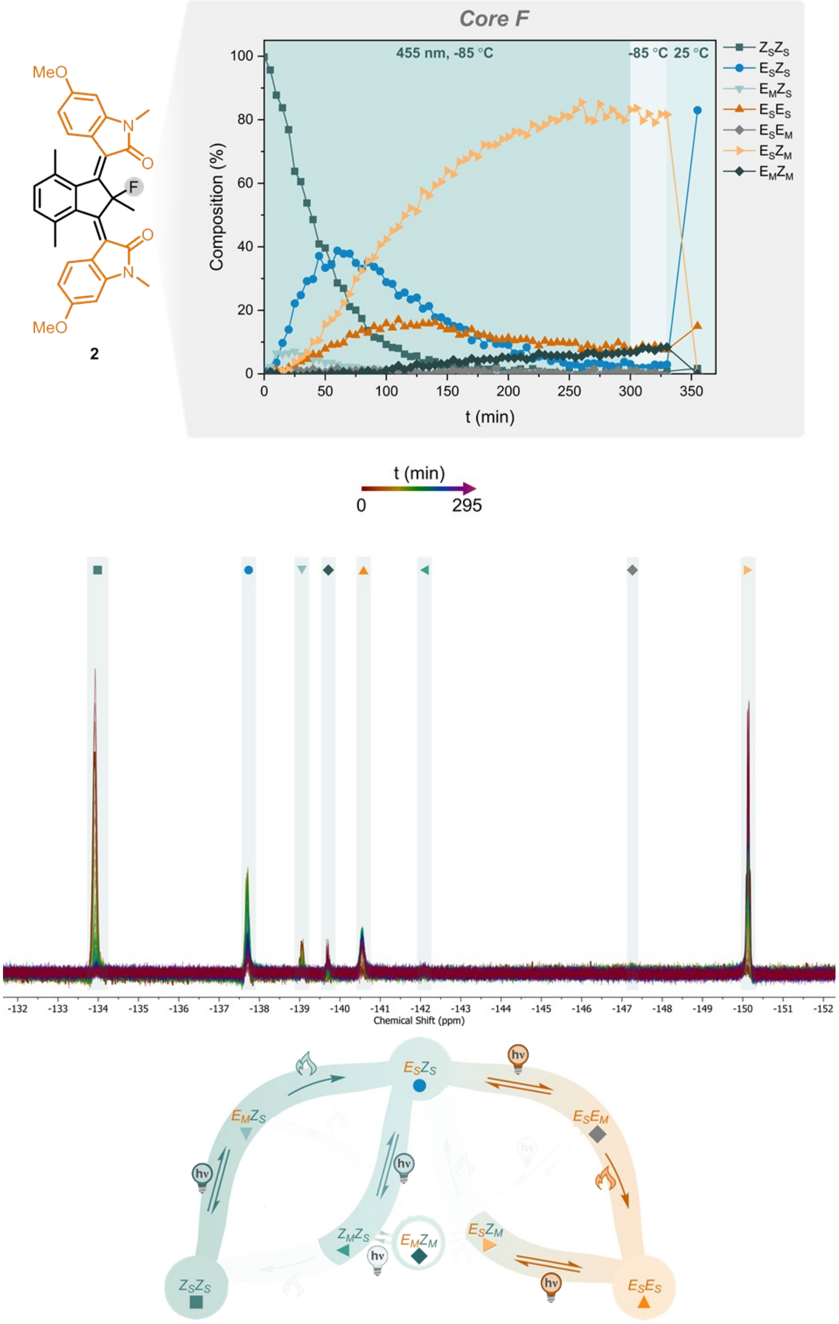

Figure S25. *Top: Sample of* (Z_SZ_S)-**2** *irradiated to PSS with 455 nm at -85 °C. The sample was subsequently kept at -85 °C for 35 min and warmed up to room temperature. The spectral changes of the core fluorine atom was followed. The measurements were performed at the labelled temperatures. Middle: The corresponding* ^{19}F *NMR superimposed spectra (470 MHz) of* (Z_SZ_S)-**2** *(4.2 mM in* CD_2Cl_2*) upon irradiation at -85 °C with isomer assignment. Bottom: Motor operation overview under these conditions (t = 0 – 295 min).*

Thermal relaxation of PSS (455 nm, -85 °C) of **2** at -70 °C

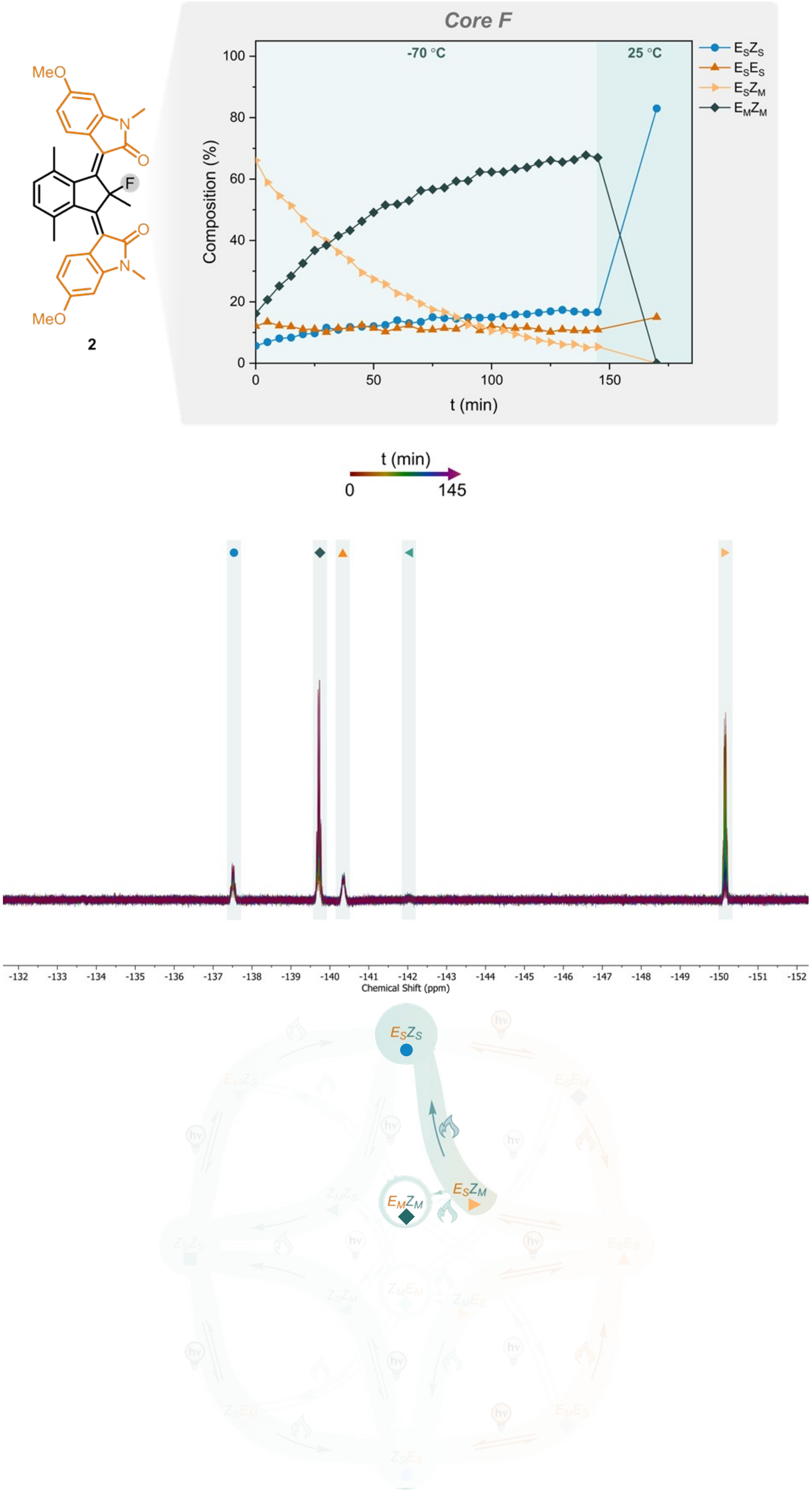

***Figure S26**. Top: Thermal decay in the dark of pre-irradiated (Z_SZ_S)-**2** (PSS with 455 nm at -85 °C) at -70 °C. The sample was subsequently warmed up to room temperature. The measurements were performed at -70 °C. The spectral changes of the core fluorine atom was followed. Bottom: Motor operation overview under these conditions (t = 0 – 145 min).*

Full thermal relaxation of partially relaxed PSS (455 nm, -85 °C, then dark, -70 °C) of **2** at -30 °C

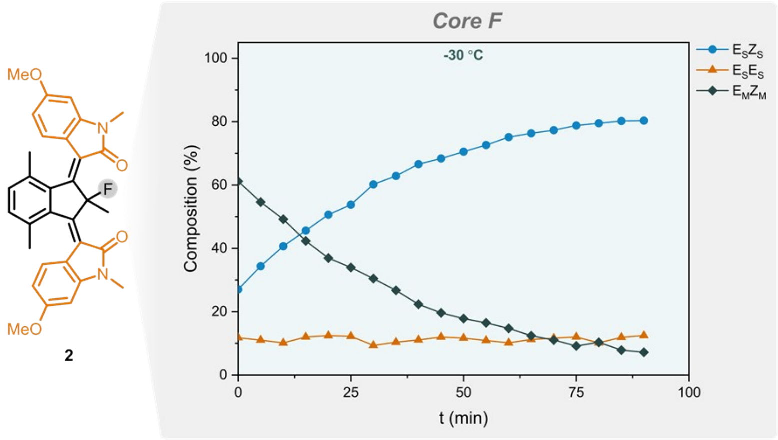

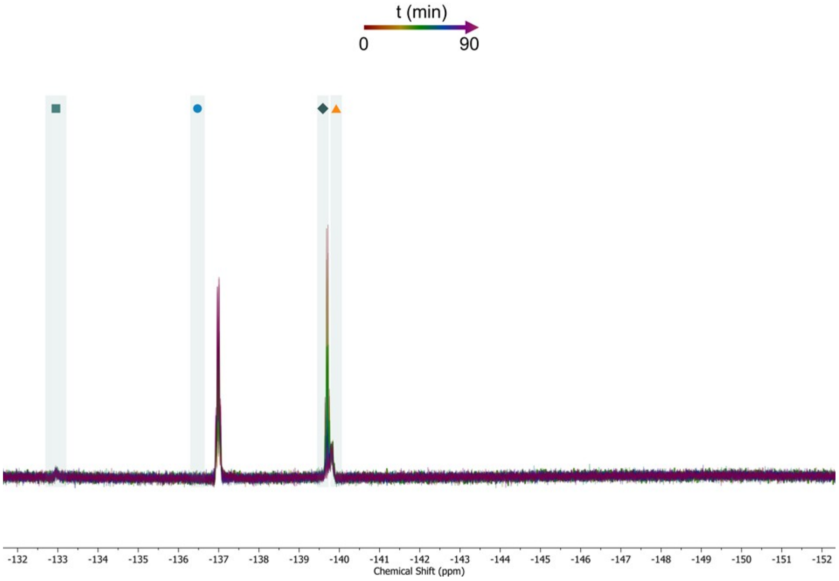

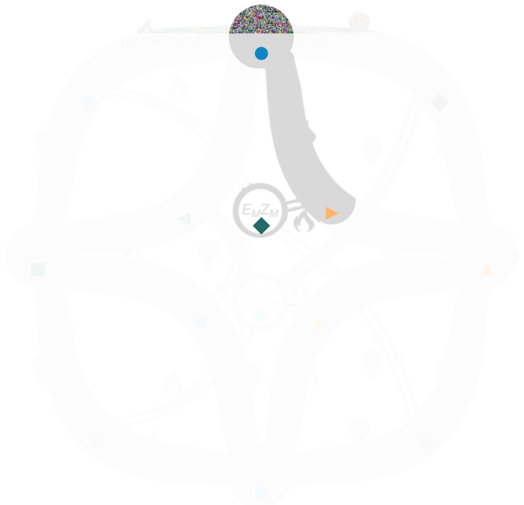

Figure S27. *Top: Thermal decay in the dark of pre-irradiated (Z_SZ_S)-**2** (PSS: 455 nm at -85 °C, then dark, -70 °C) at -30 °C. The sample was subsequently warmed up to room temperature. The measurements were performed at -30 °C. The spectral changes of the core fluorine atom was followed. Bottom: Motor operation overview under these conditions.*

*Motor **4***

Low-temperature irradiation of Z_SZ_S-**4**

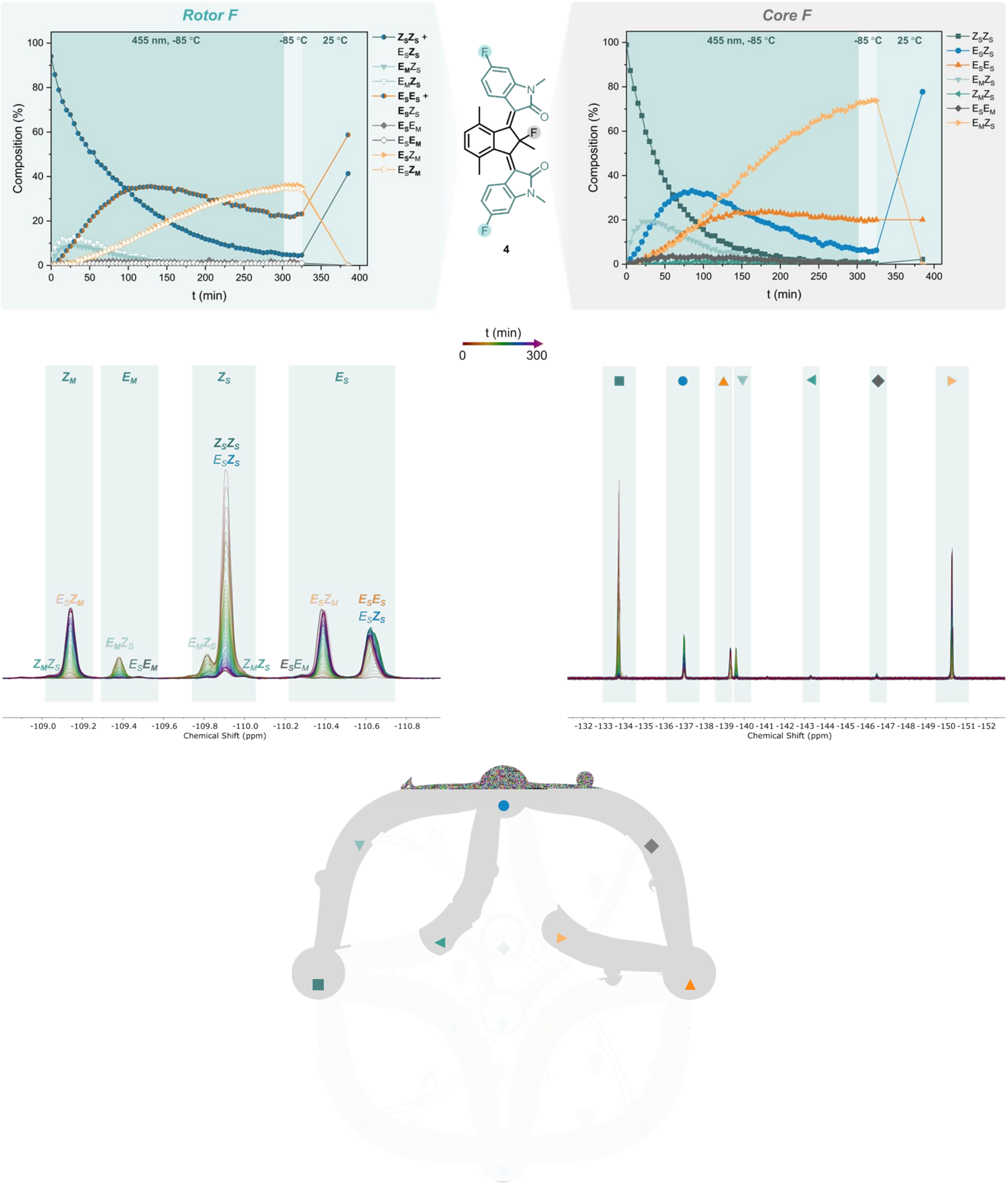

Figure S28. *Top: Sample of (Z_SZ_S)-**4** irradiated to PSS with 455 nm at -85 °C. The sample was subsequently kept at -85 °C for 30 min and warmed up to room temperature. The spectral changes of the core (right) and rotor (left) fluorine atoms were followed. The measurements were performed at the labelled temperatures. Middle: The corresponding ^{19}F NMR superimposed spectra (470 MHz) of (Z_SZ_S)-**4** (6.6 mM in CD_2Cl_2) upon irradiation at -85 °C with isomer assignment. For the rotor fluorine, the rotor shown in bold is the contributing rotor to the signal. Bottom: Motor operation overview under these conditions (t =0 – 300 min).*

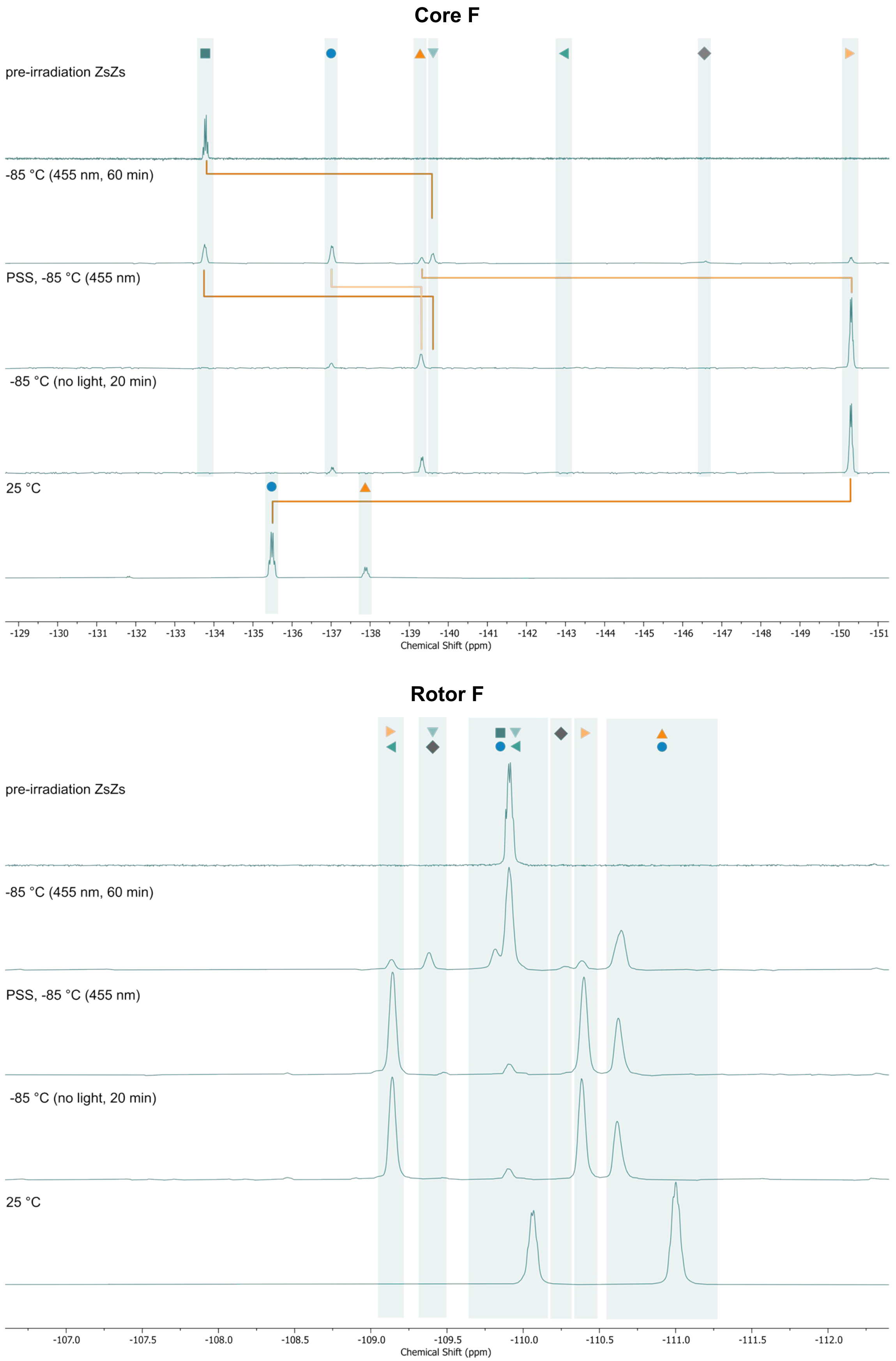

Figure S29. *The ^{19}F NMR spectra (470 MHz) of (Z_SZ_S)-**4** (6.6 mM in CD_2Cl_2) upon irradiation at -85 °C followed by full relaxation in the dark, zoomed in on the core (top) and rotor (bottom) fluorine regions. The spectra were recorded at the labelled temperatures.*

Low-temperature irradiation of E_SZ_S-**4**

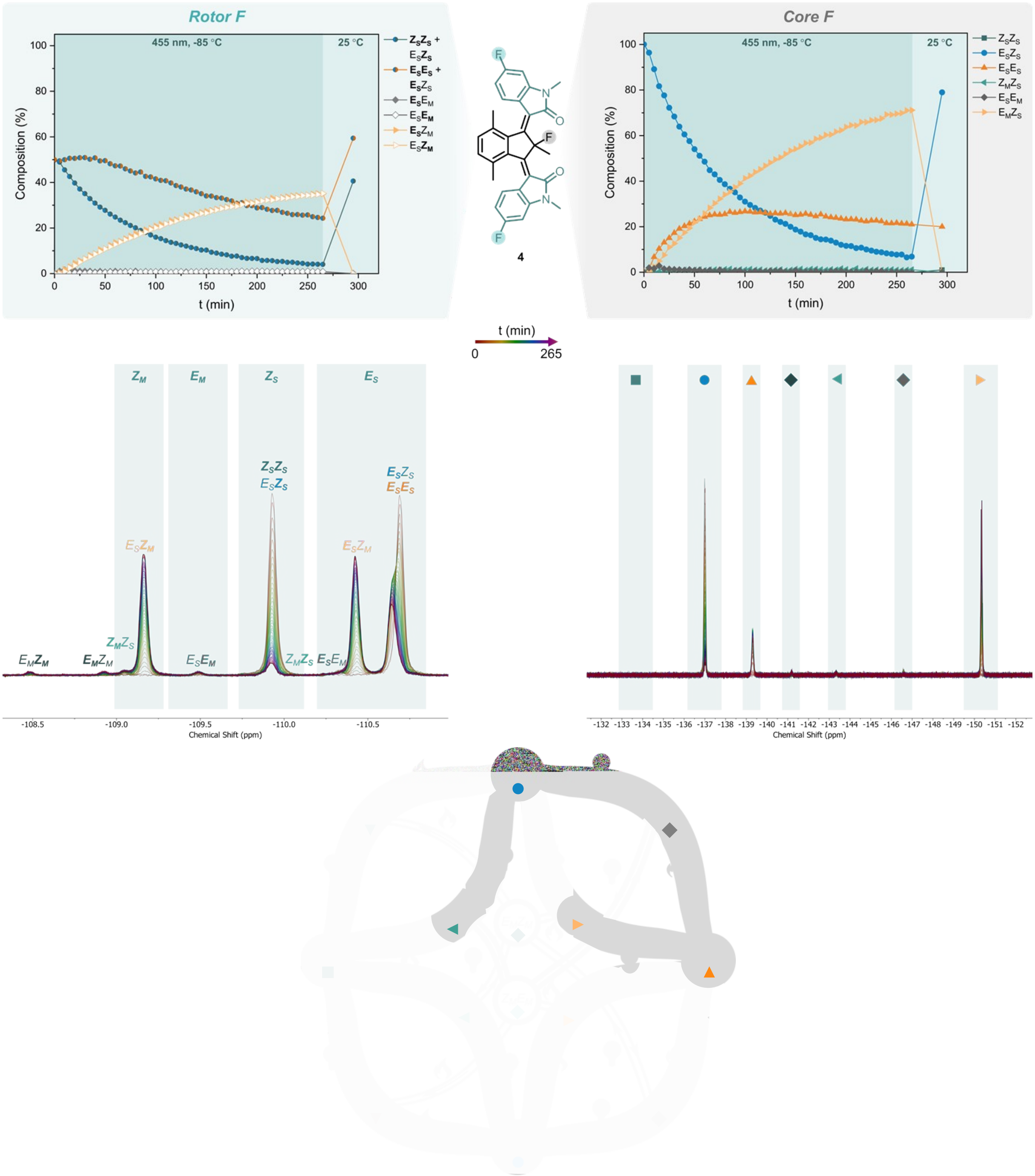

***Figure S30**. Top: Sample of (E_SZ_S)-**4** irradiated to PSS with 455 nm at -85 °C. The sample was subsequently warmed up to room temperature. The spectral changes of the core (right) and rotor (left) fluorine atoms were followed. The measurements were performed at the labelled temperatures. Middle: The corresponding ^{19}F NMR superimposed spectra (470 MHz) of (E_SZ_S)-**4** (6.1 mM in CD_2Cl_2) upon irradiation at -85 °C with isomer assignment. For the rotor fluorine, the rotor shown in bold is the contributing rotor to the signal. Bottom: Motor operation overview under these conditions (t =0 – 265 min).*

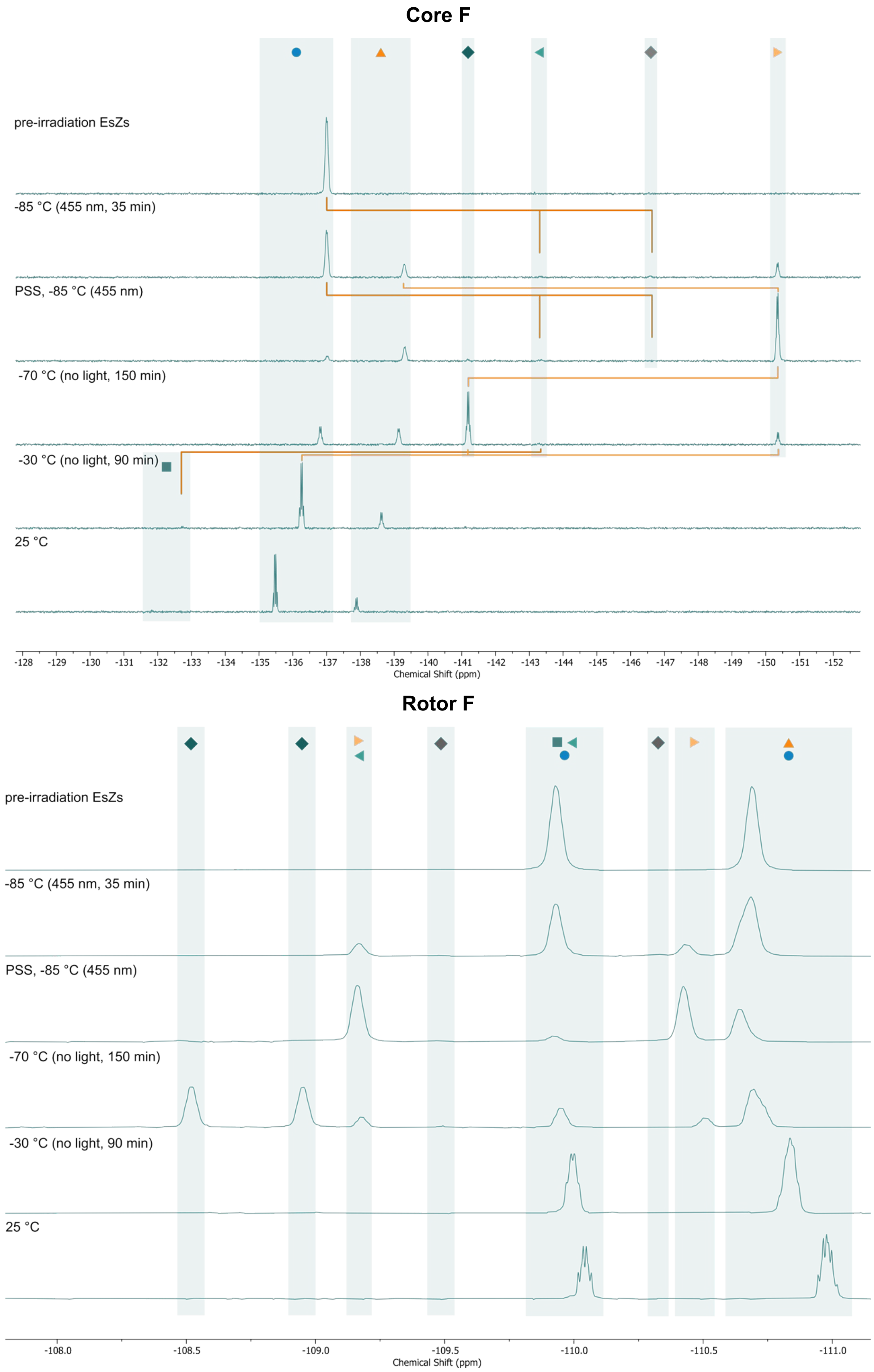

Figure S31. *The* [19]*F NMR spectra (470 MHz) of* (E_SZ_S)-**4** *(6.1 mM in* CD_2Cl_2*) upon irradiation at -85 °C, partial and full relaxation in the dark, zoomed in on the core fluorine region. The spectra were recorded at the labelled temperatures.*

Thermal relaxation of PSS (455 nm, -85 °C) of **4** at -70 °C

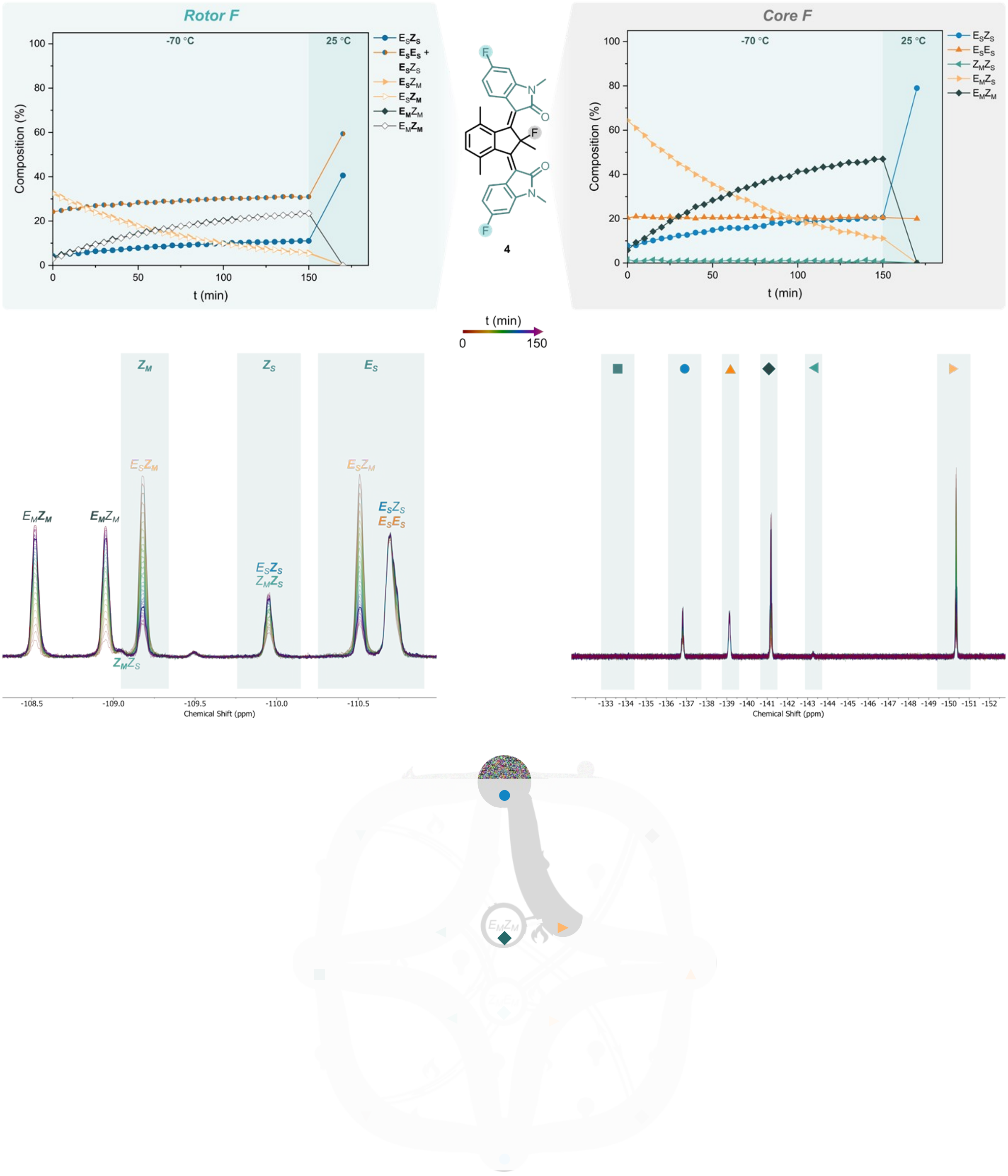

***Figure S32**. Top: Thermal decay in the dark of pre-irradiated (E_SZ_S)-**4** (PSS with 455 nm at -85 °C) at -70 °C. The sample was subsequently warmed up to room temperature. The measurements were performed at -70 °C. The spectral changes of the core (right) and rotor (left) fluorine atoms were followed. For the rotor fluorine, the rotor shown in bold is the contributing rotor to the signal. Bottom: Motor operation overview under these conditions (t =0 – 150 min).*

Full thermal relaxation of partially relaxed PSS (455 nm, -85 °C, then dark, -70 °C) of **4** at -30 °C

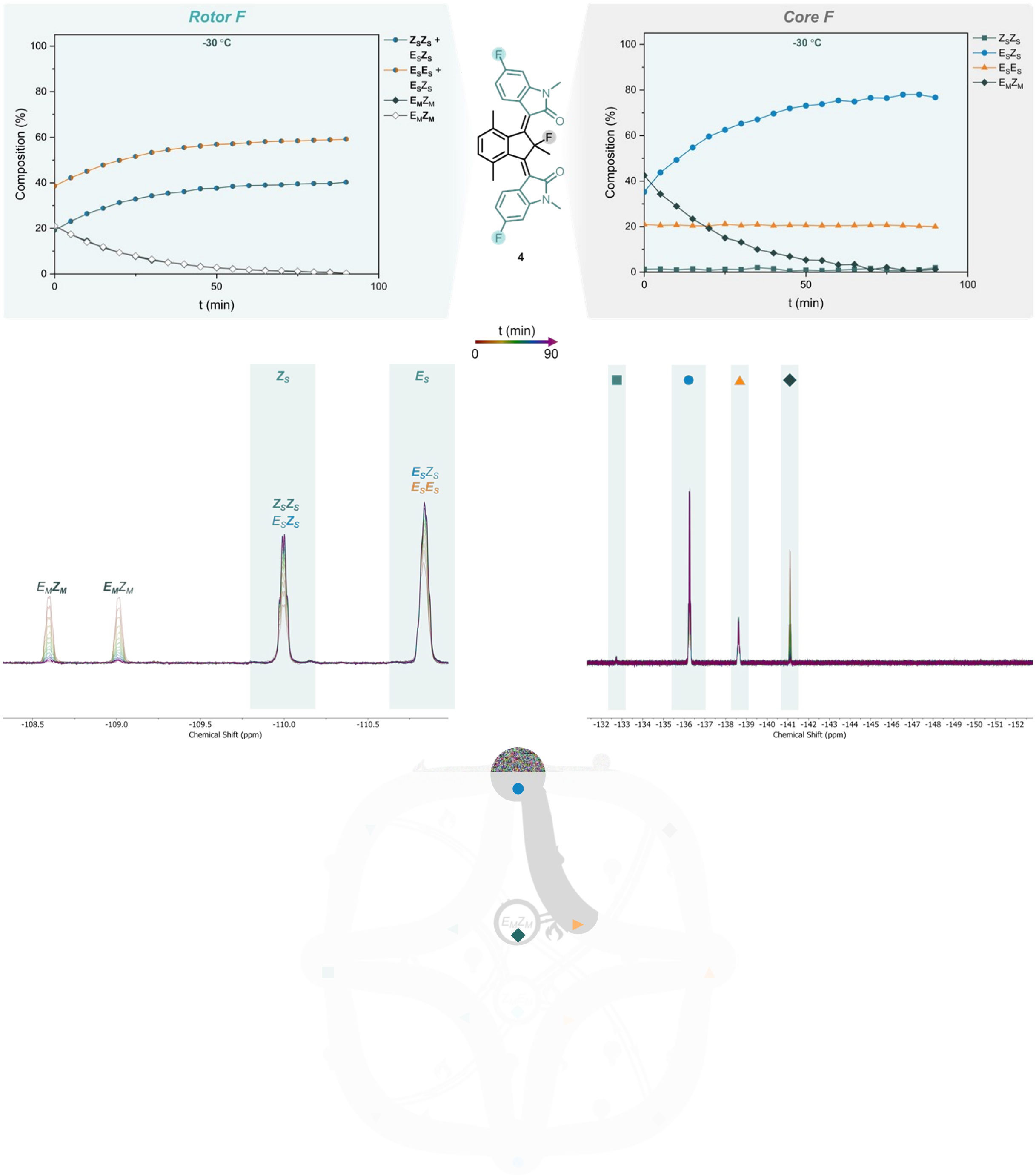

Figure S33. *Top: Thermal decay in the dark of pre-irradiated (E_SZ_S)-**4** (PSS: 455 nm at -85 °C, then dark, -70 °C) at -30 °C. The sample was subsequently warmed up to room temperature. The measurements were performed at -30 °C. The spectral changes of the core (right) and rotor (left) fluorine atoms were followed. For the rotor fluorine, the rotor shown in bold is the contributing rotor to the signal. Bottom: Motor operation overview under these conditions.*

Kinetic analysis

Fitting of the data using Origin was done with the default mono-exponential decay equation: $A = y_0 + A_1 e^{-t/(t1/2)}$, which provided the half-life ($t_{1/2}$) of interest. Fitting of the data using COPASI was performed using the default Levenberg-Marquardt algorithm with a tolerance of $1 \cdot 10^{-6}$ and an run iteration limit of 3000. The initial guesses of the rate constants for the kinetic parameter estimation were 1) that all species are in equilibrium with each other and 2) initial values of the kinetic constants were random values. Visual inspection of the value and associated error of each kinetic constant provided an indication of the relevance of each reaction. Kinetic constants with absolute values lower than 10^{-6} min^{-1} were approximated to 0. The respective reaction was deleted in the next iteration. This iterative process of fitting, inspection and model adaptation was repeated until the parameter estimation results no longer showed values indicative for further improvements to the model.

The obtained kinetic parameters for motors **2** and **4** are comparable to those previously determined for parent motor **1**[1] (see Table S2).

Table S2. *Comparison of the experimentally determined kinetic constants of the THI steps for functionalised motors* ***2*** *and* ***4****, and parent motor* ***1****.*

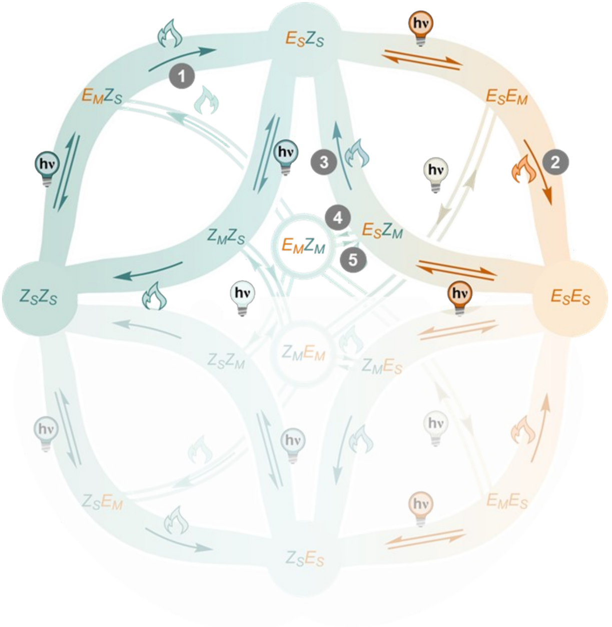

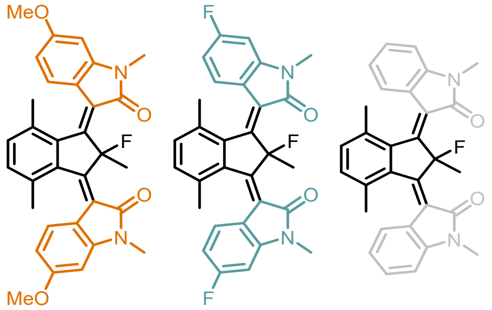

2 **4** **1**

	T (°C)	$\Delta^{‡}G$ (kcal mol^{-1})	$\Delta^{‡}G$ (kcal mol^{-1})	$\Delta^{‡}G_{parent}$ (kcal mol^{-1})	Details
1 - THI_E (E_MZ_S, E_SZ_S)					
In situ NMR irradiation of Z_SZ_S	-85	12.9	13.5	13.6	Figure S25<br>Figure S28
2 - THI_E (E_SE_M, E_SE_S)					
In situ NMR irradiation of Z_SZ_S<br>*In situ* NMR irradiation of E_SZ_S	-85	12.6	13.1<br>12.5	12.9	Figure S25<br>Figure S28<br>Figure S30
3 - THI_Z (E_SZ_M, E_SZ_S)					
NMR relaxation of E_SZ_M	-70	15.7	15.8	16.0	Figure S26<br>Figure S32
4 - CO-flip = THI_E (E_SZ_M, E_MZ_M)					
NMR relaxation of E_SZ_M	-70	15.1	15.3	15.6	Figure S26<br>Figure S32
5 - THI_Z (E_MZ_M, E_SZ_S)					
NMR relaxation of E_MZ_M	-30	17.9	17.7	18.0	Figure S27<br>Figure S33

Motor 2

*Relaxation kinetics of E_MZ_S and E_SE_M of motor **2** at -85 °C*

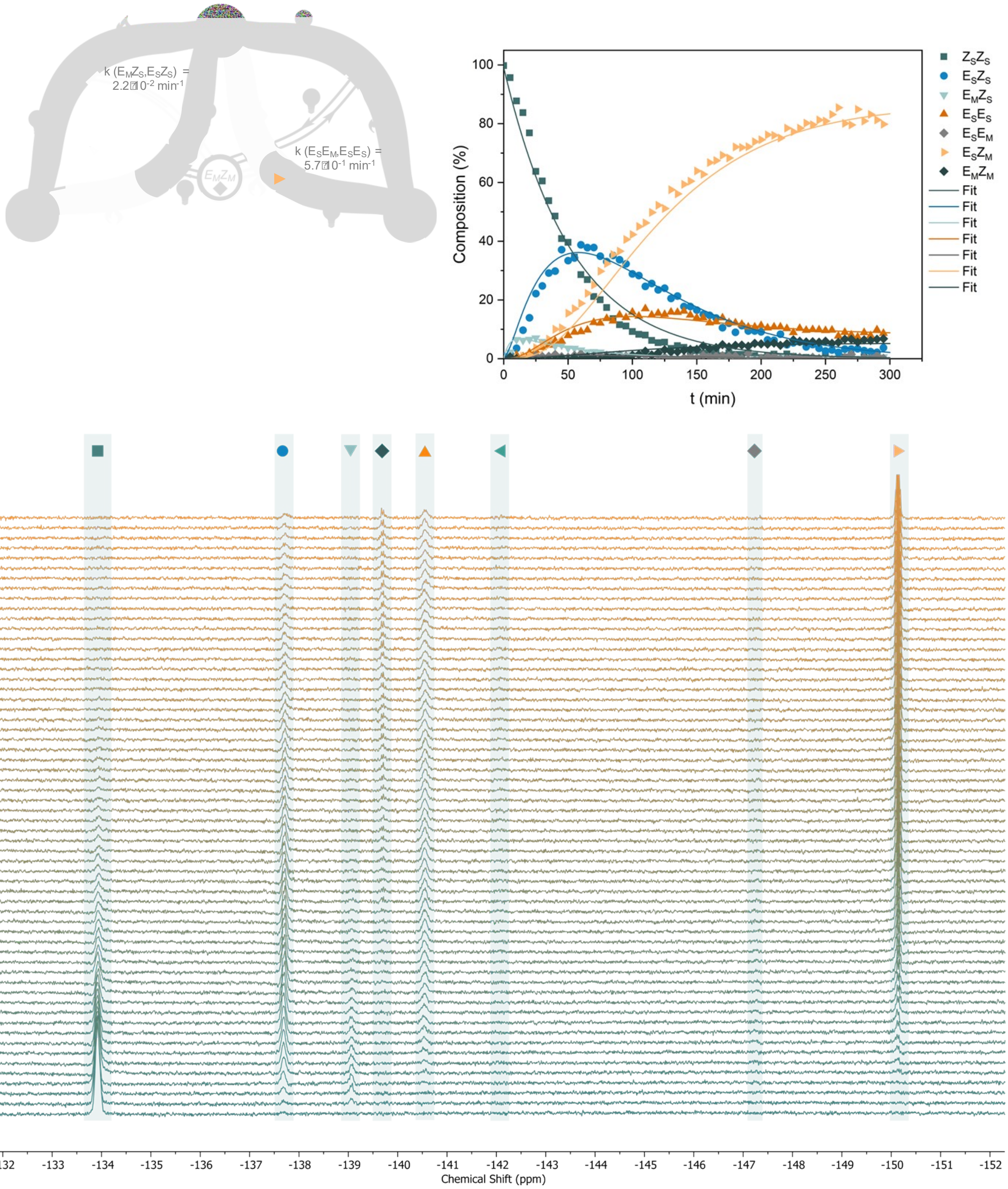

***Figure S34**. Proposed mechanism with rate constants (top left). Kinetic model fitting of (Z_SZ_S)-**2** upon irradiation to PSS with 455 nm light at -85 °C with COPASI (top right). The fit is based on the data from the NMR irradiation experiment (Figure S25). Stacked NMR data (bottom).*

*Relaxation kinetics of E_SZ_M and E_MZ_M of motor **2** at -70 °C*

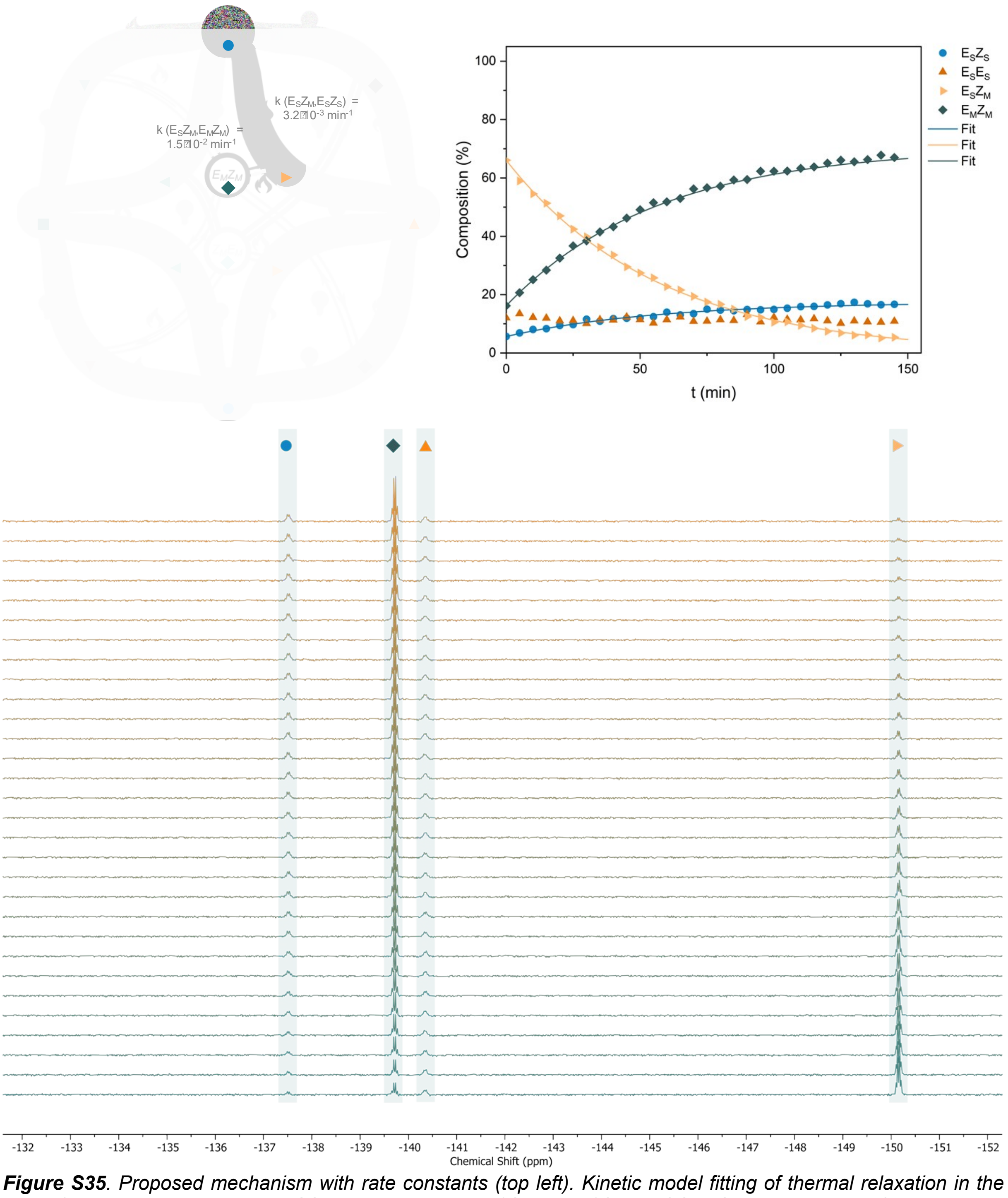

Figure S35. *Proposed mechanism with rate constants (top left). Kinetic model fitting of thermal relaxation in the dark of pre-irradiated (Z_SZ_S)-**2** (PSS with 455 nm at -85 °C) at -70 °C with COPASI (top right). The fit is based on the data from the NMR irradiation experiment (Figure S26). Stacked NMR data (bottom).*

*Relaxation kinetics of E_MZ_M of motor **2** at -30 °C*

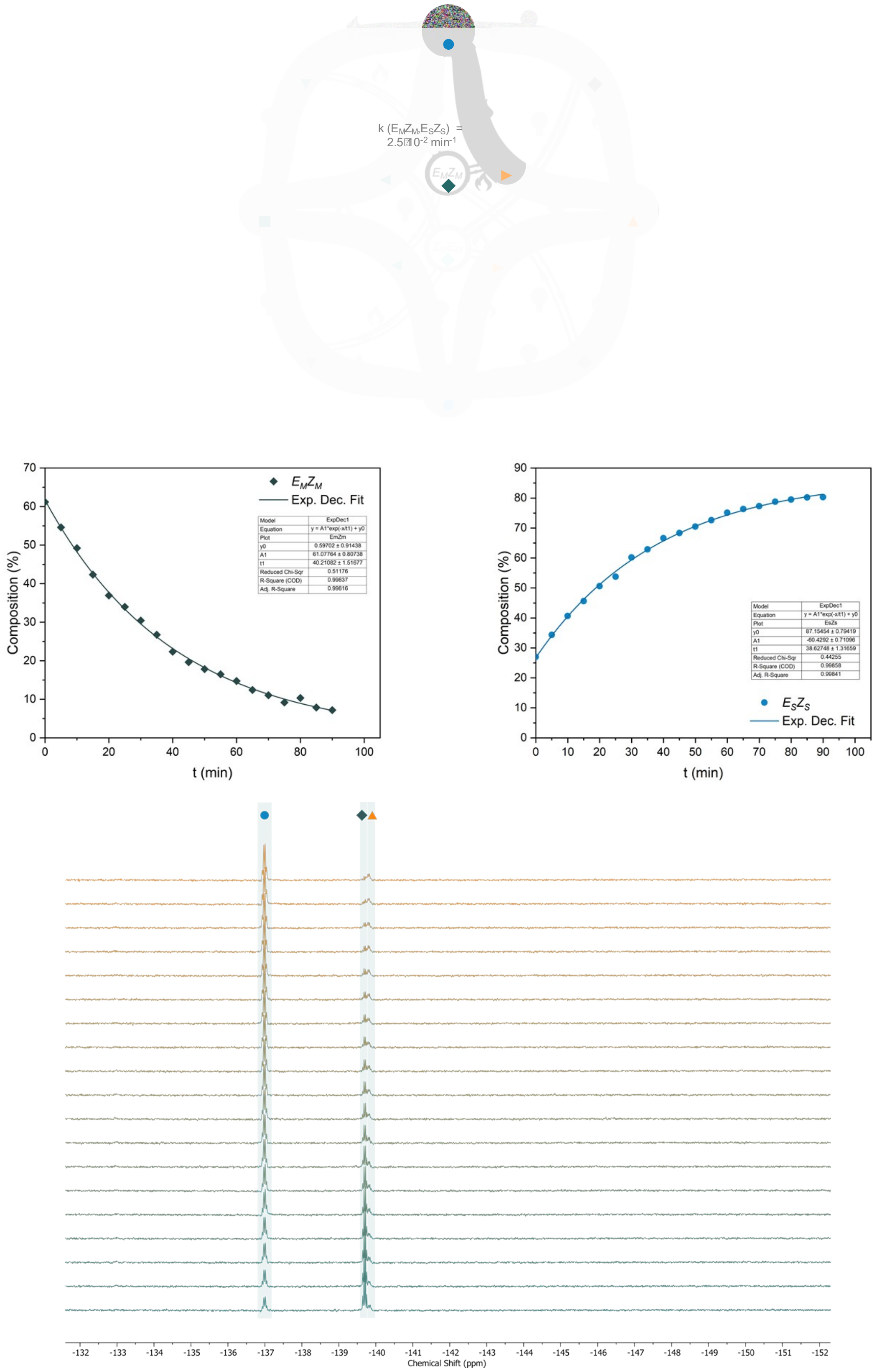

***Figure S36**. Proposed mechanism with rate constants (top). The traces of (E_MZ_M)-**2** (middle left) and (E_SZ_S)-**2** (middle right) in the thermal decay in the dark of pre-irradiated (Z_SZ_S)-**2** (PSS with 455 nm at -85 °C, then dark, -70 °C) at -30 °C versus time. The half-life ($t_{1/2}$) was obtained by fitting the data to the mono-exponential decay equation: $A = y_0 + A_1e^{-t/(t1/2)}$ using Origin software. The fit is based on the data from the NMR irradiation experiment (Figure S27). Stacked NMR data (bottom).*

Motor 4

*Relaxation kinetics of E_MZ_S and E_SE_M of motor **4** at -85 °C*

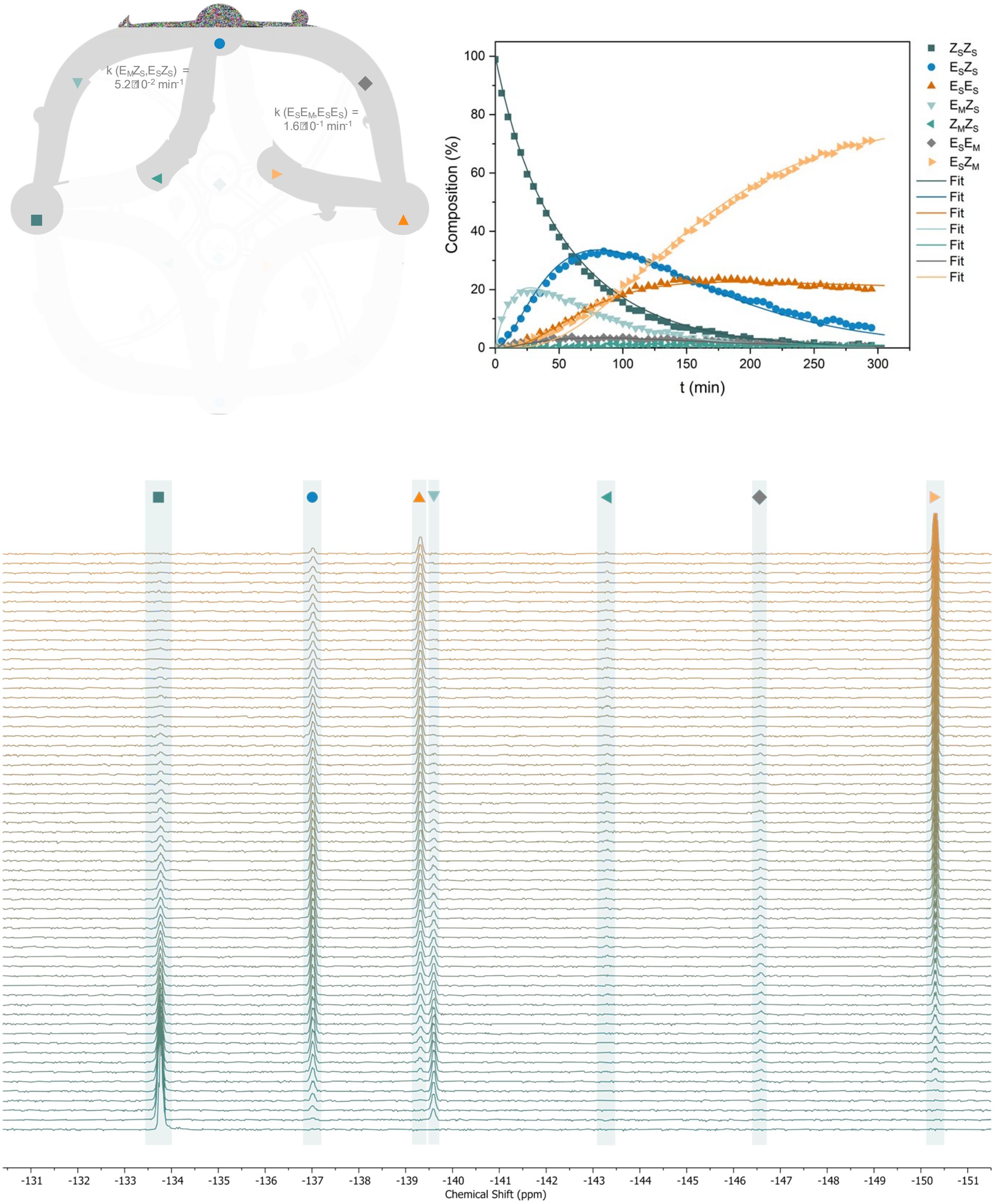

Figure S37. *Proposed mechanism with rate constants (top left). Kinetic model fitting of (Z_SZ_S)-**4** upon irradiation to PSS with 455 nm light at -85 °C with COPASI (top right). The fit is based on the data from the NMR irradiation experiment (Figure S28). Stacked NMR data (bottom).*

*Relaxation kinetics of E_ME_S of motor **4** at -85 °C*

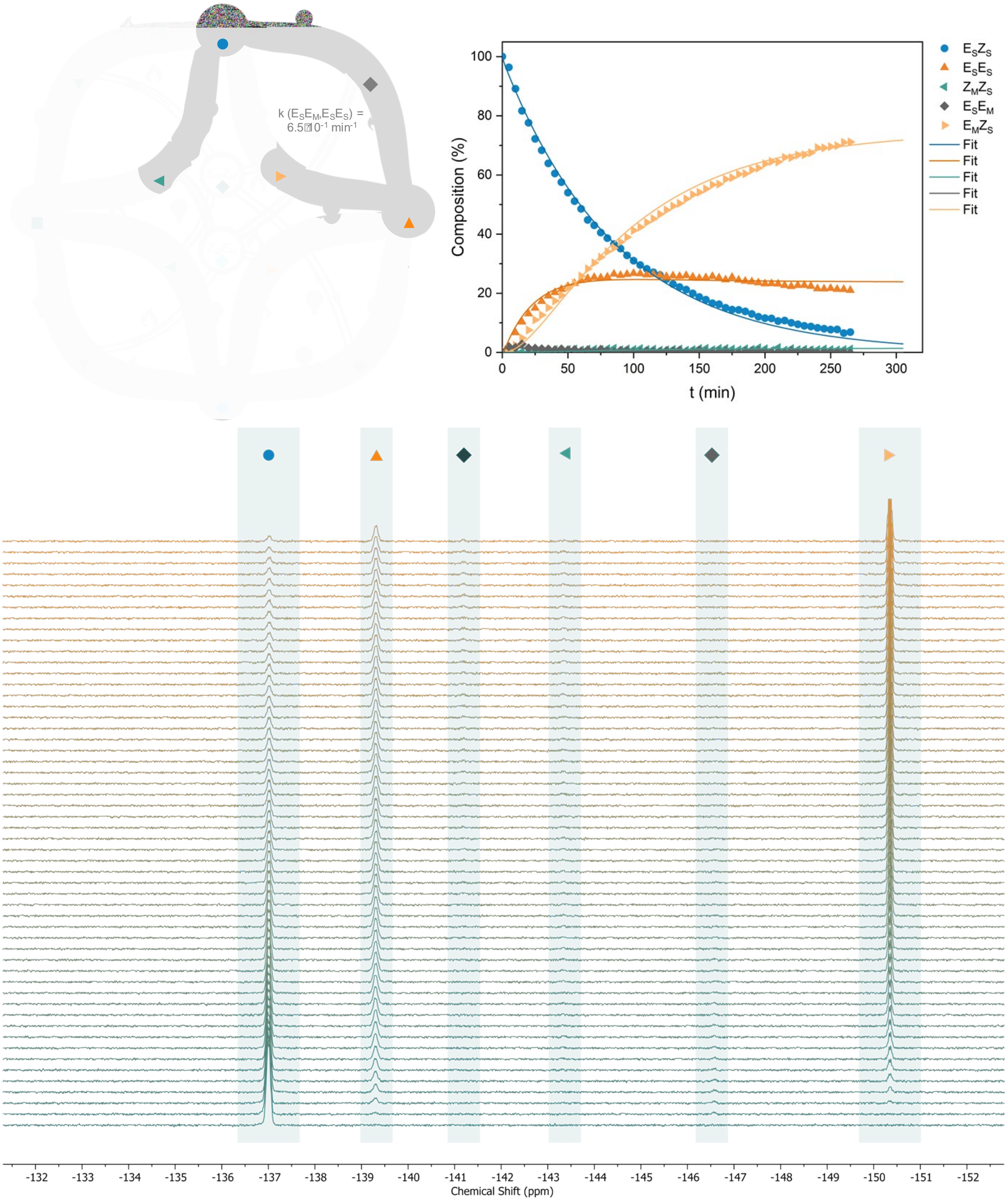

***Figure S38**. Proposed mechanism with rate constants (top left). Kinetic model fitting of (E_SZ_S)-**4** upon irradiation to PSS with 455 nm light at -85 °C with COPASI (top right). The fit is based on the data from the NMR irradiation experiment (Figure S30). Stacked NMR data (bottom).*

*Relaxation kinetics of E_SZ_M and E_MZ_M of motor **4** at -70 °C*

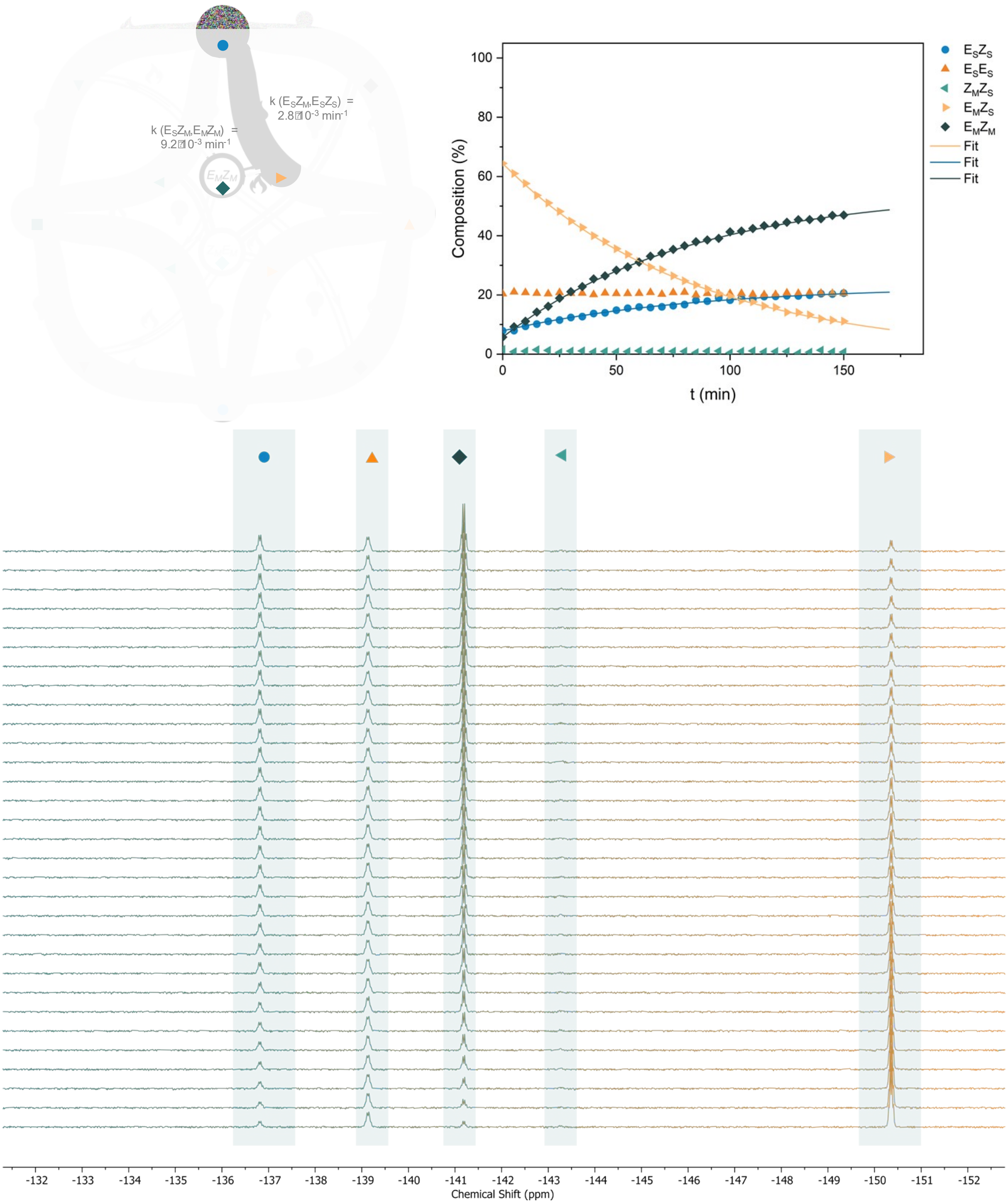

***Figure S39**. Proposed mechanism with rate constants (top left). Kinetic model fitting of thermal relaxation in the dark of pre-irradiated (E_SZ_S)-**4** (PSS with 455 nm at -85 °C) at -70 °C with COPASI (top right). The fit is based on the data from the NMR irradiation experiment (Figure S29). Stacked NMR data (bottom).*

Relaxation kinetics of EMZM of motor 4 at -30 °C

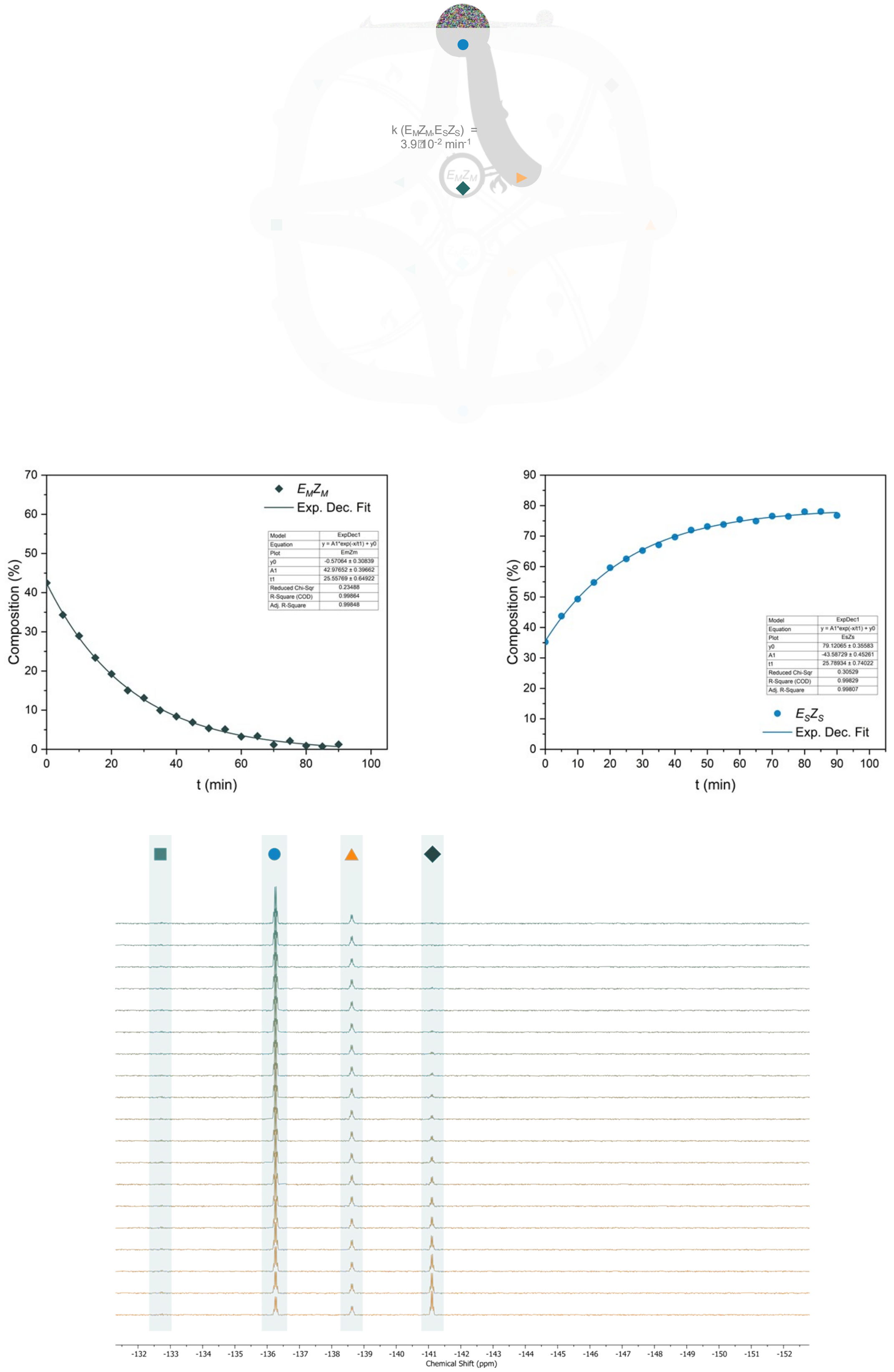

Figure S40. *Proposed mechanism with rate constants (top). The traces of (E_MZ_M)-**4** (middle left) and (E_SZ_S)-**4** (middle right) in the thermal decay in the dark of pre-irradiated (E_SZ_S)-**4** (PSS with 455 nm at -85 °C, then dark, -70 °C) at -30 °C versus time. The half-life ($t_{1/2}$) was obtained by fitting the data to the mono-exponential decay equation: $A = y_0 + A_1e^{-t/(t1/2)}$ using Origin software. The fit is based on the data from the NMR irradiation experiment (Figure S33). Stacked NMR data (bottom).*

5. Membrane-spanning Molecular Motor

Vescile preparation

Small and giant unilamellar vesicles were prepared according to a modified reported procedure[13] for the incorporation of similar molecular motors in such vesicles, see the respective sections below.

Measurements on Membrane Fluidity (Diffusion coefficient)

Small Unilamellar Vesicles (SUVs): were prepared by the thin film hydration method followed by extrusion through polycarbonate membranes of the desired size (100 nm). Briefly, 1-palmitoyl-2-oleoyl-glycero-3-phosphocholine (POPC, 4 mg/mL, *Avanti Polar Lipids, USA*) was mixed with motor **8** (1 mg/mL) at the specified molar ratio (100:10), unless otherwise indicated (saturation concentration = maximum uptake of photoactive compound). The mixture, in $CHCl_3$, was transferred to a glass vial, and the solvent was removed using a stream of N_2 gas followed by vacuum drying in a desiccator (30 min) to ensure complete removal of organic solvents. To initiate hydration, Milli-Q water was added to the dried lipid film, achieving a final lipid concentration of 10 mM. This was followed by vigorous vortexing to create a suspension of multilamellar vesicles. To promote vesicle homogenization and unilamellarity, the samples underwent three freeze-thaw cycles. Following this, sonication and extrusion was performed using the Avanti Mini Extruder (*Avanti, USA*) with an appropriate polycarbonate membrane with a pore size of 100 nm. This extrusion process was repeated 15 times, resulting in the formation of small unilamellar vesicles. Subsequently, these suspensions were further diluted as necessary.

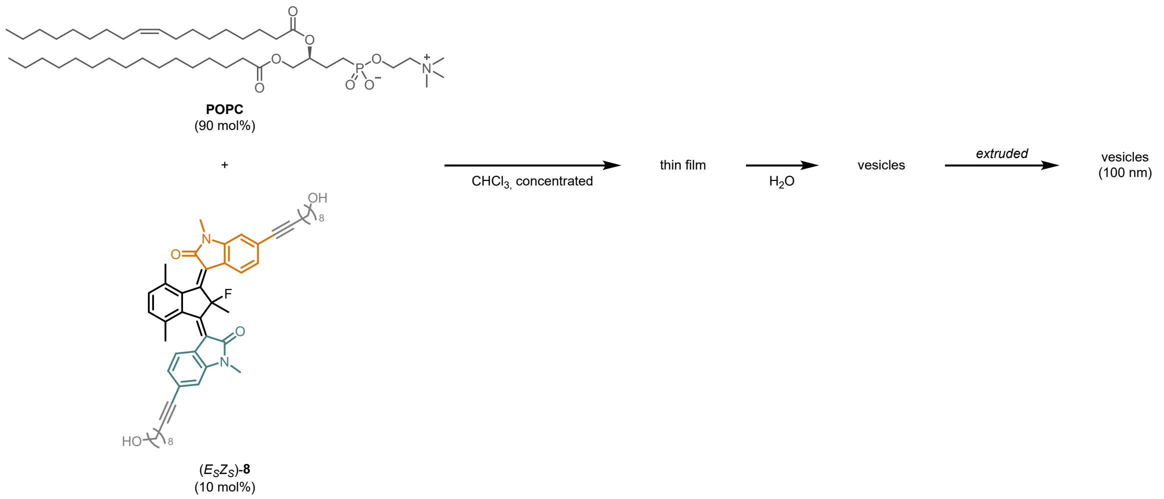

Figure S41. *Preparation of small unilamellar vesicles (SUVs) with incorporated motor (E_SZ_S)-**8**.*

Fluorescence Correlation Spectroscopy (FCS): We employed FCS to analyse the diffusion coefficient of the lipid-conjugated dye A655-DOPE (0.01 mol%) within small lipid bilayers imbedding **8**. To mitigate against the overestimation of diffusion times resulting from imperfect focal position or membrane undulations, we adopted Z-scan FCS. This method involves performing measurements at varied focal positions along the membrane normal, followed by calculation of autocorrelation curves specific to each position. FCS was performed with Zeiss LSM980 laser scanning confocal microscope (*Carl Zeiss, Germany*). A custom macro was used to define z-locations above and below the desired focus and each z-position was measured for 30 sec acquisition time. All measurements were performed at 28 °C. The individual autocorrelation curves were calculated and fitted with custom MATLAB and Python scripts, with 2D FCS diffusion fits.

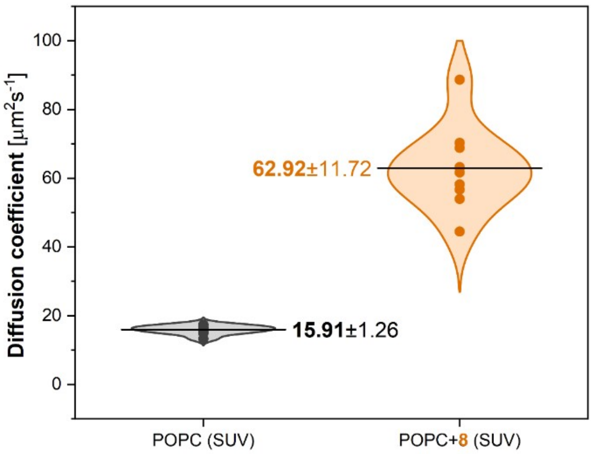

Figure S42. *Diffusion coefficient average values obtained from fluidity measurement of POPC lipid vesicles (100 nm, diameter) containing 0 mol% (black) or 10 mol% (orange) of **8**. Mean and standard deviation of ten independent measurements*

UV-Vis Absorption Studies

The incorporation of motor (E_SZ_s)-**8** into a lipid system (specifically SUVs of POPC lipids) was studied using UV-Vis absorption spectroscopy. The absorption spectrum of SUVs prepared with 10 mol% of motor (E_SZ_s)-**8** shows absorption bands in the 280-520 nm range (see Figure S43 (top right)), which can be attributed to the motor unit and thus confirms the incorporation of motor (E_SZ_s)-**8** into the SUVs. The absorption bands of imbedded motor (E_SZ_s)-**8** resembles those of motor (E_SZ_s)-**3** in CH_2Cl_2. Upon irradiation with 455 nm light, small changes in the absorption spectrum occurred until the PSS was reached. The observed changes are in line with our results obtained for other functionalised bridged-isoindigo molecular motors in an organic solvent (see also the section UV/Vis Absorption Spectra). These results show that the imbedded motor is still operational.

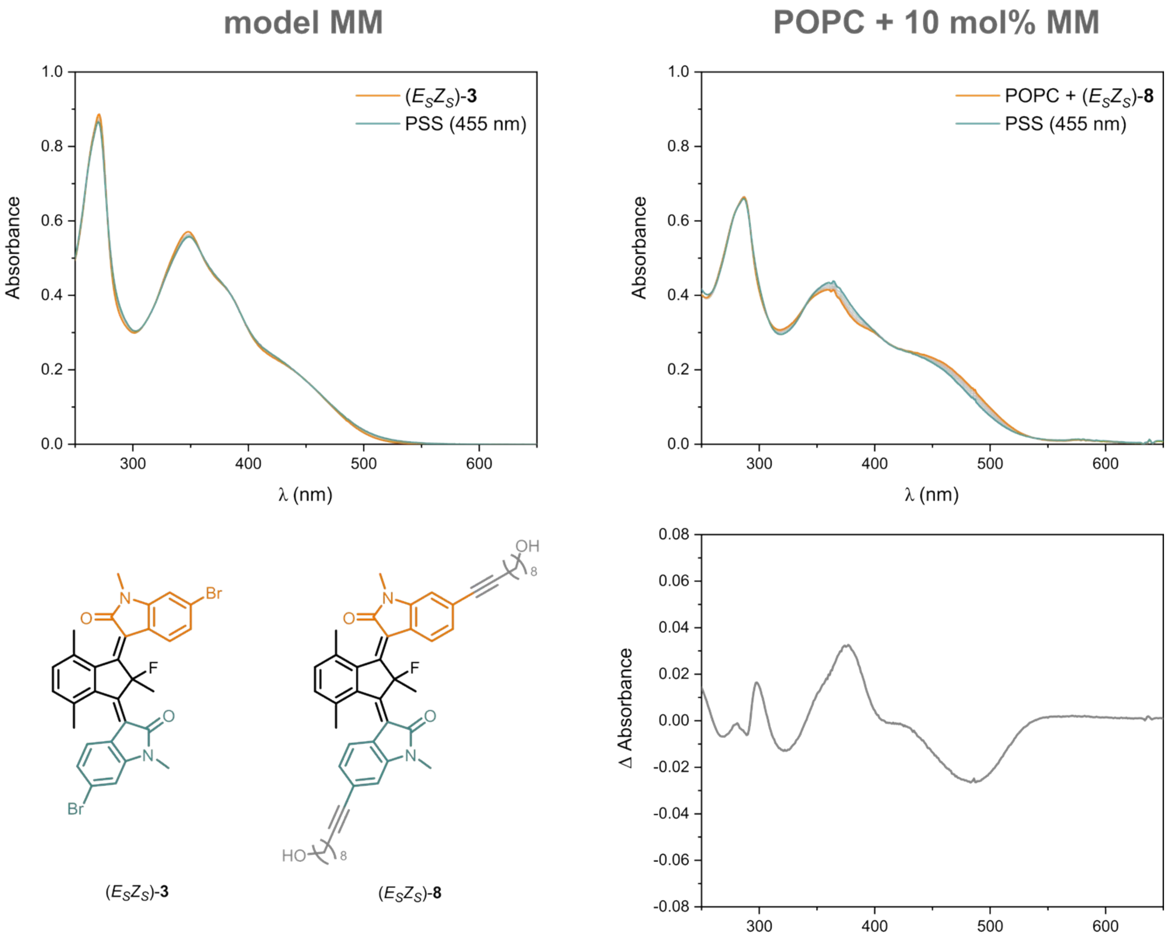

***Figure S43**. UV/Vis absorption spectra of model motor (E_SZ_S)-**3** in CH_2Cl_2 (~3 × 10^{-5} M, 20 °C) (left) and motor (E_SZ_S)-**8** in a lipid environment (10 mol%, SUVs 100 nm Ø, MilliQ, 5 °C) upon irradiation with 455 nm.*

Linear Dichroism

The linear dichroism absorption spectra were obtained using a JASCO J-815 spectropolarimeter equipped with a Dioptica microvolume flow LD cell (Dioptica Scientific Ltd.). This cell consists of a coaxially aligned stationary quartz rod (D = 2.5 mm) and a rotating quartz cylinder (ID = 3 mm). The revolution speed of 3000 rpm was used. The measurements were performed with 60 µL of (2 mg/mL lipid content) aqueous solution at room temperature using pure water as a reference. Linear dichroism was calculated with the following equation:

$$Linear\ Dichroism\ (LD) = A_{\parallel} - A_{\perp}$$

where $A_{\parallel}$ and $A_{\perp}$ are the parallel absorbance and the perpendicular absorbance of the polarised light, respectively. The sign of the LD signal provides insights into the orientation of the motor **8** in the lipid membrane. A negative LD value shows that the electronic transition dipole moment of the motor is orientated perpendicular to the applied shear force, that is parallel to the membrane normal. On the other hand, a positive LD value results from a parallel orientation to the applied shear force, which is orthogonal to the membrane normal.

TD-DFT calculations were performed to obtain the electronic transition moment associated with the photoisomerisation transition ($S^0 \rightarrow S^1$) of a truncated analogue of motor **8** (see also the section Computational Details).The orientation of the calculated electronic transition moment nearly aligns with the two C=C double bonds of the motor (Figure S41) and thus provides approximation of the motor's alignment in the membrane's bilayer.

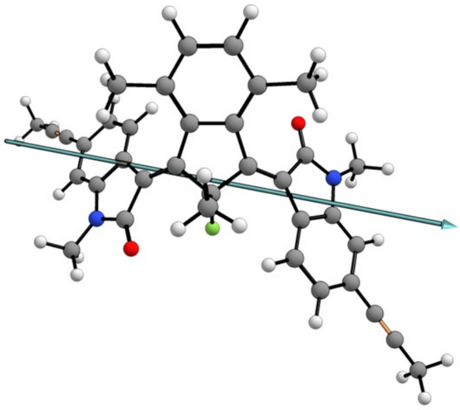

***Figure S44**. Transition dipole moment of a simplified analogue of ($E_S Z_S$)-**8** (TD-DFT ωB97X-D3/def2-TZVPP//r²SCAN-3c).*

The LD measurement performed with motor containing SUVs showed a positive LD signal (in the absorption range of motor **8** corresponding to the C=C transition dipole moment) which suggests a preferential perpendicular alignment of the motor's C=C bonds with respect to the membrane normal (Figure S45).

Irradiation of the sample induced a small change in the LD signal, providing additional evidence that the imbedded motor is still operational. The positive LD signal arising from the motor is maintained which shows that the alignment is not disrupted by the motor functioning.

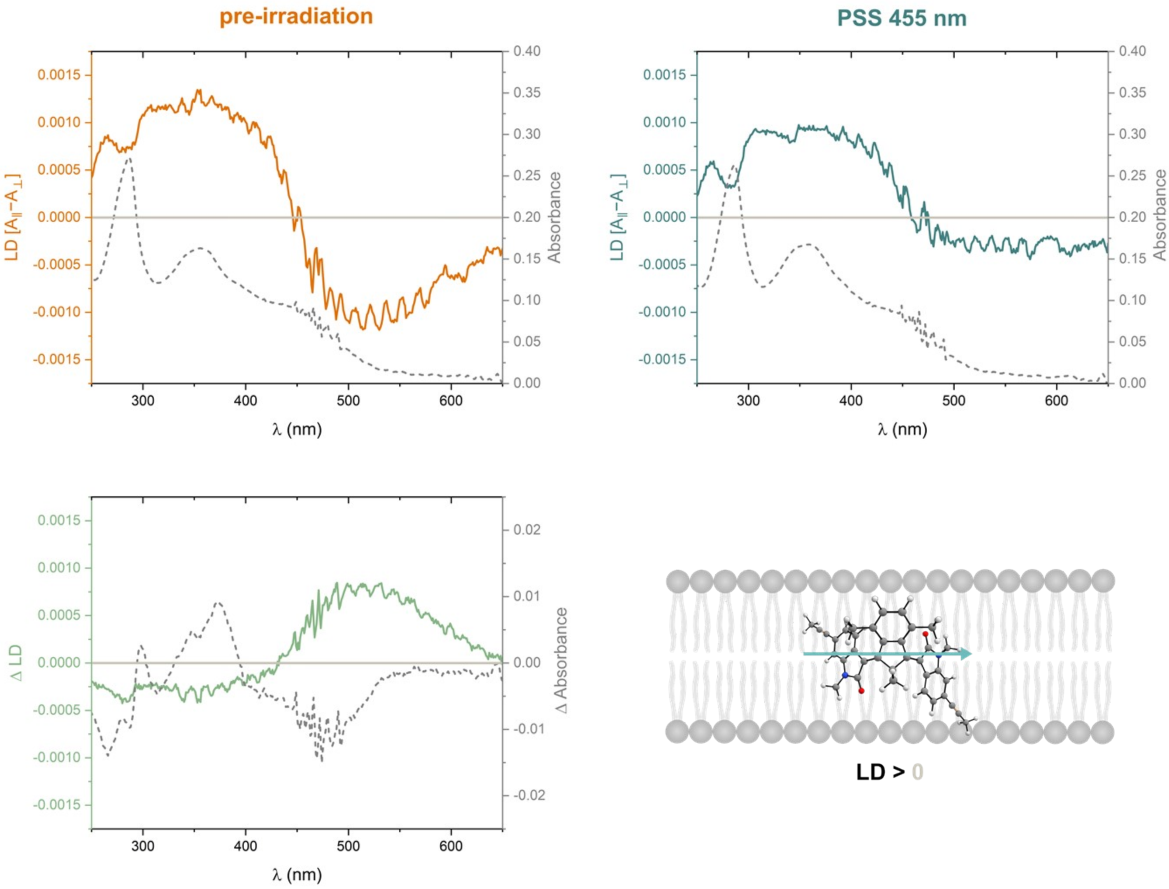

Figure S45. *Top: UV/Vis absorption (grey) and linear dichroism spectra of motor* (E_SZ_S)*-**8** in a lipid environment (10 mol%, SUVs 100 nm Ø, MiliQ, 5 °C) before (orange) and after (blue) irradiation with 455 nm. Bottom: The differential spectra and schematic representation of the preferential membrane alignment of motor **8**.*

Cryo-TEM

Motorised SUV (10 mol% of **8**) samples of 2.5 µL (2 mM) were placed on a glow-discharged holy carbon-coated grid (Quantifoil 3.5/1, QUANTIFOIL Micro Tools GmbH). After blotting, the corresponding grid was rapidly frozen in liquid ethane (Vitrobot, FEI) and kept in liquid nitrogen until the measurement. The grids were observed with a Gatan model 626 cryostage in a Tecnai T20 Field Electron and Ion Company (FEI) cryo-electron microscope operating at 200 keV. Cryogenic transmission electron microscopy (cryo-TEM) images were recorded under low-dose conditions on a slow-scan Charge-Coupled Device (CCD) camera. All processes were performed in the dark.

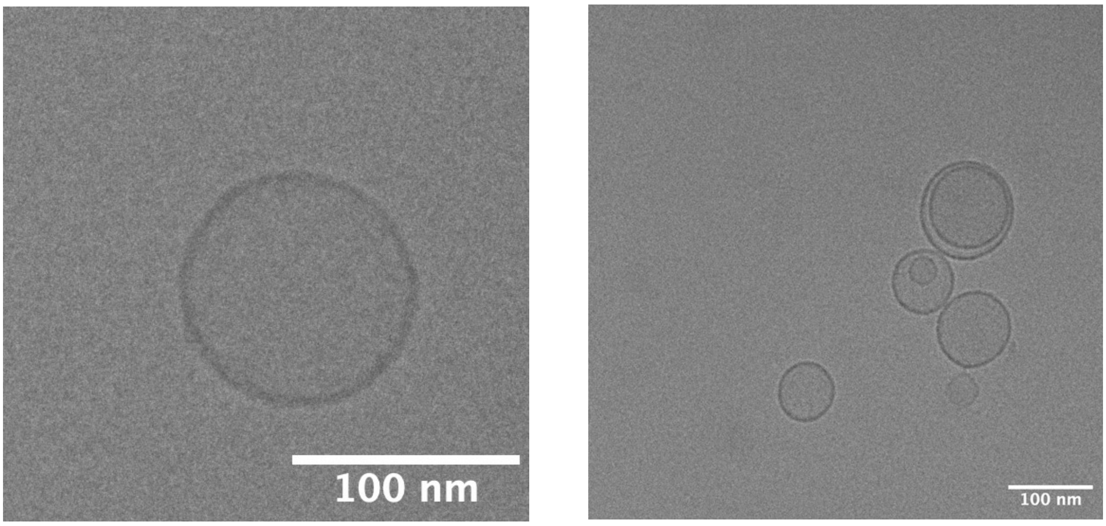

***Figure S46**. Cryo-TEM images of POPC-based SUVs prepared with 10 mol% **8**.*

Measurements of Membrane Fluctuations

Giant Unilamellar Vesicles (GUVs): Giant Unilamellar Vesicles (GUVs) were prepared through a PVA-assisted swelling method, following established procedures[13,14]. Briefly, a 100µL solution of polyvinyl alcohol (PVA, MW:145 000, *Merck KGaA, Germany*) was dissolved in Milli-Q water at a concentration of 5% (w/v). This PVA solution was deposited onto a plasma-cleaned coverslip (ϕ 30mm, #1.5). After a 5 min incubation, the excess PVA solution was removed, and the coverslip was then dried in a 60 °C oven for 15 min. Using a spin-coating device (*Schaefer Technologie GmbH, Germany*), a 50 µL solution of the desired lipid and molecular motor (100:10), prepared at a lipid concentration of 1mg/mL, was deposited onto the coverslip. The deposition was conducted at a rotation speed of five revolutions per second (rps) for a duration of 55 s. Subsequently, the coverslips were placed in a desiccator for 15 min. To initiate the formation of GUVs, 1 mL of a sucrose buffer with a concentration of 300 mM was deposited onto the coverslip and allowed to swell the vesicles in the dark for 1 h. After that, the resulting GUVs were diluted in a glucose buffer with an equimolar concentration of glucose 300 mM. All experiments were conducted after ensuring that the osmolality of the inner aqueous solution was 5% higher than the osmolality of the outer aqueous solution. For microscopy experiments, 1 mol% of lipid-conjugated dye A655-DOPE was used.

Fluorescence Microscopy: Fluorescence imaging was performed on a Zeiss Elyra 7 system for initiated area expansion analysis, using 405 and 641 nm laser lines. For the image capture alpha Plan-Apochromat 63x/1.46 Oil Korr M27 Var2 (Carl Zeiss, Germany) oil objective was used. The images were acquired on pco.edge sCMOS cameras (pco.edge 4.2 CLHS) at 100 ms exposure time. Images were processed using the Fiji open-source platfrom[15]. At least 30 vesicles were analysed. GUVs with diameters less than 20 µm or those that showed perturbations (fluctuations, tubulations, and budding) prior to sample irradiation were excluded from the analysis.

Spinning Disk Microscopy: Spinning disk confocal imaging was performed on a Nikon/Yokogawa CSU-W1 spinning disk confocal microscope for image and videos acquisition, using 405, and 641 nm laser lines. The 50 µm pinhole spinning disk was used at 4000 rpm. The sample was illuminated through a Nikon Apo TIRF 60x Oil DIC N2 immersion oil objective and the images were acquired in pco.edge sCMOS cameras (pco.edge 4.2 LT USB) at 100 ms exposure time. Images were processed using the Fiji open-source platfrom[15].

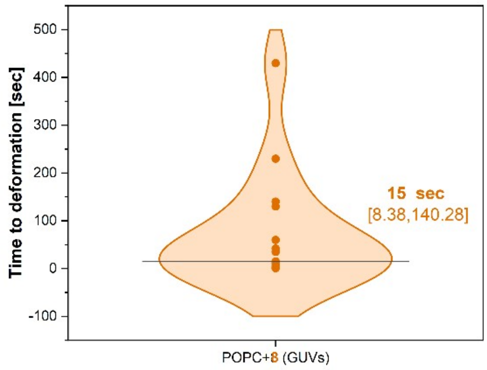

***Figure S47**. Reported times for initiated area expansion observations in giant lipid vesicles incorporating 10 mol% of **8**. Violin plot shows individual datapoints of 15 independent vesicles with black line representing median ± [95% confidence interval].*

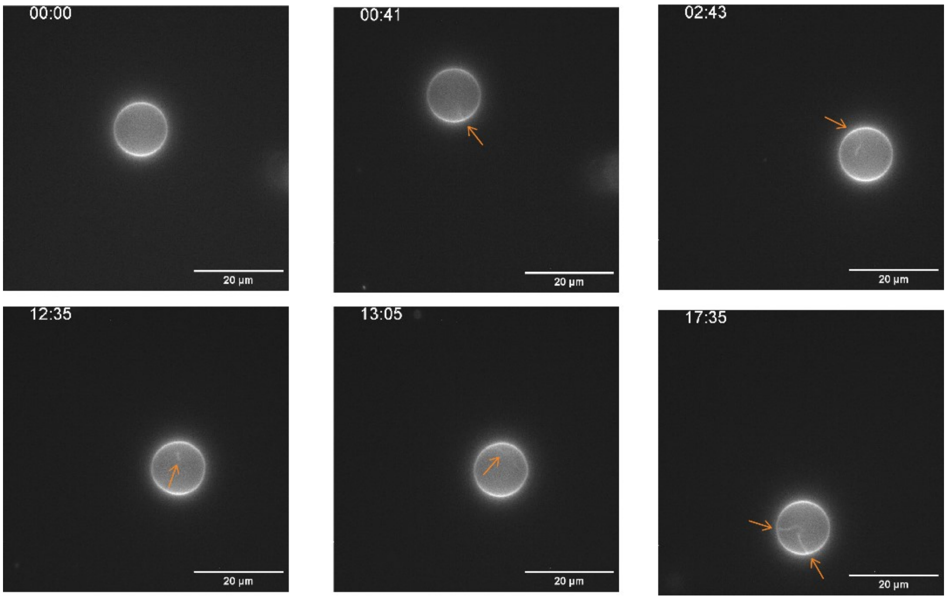

Figure S48. *Representative time-lapse of GUVs containing 10 mol% of* ***8*** *under 405 nm light exposure. Arrows indicate observed tubulations, resulting from lipid membrane area expansion.*

<Supplementary videos captions>

Supplementary Video 1. *Process of lipid area expansion upon irradiation of giant lipid vesicles containing 10 mol% of* ***8****.*

Supplementary Video 2. *Process of lipid area expansion upon irradiation of giant lipid vesicles containing saturation concentration of* ***8****.*

6. Computational Details

All calculations were done using the ORCA 5.0.4 software package.[16] Geometry optimisations were performed at the r^{2}SCAN-3c level of theory.[17] Solvation was included in all calculations using conductor-like polarisable continuum models (CPCM)[18] with parameters for CH_2Cl_2 as the solvent. The stationary points were confirmed using frequency calculations and evaluation of the number of imaginary frequencies (0 for minima and 1 for transition states). Using the optimised structures of the stable states of truncated motor **8**, full TD-DFT calculations were performed at a ωB97X-D3/def2-TZVPP (performed with TDA) level of theory.[19–21]

Optimised structure of truncated motor **8**

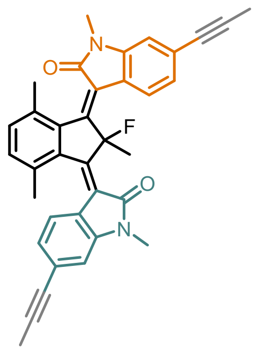

70
C36N2H29O2F

C	0.648936000	3.387710000	-2.044709000
C	-0.739247000	3.342062000	-2.096493000
C	-1.470948000	2.220652000	-1.690101000
C	-0.730317000	1.162253000	-1.143391000
C	0.699951000	1.206033000	-1.098092000
C	1.415672000	2.310129000	-1.589239000
C	-1.154630000	-0.180158000	-0.794676000
C	0.055286000	-1.075879000	-1.080815000
C	1.195317000	-0.107846000	-0.750610000
C	2.361658000	-0.460784000	-0.141598000
C	-2.313770000	-0.603567000	-0.218620000
C	3.211757000	0.335026000	0.725207000
C	4.278683000	-0.491690000	1.142010000
N	4.153542000	-1.741934000	0.547316000
C	3.003408000	-1.815735000	-0.222492000
C	-3.190630000	0.322595000	0.577579000
N	-4.233222000	-0.443769000	1.060607000
C	-4.090690000	-1.772568000	0.679772000
C	-2.905522000	-1.928166000	-0.077937000
C	3.073040000	1.593129000	1.305281000
C	4.022489000	2.036446000	2.219891000
C	5.114653000	1.224817000	2.582526000
C	5.237009000	-0.074542000	2.044195000
C	-4.948942000	-2.816393000	0.962410000
C	-4.624090000	-4.092951000	0.459111000
C	-3.461656000	-4.261916000	-0.316090000
C	-2.613793000	-3.193329000	-0.587190000

C	-5.271914000	0.054591000	1.941678000
C	5.101110000	-2.828352000	0.704930000
O	2.684904000	-2.787370000	-0.899417000
O	-3.007430000	1.501082000	0.863088000
C	0.097088000	-1.573234000	-2.524138000
C	2.904024000	2.341937000	-1.763448000
C	-2.943758000	2.177704000	-1.958053000
H	1.161170000	4.262617000	-2.438059000
H	-1.275661000	4.184513000	-2.526964000
H	2.226545000	2.228000000	1.061244000
H	3.927244000	3.021004000	2.665743000
H	6.054239000	-0.718472000	2.351294000
H	-5.849158000	-2.672886000	1.550339000
H	-3.225540000	-5.247807000	-0.702877000
H	-1.725872000	-3.368909000	-1.180004000
H	-5.116985000	1.126655000	2.075033000
H	-6.260994000	-0.117764000	1.502559000
H	-5.223072000	-0.443567000	2.917118000
H	6.095153000	-2.524580000	0.357388000
H	5.168341000	-3.131515000	1.756344000
H	4.750977000	-3.672232000	0.107656000
H	-0.808832000	-2.136075000	-2.762919000
H	0.159545000	-0.714750000	-3.198570000
H	0.977164000	-2.205329000	-2.657614000
H	3.309930000	1.338771000	-1.921964000
H	3.162313000	2.971517000	-2.619336000
H	3.411730000	2.754362000	-0.883243000
H	-3.503349000	2.646803000	-1.142386000
H	-3.308203000	1.150274000	-2.048982000
H	-3.168079000	2.716919000	-2.883182000
F	0.060141000	-2.163679000	-0.209024000
C	6.083088000	1.701956000	3.503849000
C	6.908955000	2.109629000	4.291763000
C	-5.470396000	-5.198477000	0.734138000
C	-6.195056000	-6.140275000	0.972506000
C	-7.057945000	-7.268804000	1.259963000
H	-6.993101000	-8.022575000	0.468077000
H	-6.773747000	-7.746675000	2.204457000
H	-8.102656000	-6.950919000	1.344549000
C	7.894404000	2.600889000	5.234413000
H	7.453036000	2.730654000	6.228999000
H	8.732817000	1.902250000	5.323918000
H	8.291583000	3.570676000	4.914763000

7. NMR Spectra

Rotors

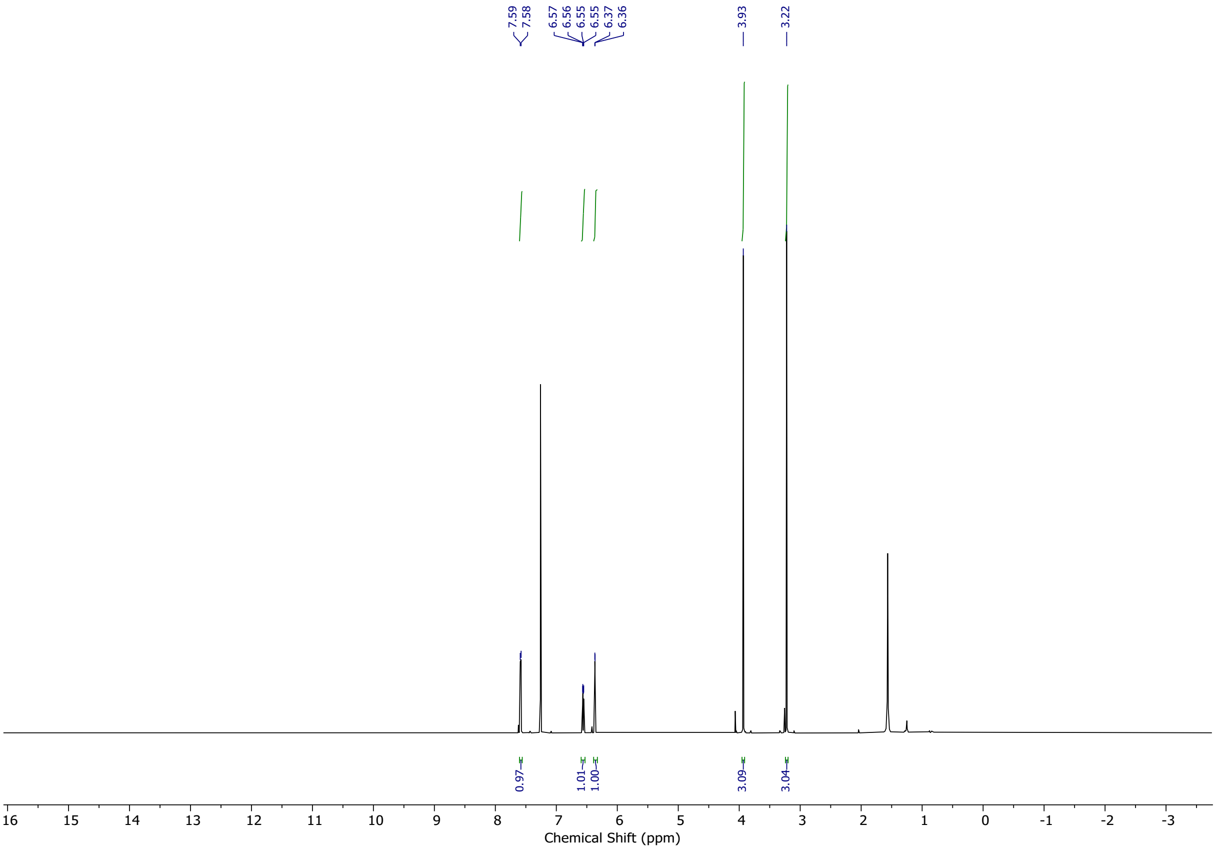

Figure S49. [1]*H NMR spectrum of **S2a** (CDCl$_3$, 20 °C).*

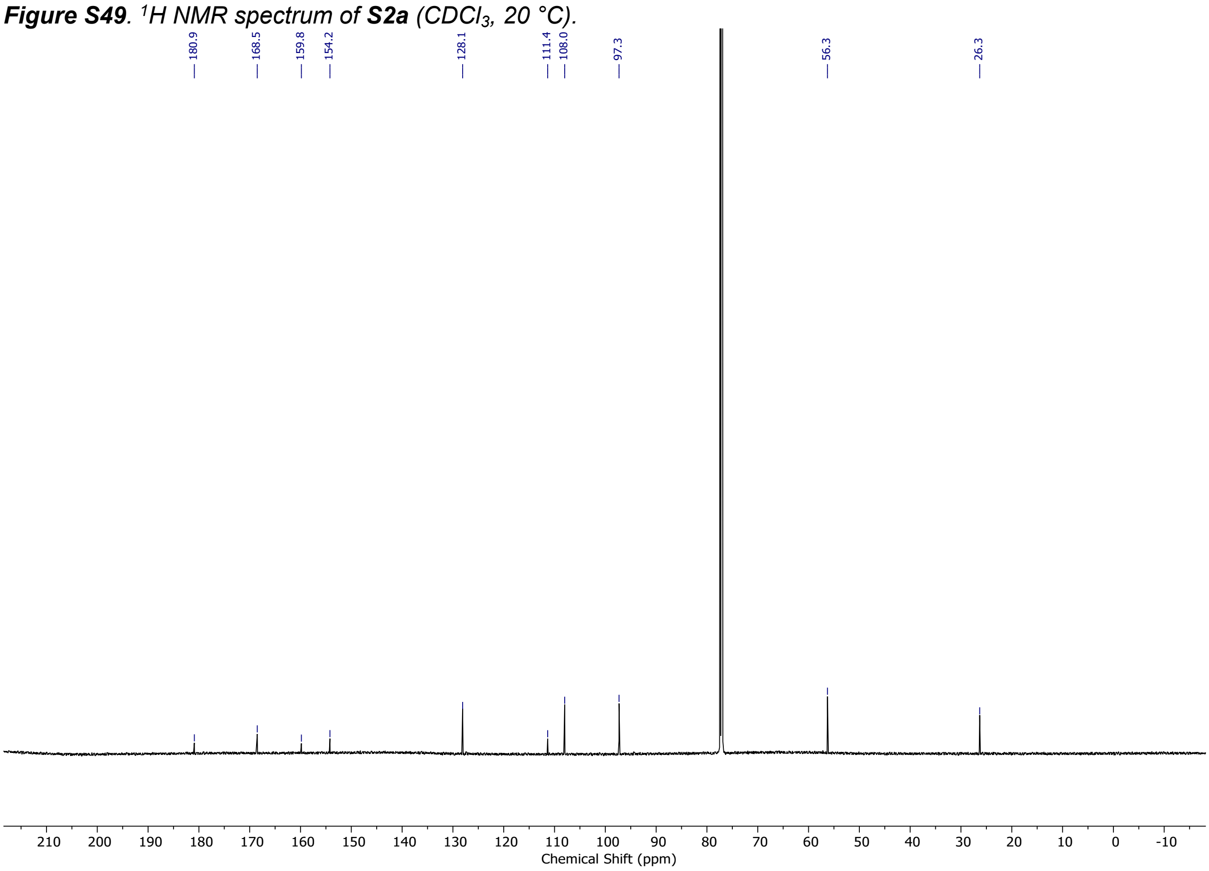

Figure S50. [13]*C NMR spectrum of **S2a** (CDCl$_3$, 20 °C).*

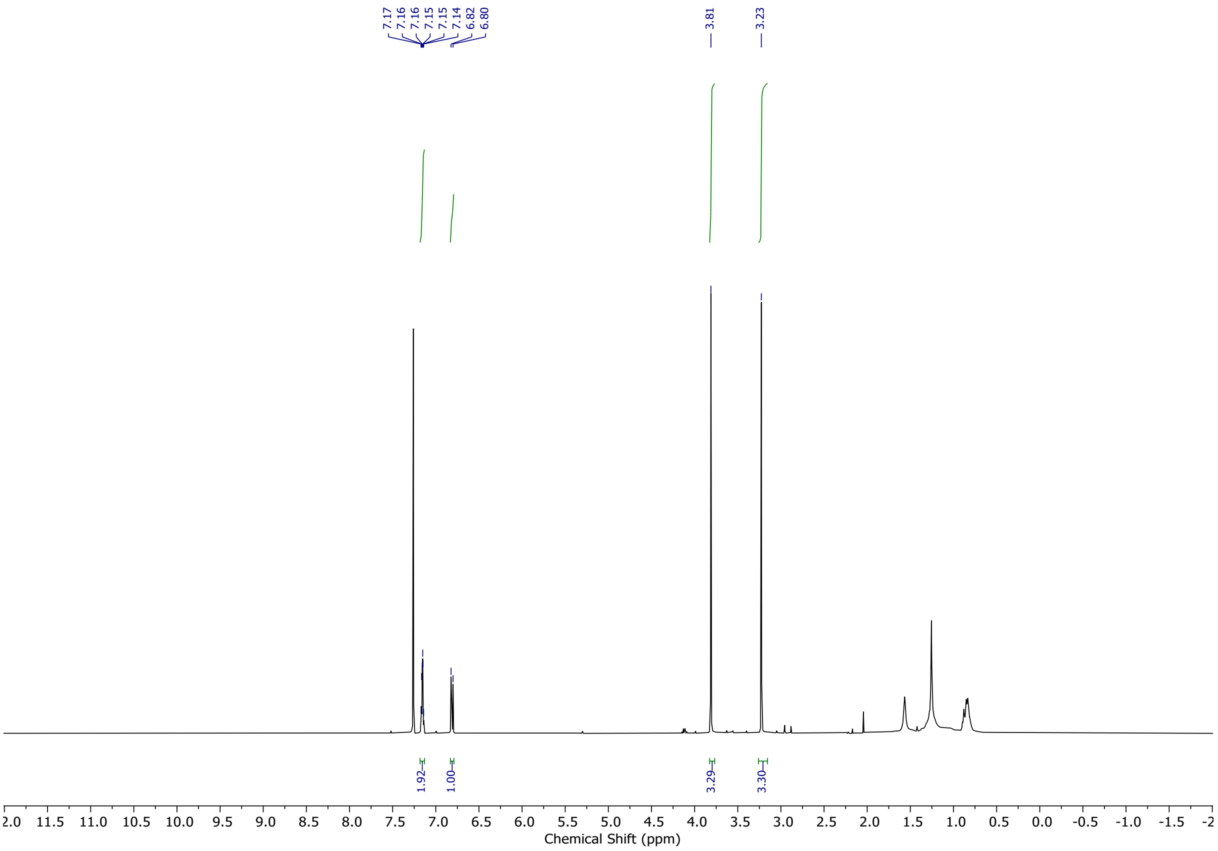

Figure S51. *¹H NMR spectrum of **S5a** (CDCl$_3$, 20 °C).*

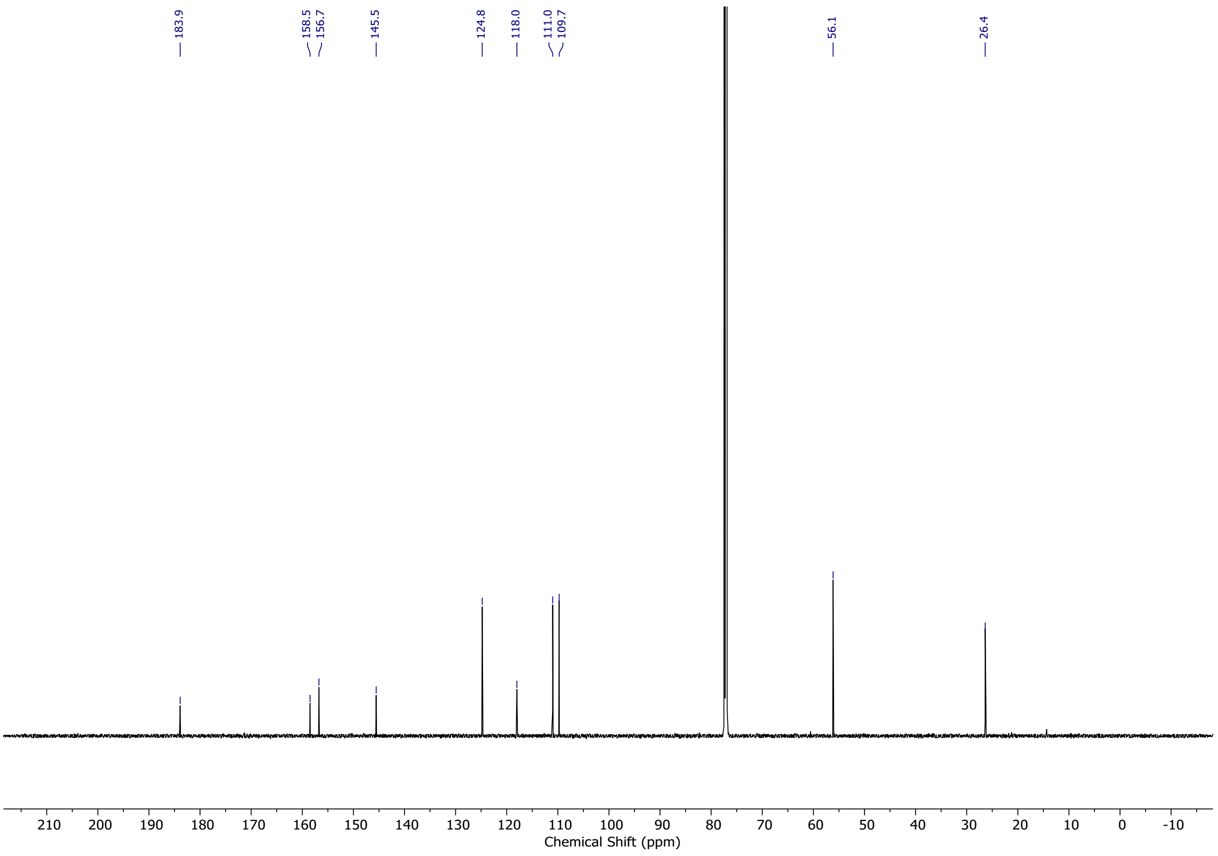

Figure S52. *¹³C NMR spectrum of **S5a** (CDCl$_3$, 20 °C).*

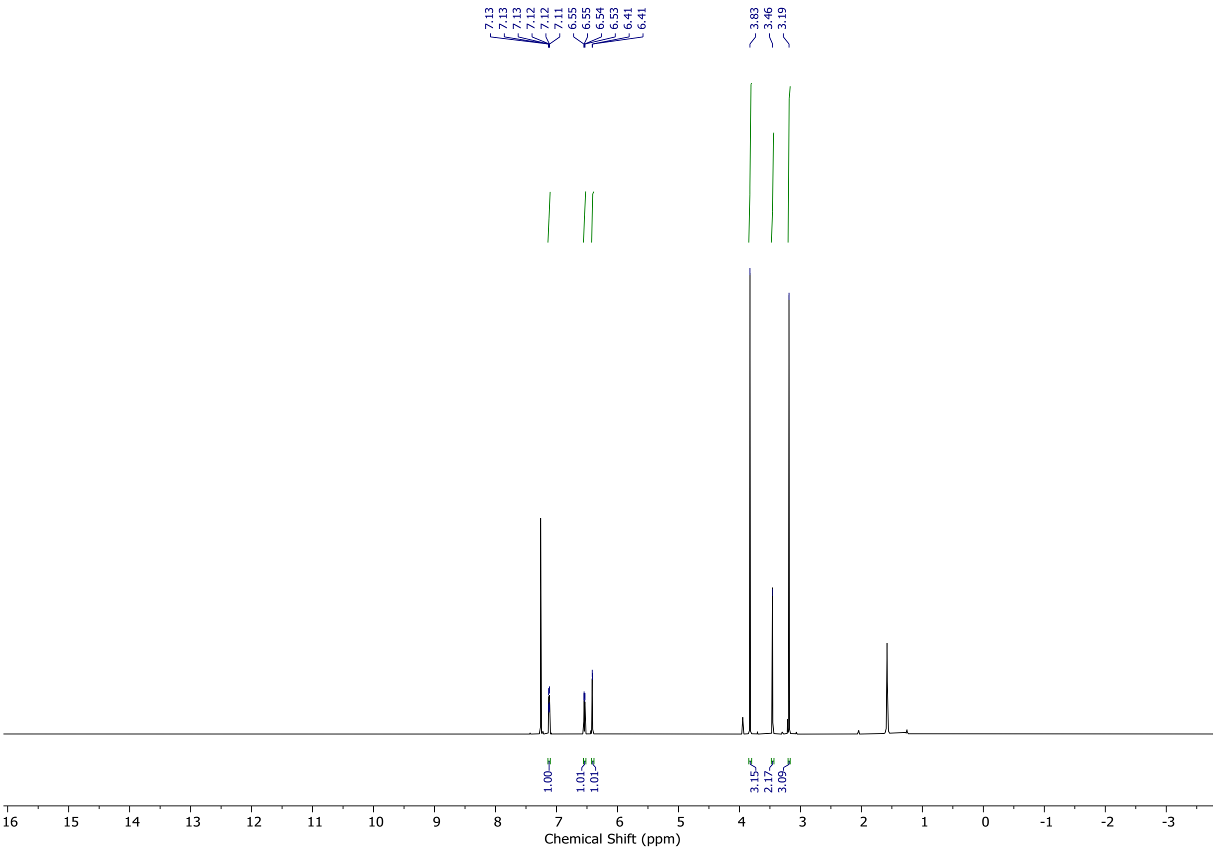

Figure S53. *^{1}H NMR spectrum of **S2** ($CDCl_3$, 20 °C).*

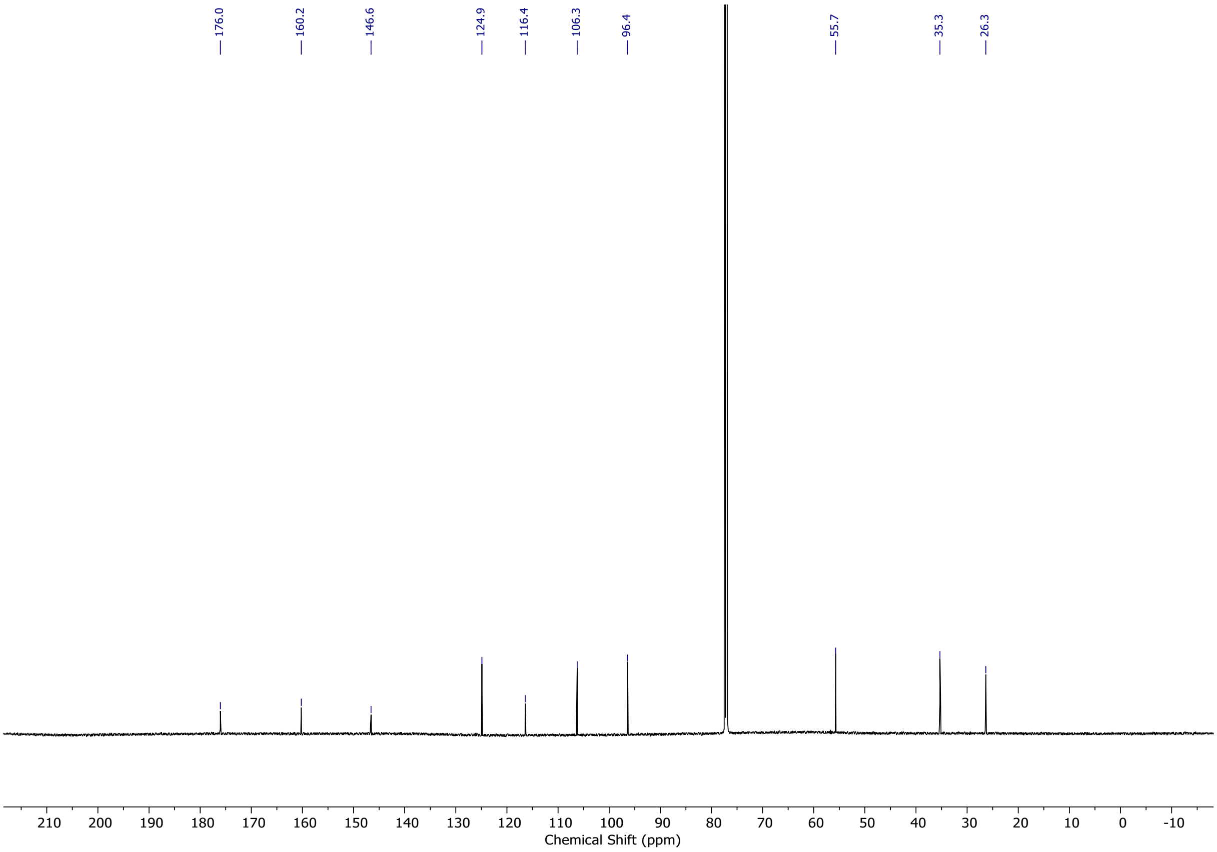

Figure S54. *^{13}C NMR spectrum of **S2** ($CDCl_3$, 20 °C).*

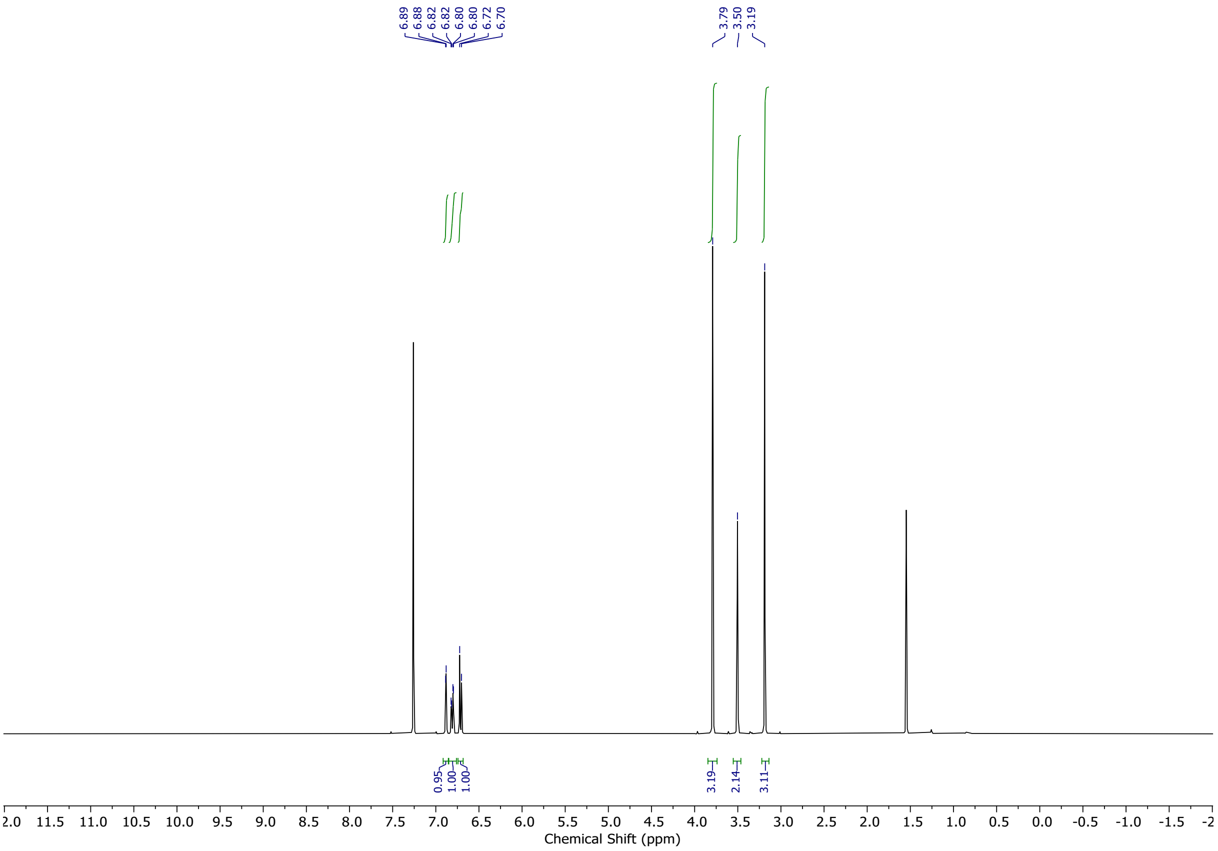

Figure S55. *^{1}H NMR spectrum of **S5** ($CDCl_3$, 20 °C).*

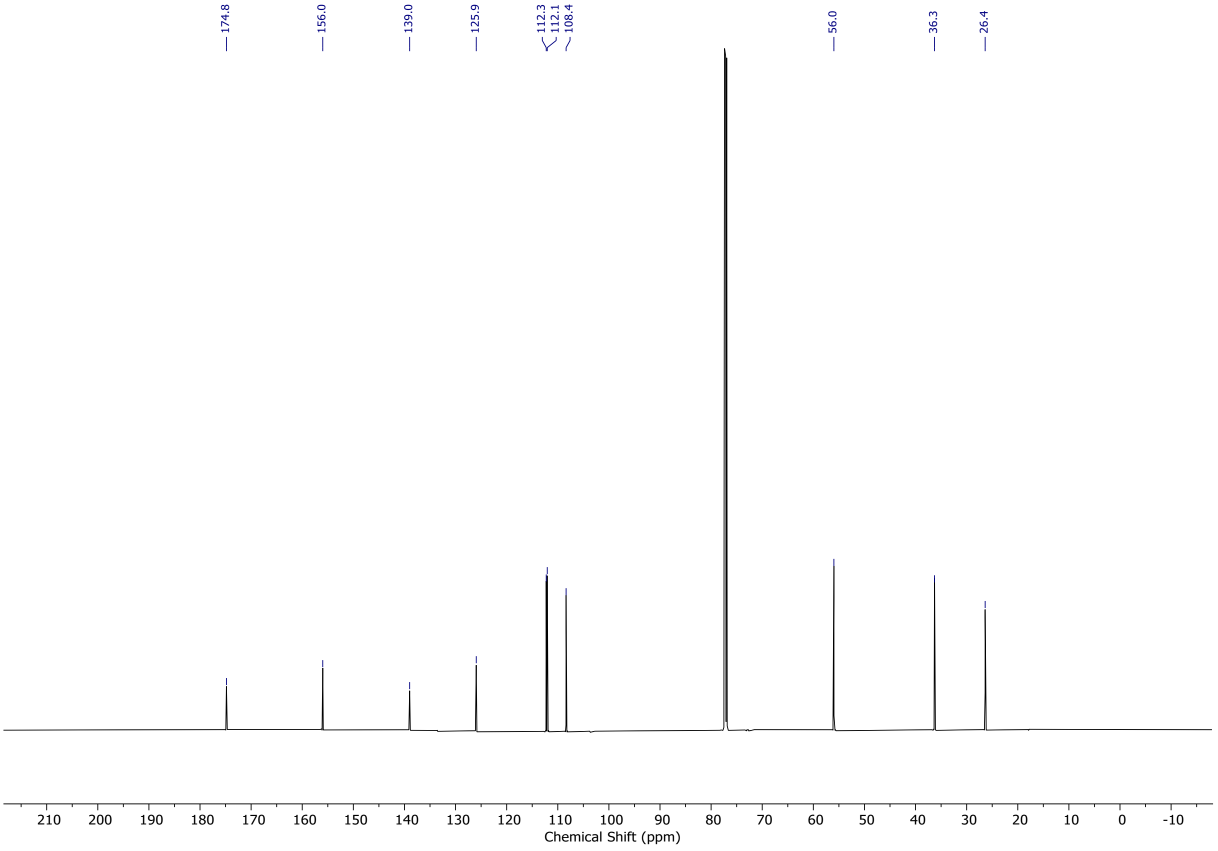

Figure S56. *^{13}C NMR spectrum of **S5** ($CDCl_3$, 20 °C).*

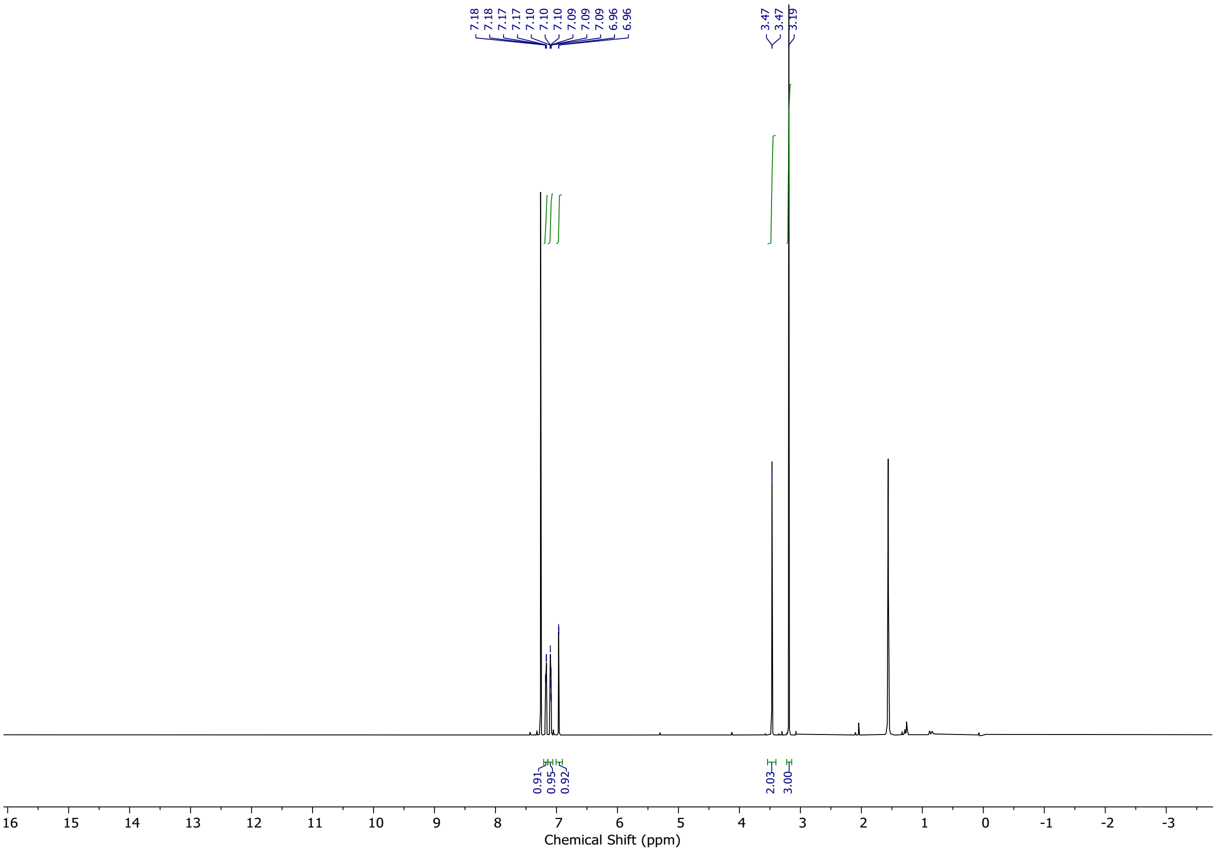

Figure S57. *¹H NMR spectrum of **S3** ($CDCl_3$, 20 °C).*

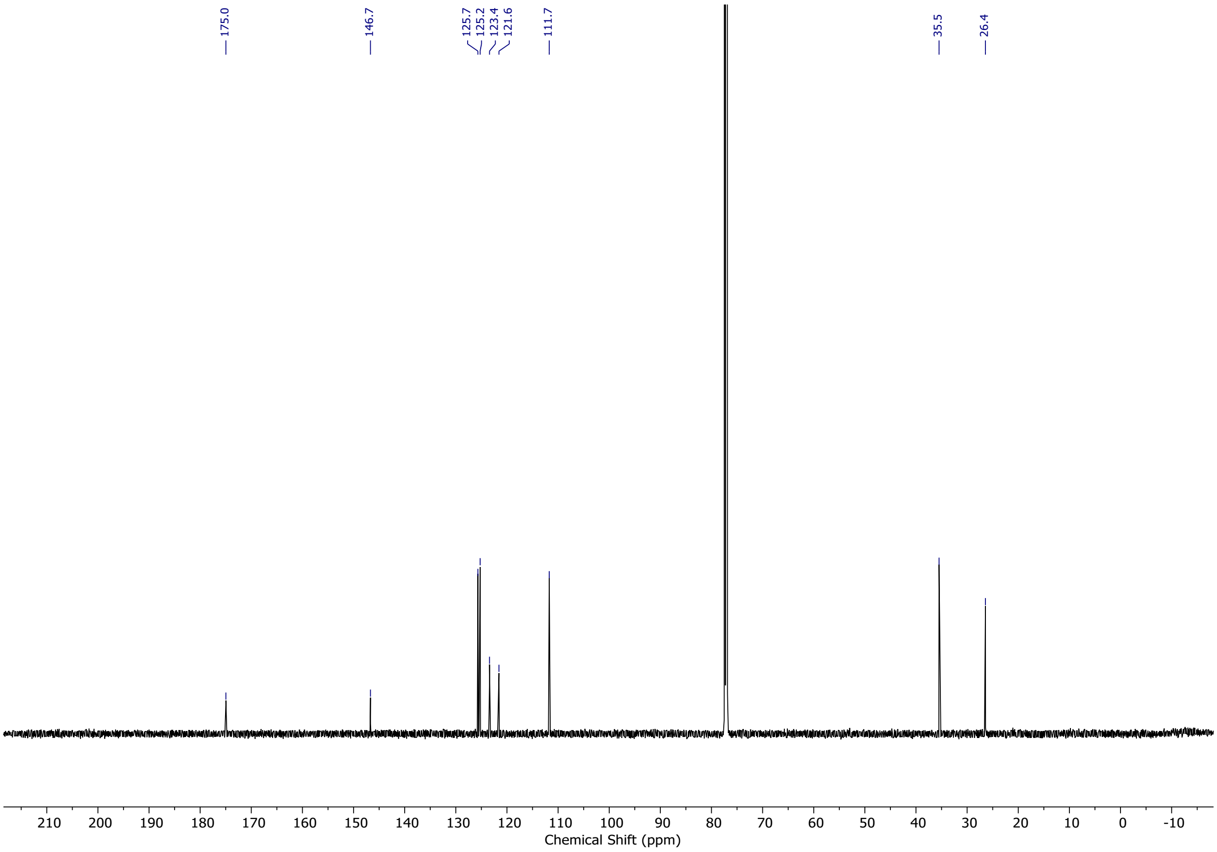

Figure S58. *¹³C NMR spectrum of **S3** ($CDCl_3$, 20 °C).*

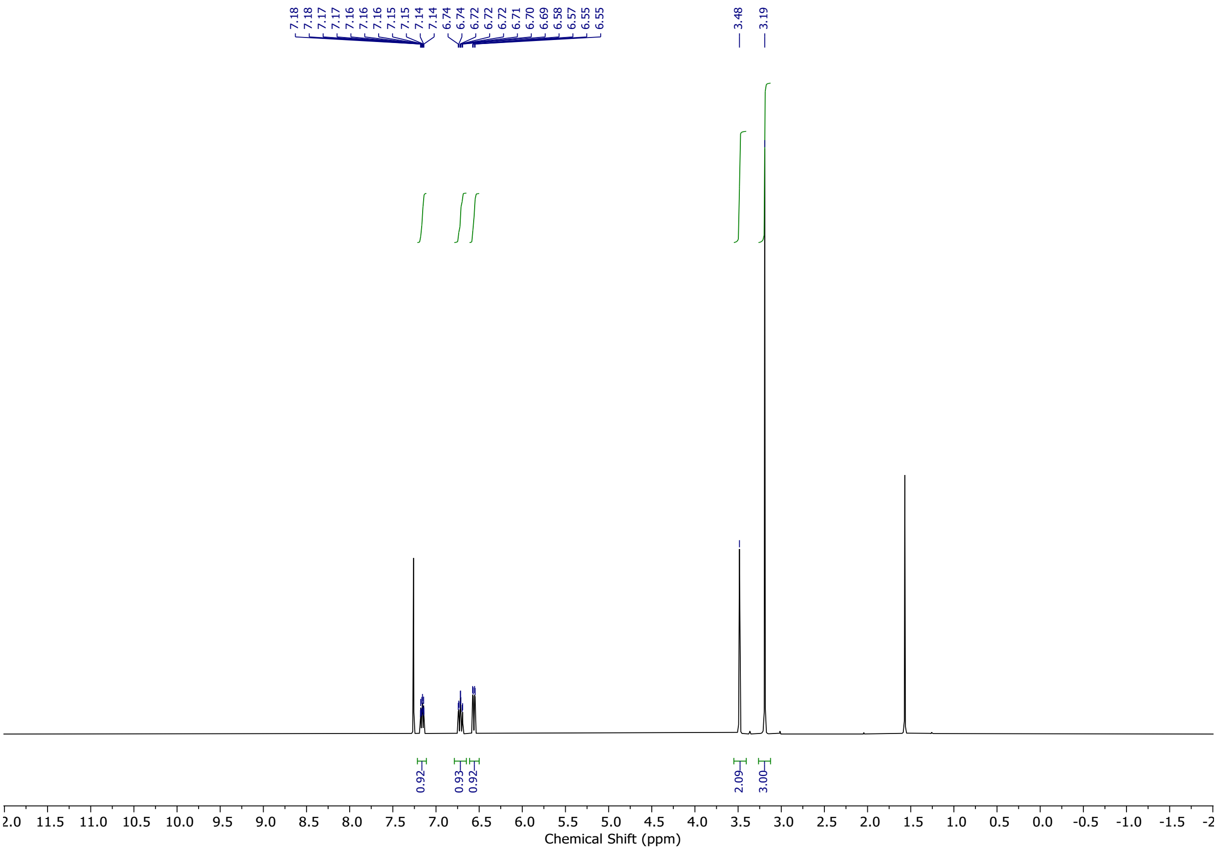

Figure S59. [1]*H NMR spectrum of* ***S4*** *(CDCl$_3$, 20 °C).*

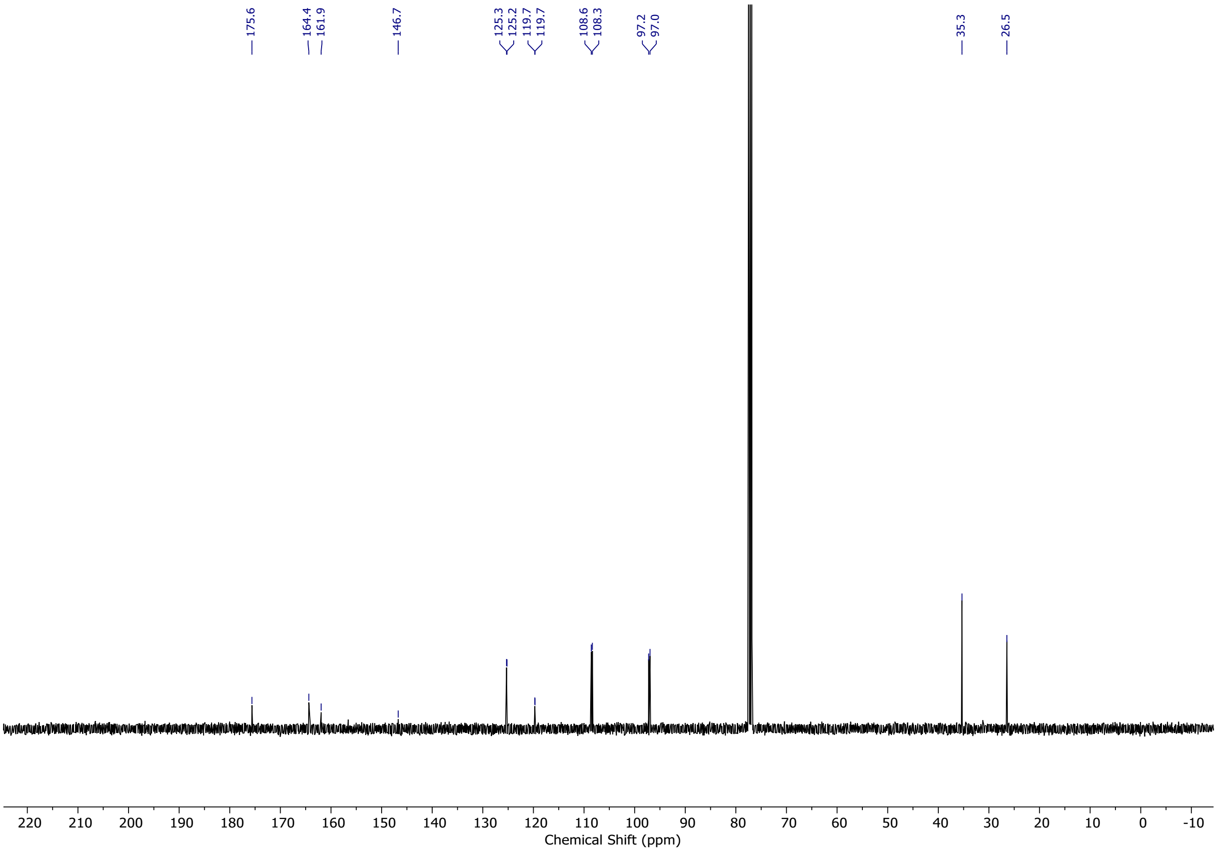

Figure S60. [13]*C NMR spectrum of* ***S4*** *(CDCl$_3$, 20 °C).*

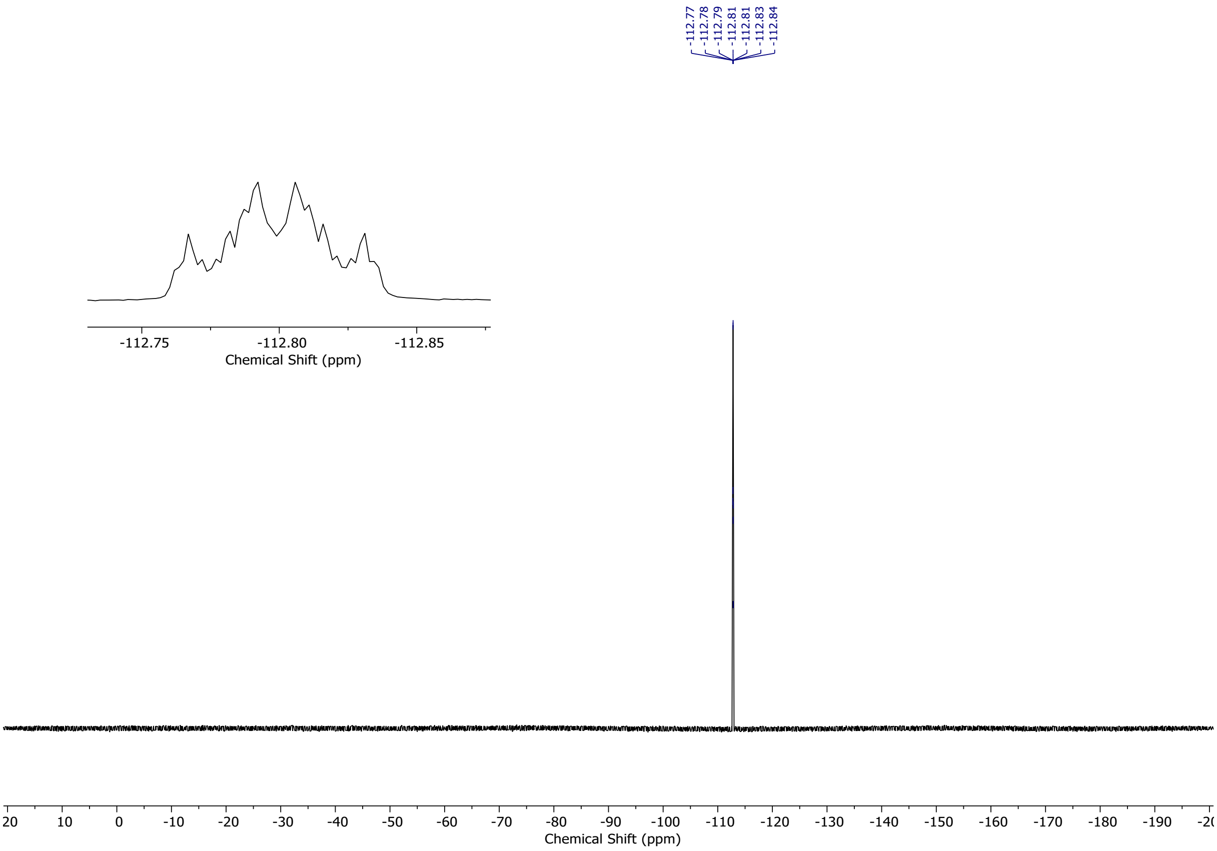

Figure S61. *^{19}F NMR spectrum of **S4** ($CDCl_3$, 20 °C).*

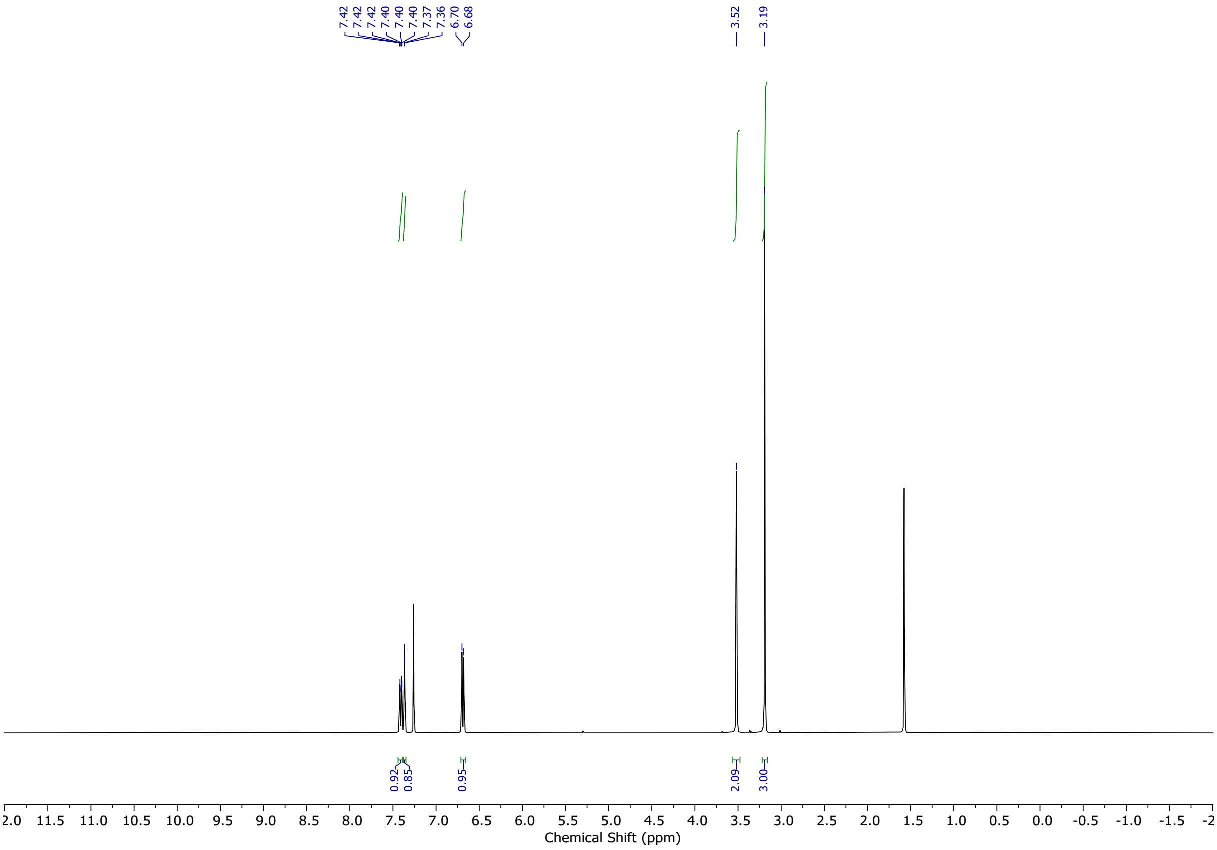

Figure S62. *^{1}H NMR spectrum of **S6** ($CDCl_3$, 20 °C).*

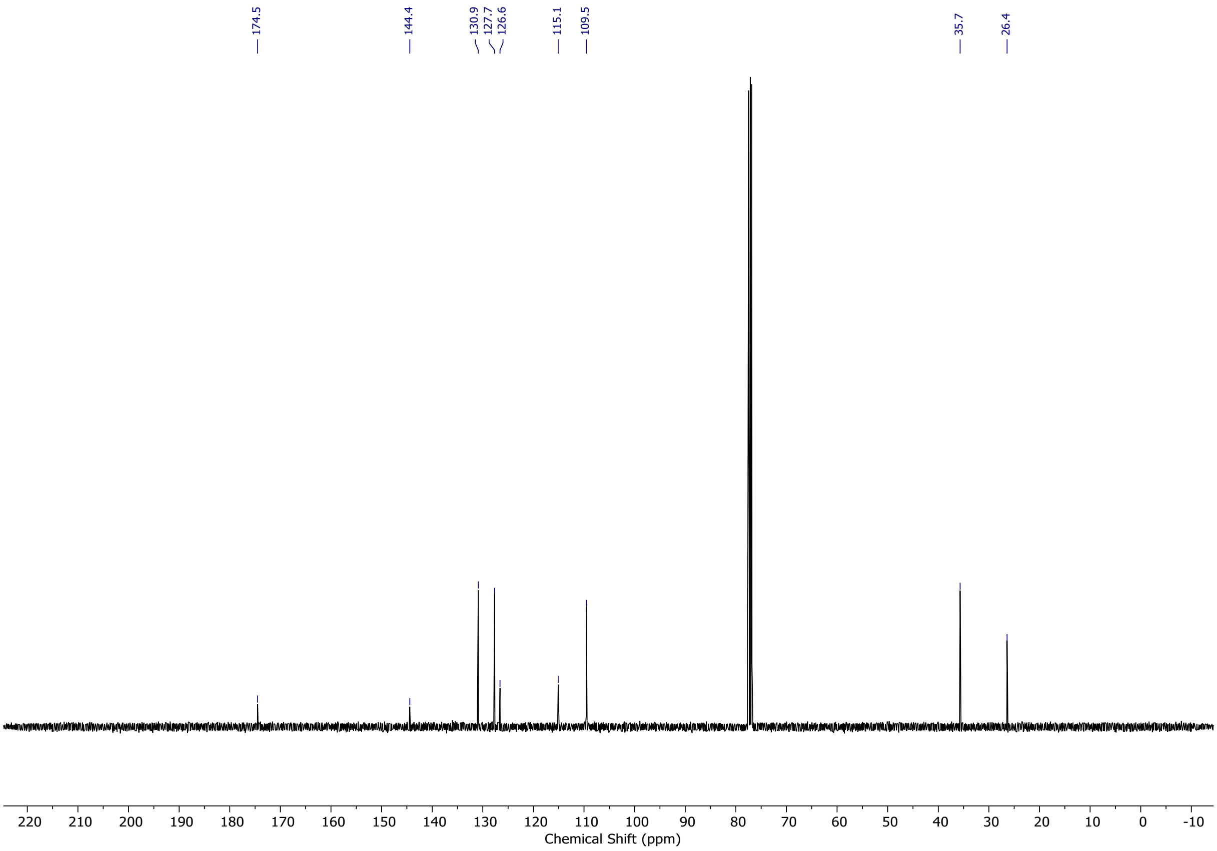

Figure S63. *^{13}C NMR spectrum of **S6** ($CDCl_3$, 20 °C).*

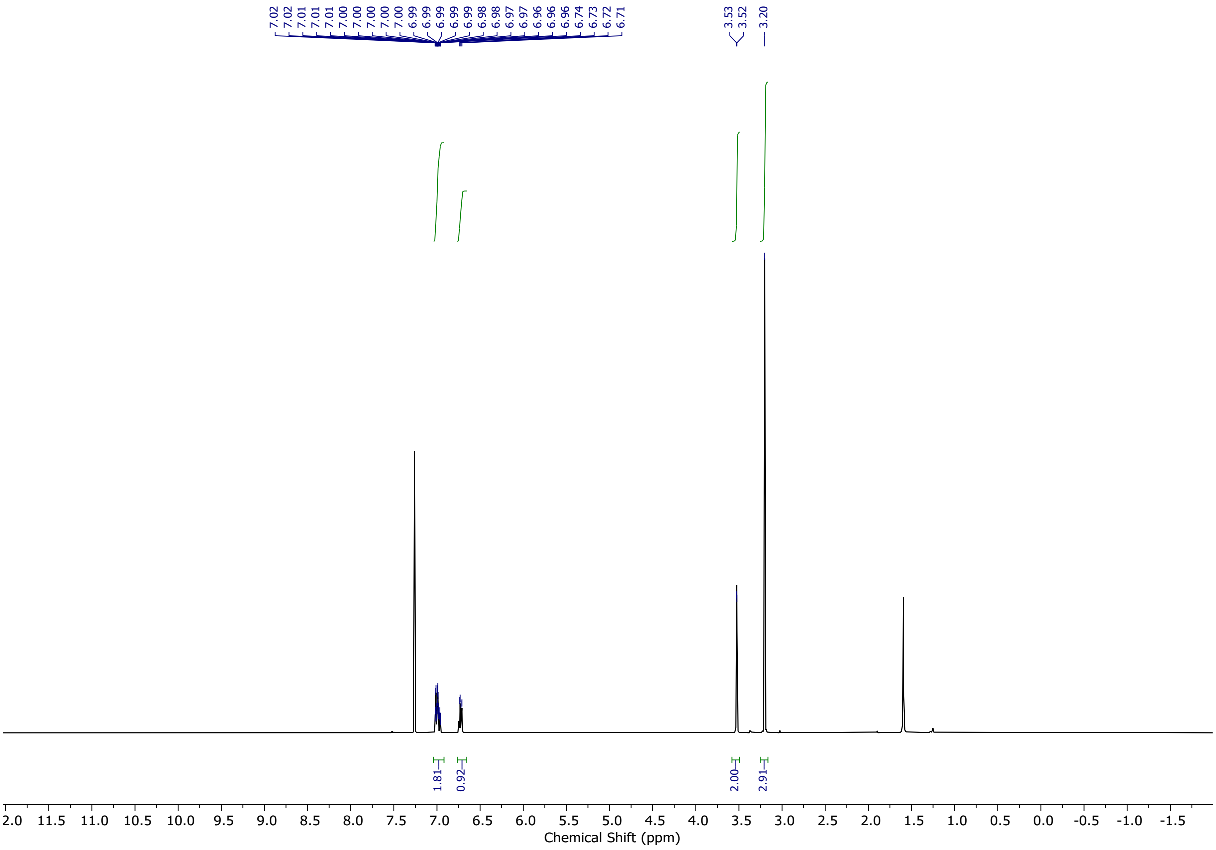

Figure S64. *^{1}H NMR spectrum of **S7** ($CDCl_3$, 20 °C).*

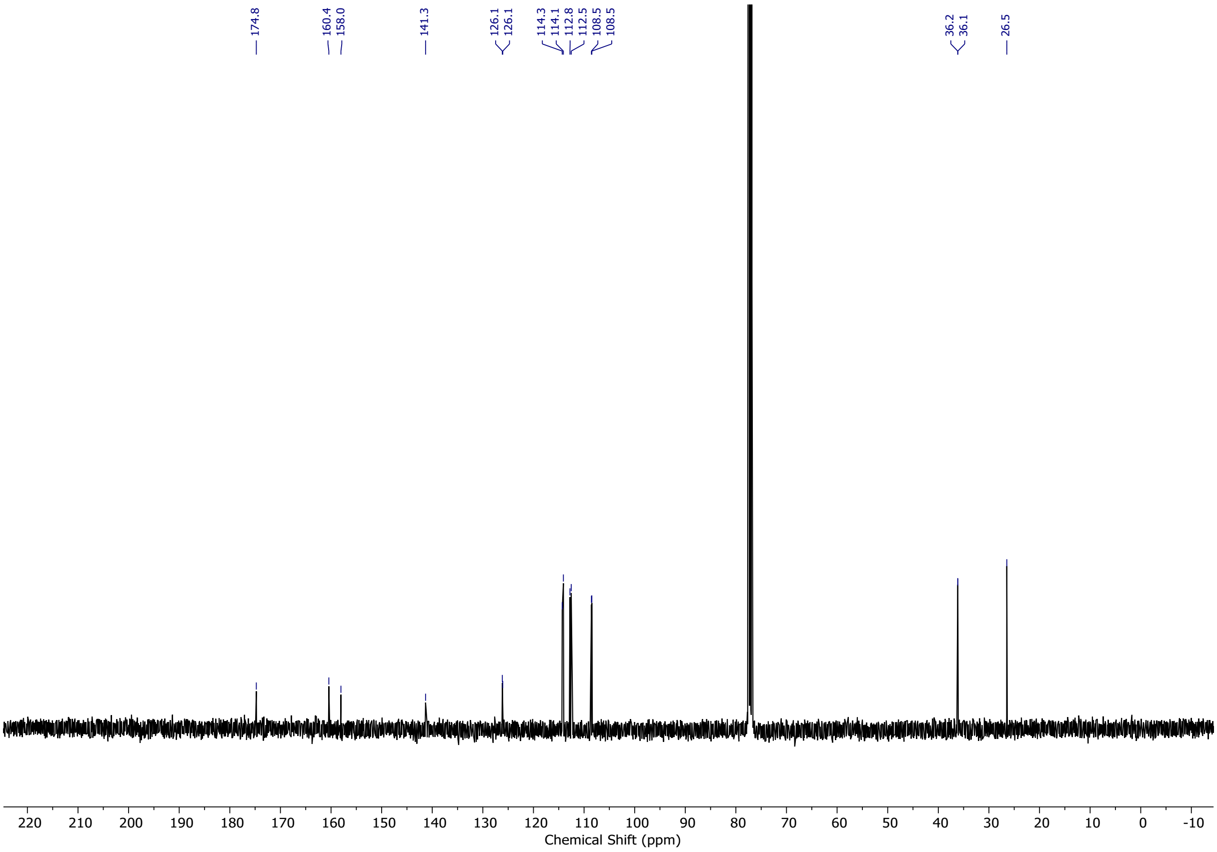

Figure S65. ^{13}C *NMR spectrum of* ***S7*** *($CDCl_3$, 20 °C).*

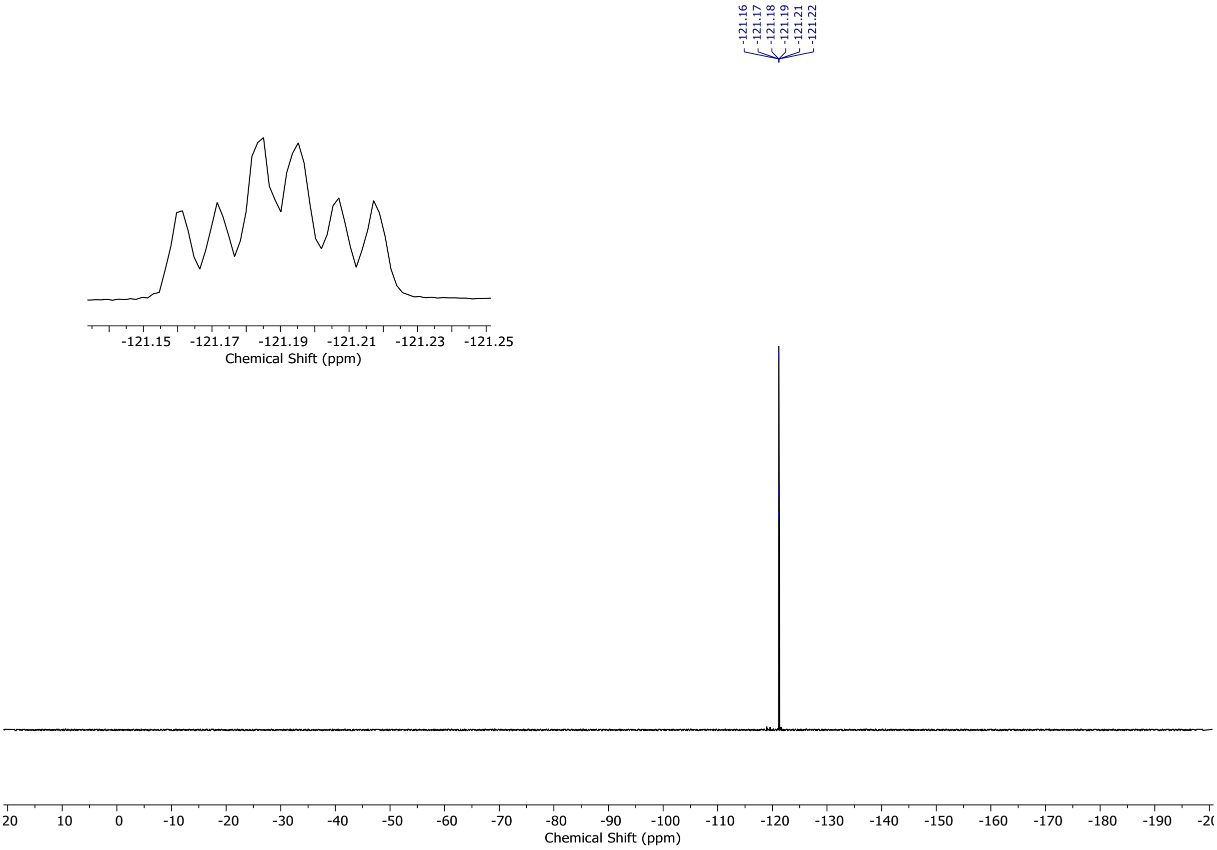

Figure S66. ^{19}F *NMR spectrum of* ***S7*** *($CDCl_3$, 20 °C).*

Motor 2

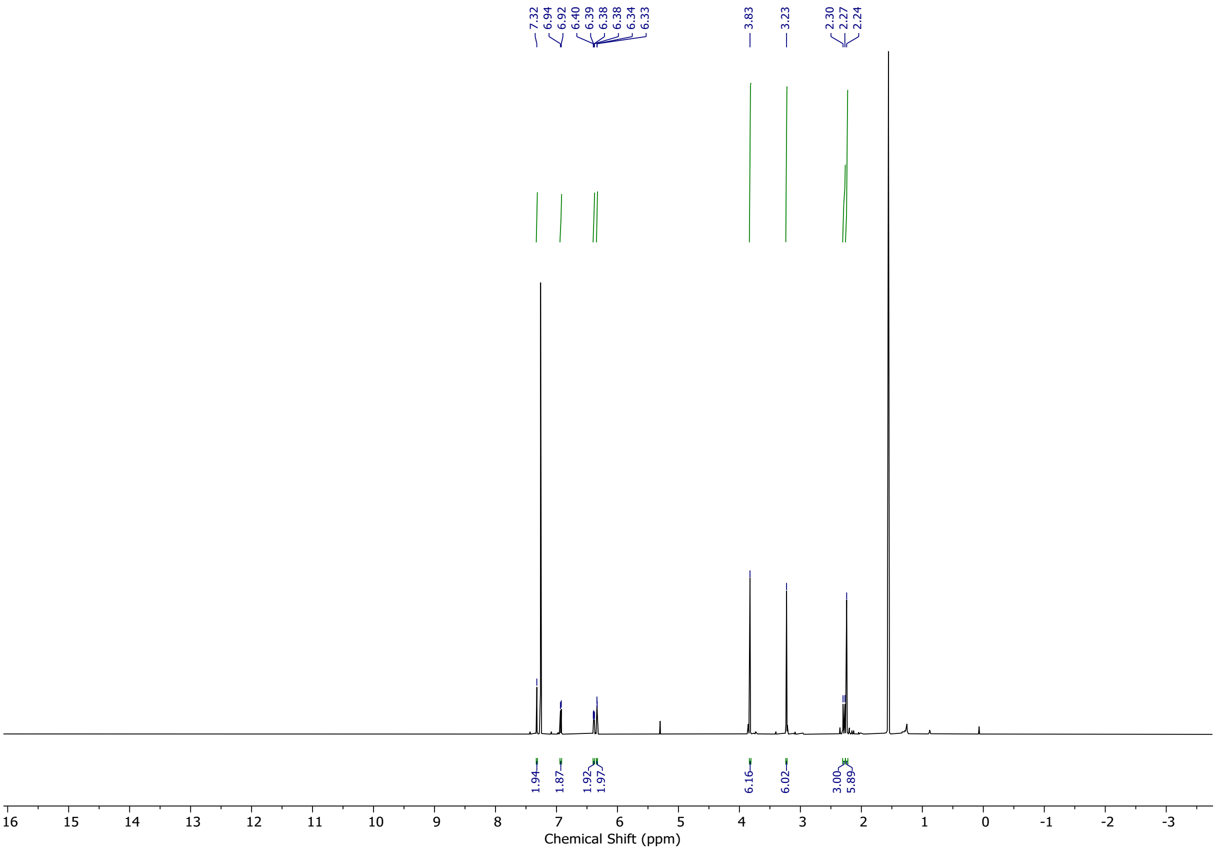

***Figure S67**. ^{1}H NMR spectrum of (Z_SZ_S)-**2** ($CDCl_3$, 20 °C).*

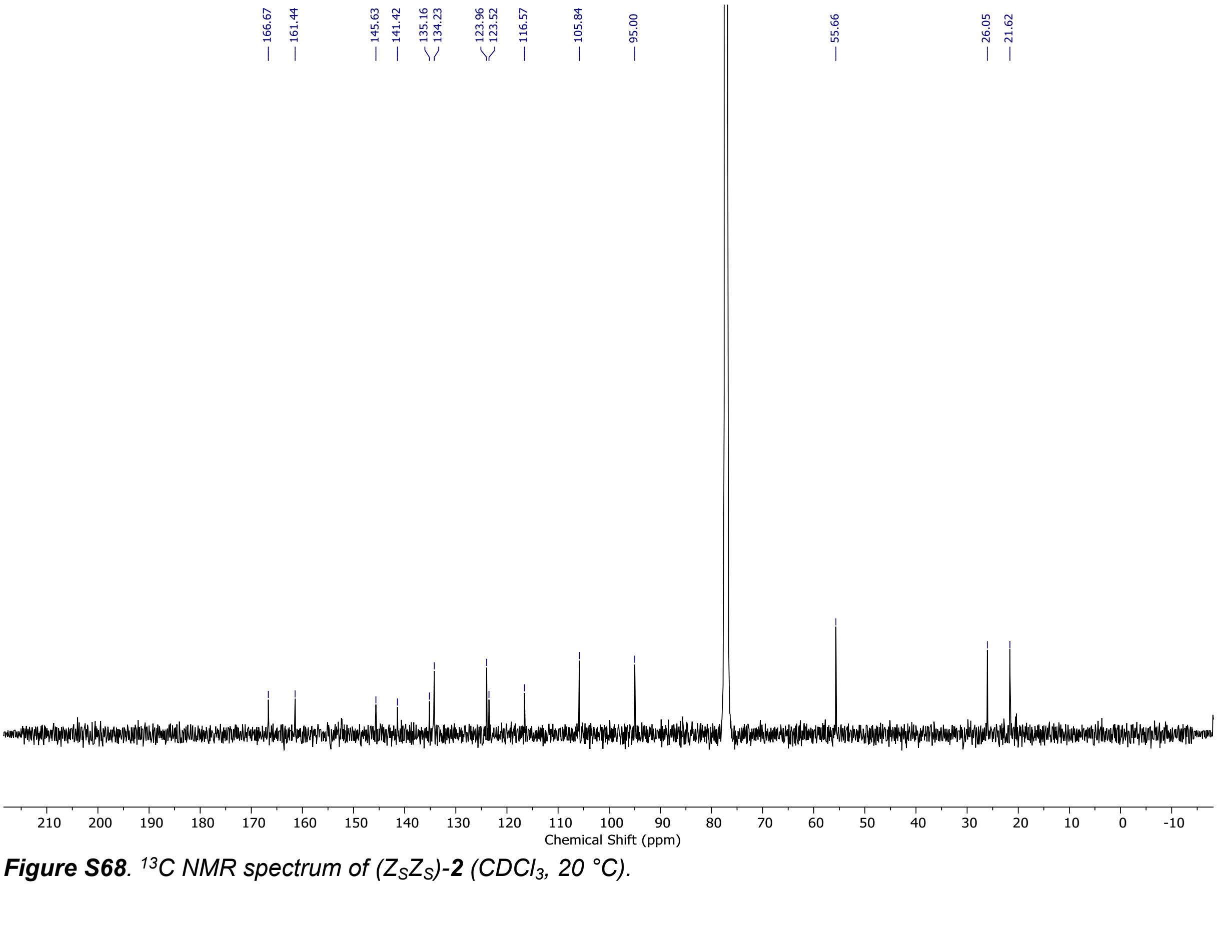

***Figure S68**. ^{13}C NMR spectrum of (Z_SZ_S)-**2** ($CDCl_3$, 20 °C).*

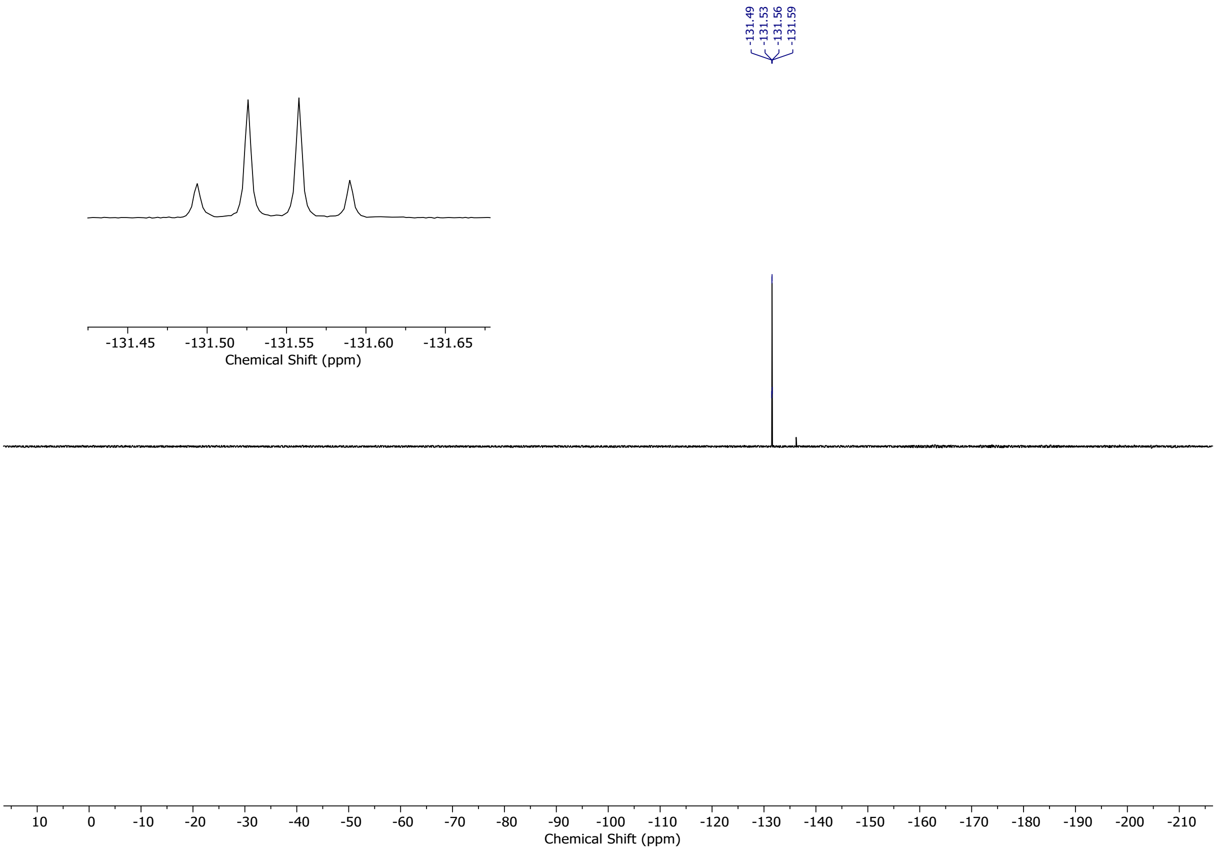

***Figure S69**. ^{19}F NMR spectrum of (Z_SZ_S)-**2** ($CDCl_3$, 20 °C).*

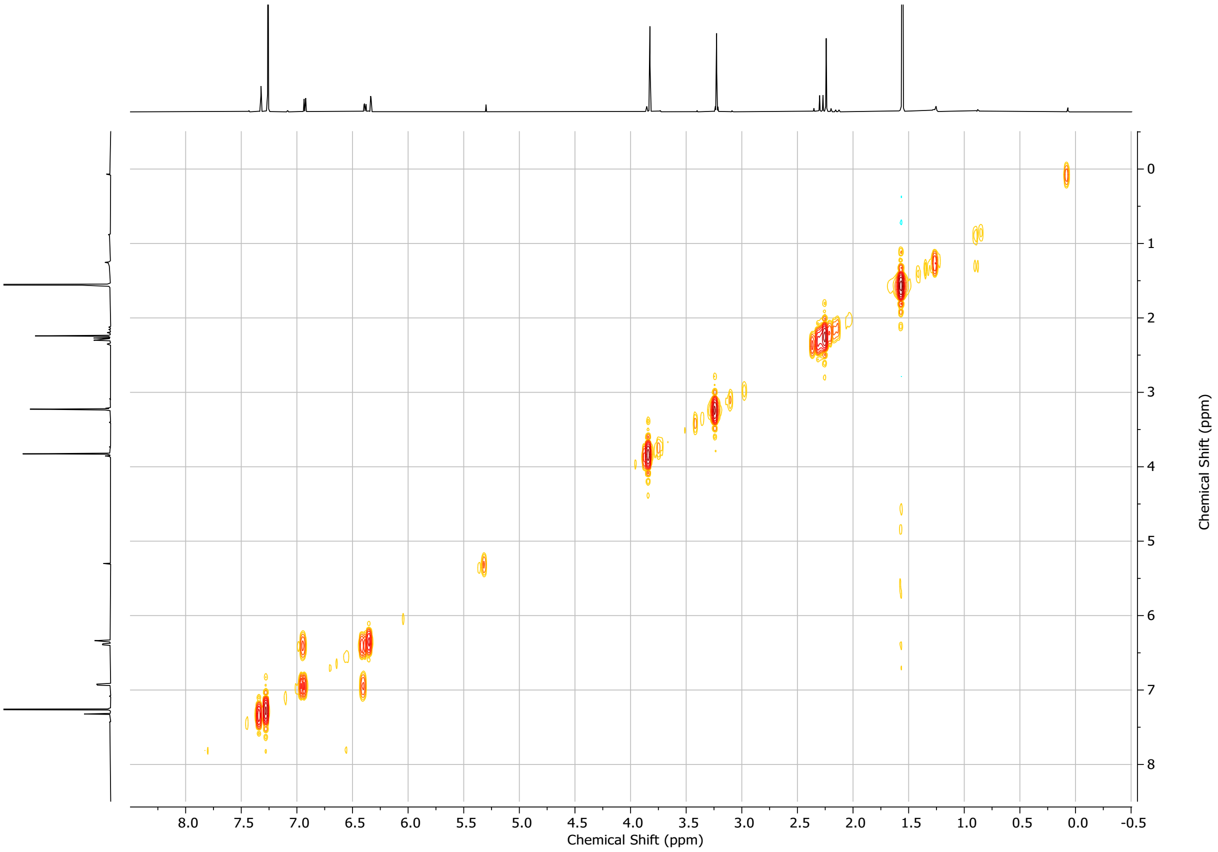

***Figure S70**. COSY NMR spectrum of (Z_SZ_S)-**2** ($CDCl_3$, 20 °C).*

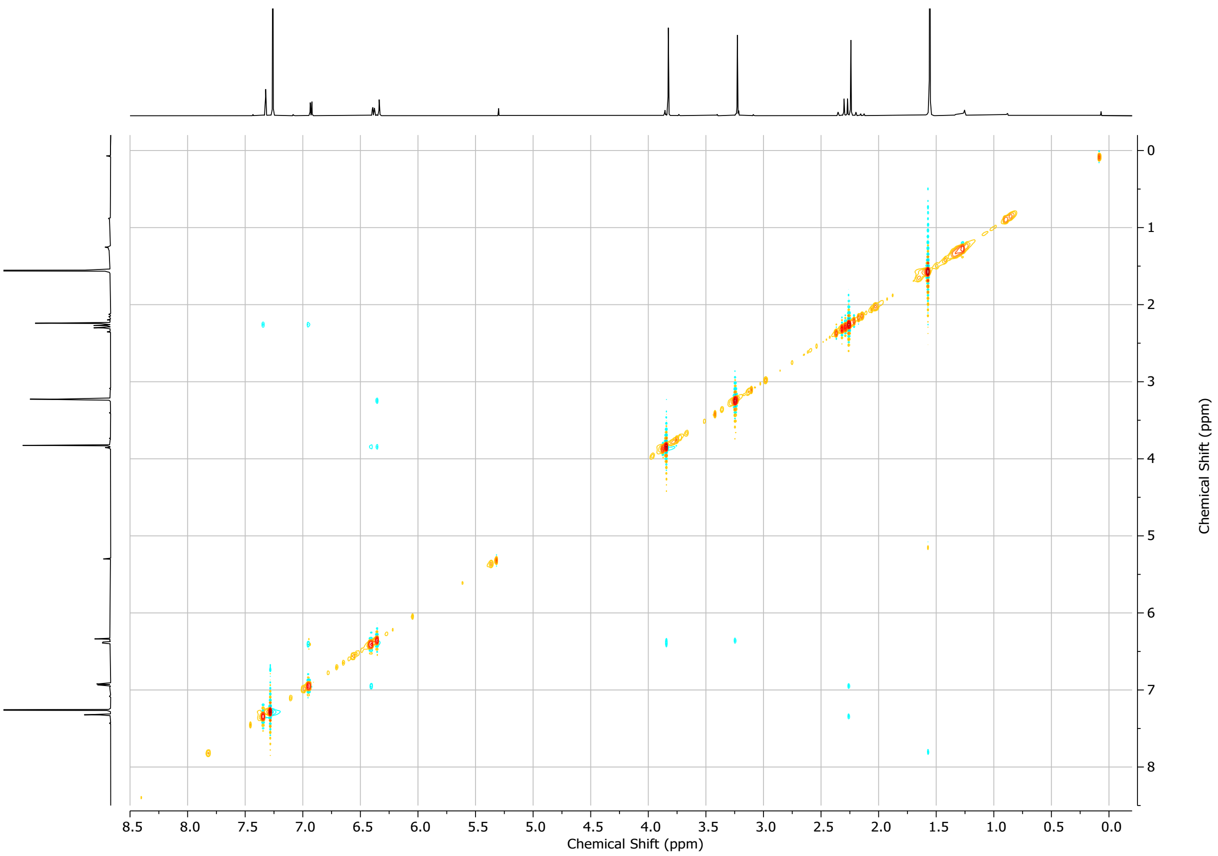

Figure S71. *NOESY NMR spectrum of (*Z_SZ_S*)-**2** (*$CDCl_3$*, 20 °C).*

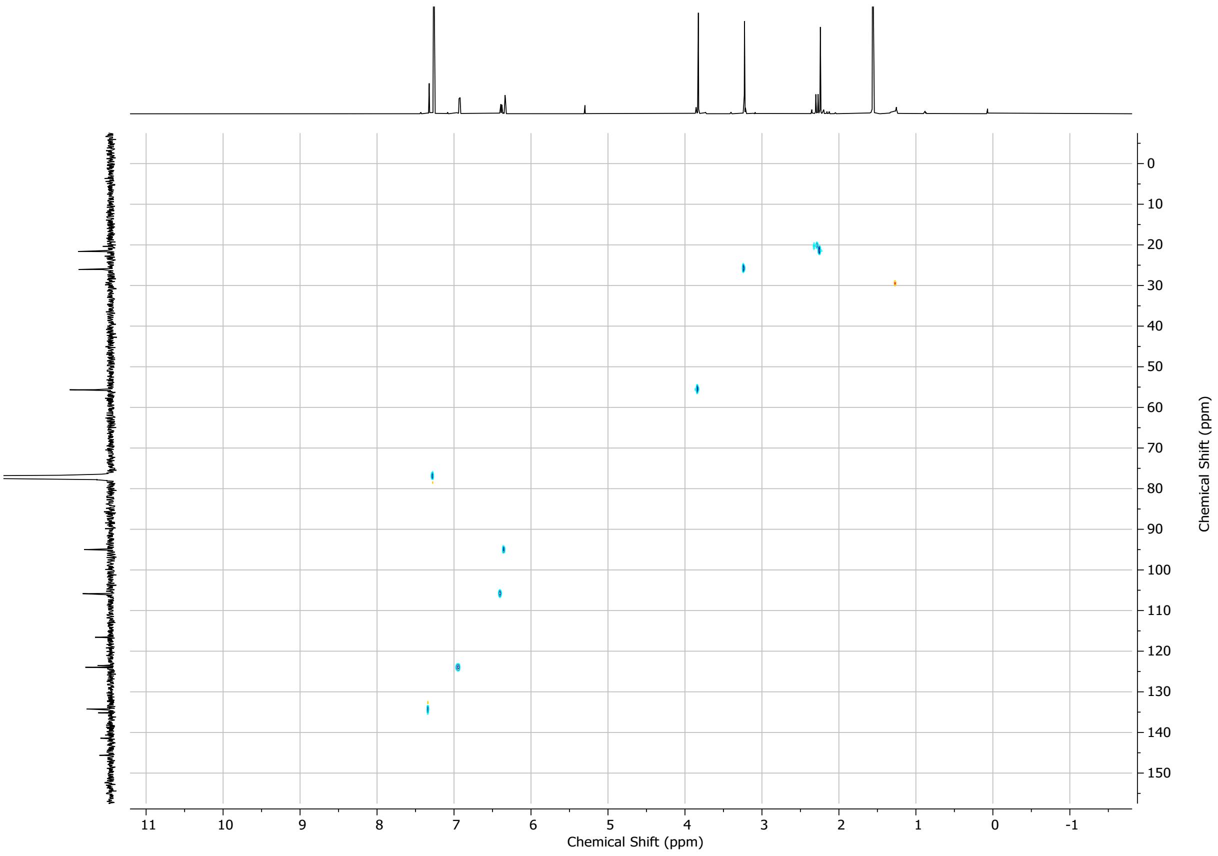

Figure S72. *HSQC NMR spectrum of (*Z_SZ_S*)-**2** (*$CDCl_3$*, 20 °C).*

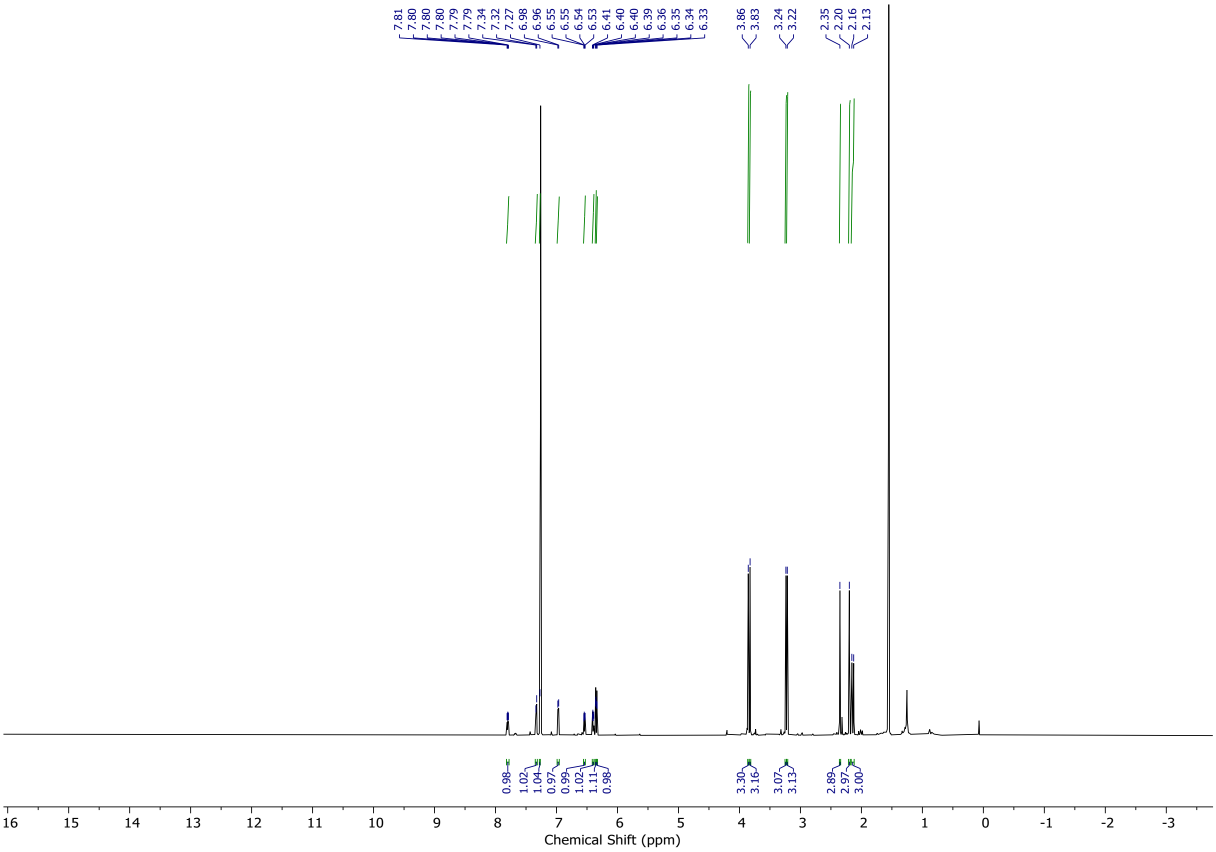

Figure S73. *^{1}H NMR spectrum of ($E_S$$Z_S$)-**2** ($CDCl_3$, 20 °C).*

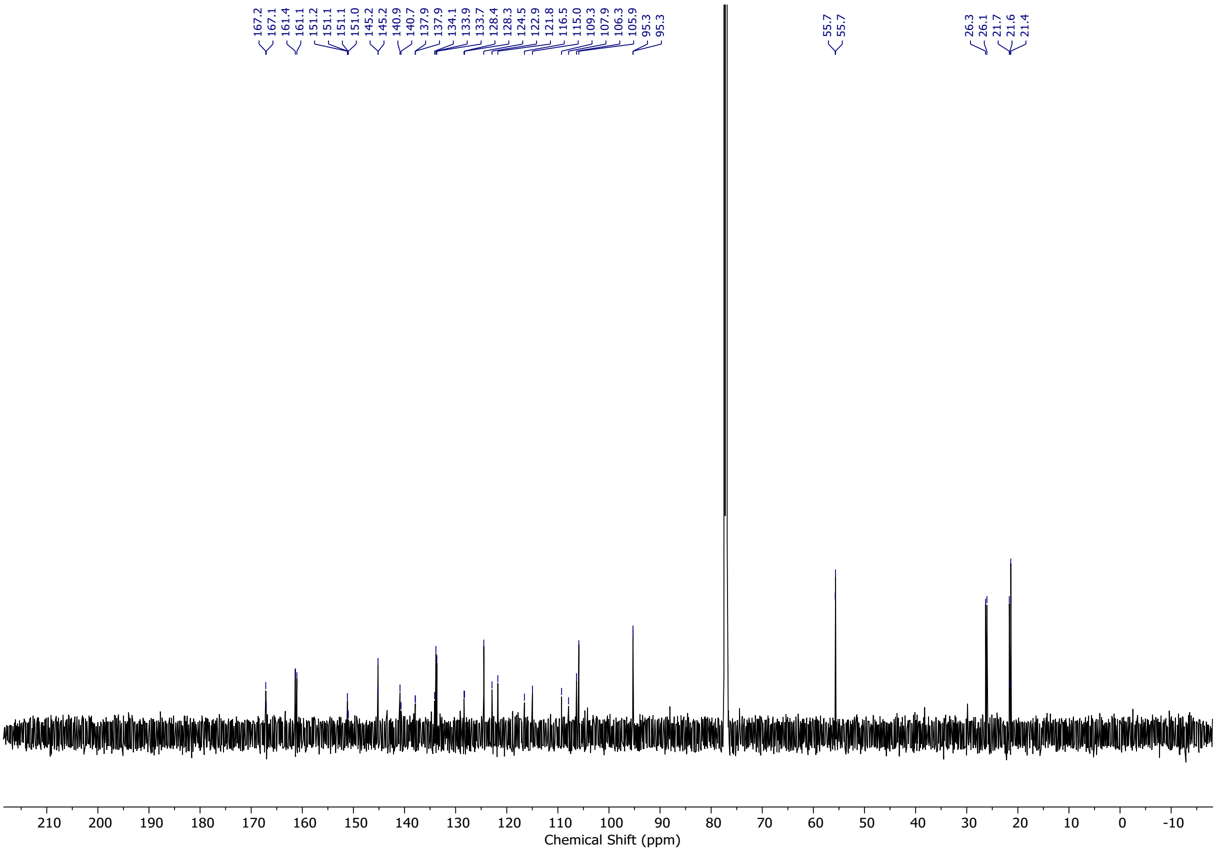

Figure S74. *^{13}C NMR spectrum of ($E_S$$Z_S$)-**2** ($CDCl_3$, 20 °C).*

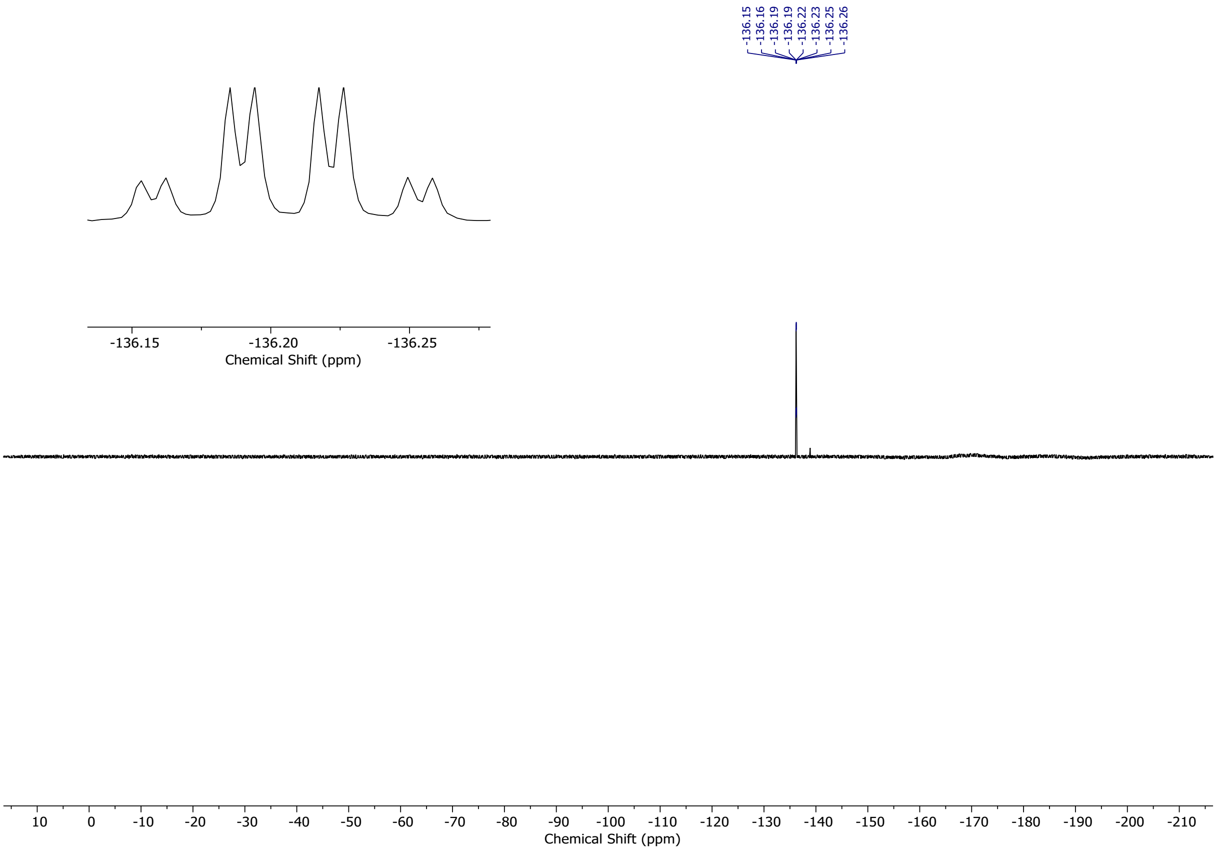

Figure S75. *^{19}F NMR spectrum of (E_SZ_S)-**2** ($CDCl_3$, 20 °C).*

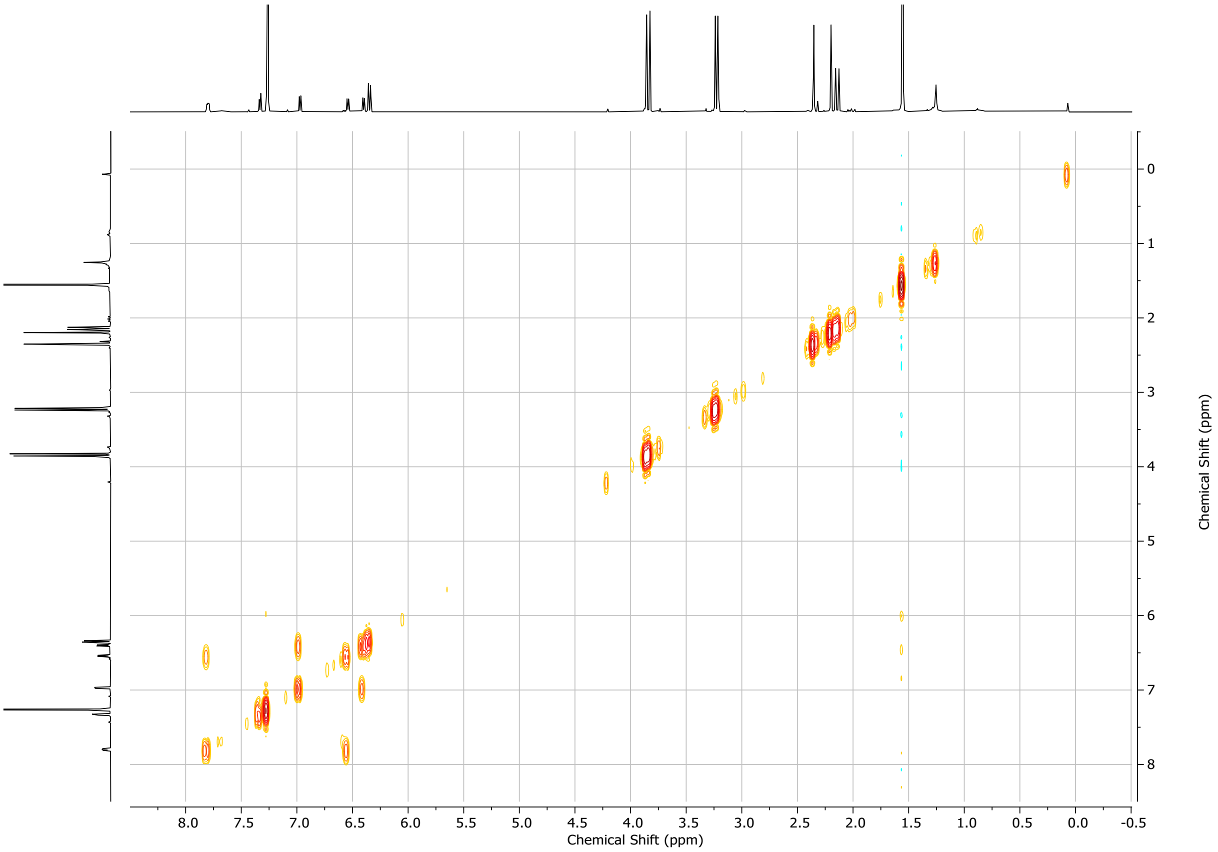

Figure S76. *COSY NMR spectrum of (E_SZ_S)-**2** ($CDCl_3$, 20 °C).*

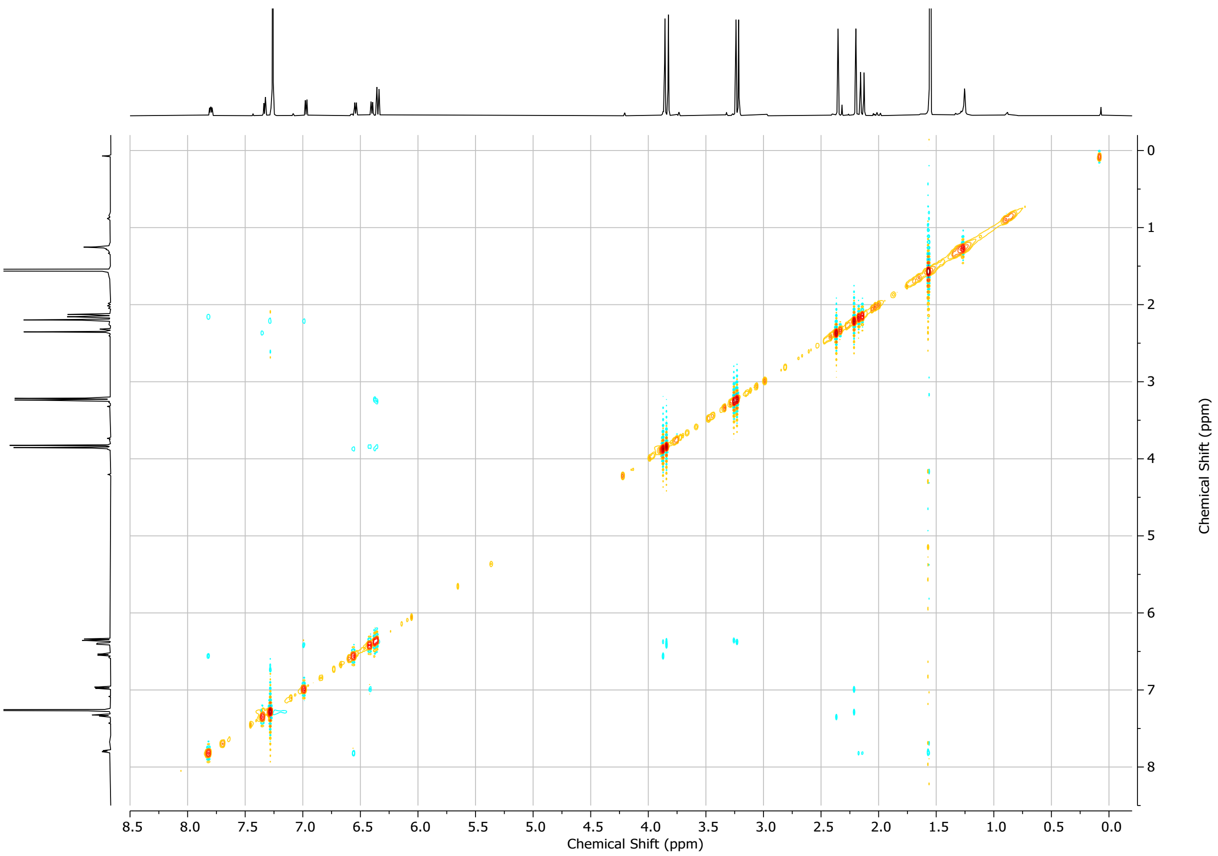

Figure S77. *NOESY NMR spectrum of (E_SZ_S)-**2** (CDCl$_3$, 20 °C).*

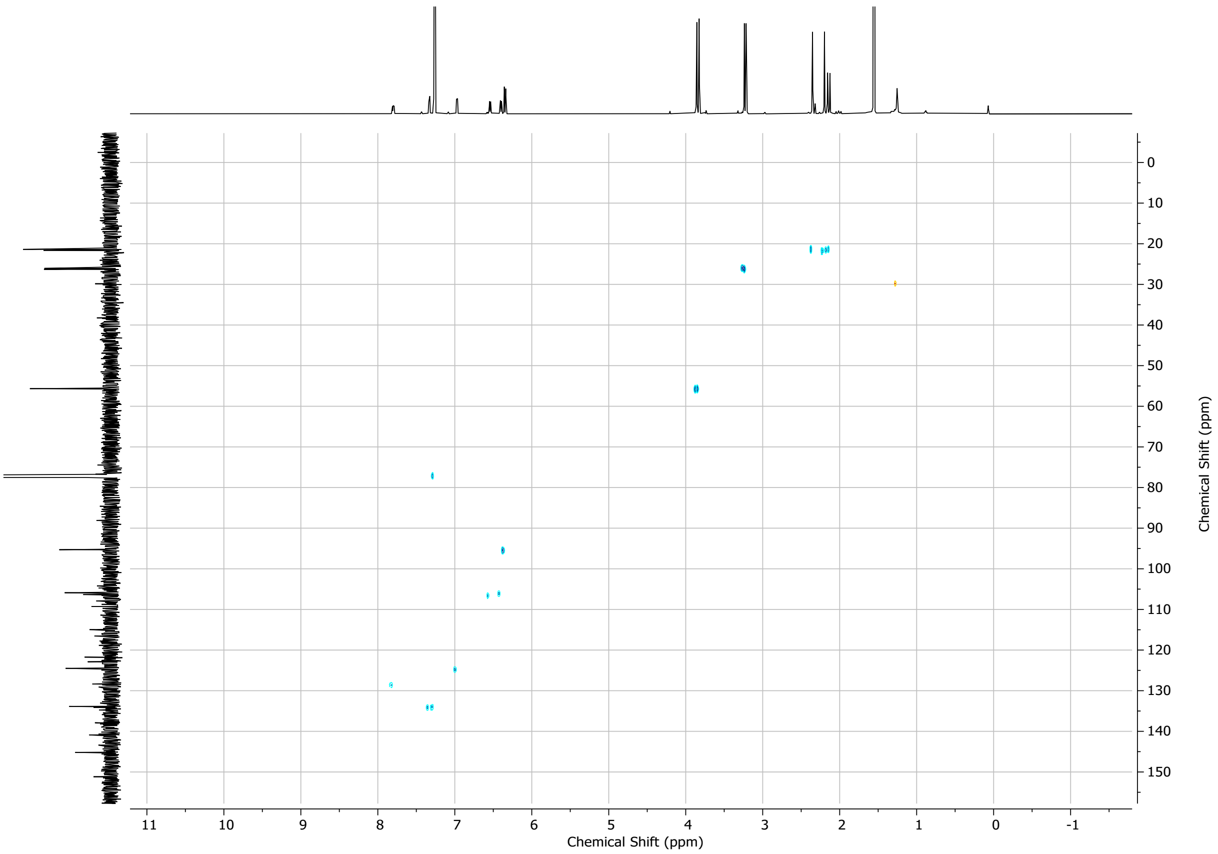

Figure S78. *HSQC NMR spectrum of (E_SZ_S)-**2** (CDCl$_3$, 20 °C).*

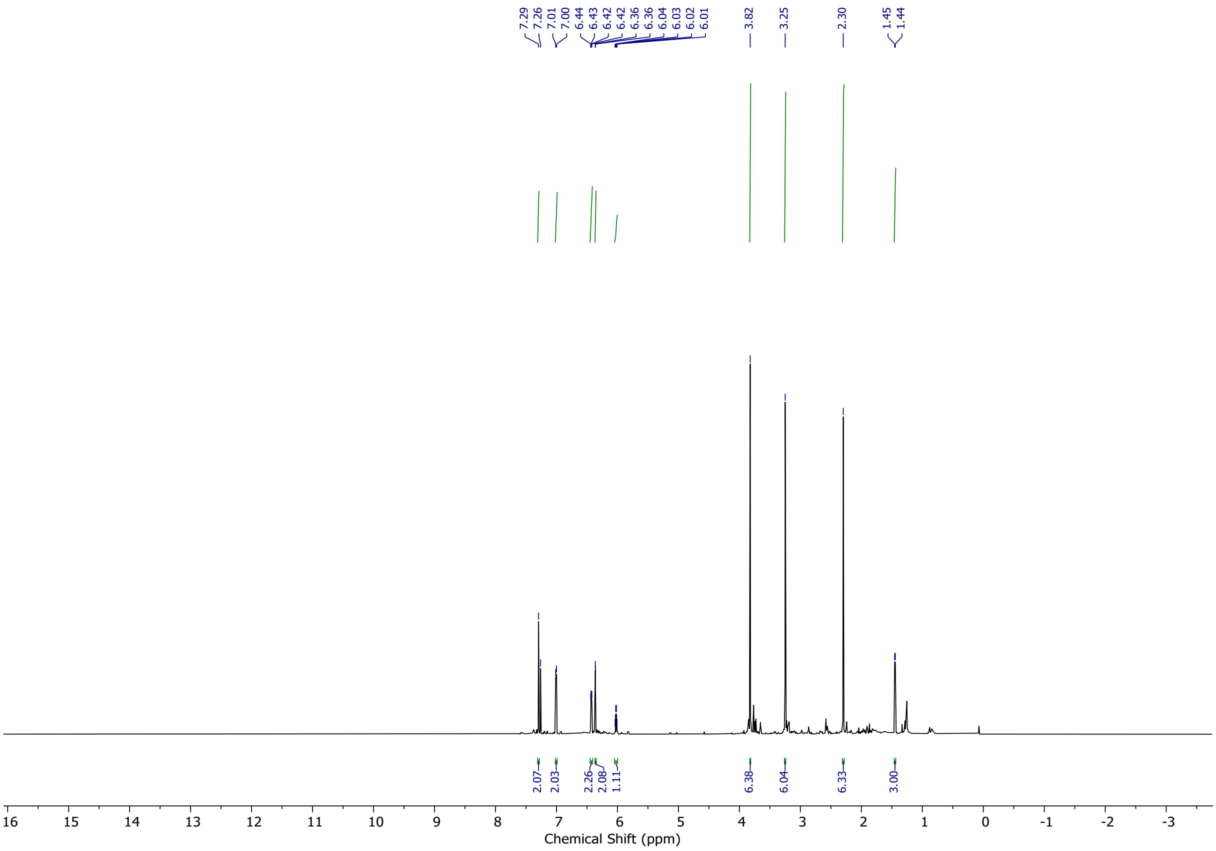

Figure S79. ¹H NMR spectrum of (Z_SZ_S)-**2DeF** ($CDCl_3$, 20 °C).

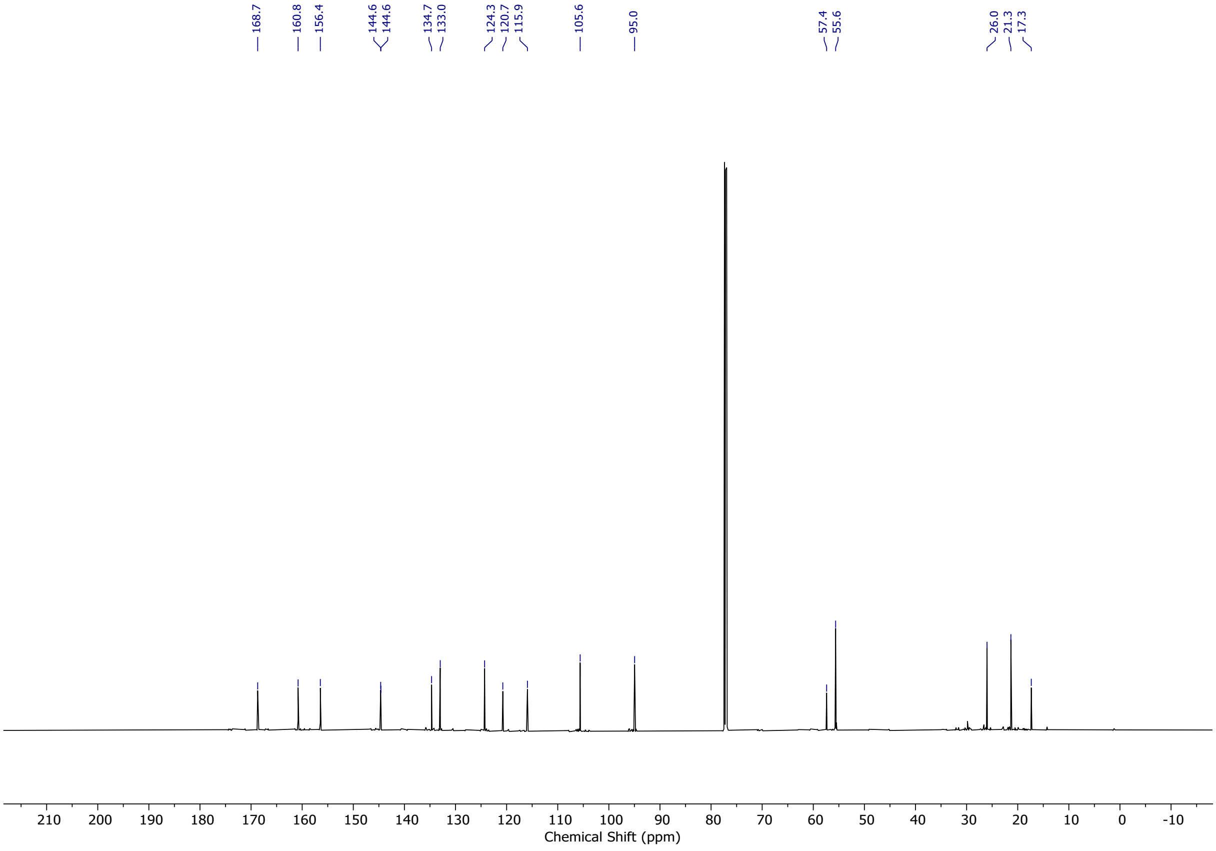

Figure S80. ¹³C NMR spectrum of (Z_SZ_S)-**2DeF** ($CDCl_3$, 20 °C).

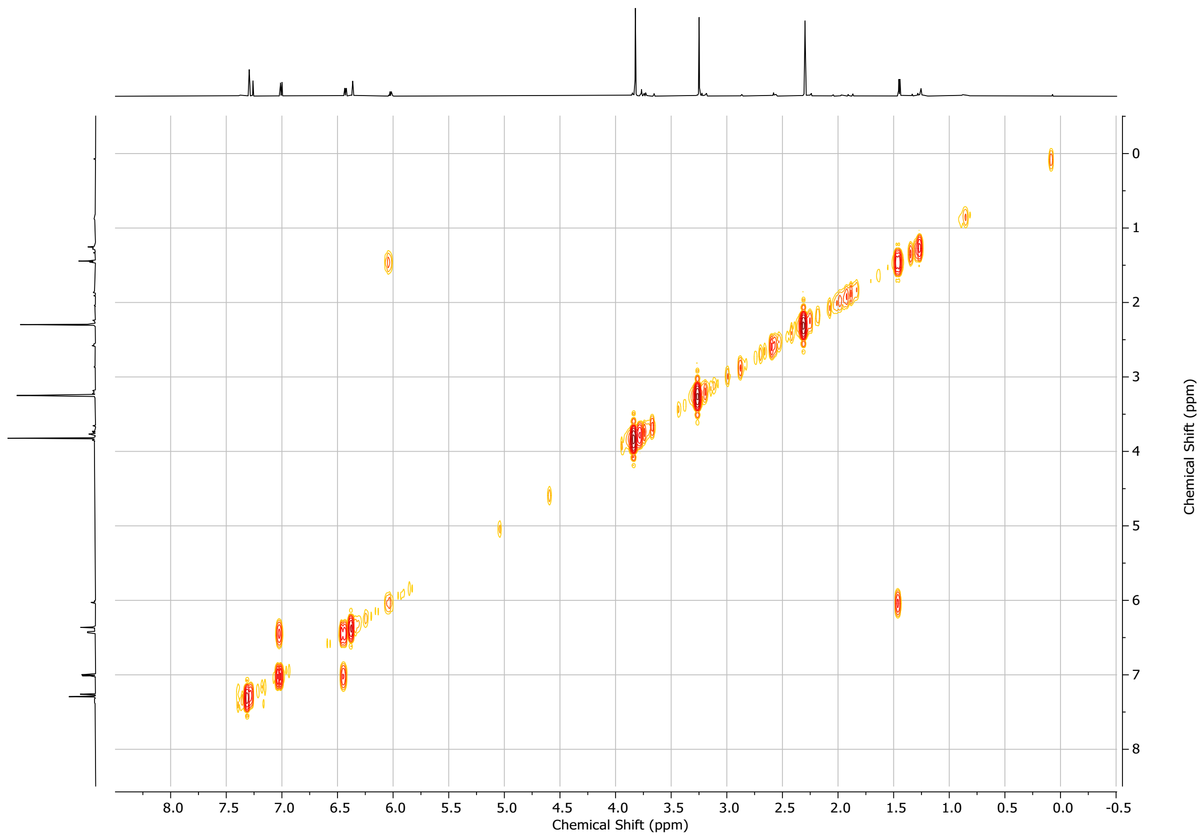

Figure S81. *COSY NMR spectrum of (Z_SZ_S)-**2DeF** ($CDCl_3$, 20 °C).*

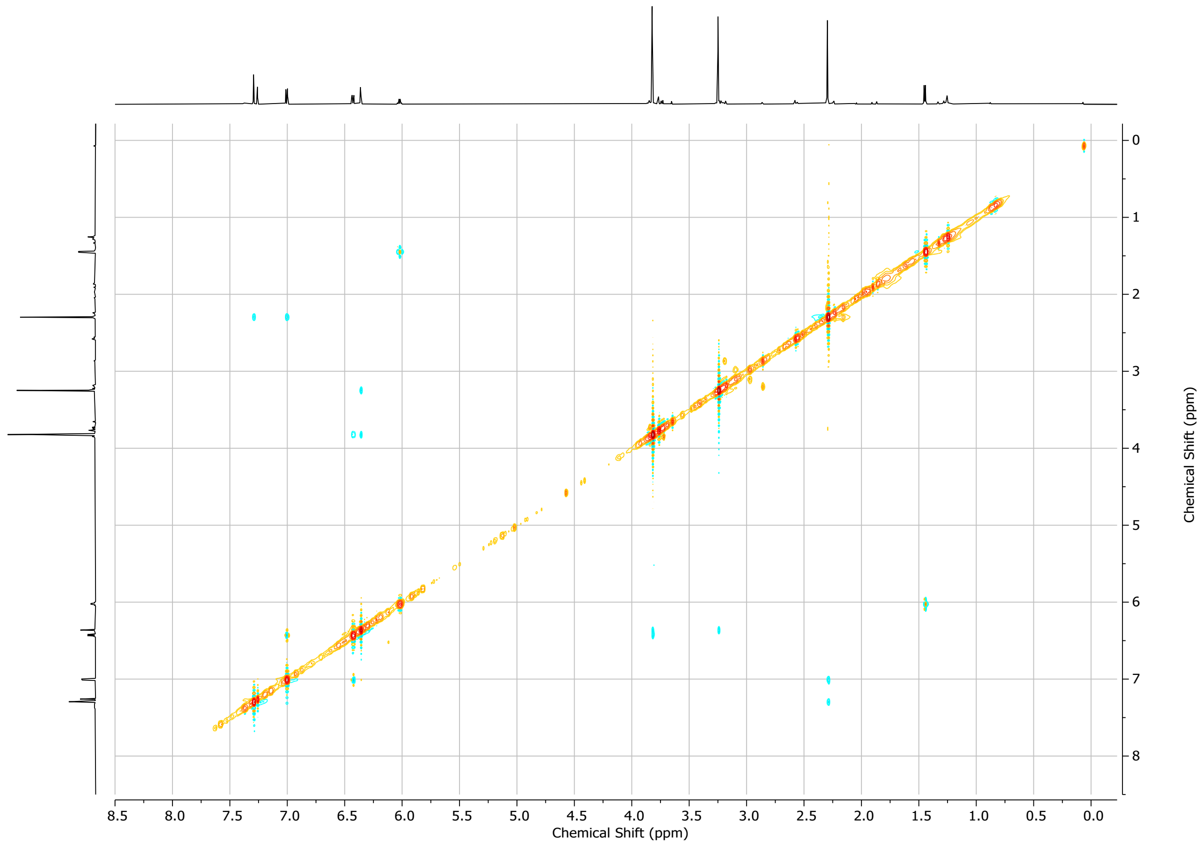

Figure S82. *NOESY NMR spectrum of (Z_SZ_S)-**2DeF** ($CDCl_3$, 20 °C).*

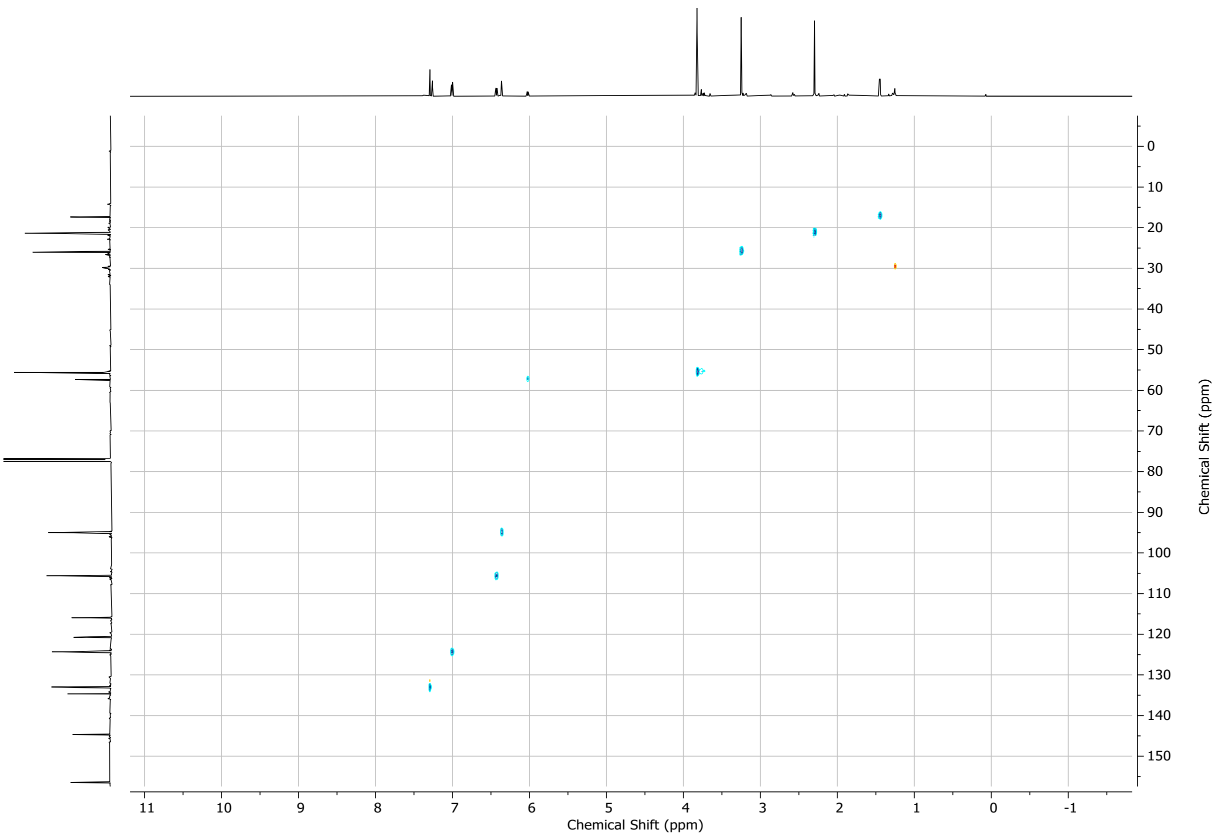

***Figure S83**. HSQC NMR spectrum of (Z_SZ_S)-**2DeF** ($CDCl_3$, 20 °C).*

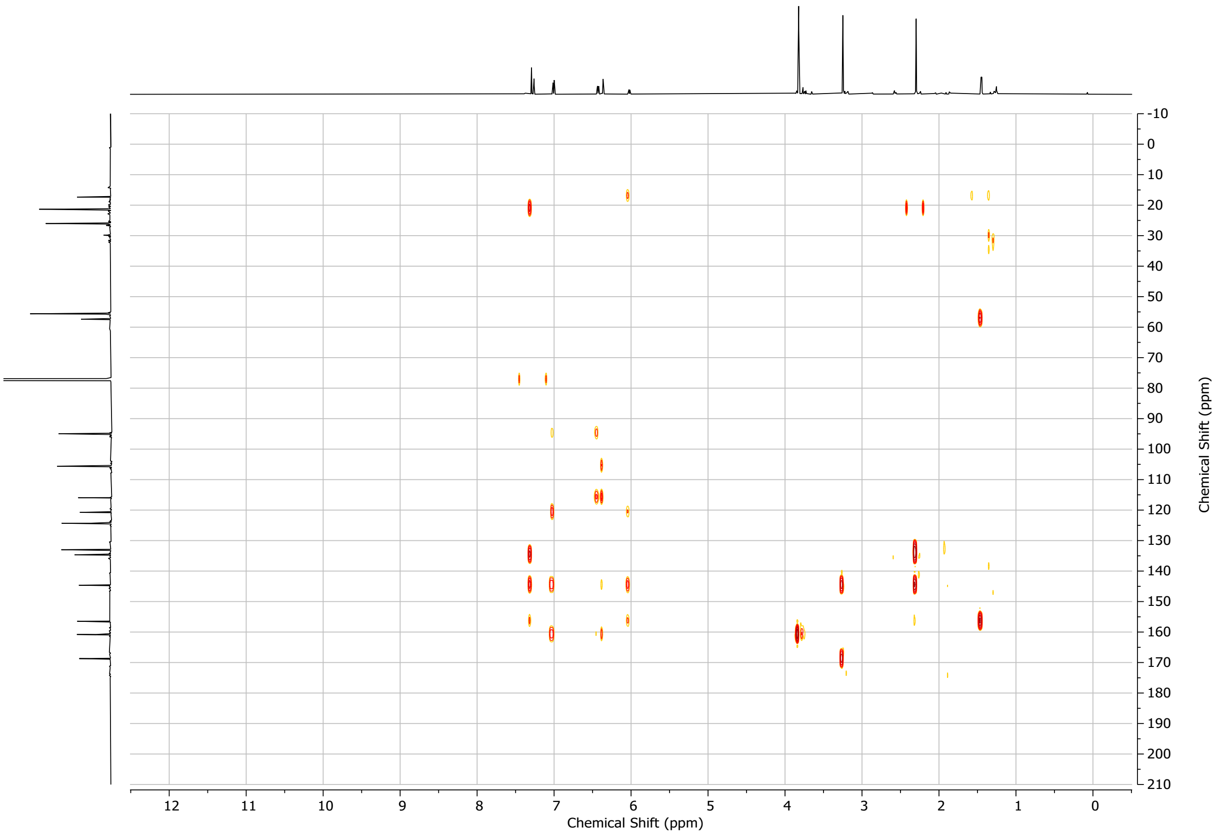

***Figure S84**. HMBC NMR spectrum of (Z_SZ_S)-**2DeF** ($CDCl_3$, 20 °C).*

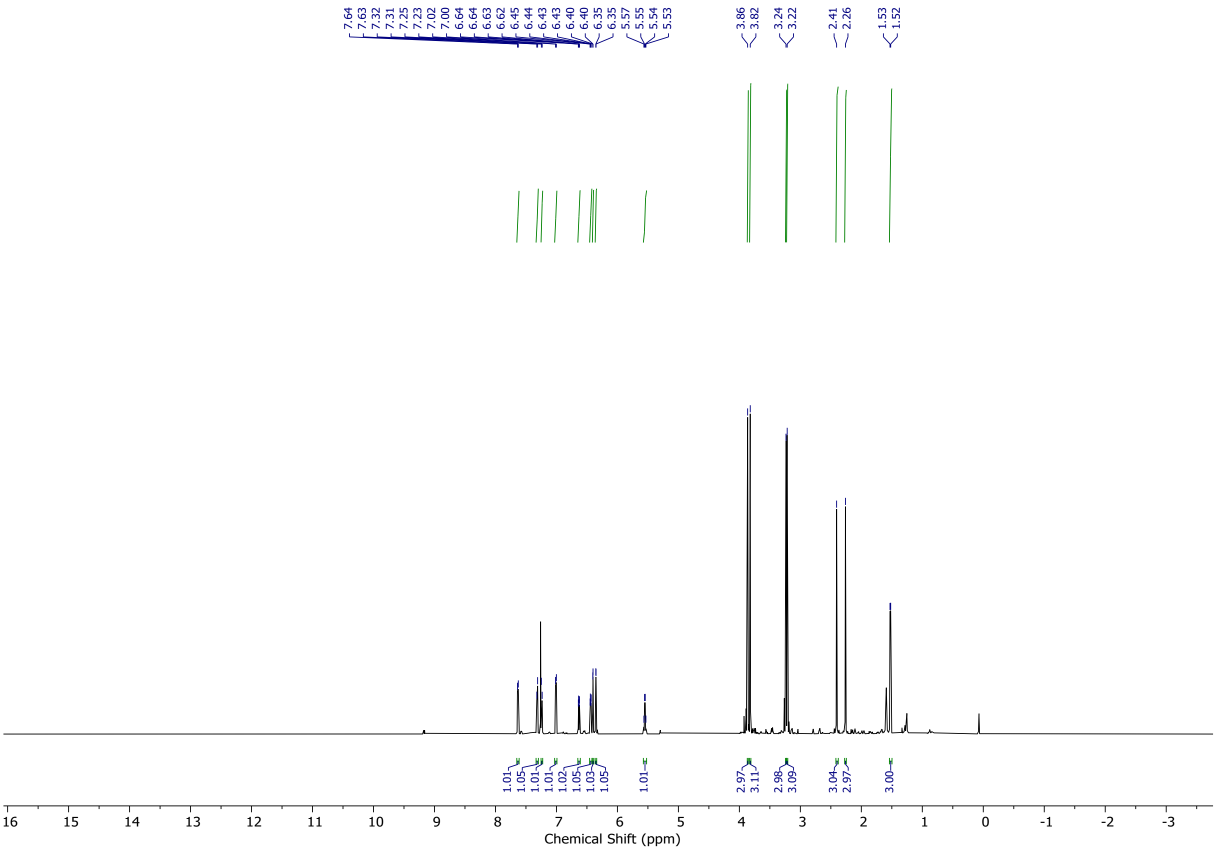

Figure S85. ^{1}*H NMR spectrum of (*E_SZ_S*)-**2DeF** (CDCl*$_3$*, 20 °C).*

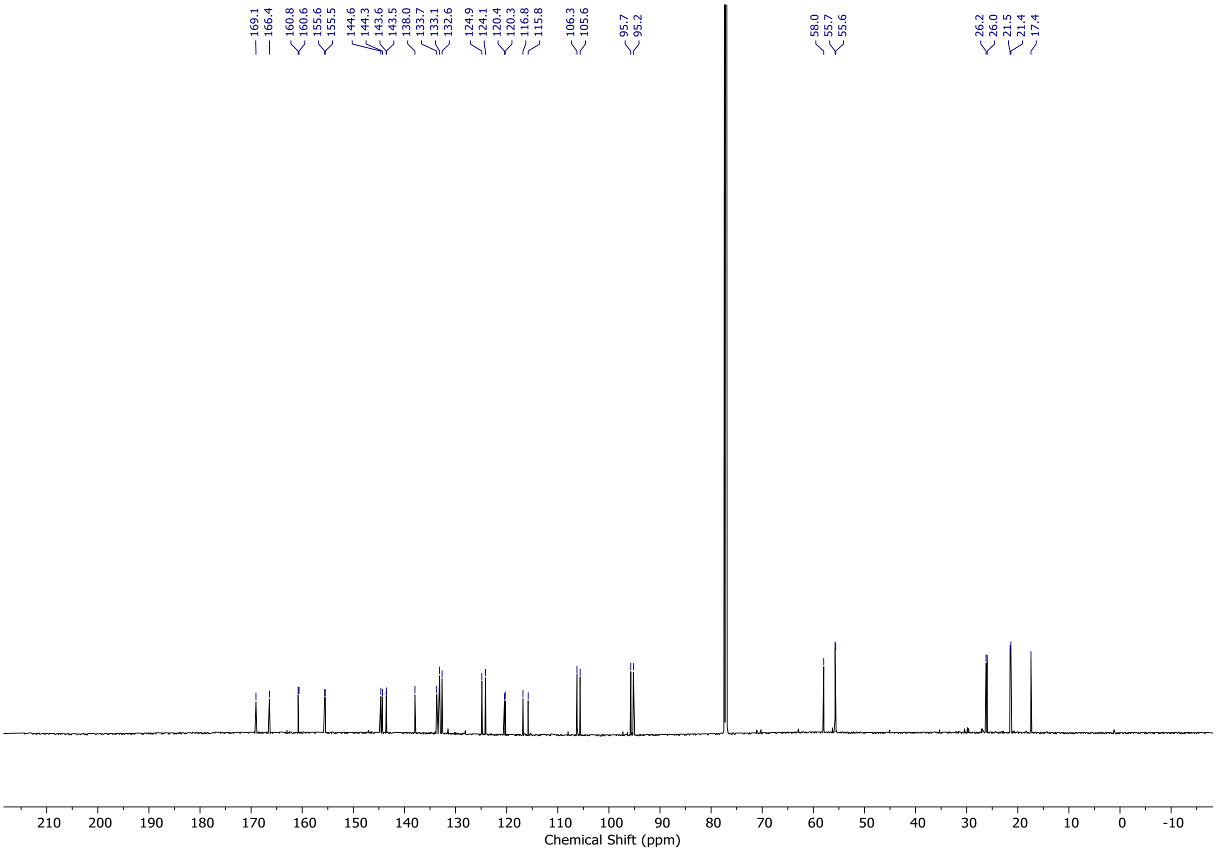

Figure S86. ^{13}*C NMR spectrum of (*E_SZ_S*)-**2DeF** (CDCl*$_3$*, 20 °C).*

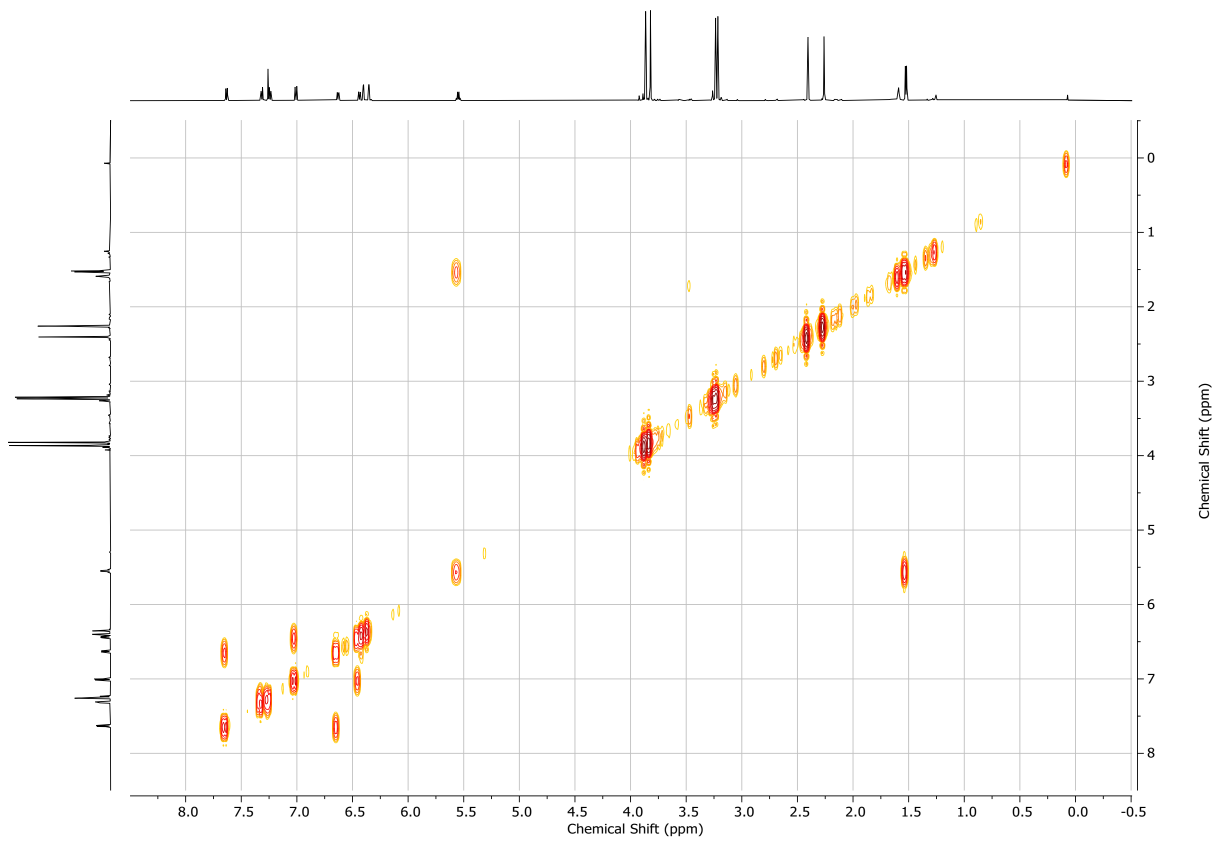

Figure S87. *COSY NMR spectrum of (E_SZ_S)-**2DeF** ($CDCl_3$, 20 °C).*

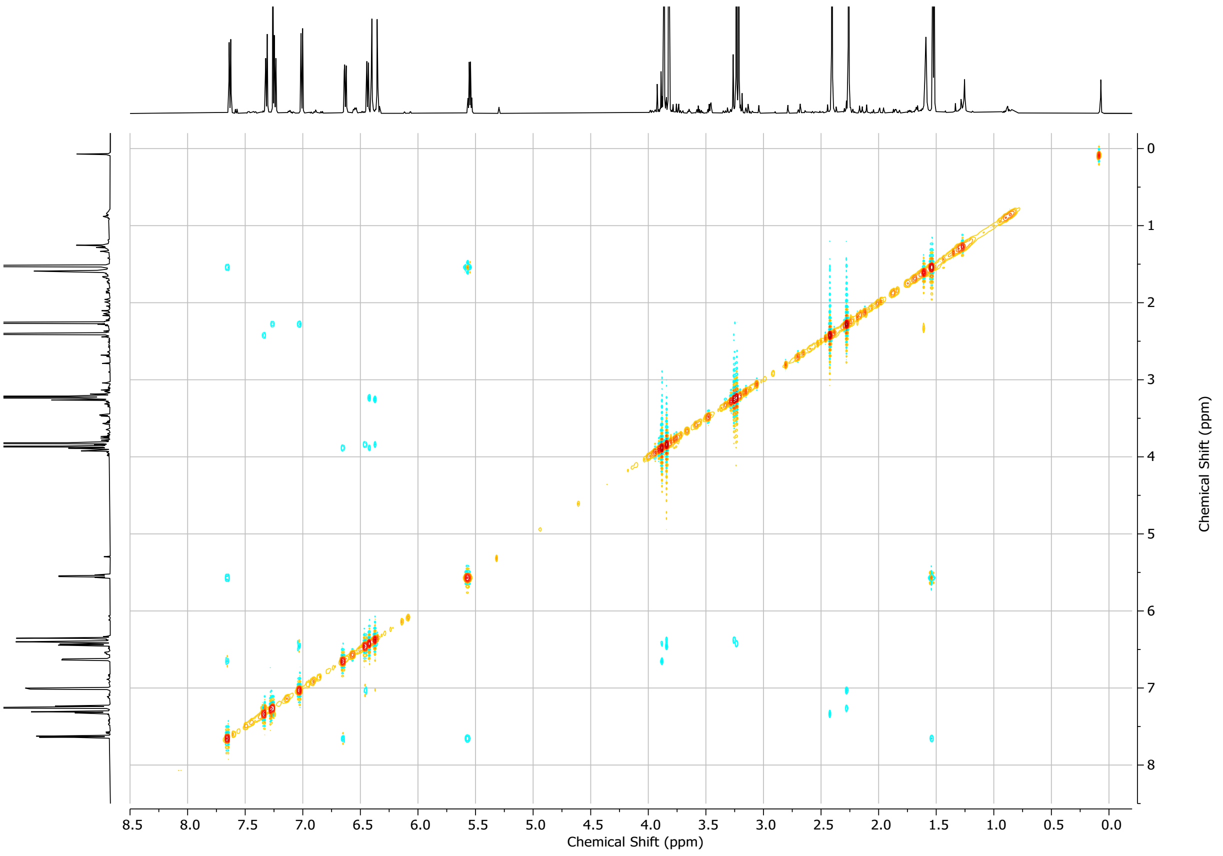

Figure S88. *NOESY NMR spectrum of (E_SZ_S)-**2DeF** ($CDCl_3$, 20 °C).*

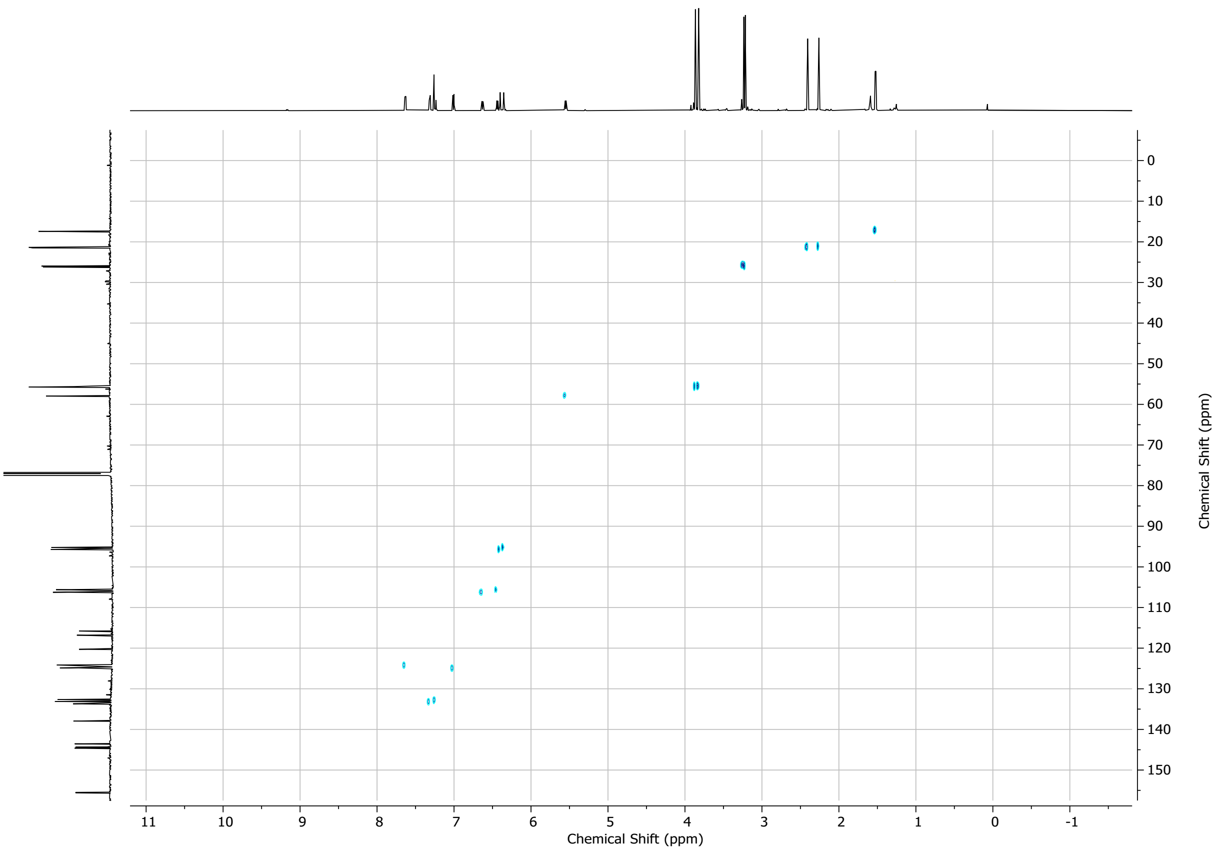

Figure S89. *HSQC NMR spectrum of* (E_SZ_S)-***2DeF*** *($CDCl_3$, 20 °C).*

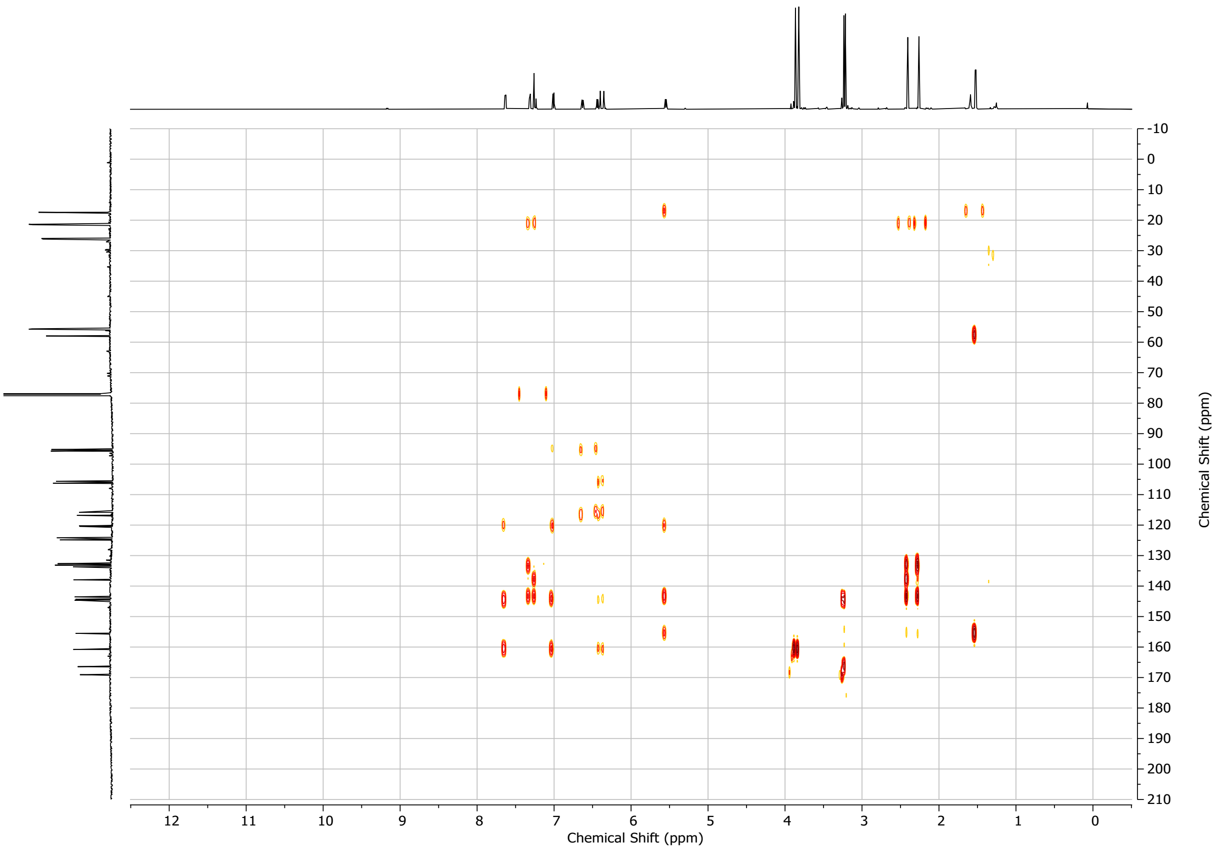

Figure S90. *HMBC NMR spectrum of* (E_SZ_S)-***2DeF*** *($CDCl_3$, 20 °C).*

Motor 3

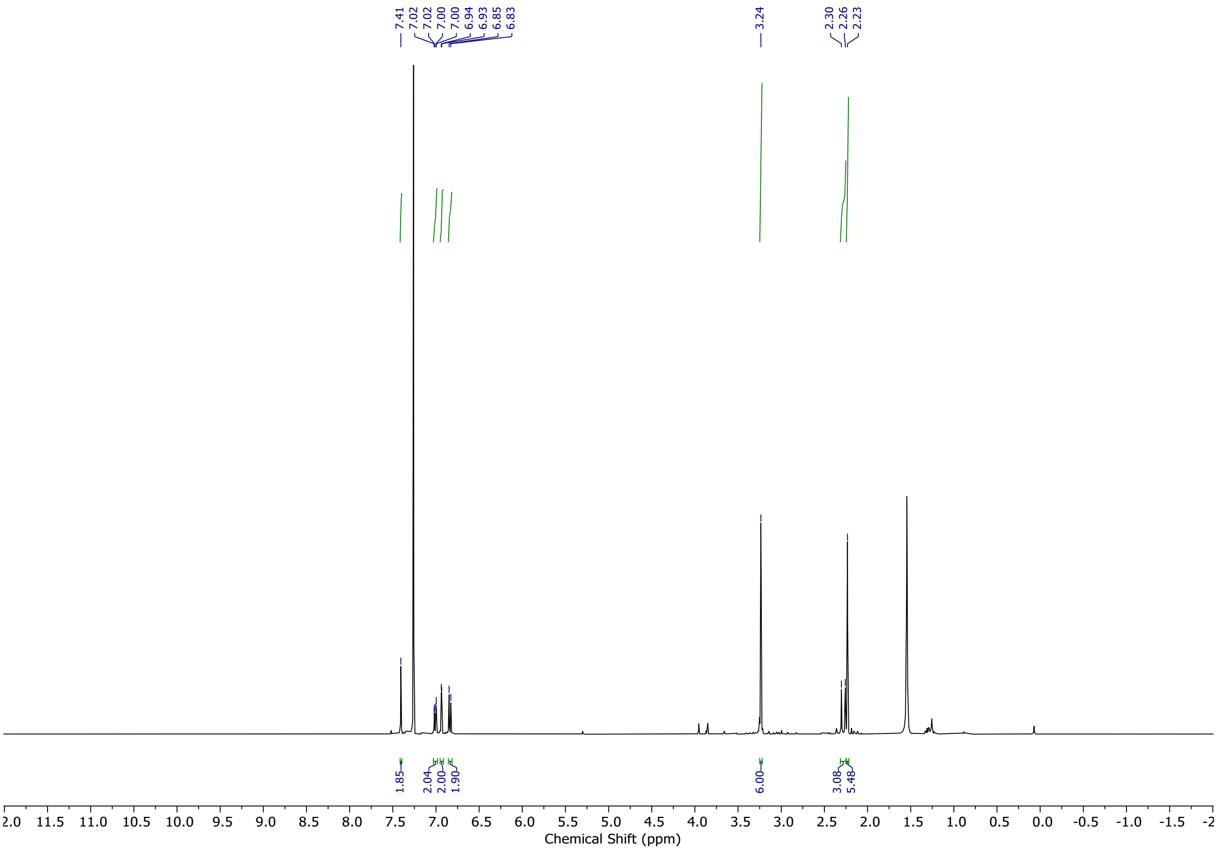

Figure S91. *¹H NMR spectrum of (Z_SZ_S)-**3** ($CDCl_3$, 20 °C).*

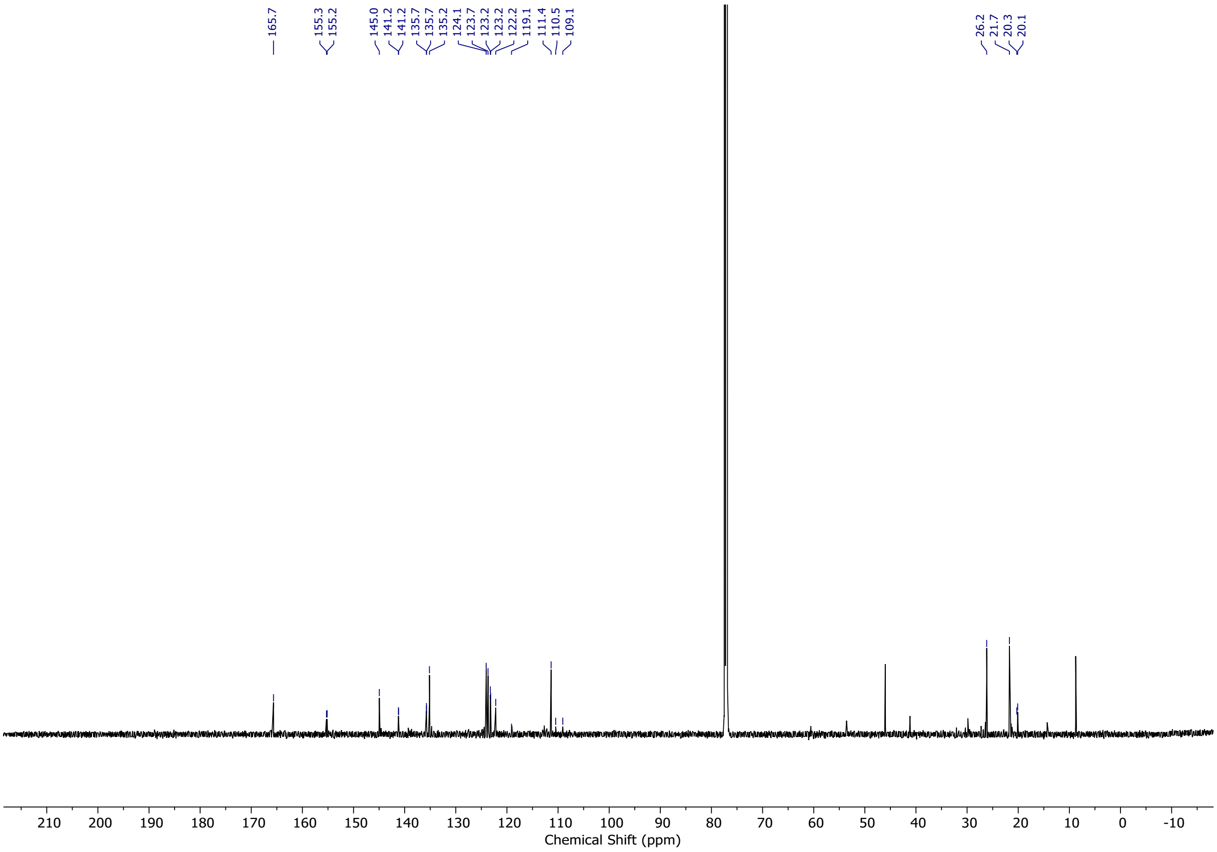

Figure S92. *¹³C NMR spectrum of (Z_SZ_S)-**3** ($CDCl_3$, 20 °C).*

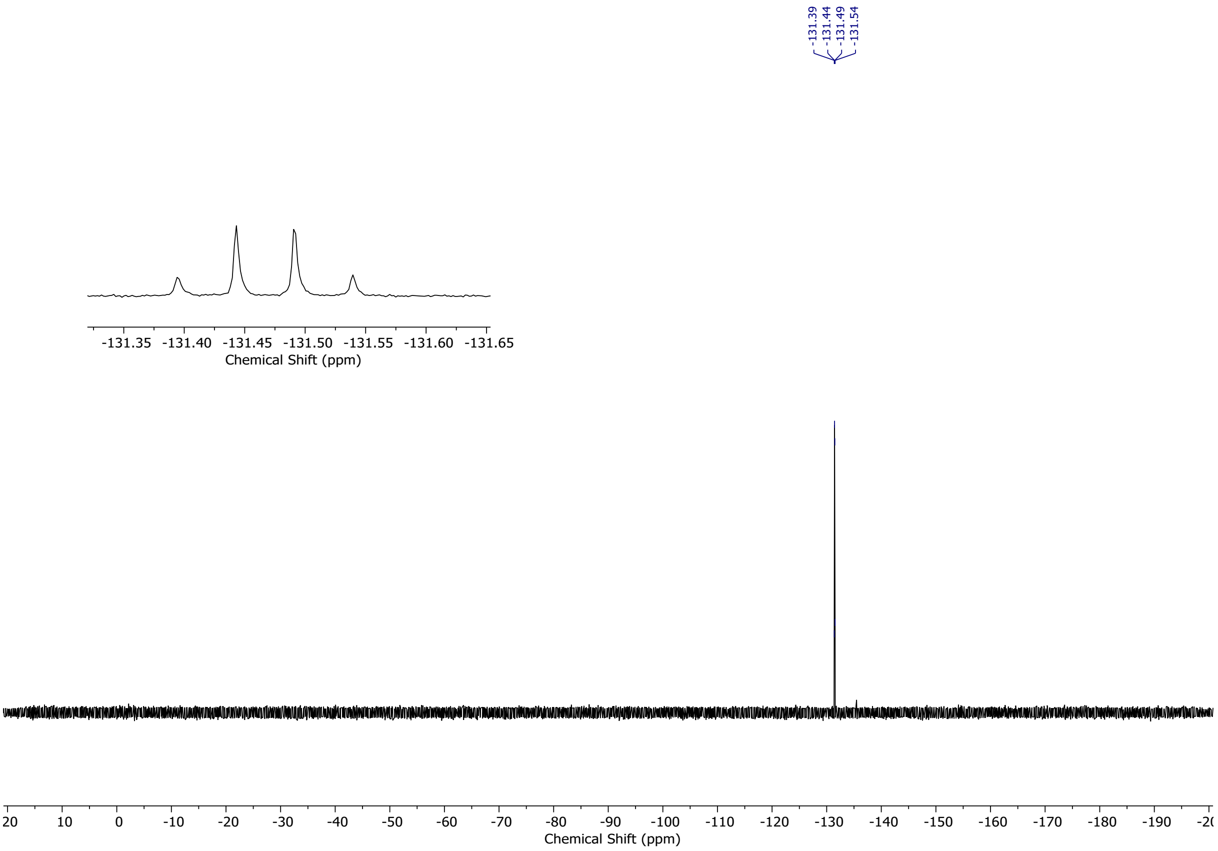

Figure S93. *^{19}F NMR spectrum of (Z_SZ_S)-**3** ($CDCl_3$, 20 °C).*

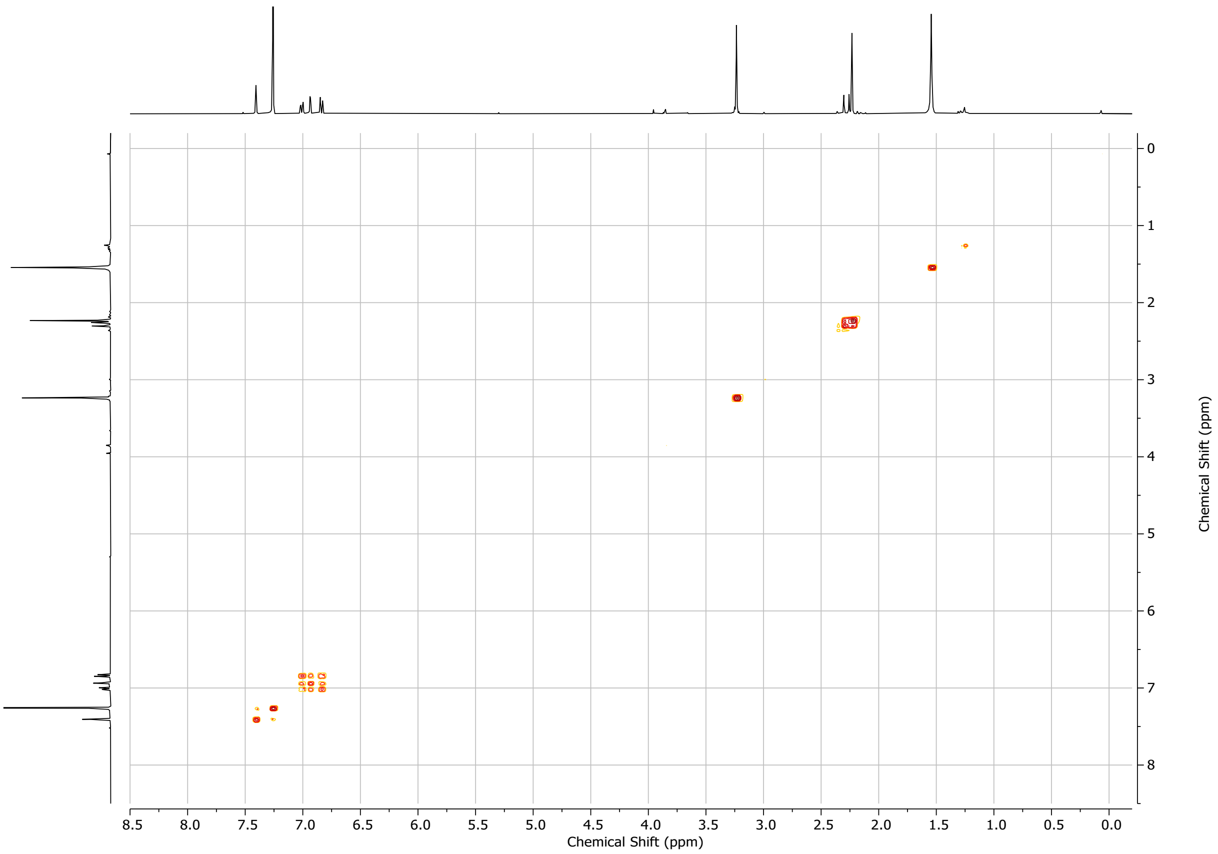

Figure S94. *COSY NMR spectrum of (Z_SZ_S)-**3** ($CDCl_3$, 20 °C).*

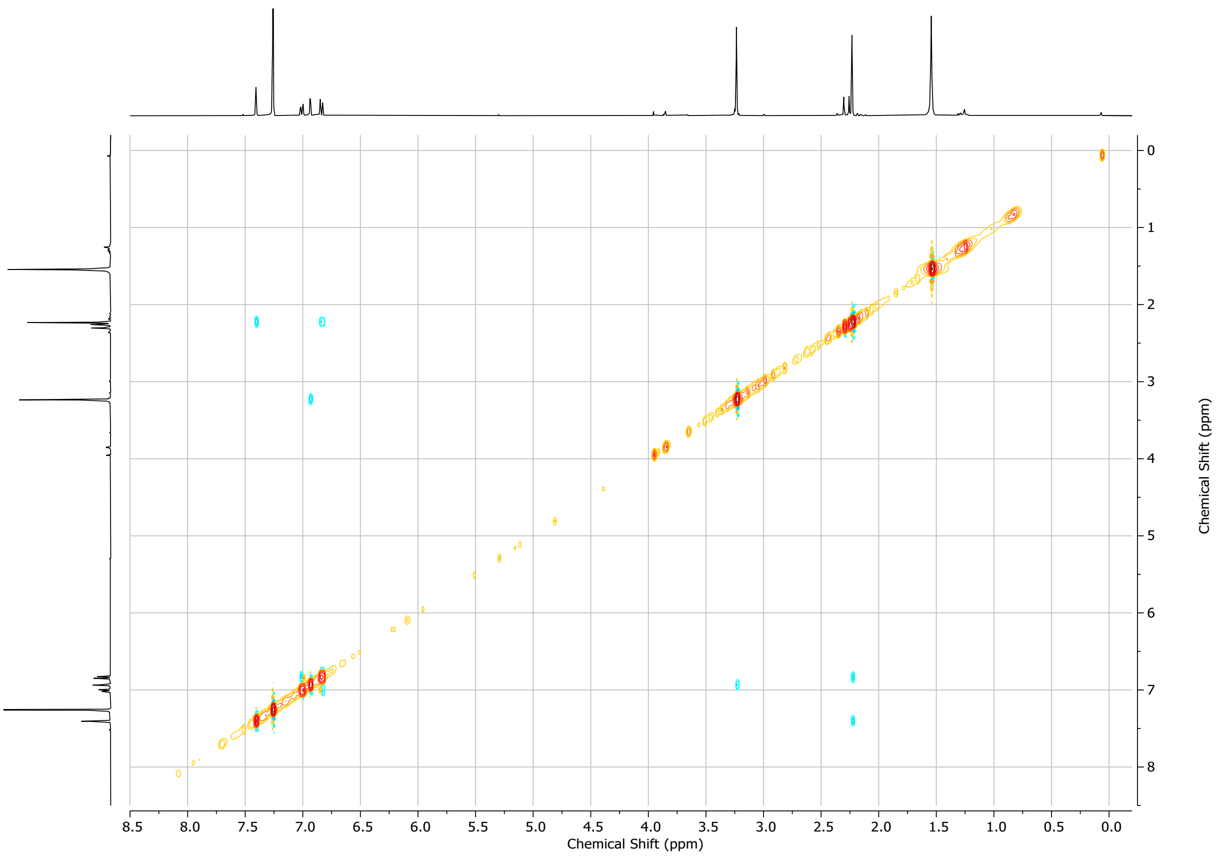

Figure S95. *NOESY NMR spectrum of (Z$_S$Z$_S$)-**3** (CDCl$_3$, 20 °C).*

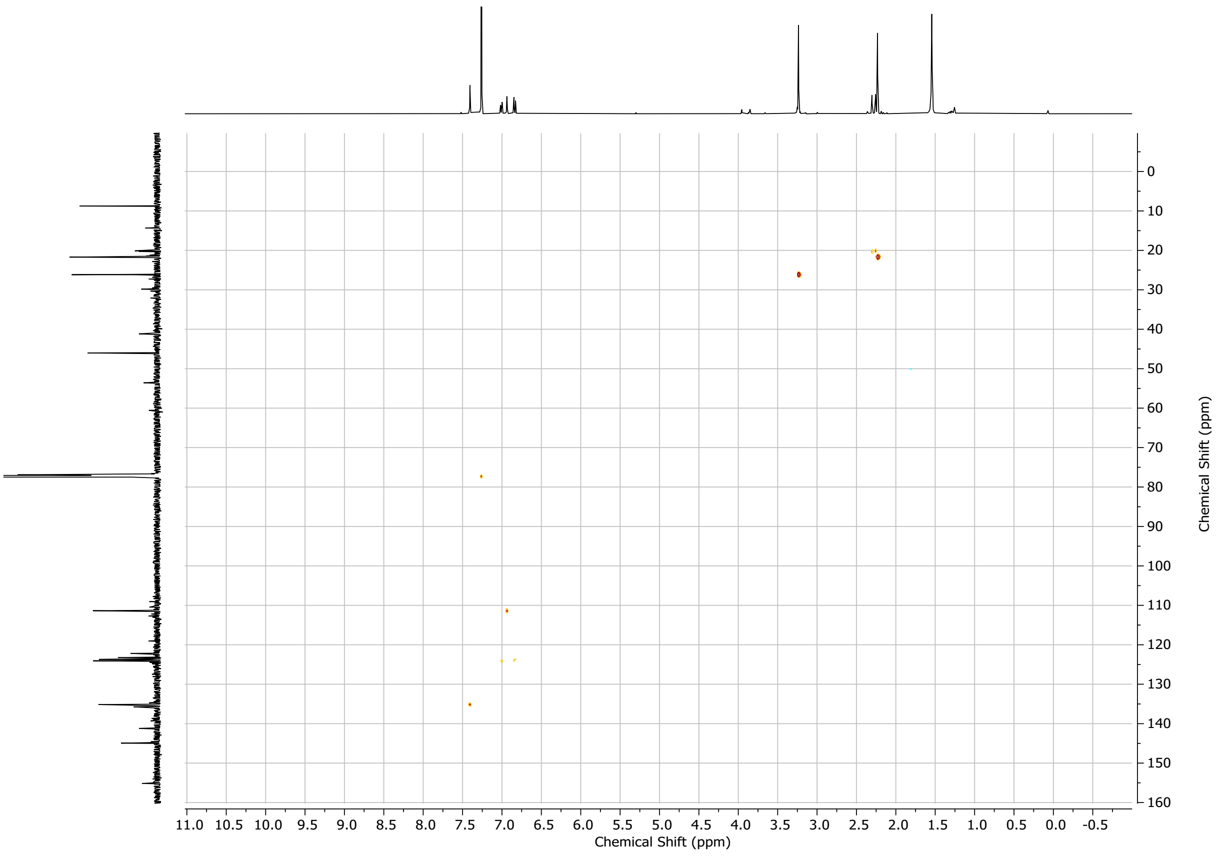

Figure S96. *HSQC NMR spectrum of (Z$_S$Z$_S$)-**3** (CDCl$_3$, 20 °C).*

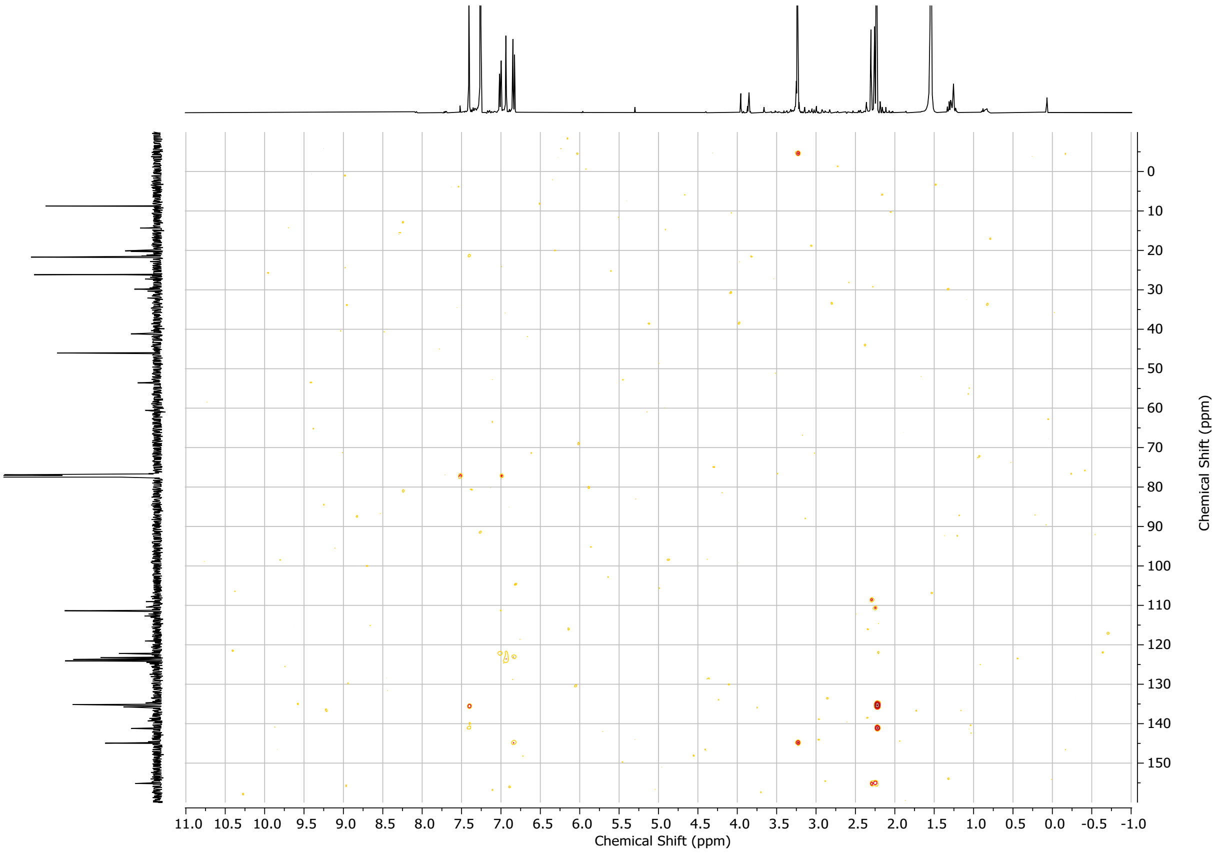

Figure S97. *HMBC NMR spectrum of (Z_SZ_S)-**3** ($CDCl_3$, 20 °C).*

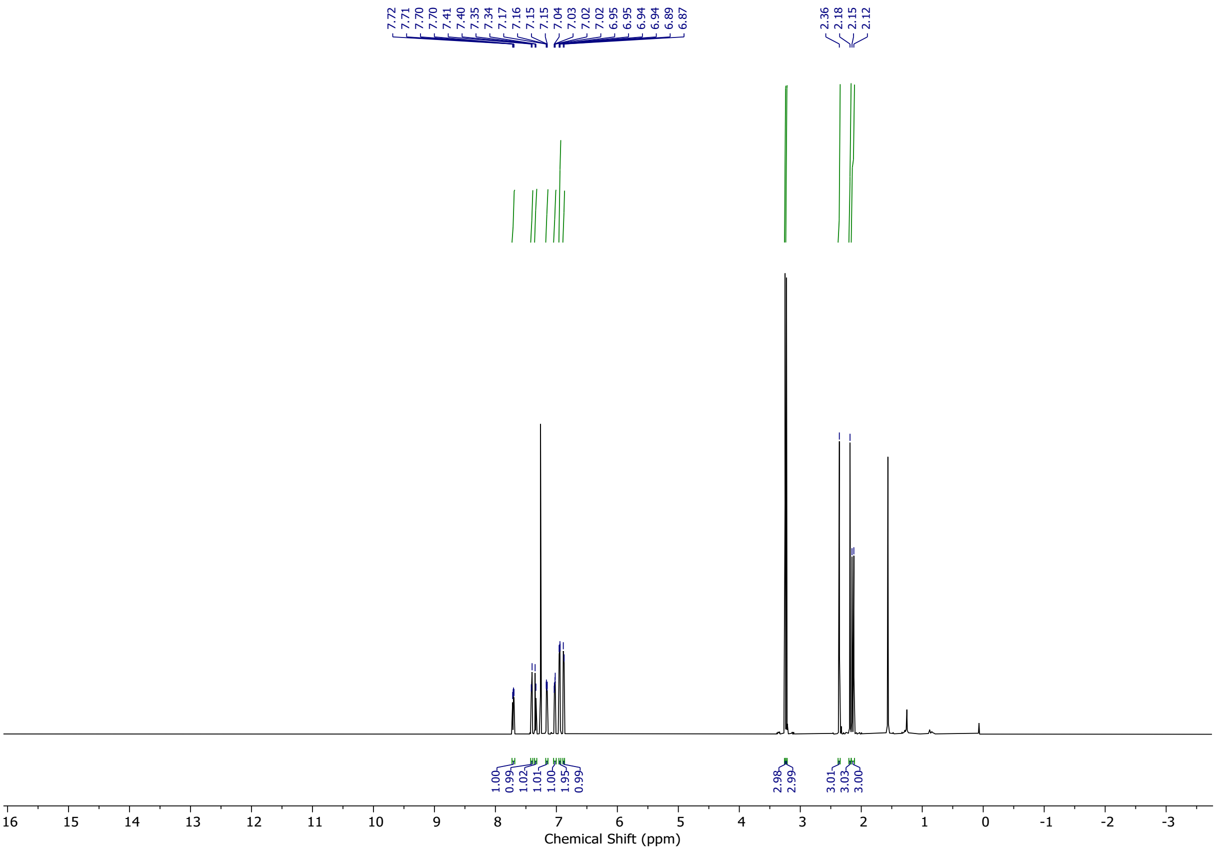

Figure S98. *^{1}H NMR spectrum of (E_SZ_S)-**3** ($CDCl_3$, 20 °C).*

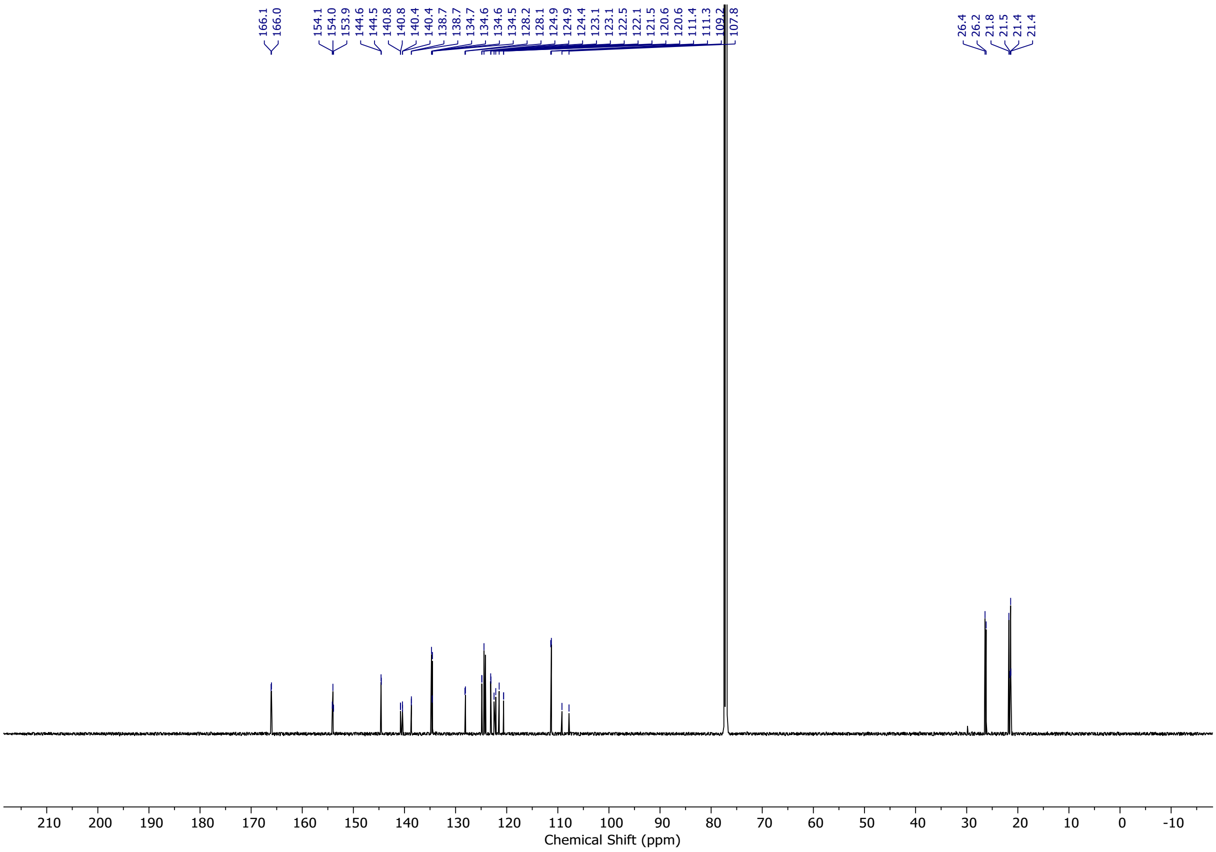

Figure S99. *^{13}C NMR spectrum of (E_SZ_S)-**3** ($CDCl_3$, 20 °C).*

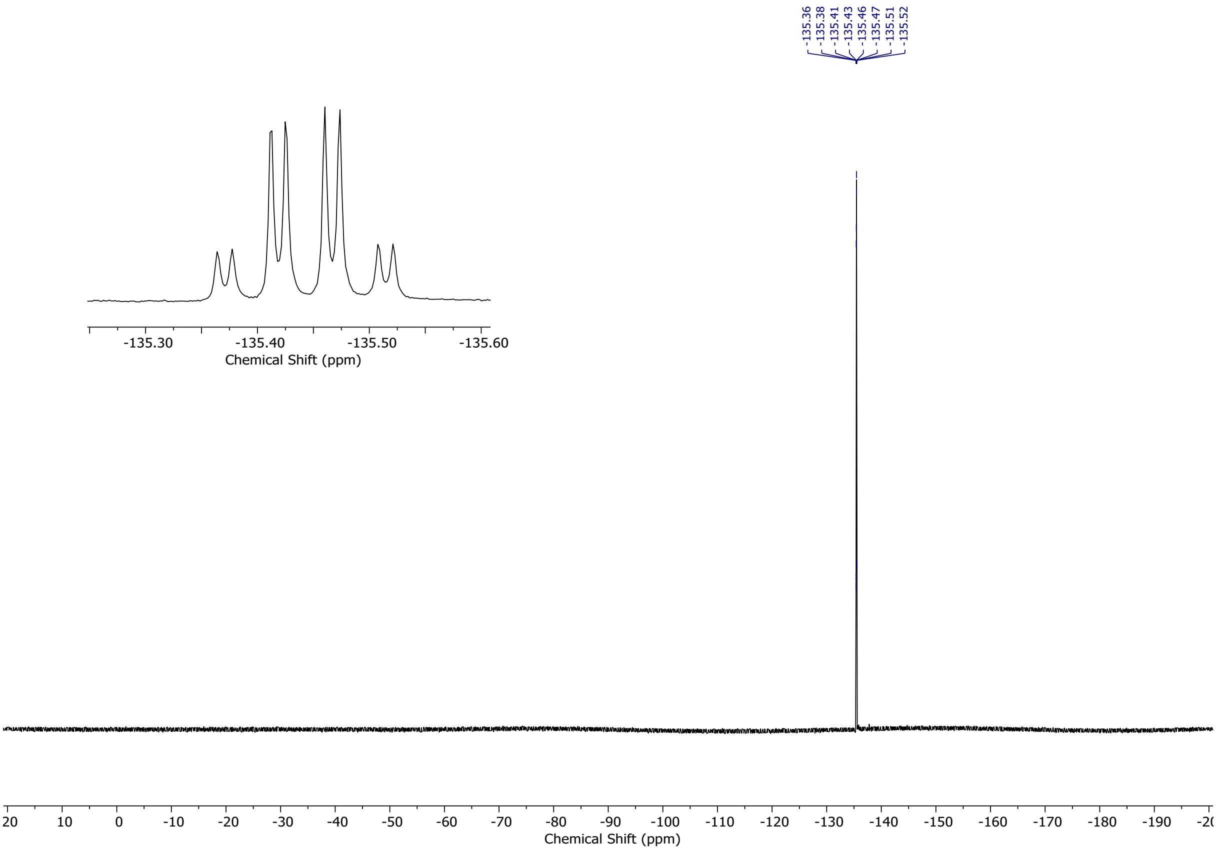

Figure S100. *^{19}F NMR spectrum of (E_SZ_S)-**3** ($CDCl_3$, 20 °C).*

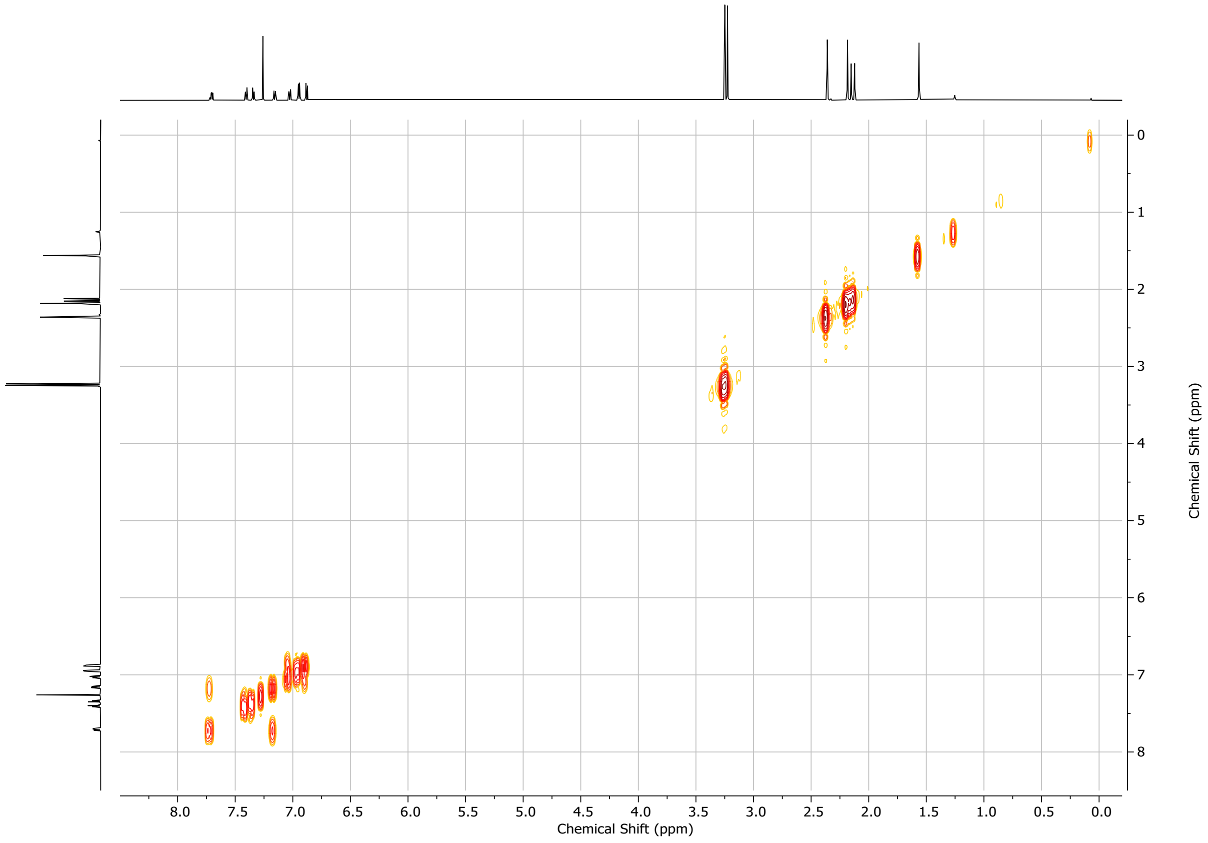

Figure S101. *COSY NMR spectrum of (E_SZ_S)-**3** ($CDCl_3$, 20 °C).*

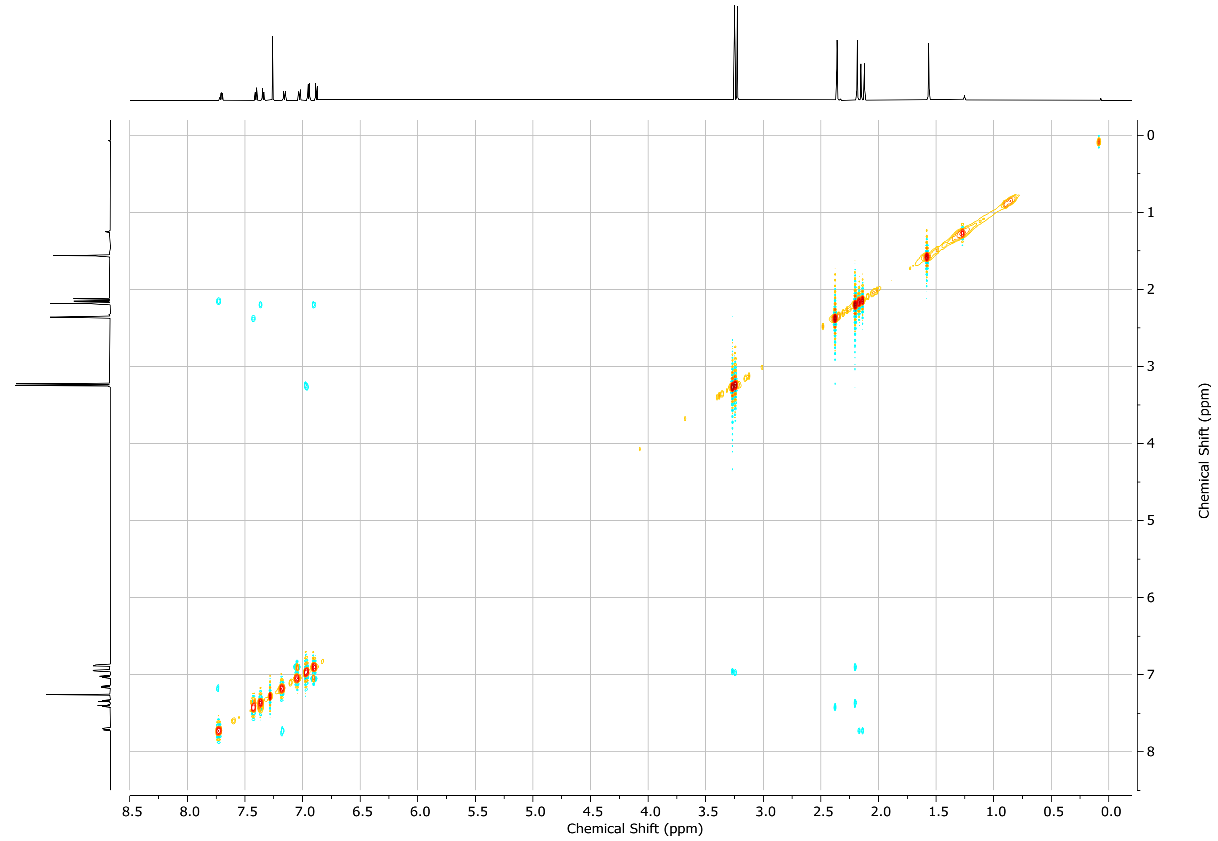

Figure S102. *NOESY NMR spectrum of (E_SZ_S)-**3** ($CDCl_3$, 20 °C).*

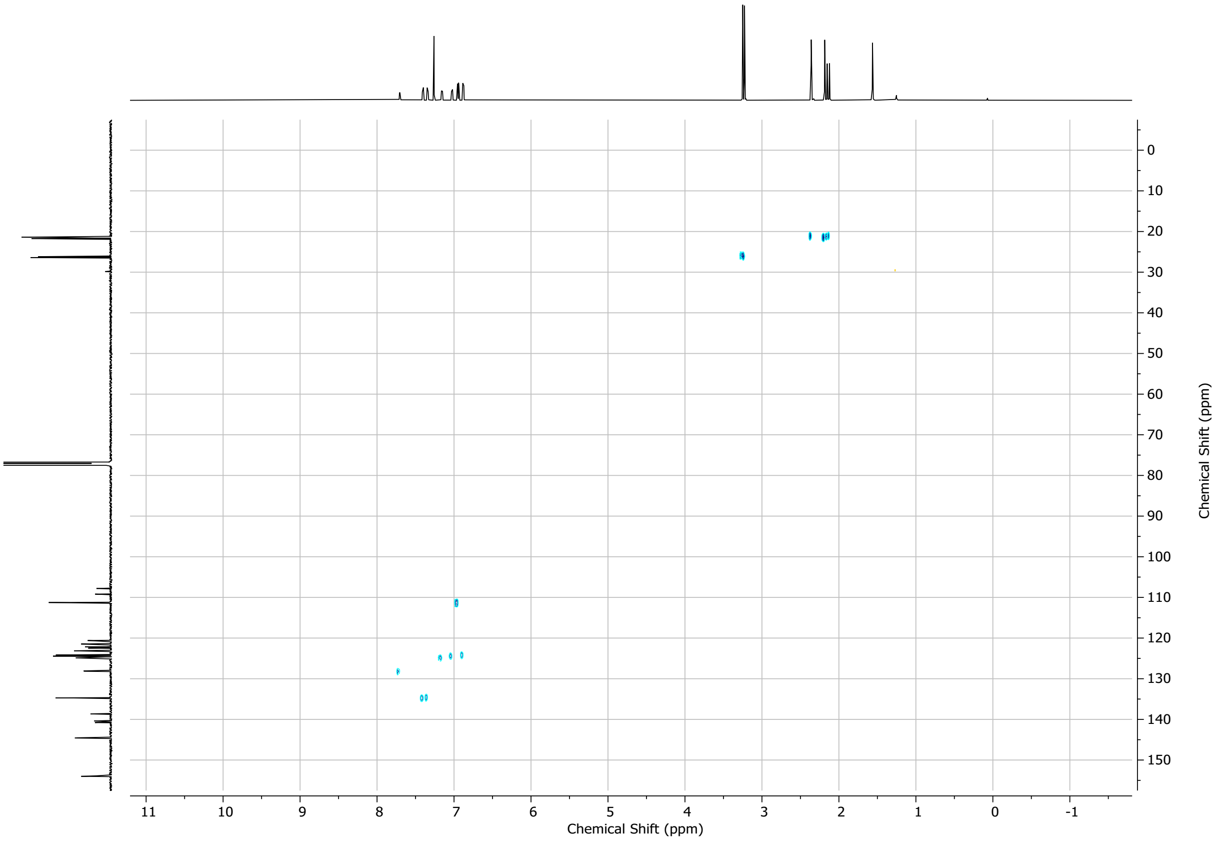

Figure S103. *HSQC NMR spectrum of (E_SZ_S)-**3** ($CDCl_3$, 20 °C).*

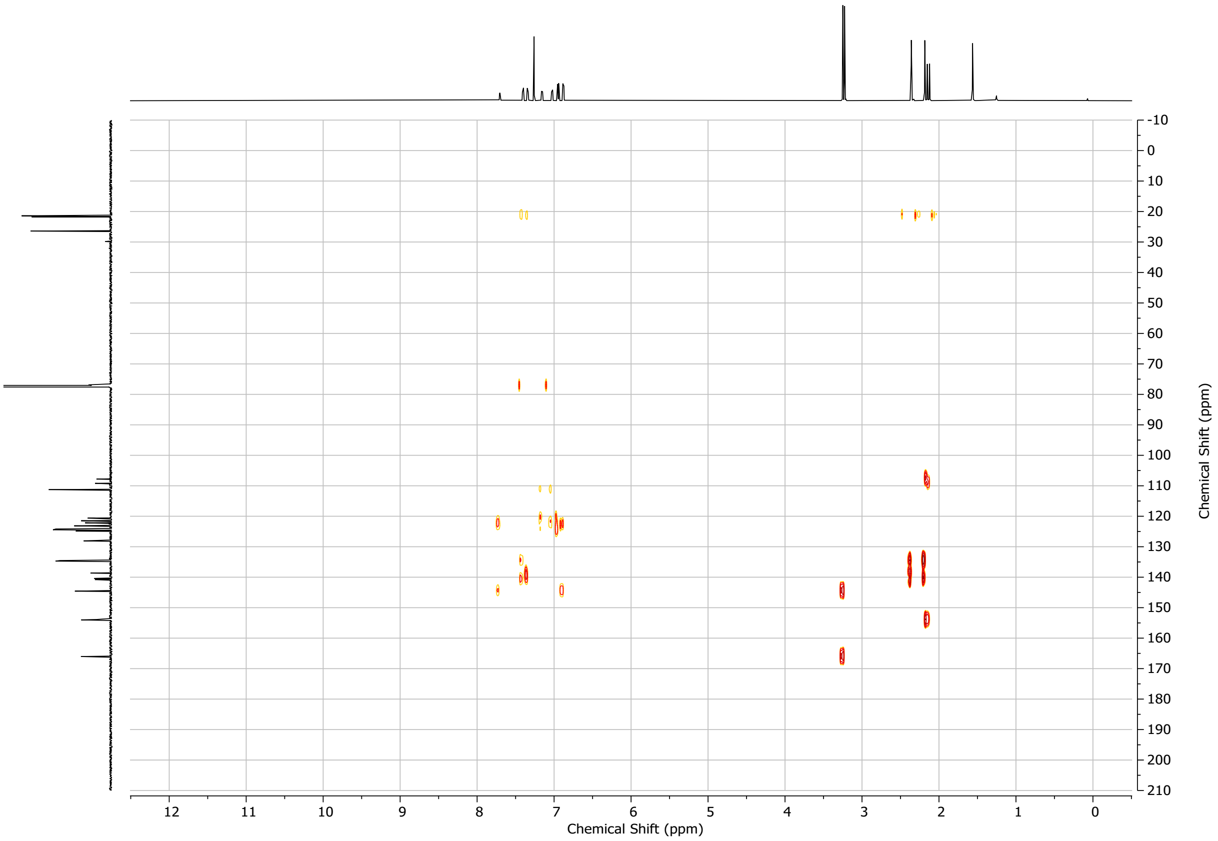

Figure S104. *HMBC NMR spectrum of (E_SZ_S)-**3** ($CDCl_3$, 20 °C).*

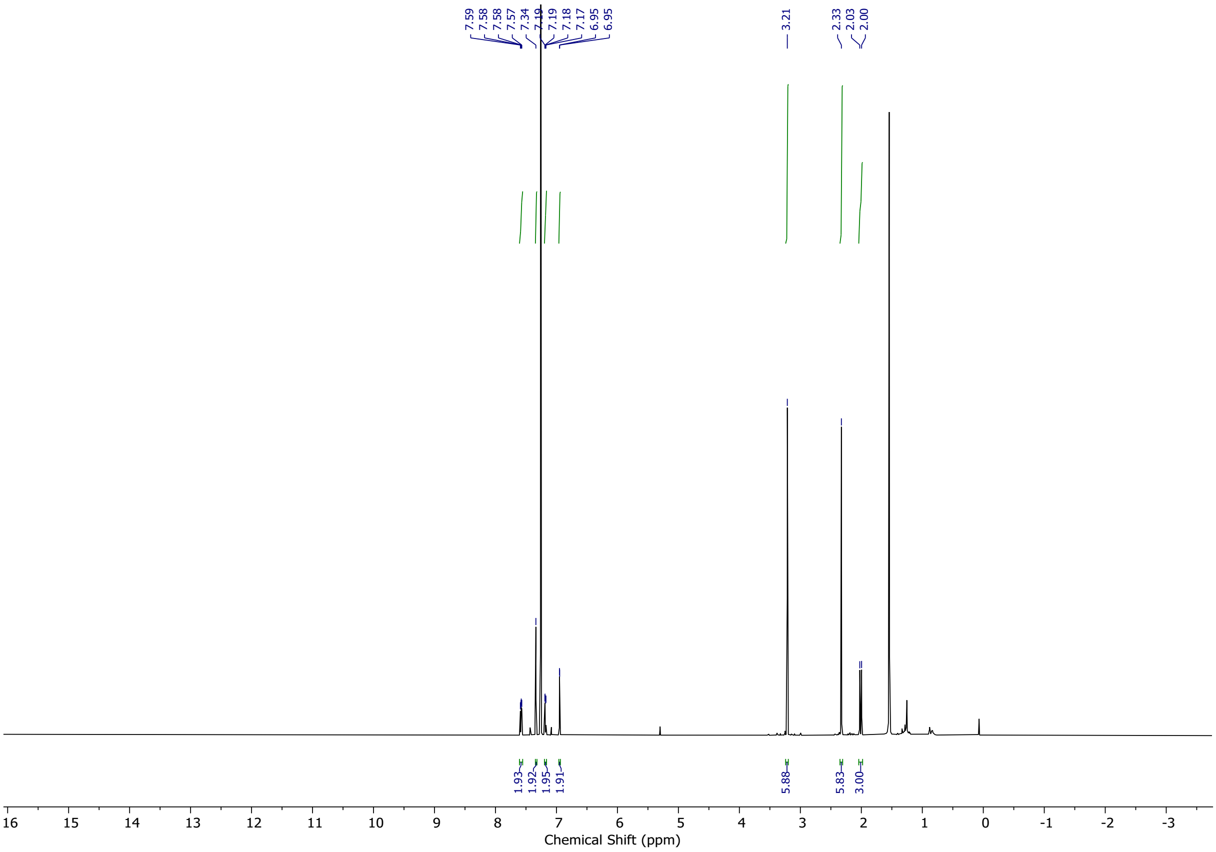

Figure S105. *^{1}H NMR spectrum of (E_SE_S)-**3** ($CDCl_3$, 20 °C).*

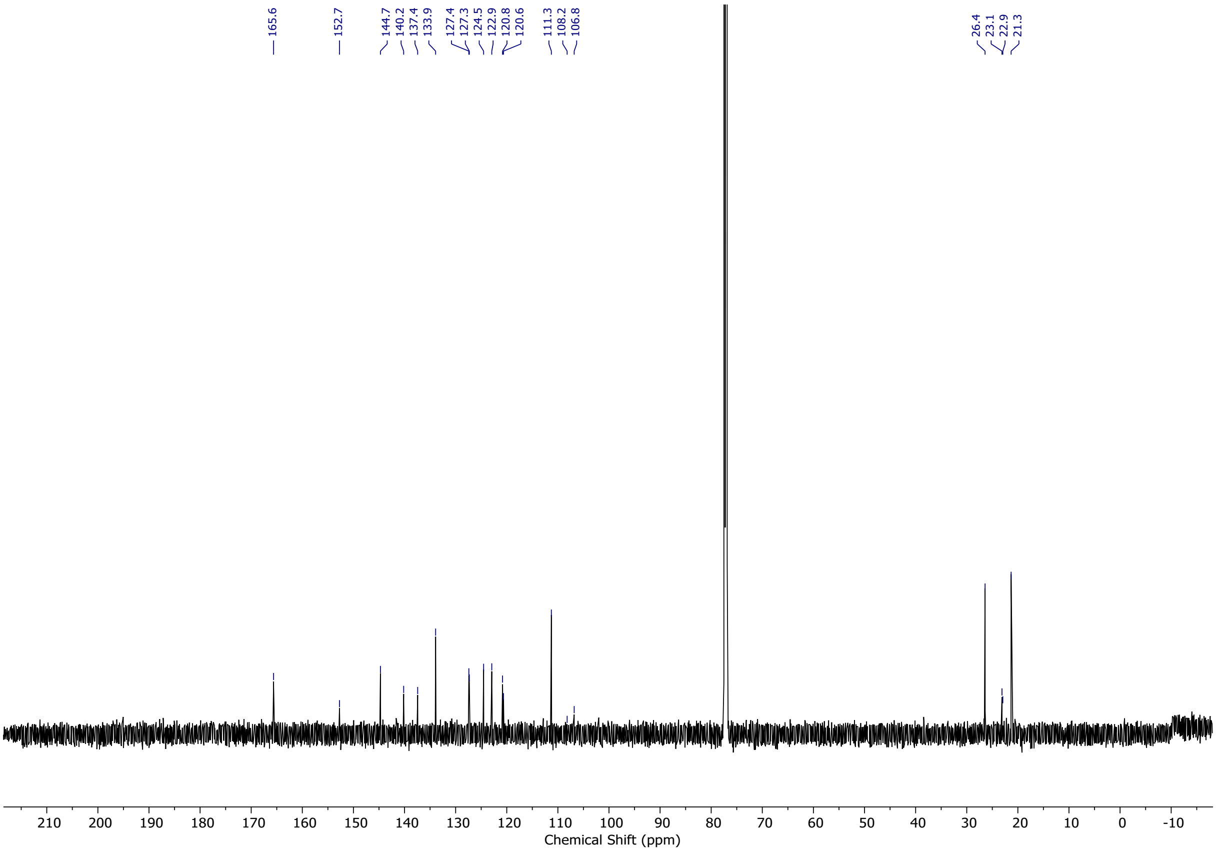

Figure S106. *^{13}C NMR spectrum of (E_SE_S)-**3** ($CDCl_3$, 20 °C).*

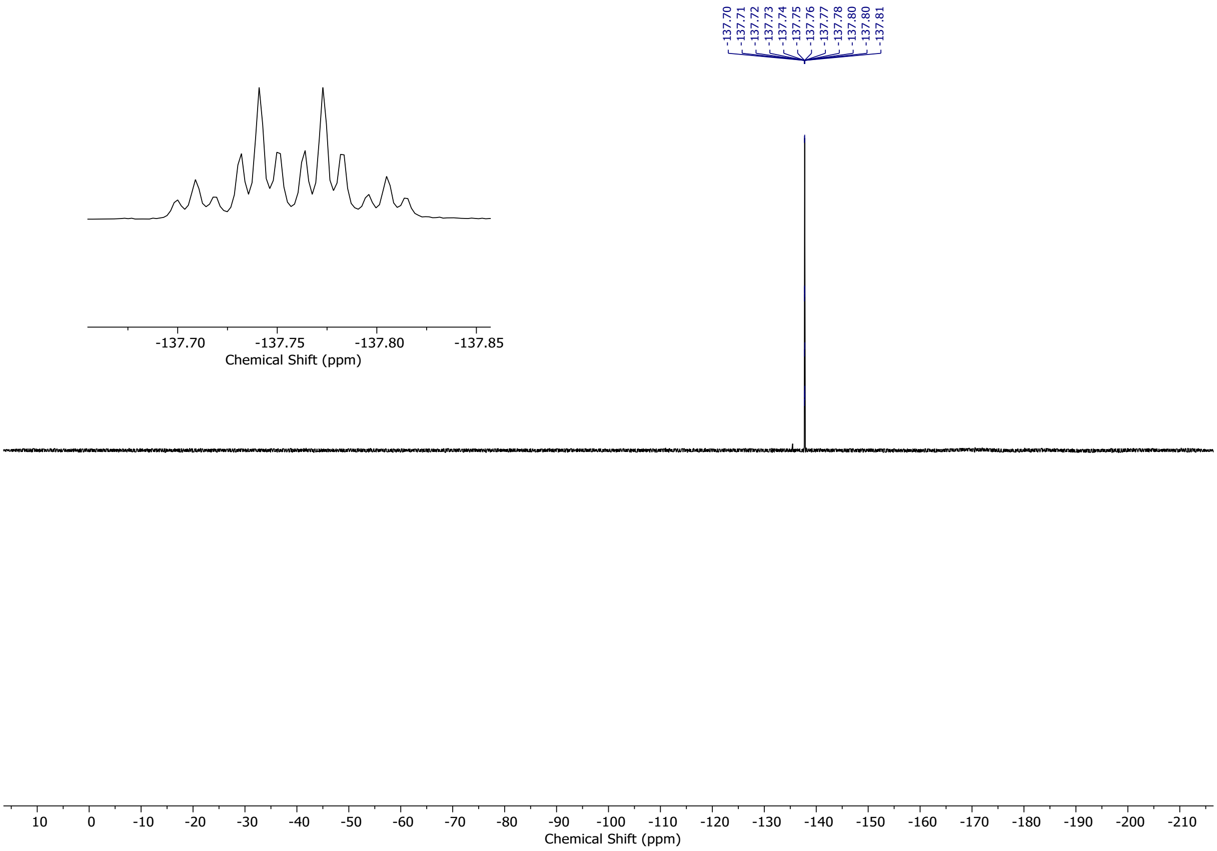

Figure S107. *^{19}F NMR spectrum of (E_SE_S)-**3** ($CDCl_3$, 20 °C).*

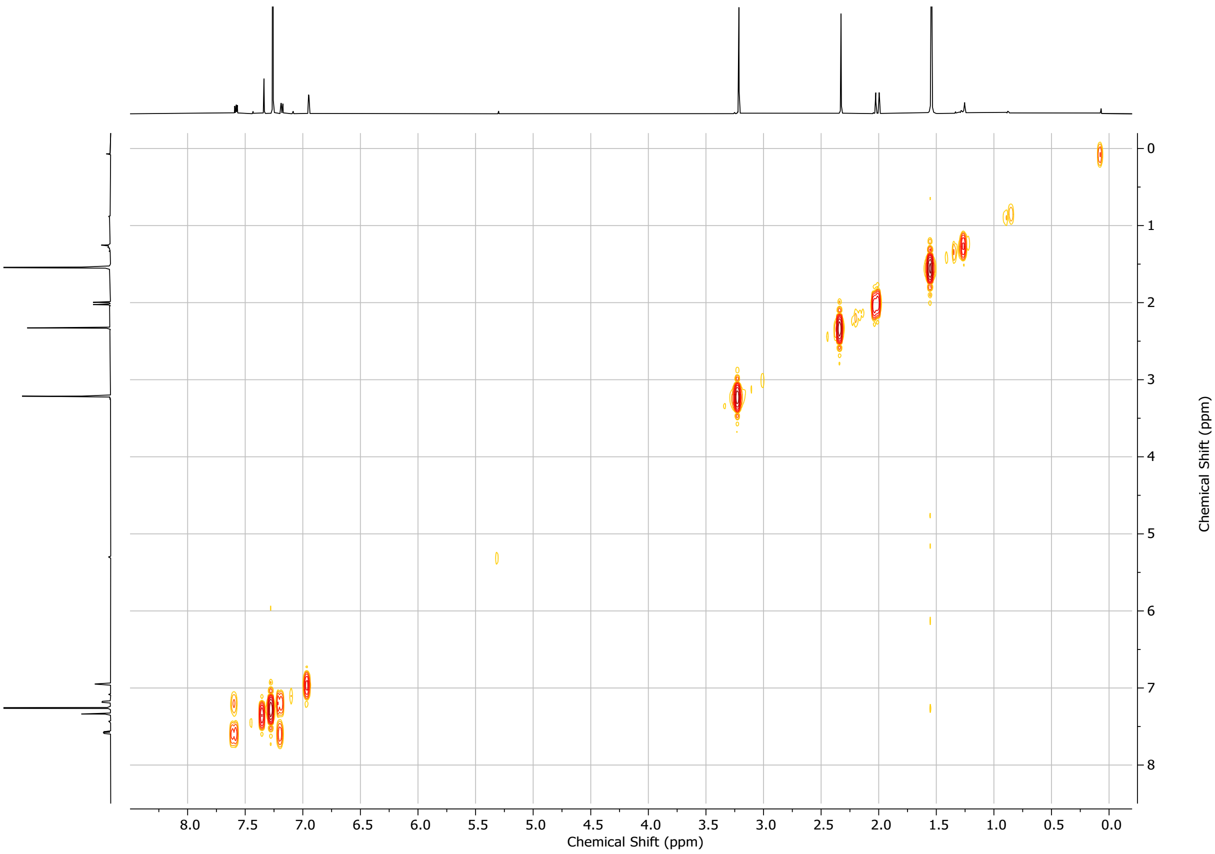

Figure S108. *COSY NMR spectrum of (E_SE_S)-**3** ($CDCl_3$, 20 °C).*

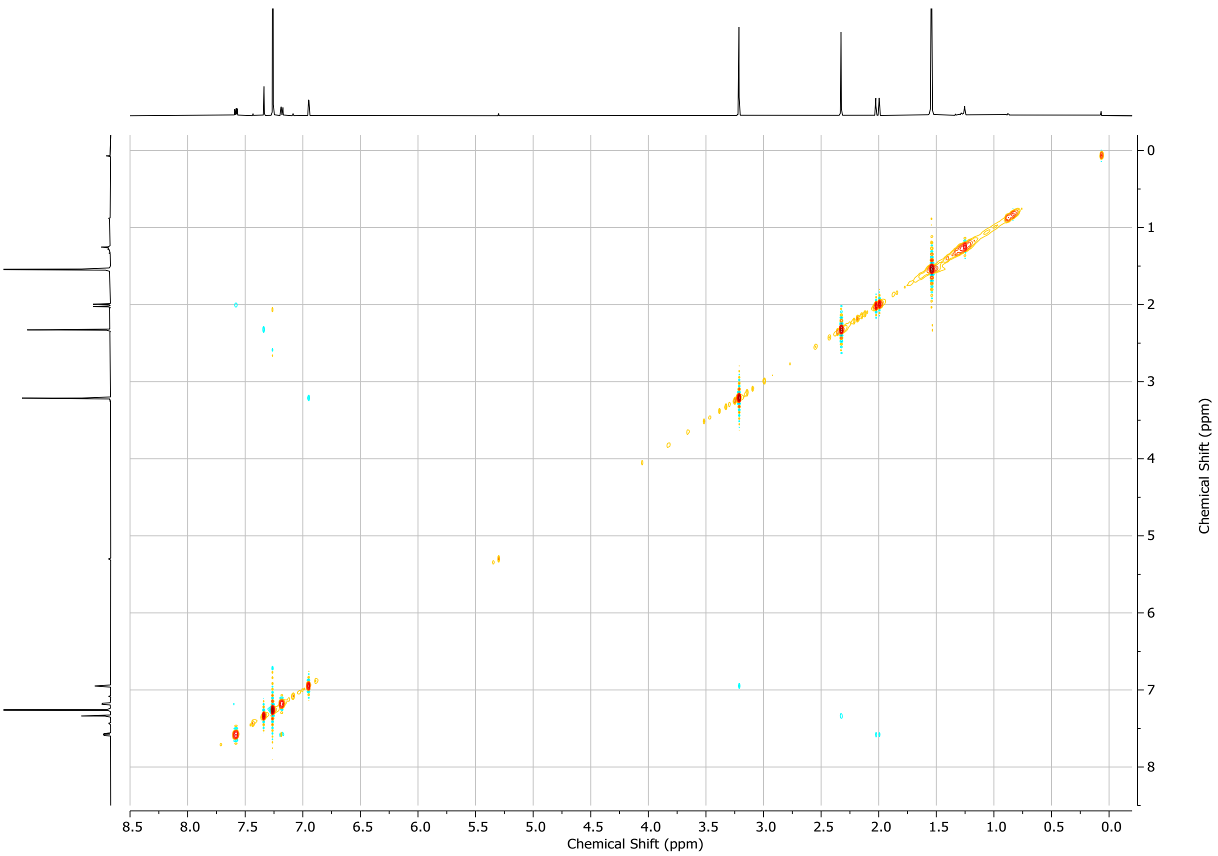

Figure S109. *NOESY NMR spectrum of (E$_S$E$_S$)-**3** (CDCl$_3$, 20 °C).*

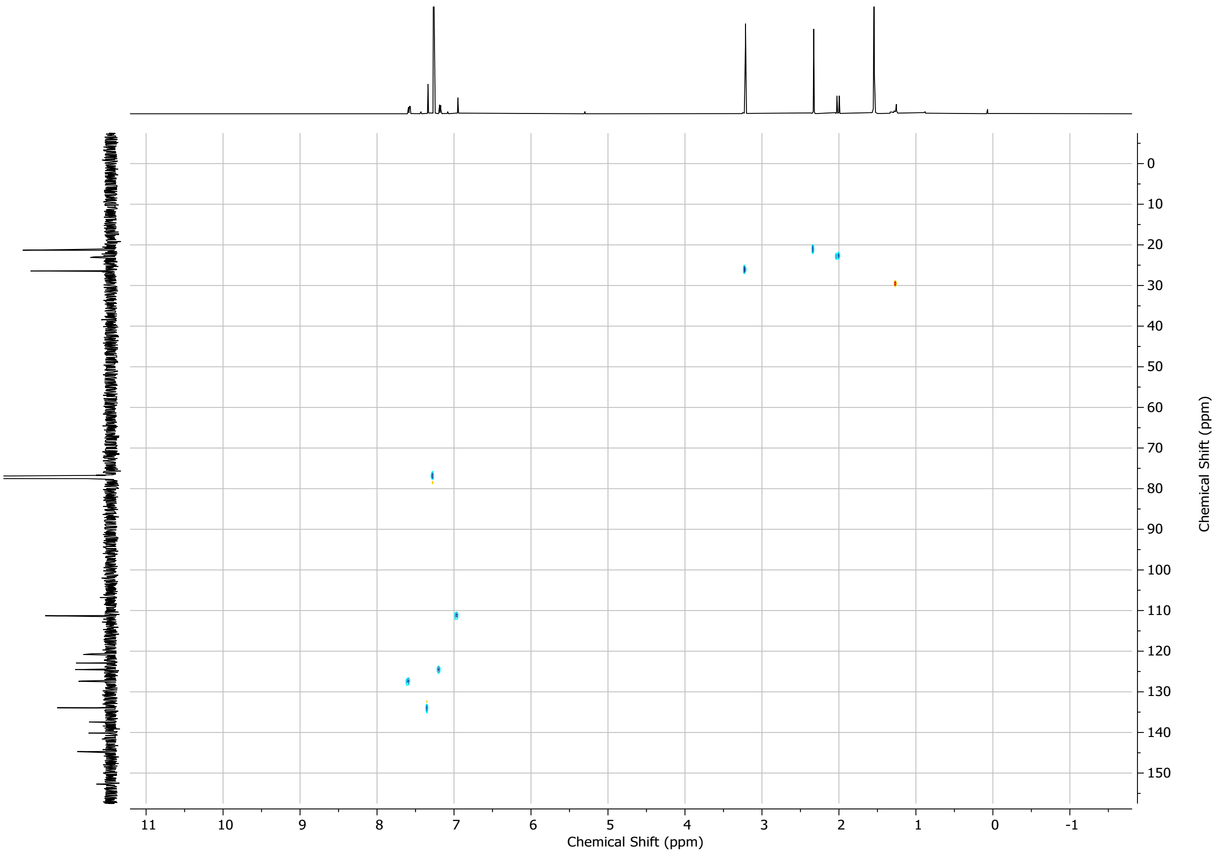

Figure S110. *HSQC NMR spectrum of (E$_S$E$_S$)-**3** (CDCl$_3$, 20 °C).*

Motor 4

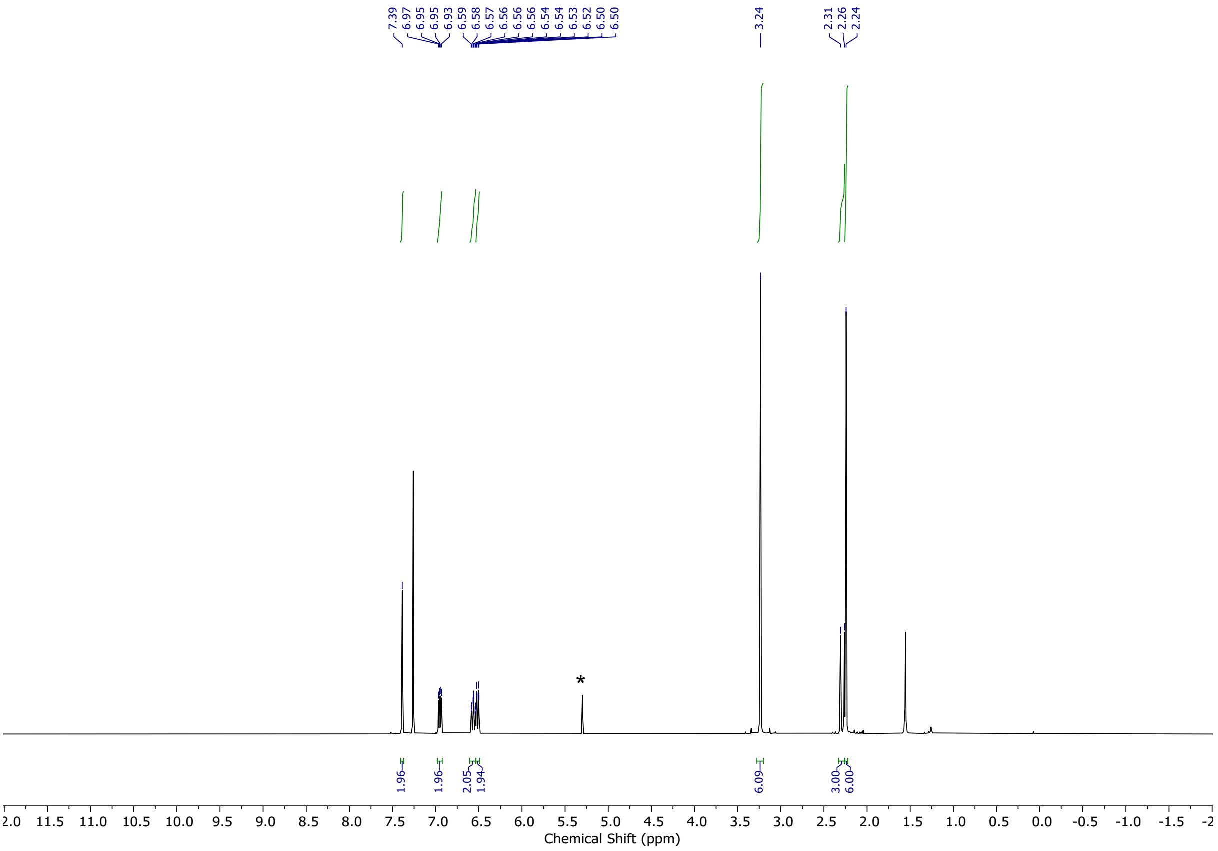

Figure S111. *^{1}H NMR spectrum of (Z$_S$Z$_S$)-**4** ($CDCl_3$, 20 °C). The peak marked with an asterisk arises from residual CH_2Cl_2.*

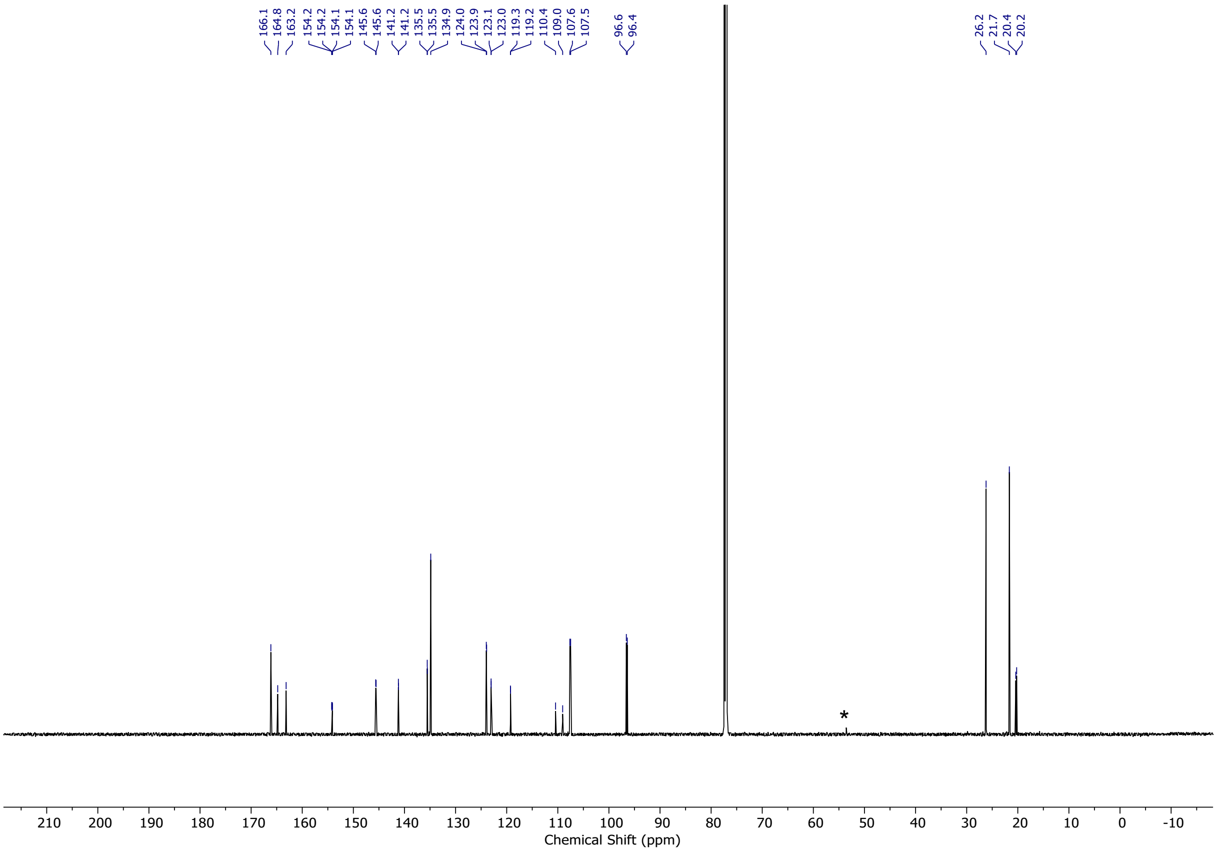

Figure S112. *^{13}C NMR spectrum of (Z$_S$Z$_S$)-**4** ($CDCl_3$, 20 °C). The peak marked with an asterisk arises from residual CH_2Cl_2.*

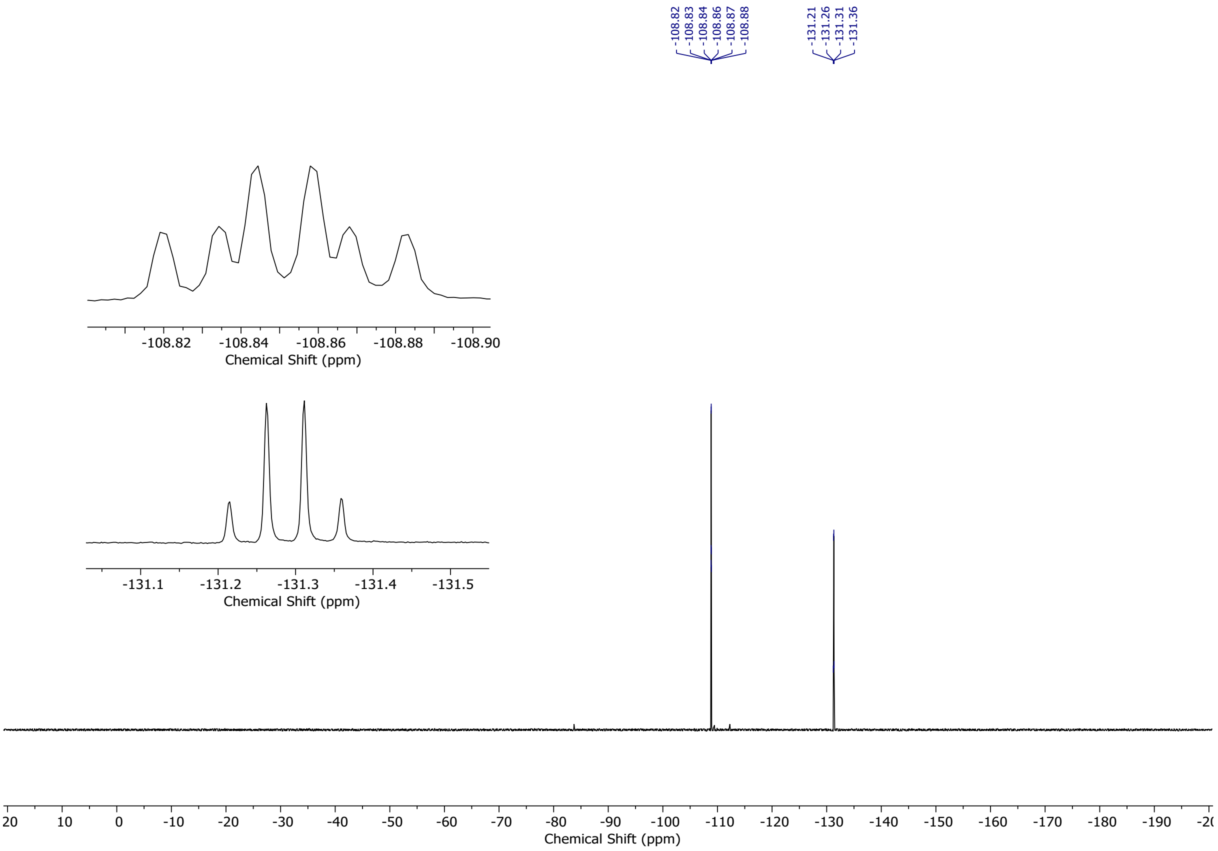

Figure S113. *^{19}F NMR spectrum of (Z$_S$Z$_S$)-**4** (CDCl$_3$, 20 °C).*

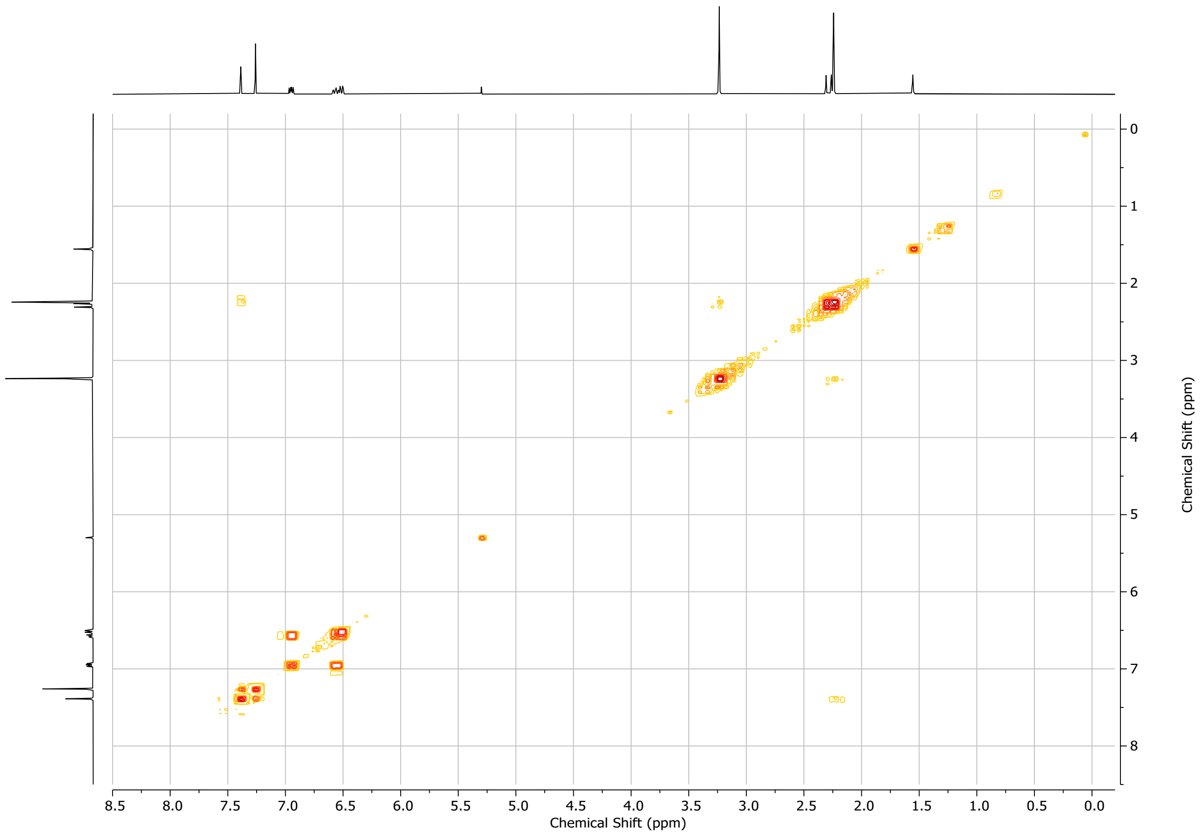

Figure S114. *COSY NMR spectrum of (Z$_S$Z$_S$)-**4** (CDCl$_3$, 20 °C).*

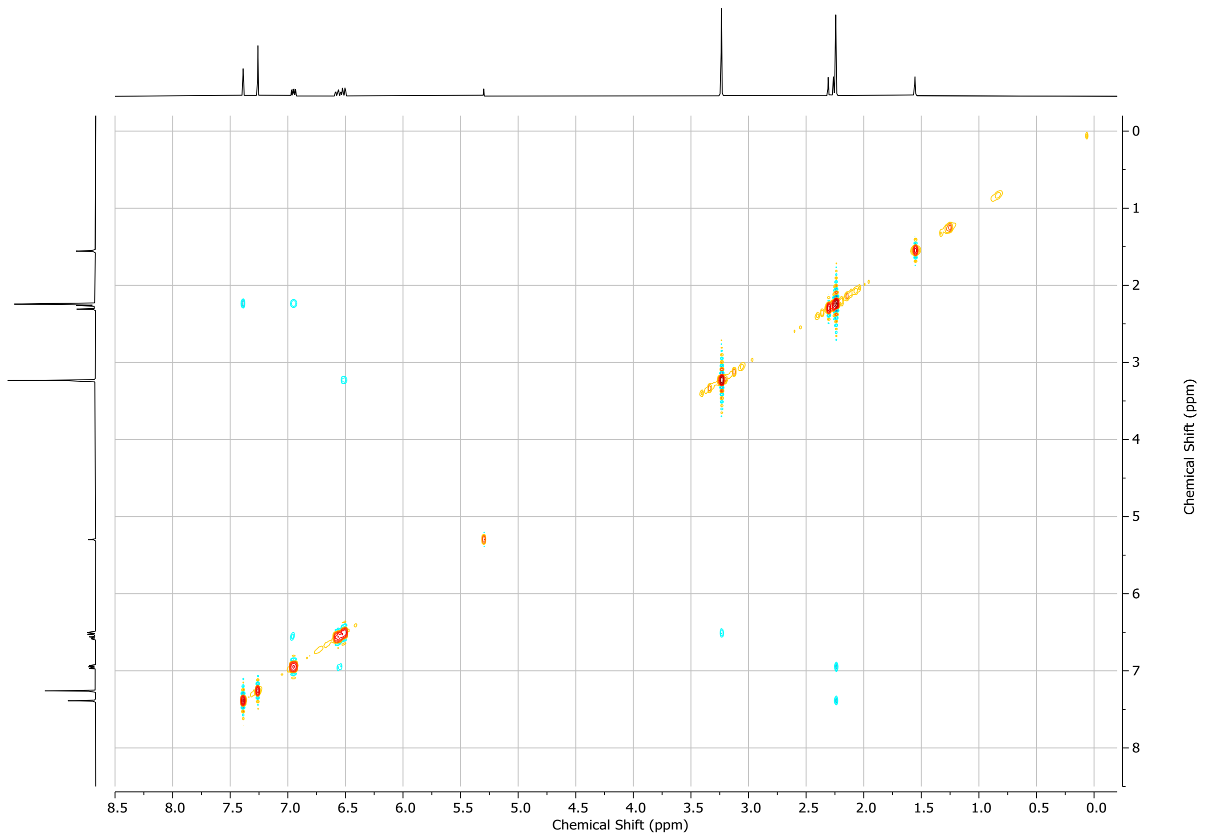

Figure S115. *NOESY NMR spectrum of (Z$_S$Z$_S$)-**4** (CDCl$_3$, 20 °C).*

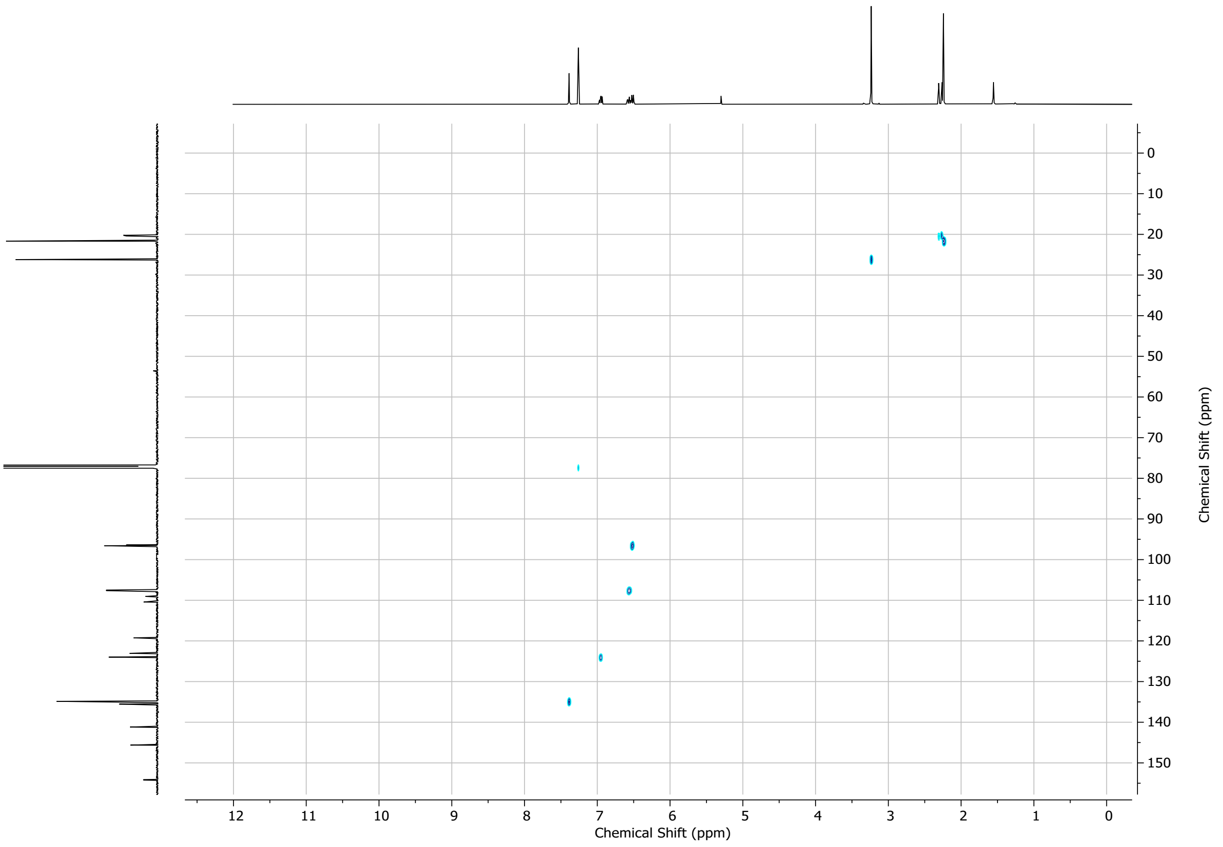

Figure S116. *HSQC NMR spectrum of (Z$_S$Z$_S$)-**4** (CDCl$_3$, 20 °C).*

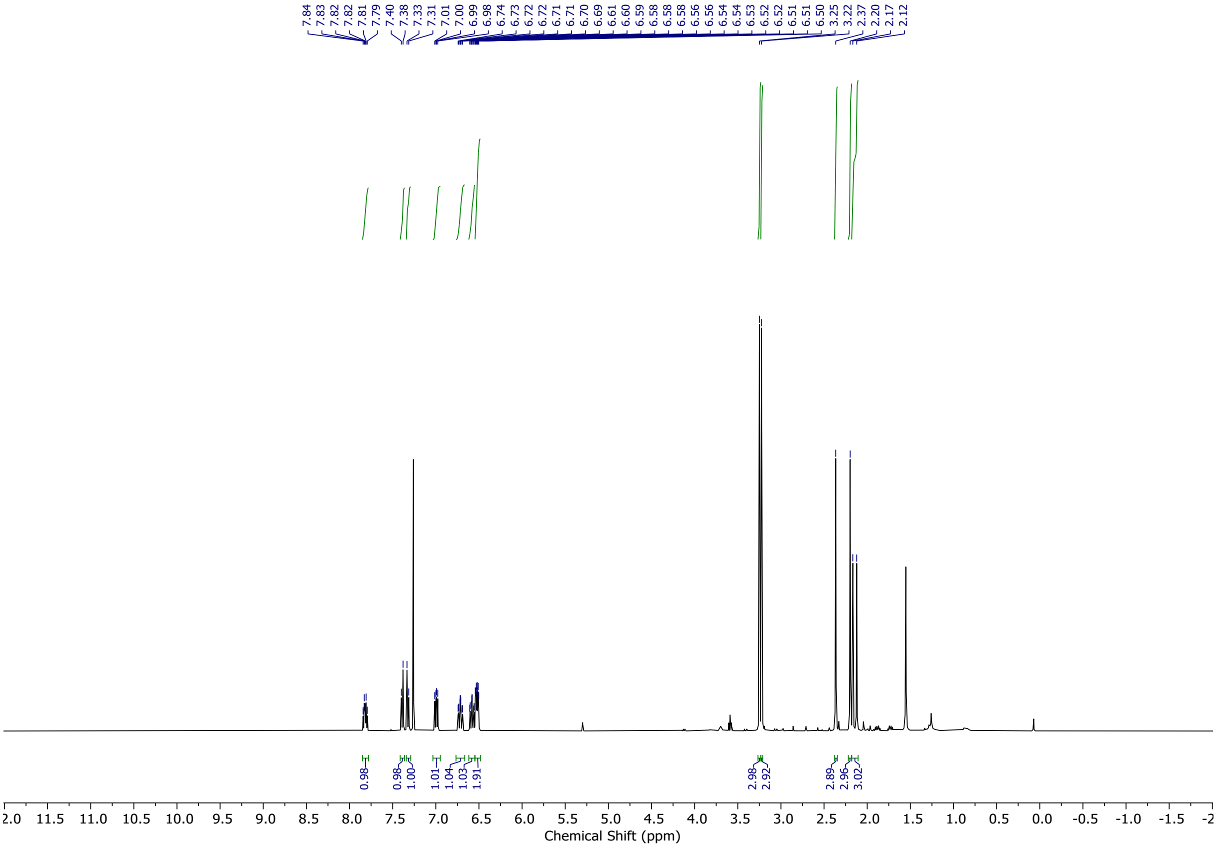

Figure S117. ^{1}H NMR spectrum of (E_SZ_S)-**4** ($CDCl_3$, 20 °C).

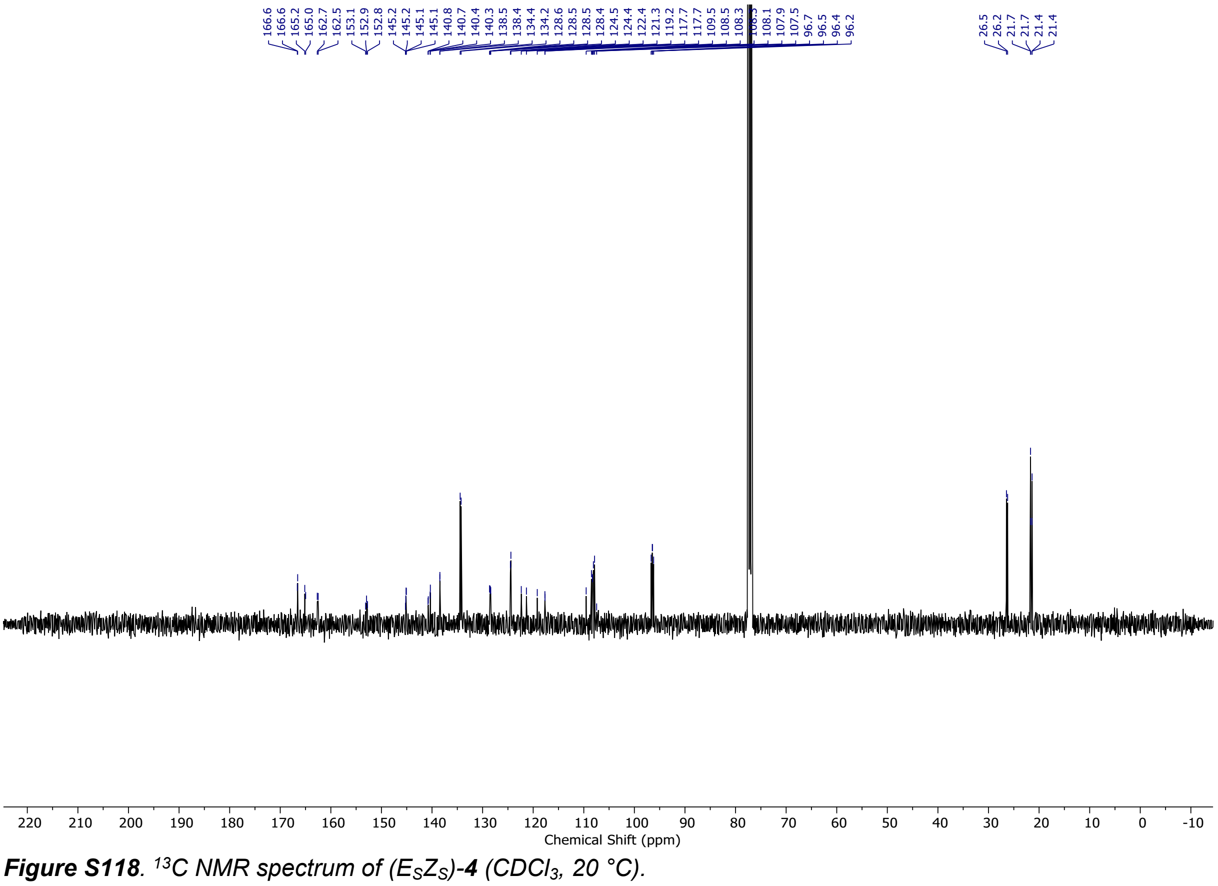

Figure S118. ^{13}C NMR spectrum of (E_SZ_S)-**4** ($CDCl_3$, 20 °C).

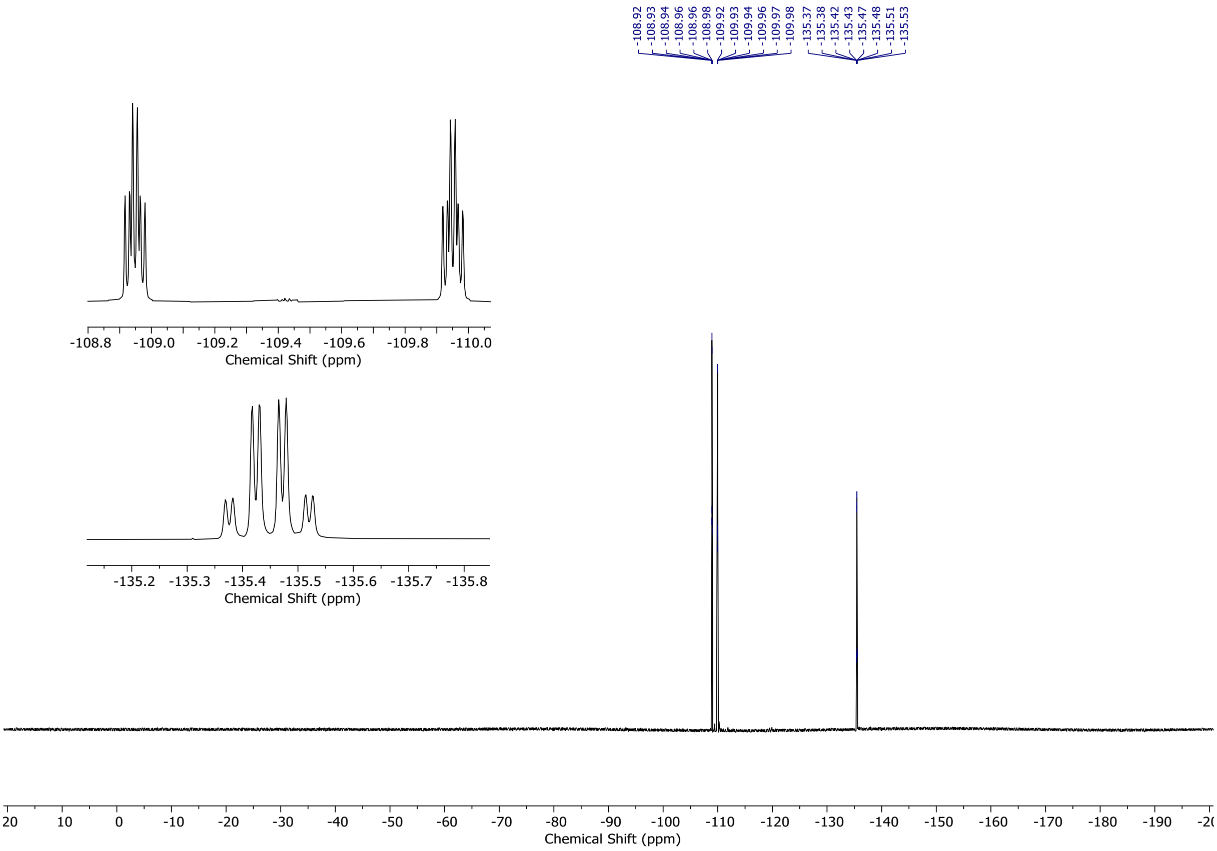

Figure S119. *^{19}F NMR spectrum of (E_SZ_S)-**4** ($CDCl_3$, 20 °C).*

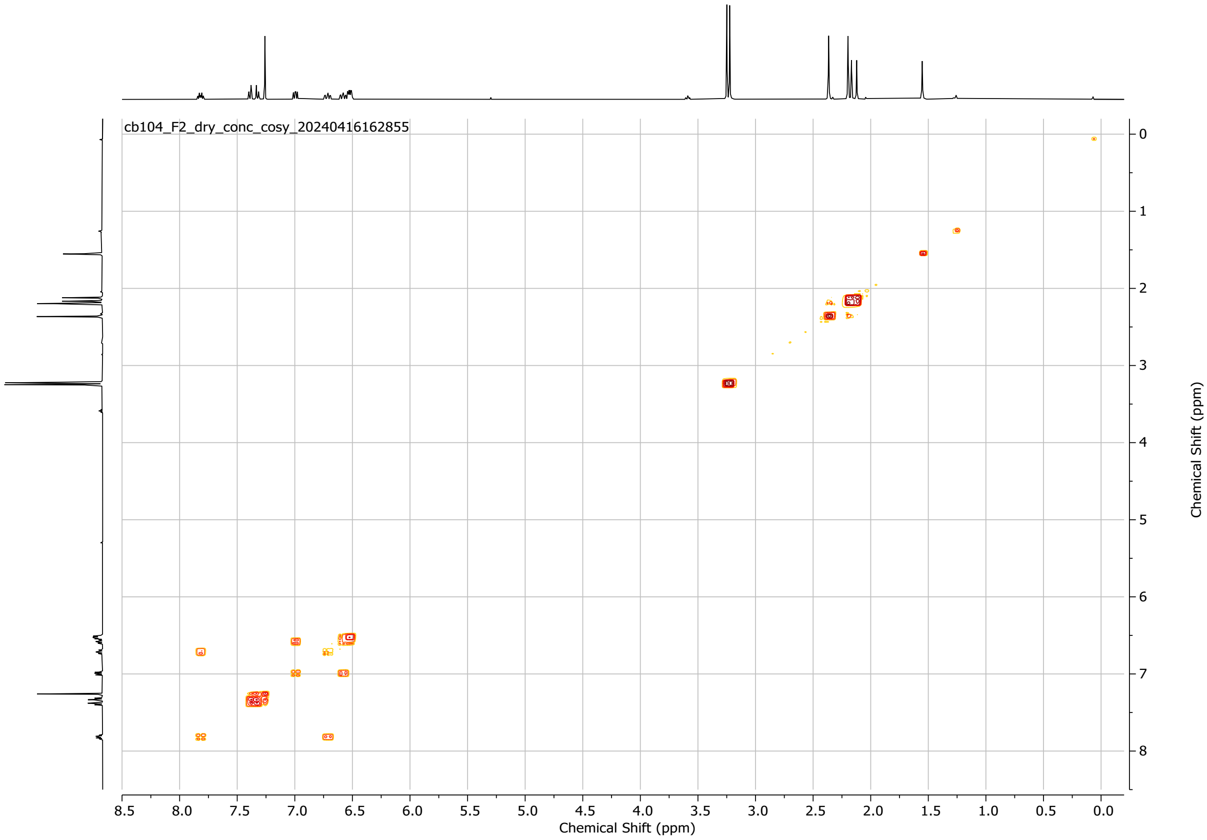

Figure S120. *COSY NMR spectrum of (E_SZ_S)-**4** ($CDCl_3$, 20 °C).*

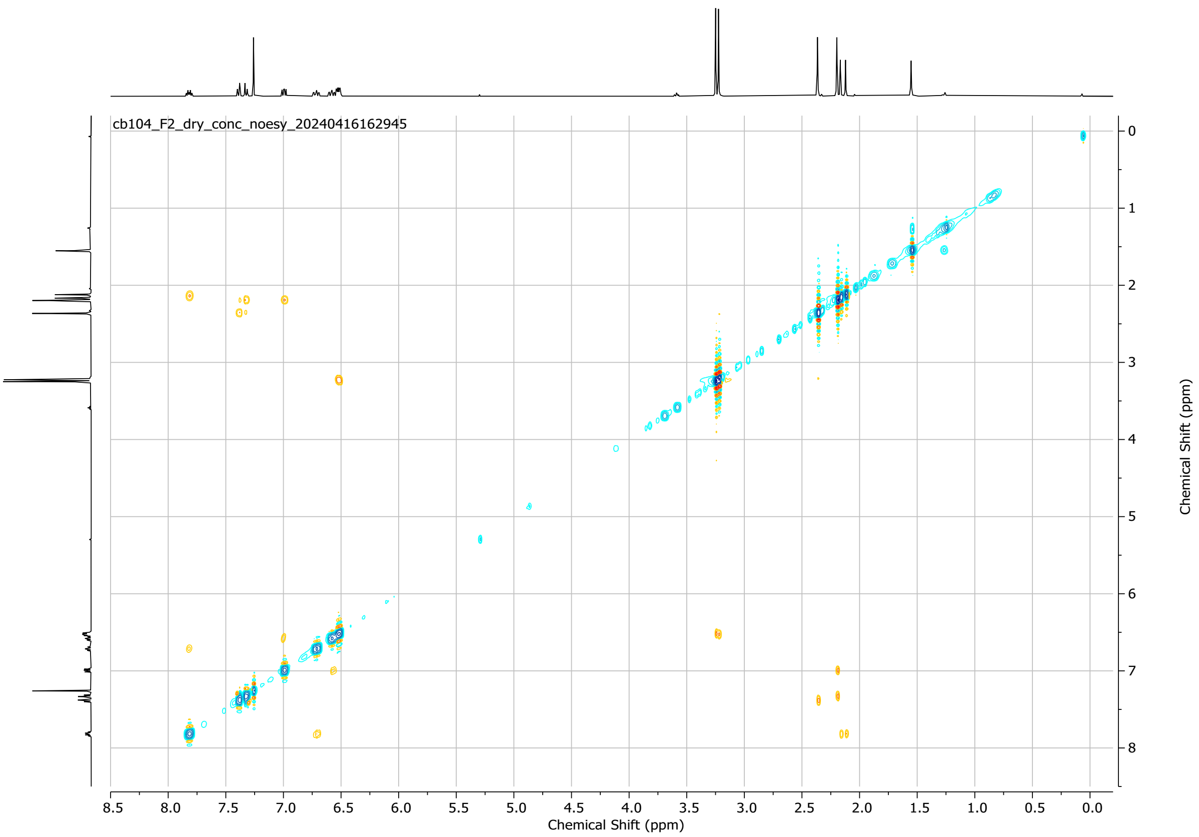

Figure S121. *NOESY NMR spectrum of (E_SZ_S)-**4** ($CDCl_3$, 20 °C).*

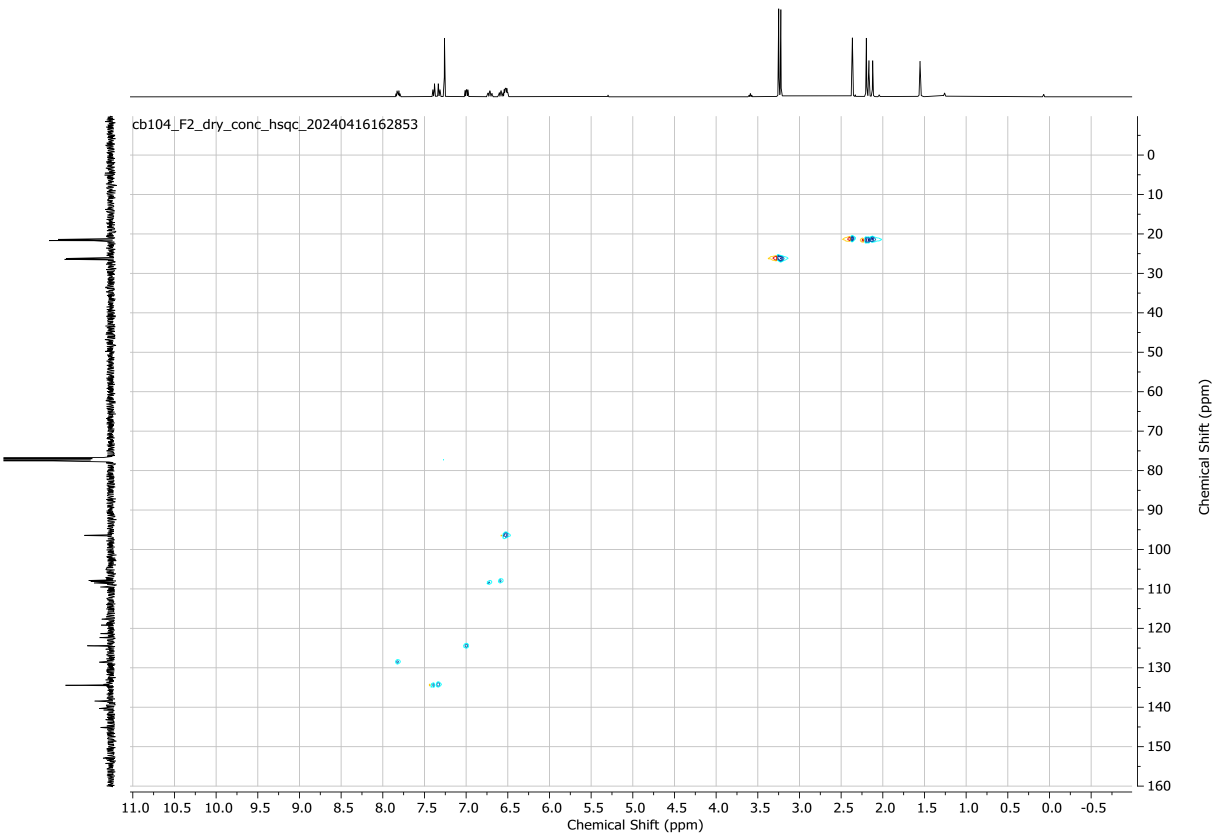

Figure S122. *HSQC NMR spectrum of (E_SZ_S)-**4** ($CDCl_3$, 20 °C).*

Motor 5

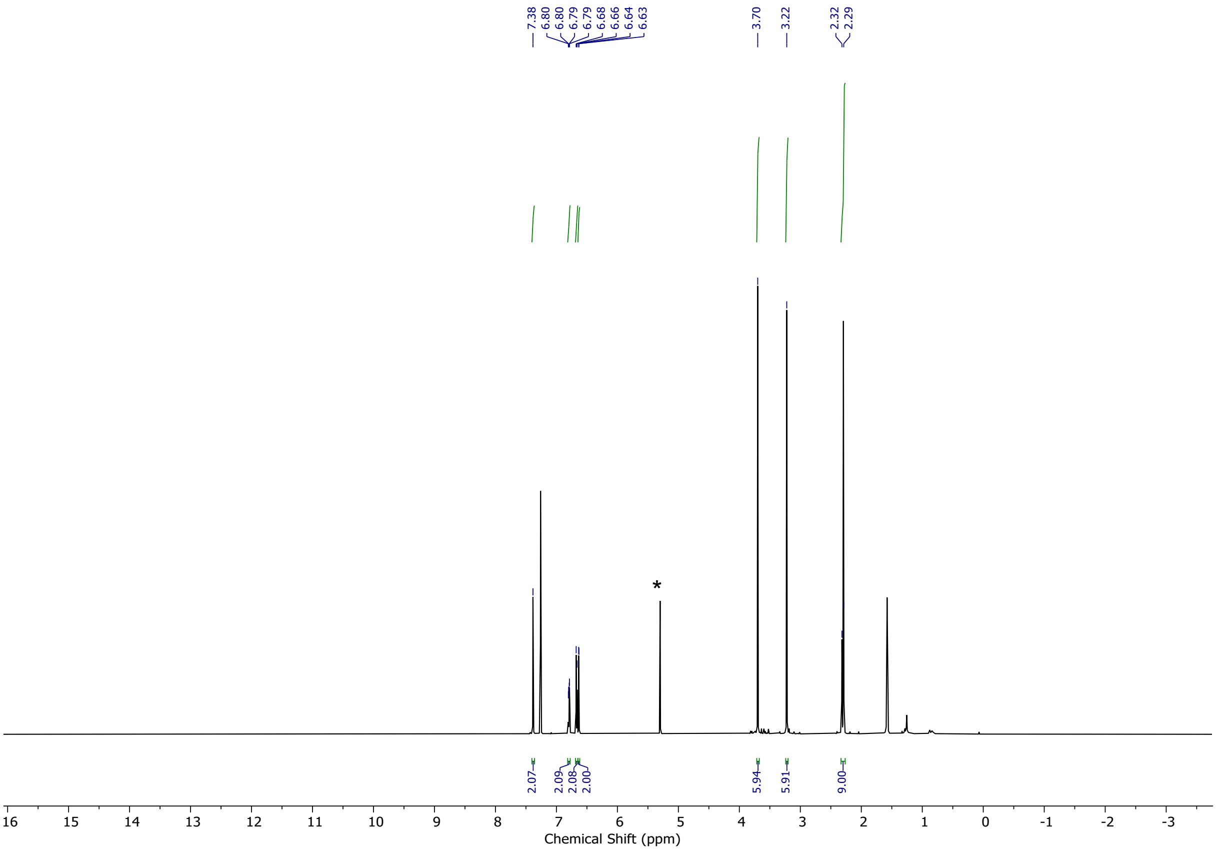

Figure S123. *^{1}H NMR spectrum of* (Z_SZ_S)*-**5** (*$CDCl_3$*, 20 °C). The peak marked with an asterisk arises from residual* CH_2Cl_2.

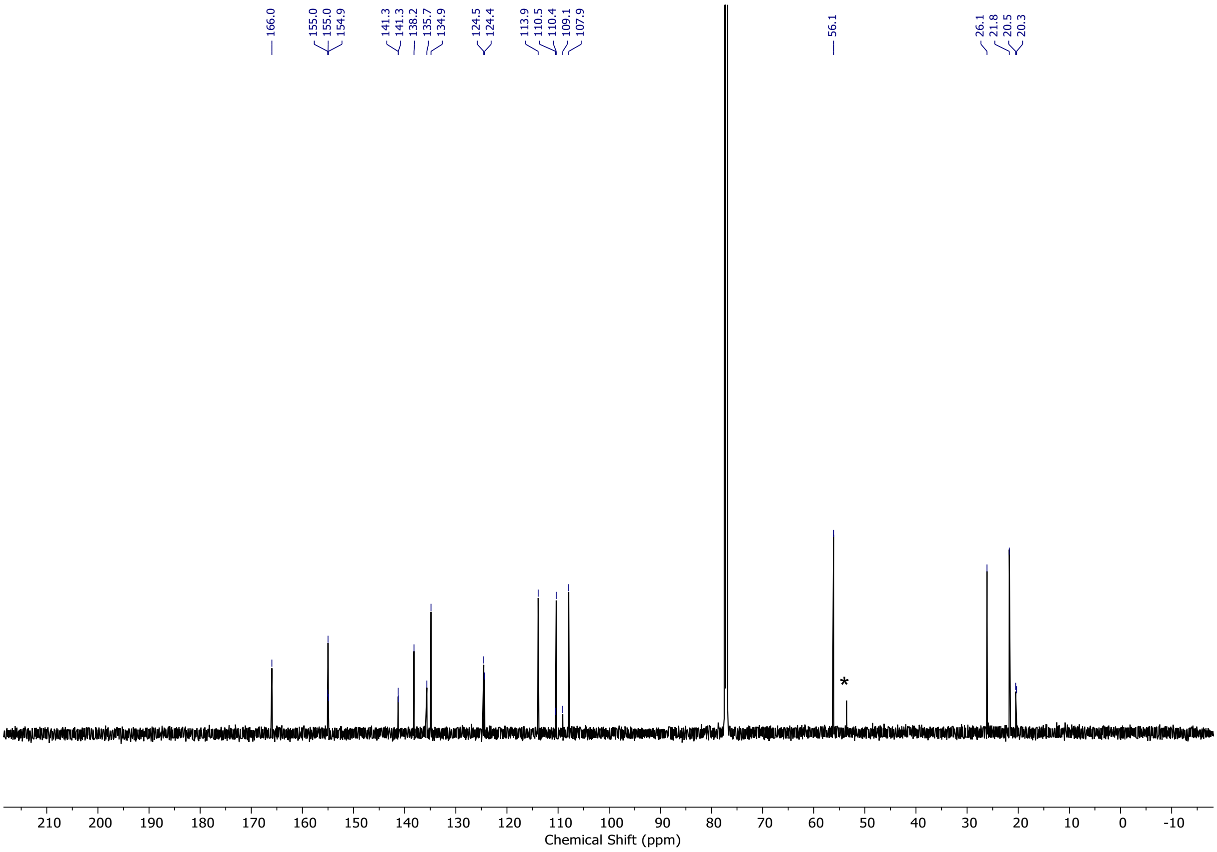

Figure S124. *^{13}C NMR spectrum of* (Z_SZ_S)*-**5** (*$CDCl_3$*, 20 °C). The peak marked with an asterisk arises from residual* CH_2Cl_2.

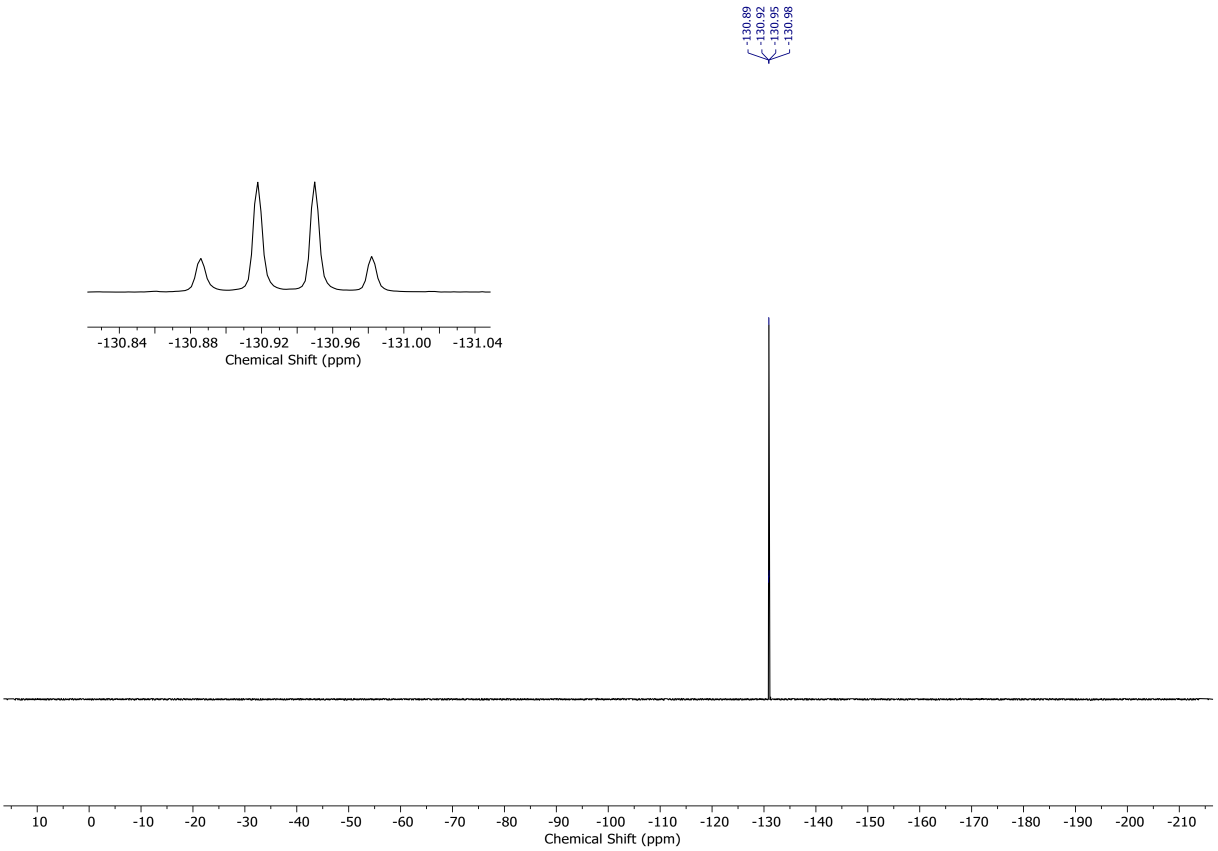

***Figure S125**. ^{19}F NMR spectrum of (Z_SZ_S)-**5** ($CDCl_3$, 20 °C).*

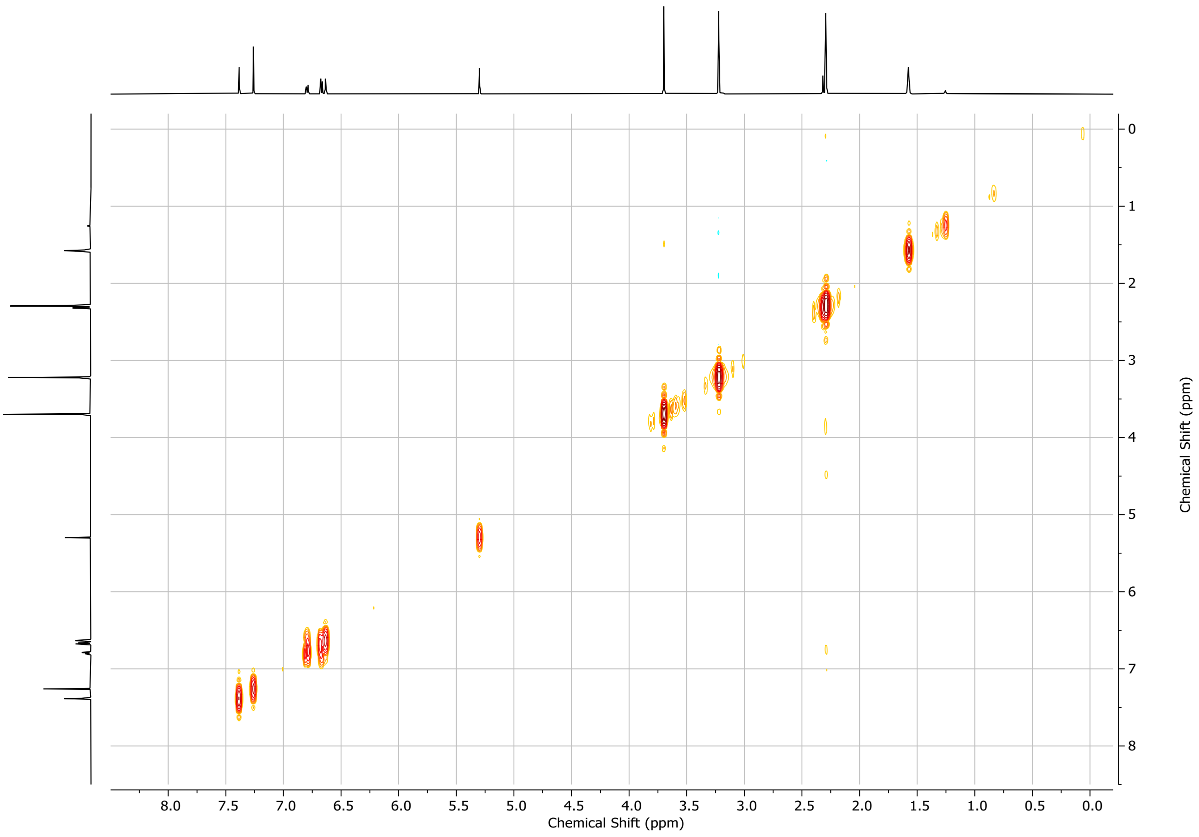

***Figure S126**. COSY NMR spectrum of (Z_SZ_S)-**5** ($CDCl_3$, 20 °C).*

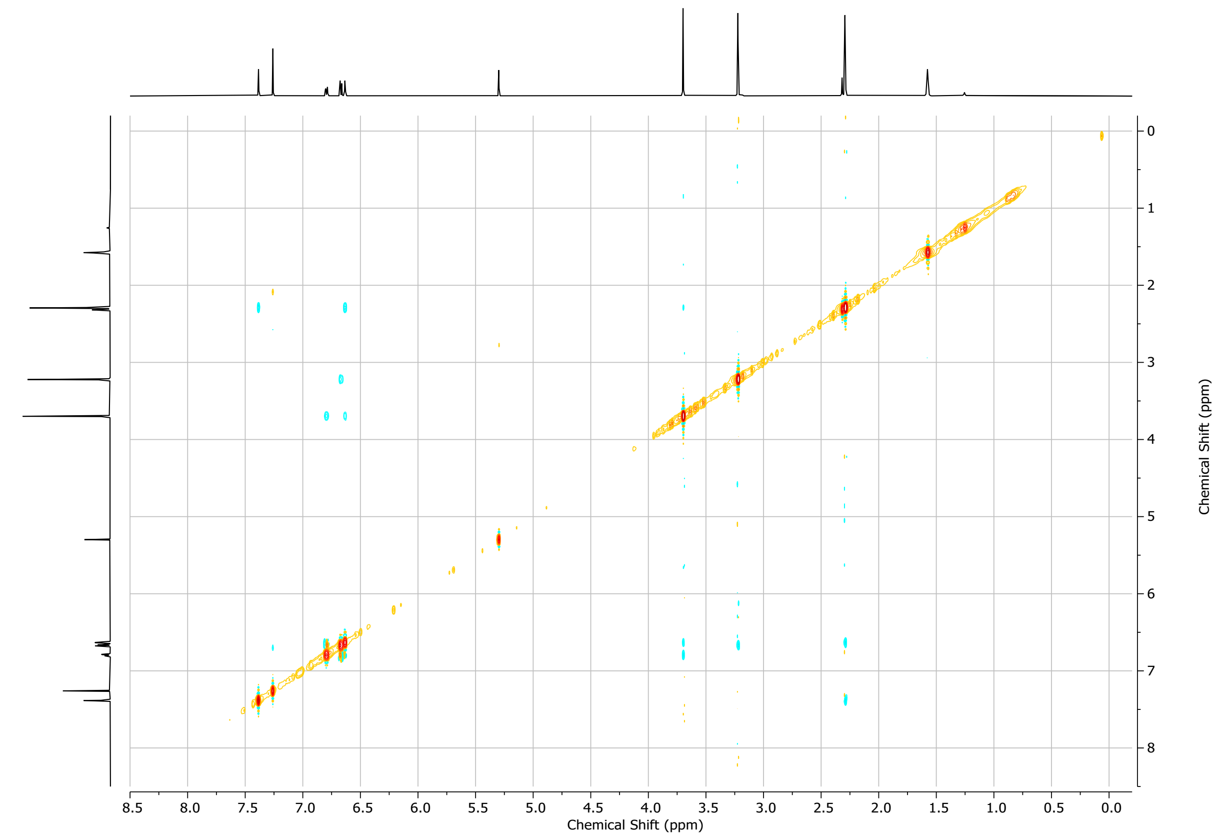

Figure S127. *NOESY NMR spectrum of (Z_SZ_S)-**5** ($CDCl_3$, 20 °C).*

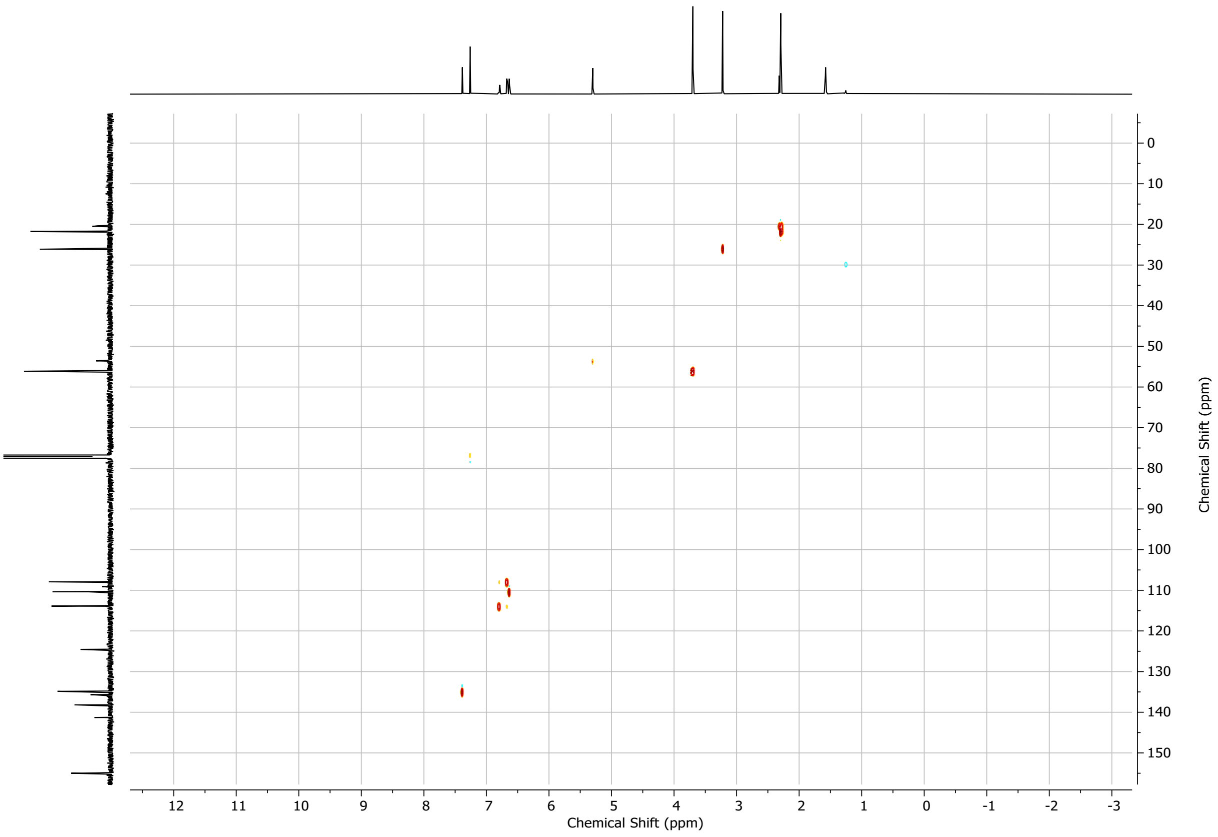

Figure S128. *HSQC NMR spectrum of (Z_SZ_S)-**5** ($CDCl_3$, 20 °C).*

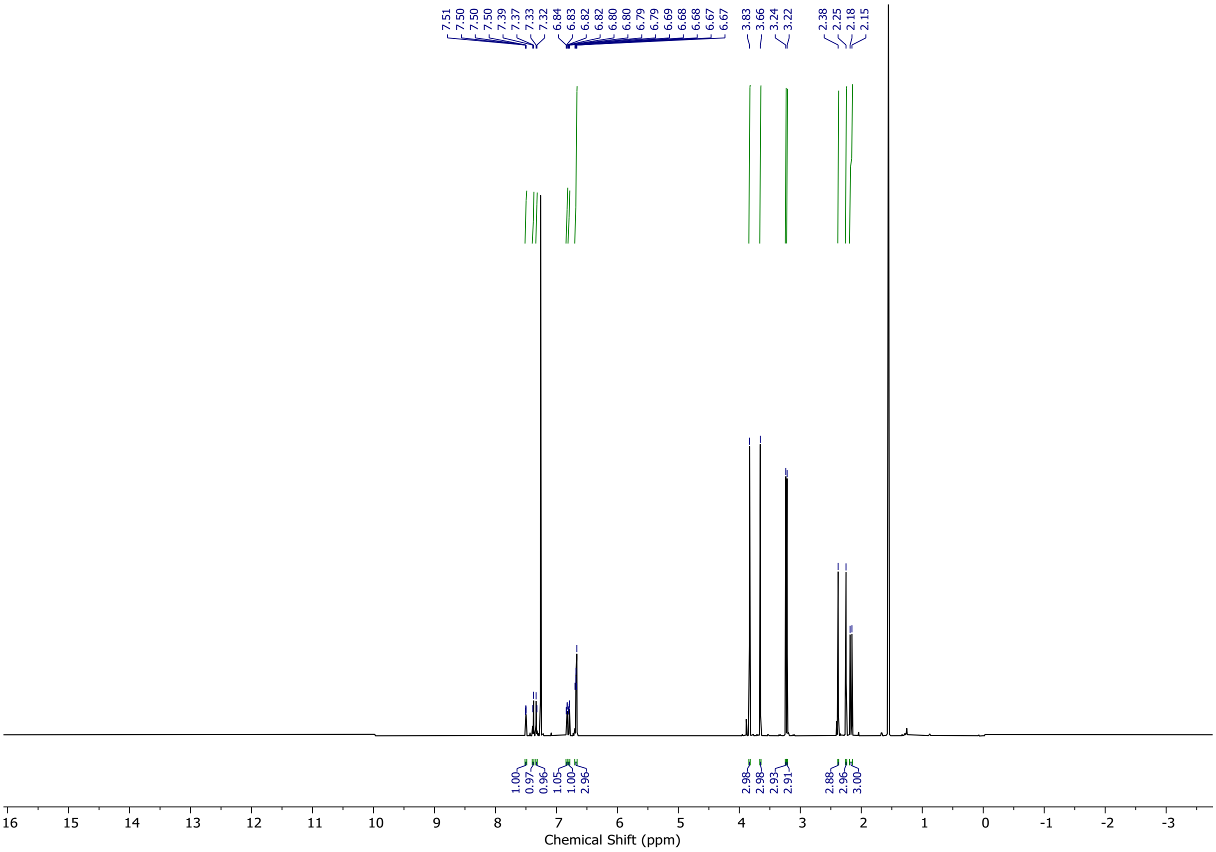

Figure S129. *^{1}H NMR spectrum of (E_SZ_S)-**5** ($CDCl_3$, 20 °C).*

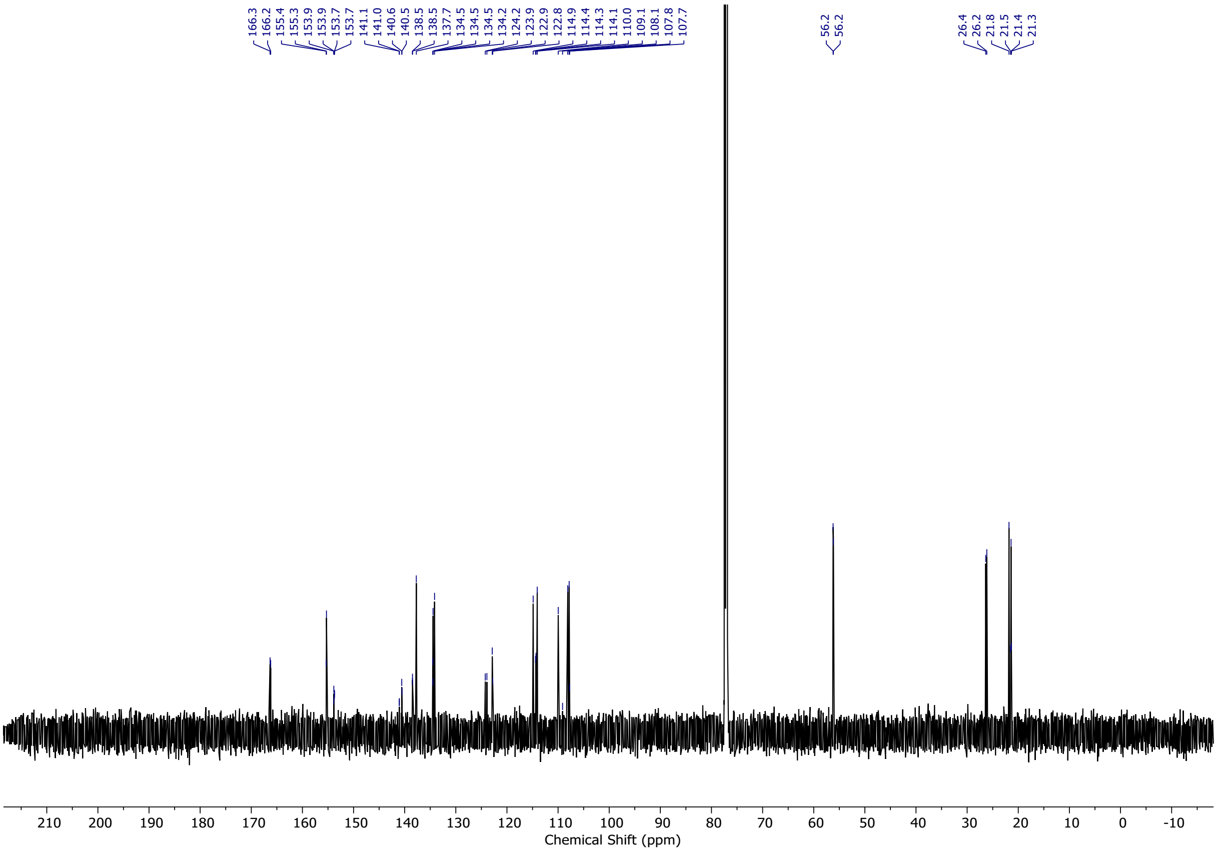

Figure S130. *^{13}C NMR spectrum of (E_SZ_S)-**5** ($CDCl_3$, 20 °C).*

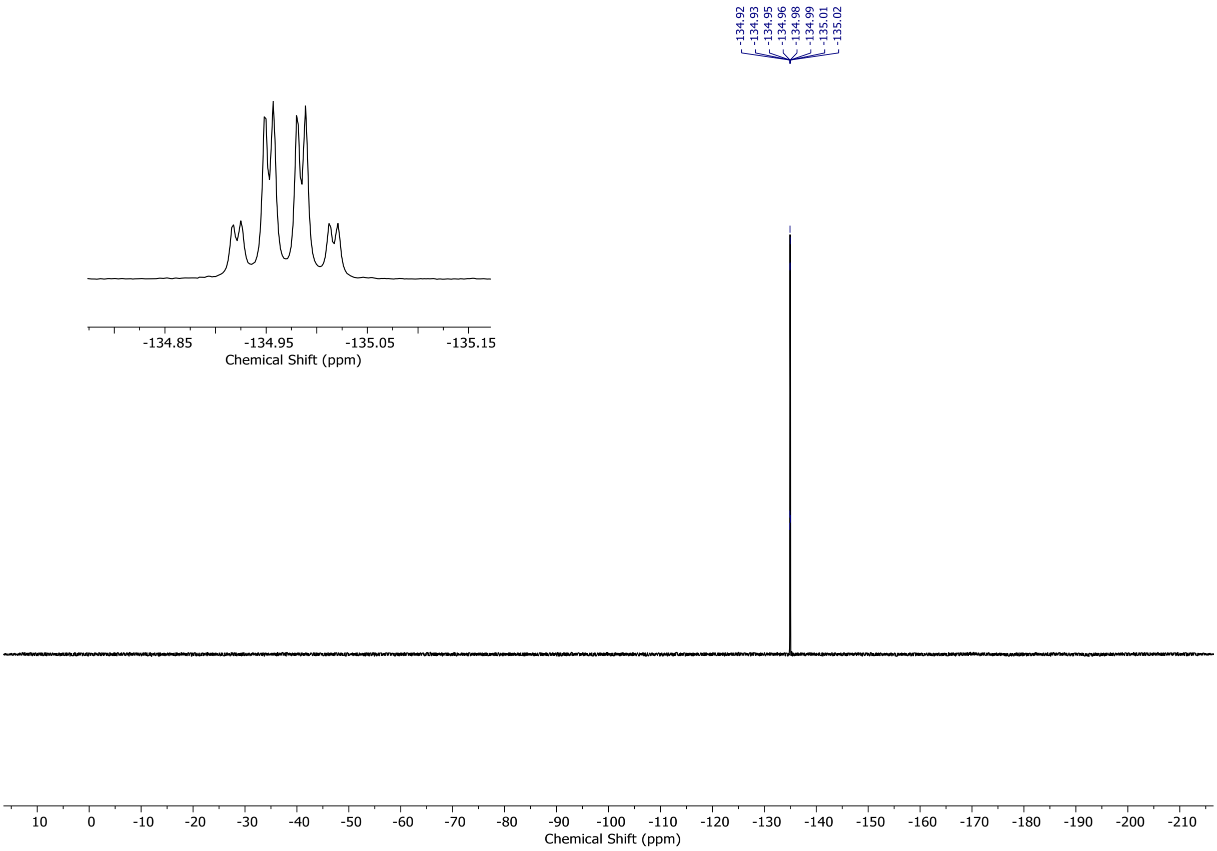

Figure S131. *^{19}F NMR spectrum of (E_SZ_S)-**5** ($CDCl_3$, 20 °C).*

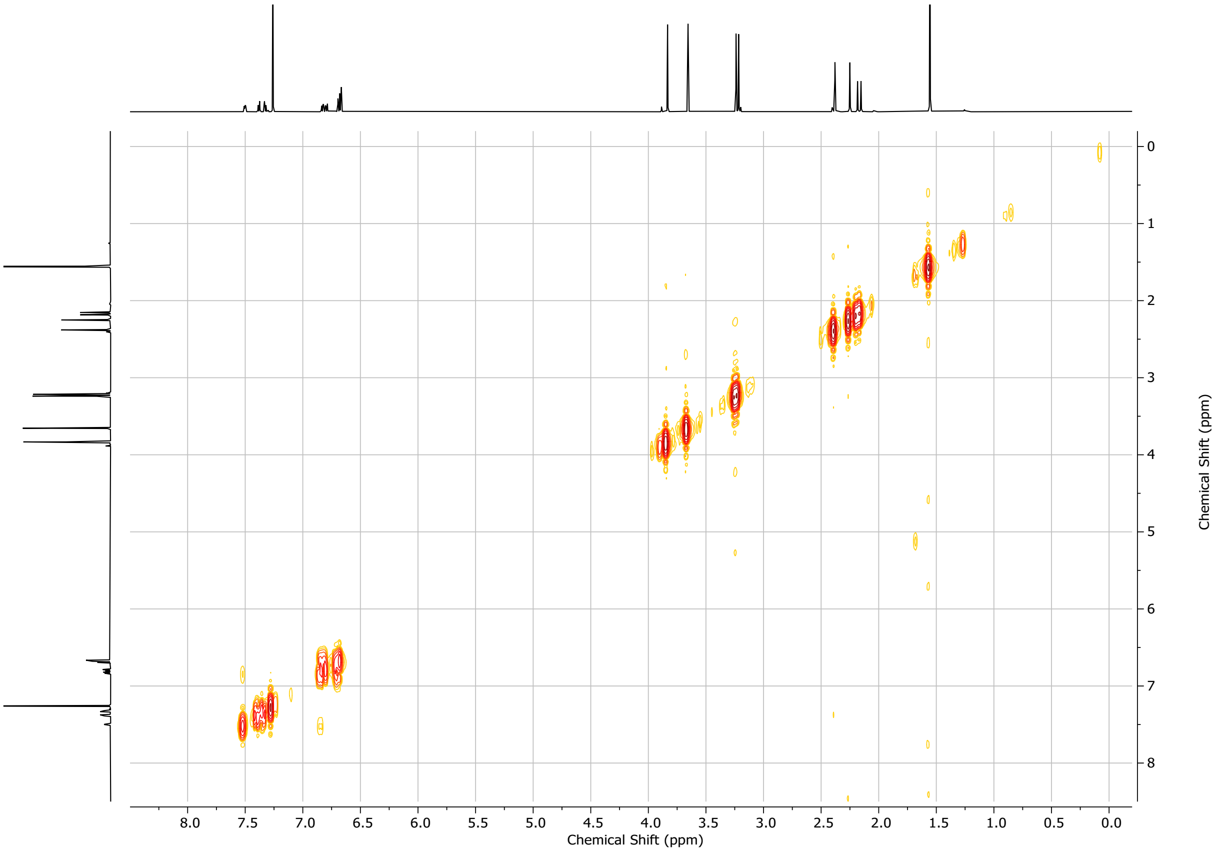

Figure S132. *COSY NMR spectrum of (E_SZ_S)-**5** ($CDCl_3$, 20 °C).*

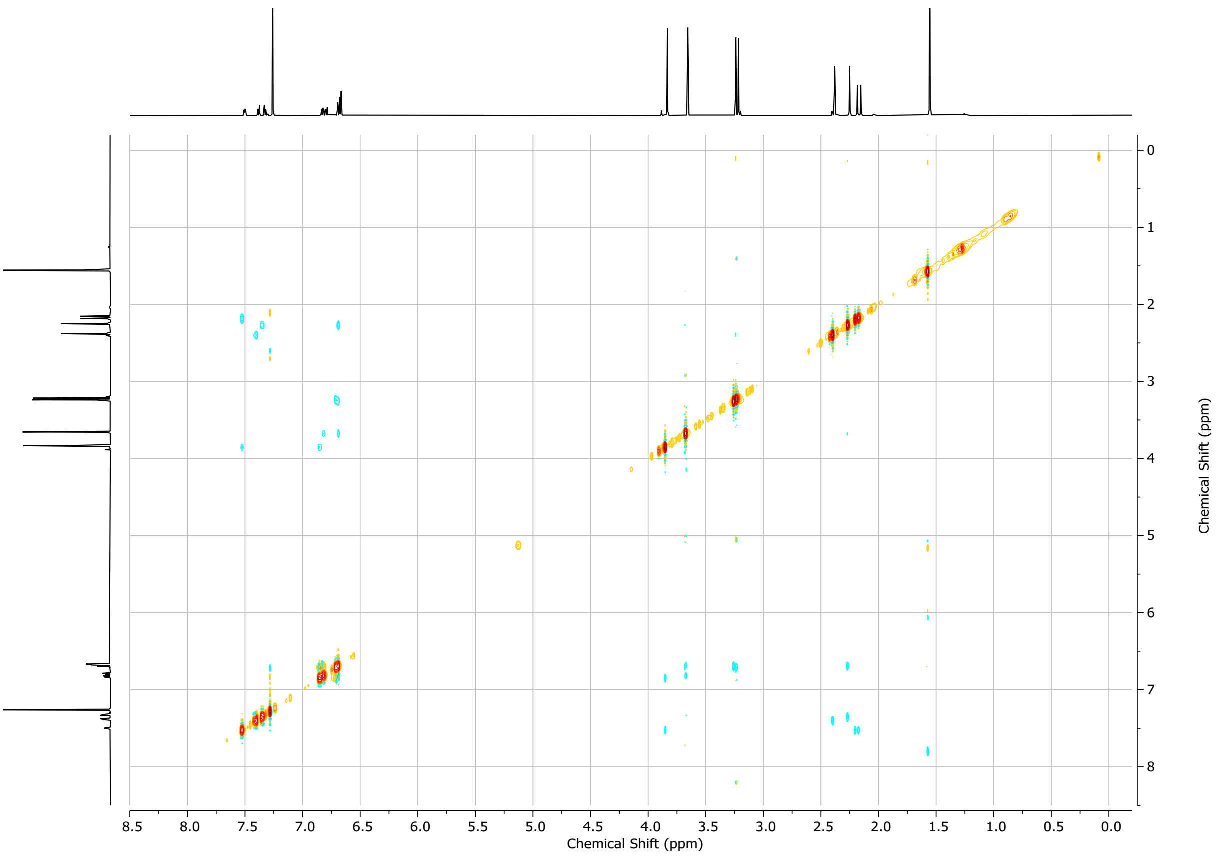

Figure S133. *NOESY NMR spectrum of (E_SZ_S)-**5** ($CDCl_3$, 20 °C).*

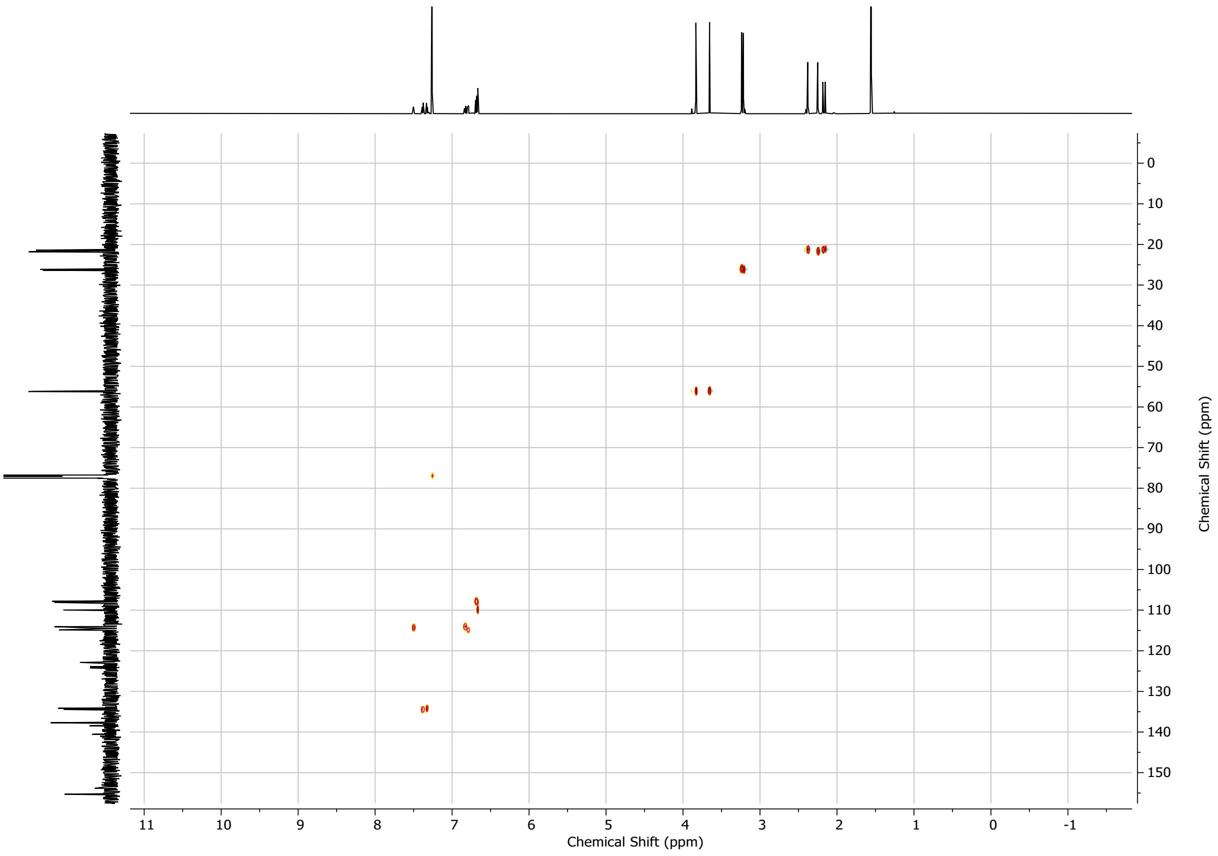

Figure S134. *HSQC NMR spectrum of (E_SZ_S)-**5** ($CDCl_3$, 20 °C).*

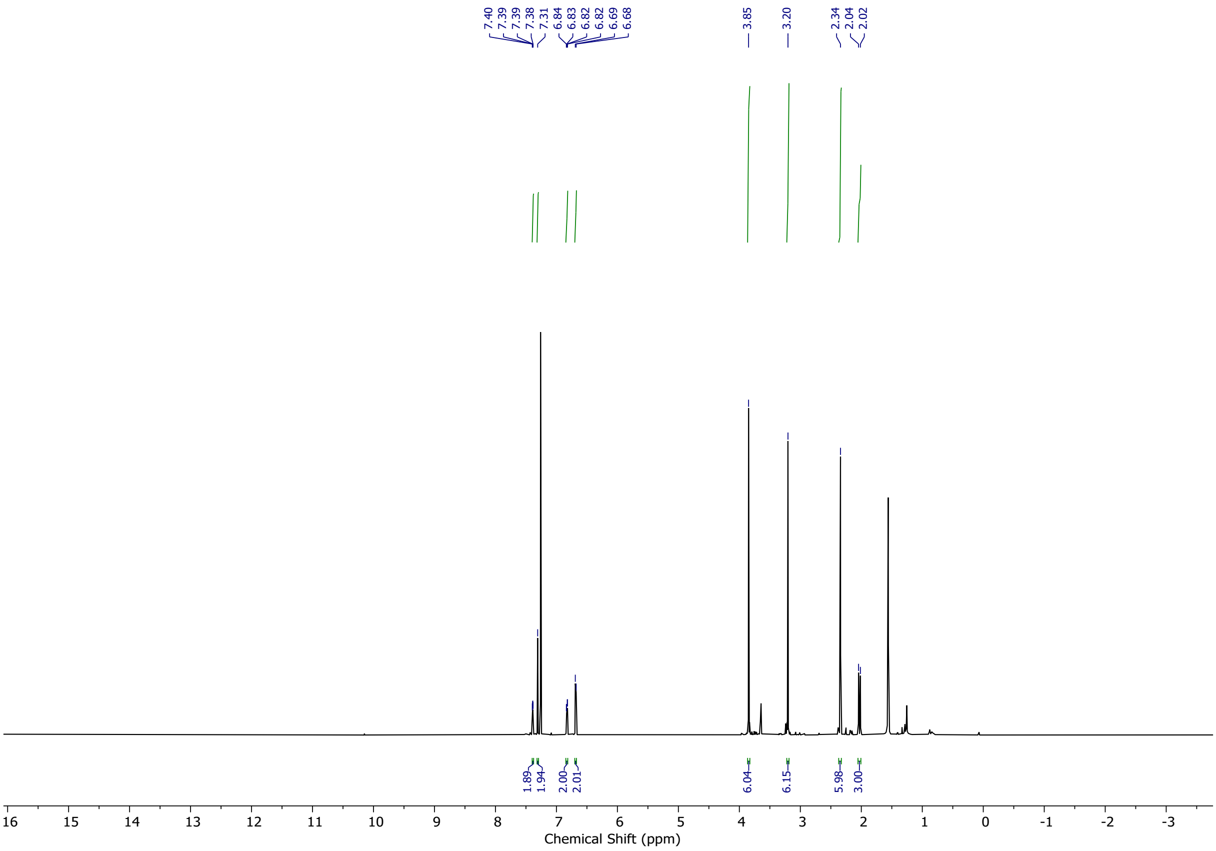

Figure S135. *^{1}H NMR spectrum of (E_SE_S)-**5** ($CDCl_3$, 20 °C).*

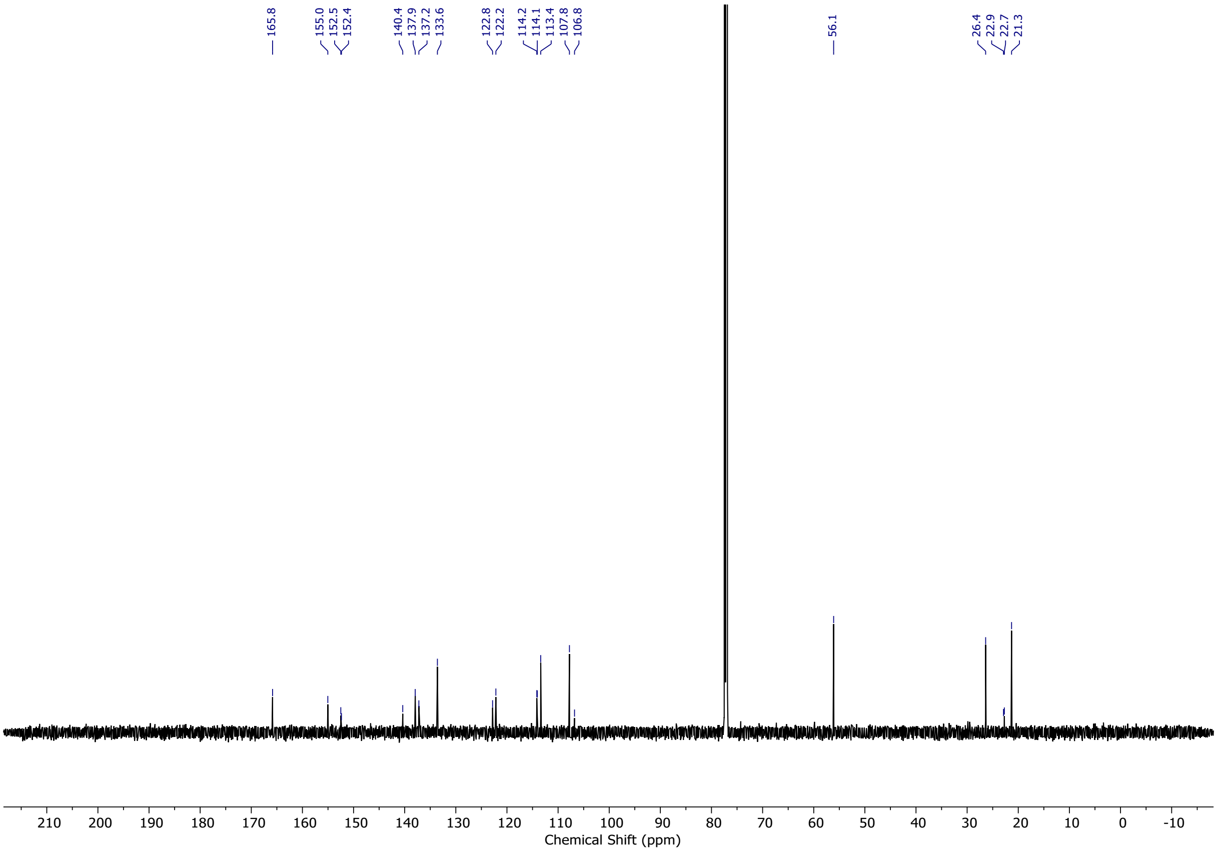

Figure S136. *^{13}C NMR spectrum of (E_SE_S)-**5** ($CDCl_3$, 20 °C).*

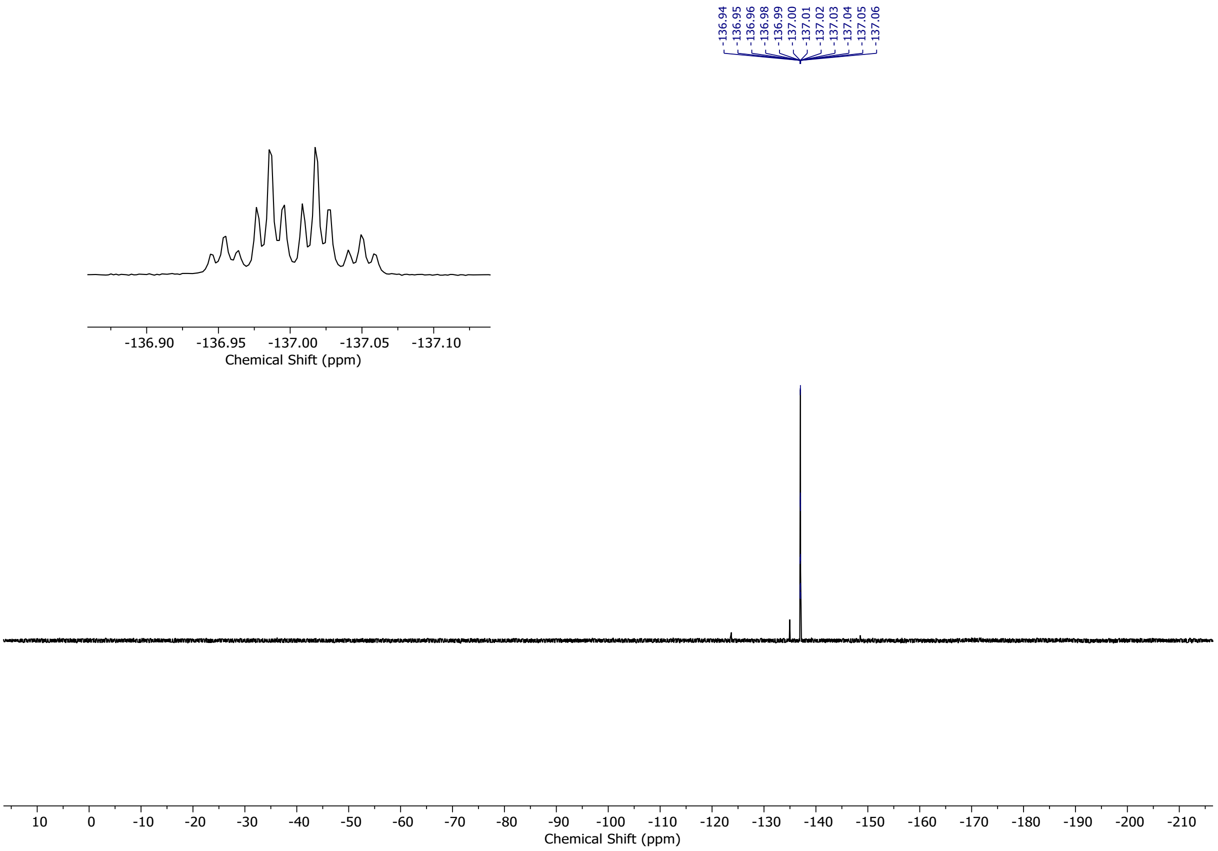

Figure S137. *^{19}F NMR spectrum of (E_SE_S)-**5** ($CDCl_3$, 20 °C).*

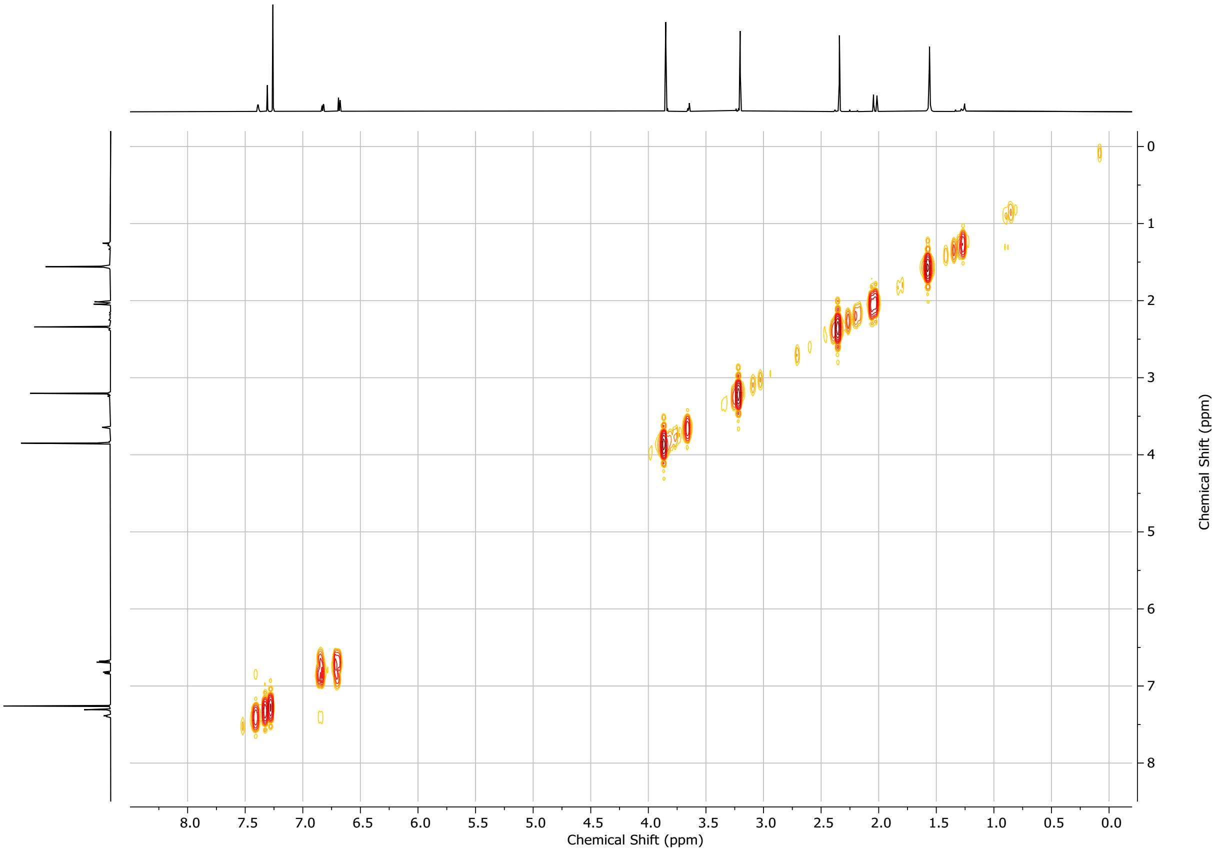

Figure S138. *COSY NMR spectrum of (E_SE_S)-**5** ($CDCl_3$, 20 °C).*

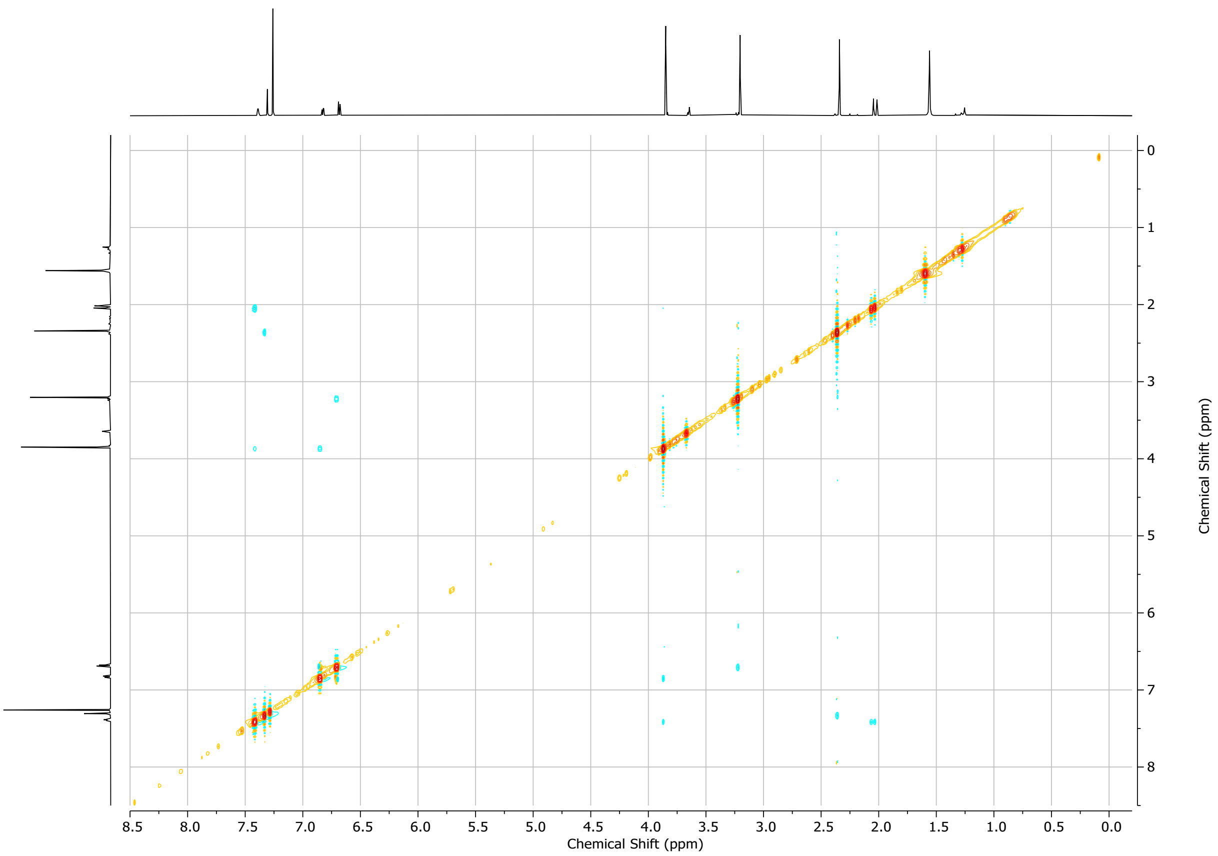

Figure S139. *NOESY NMR spectrum of (E_SE_S)-**5** ($CDCl_3$, 20 °C).*

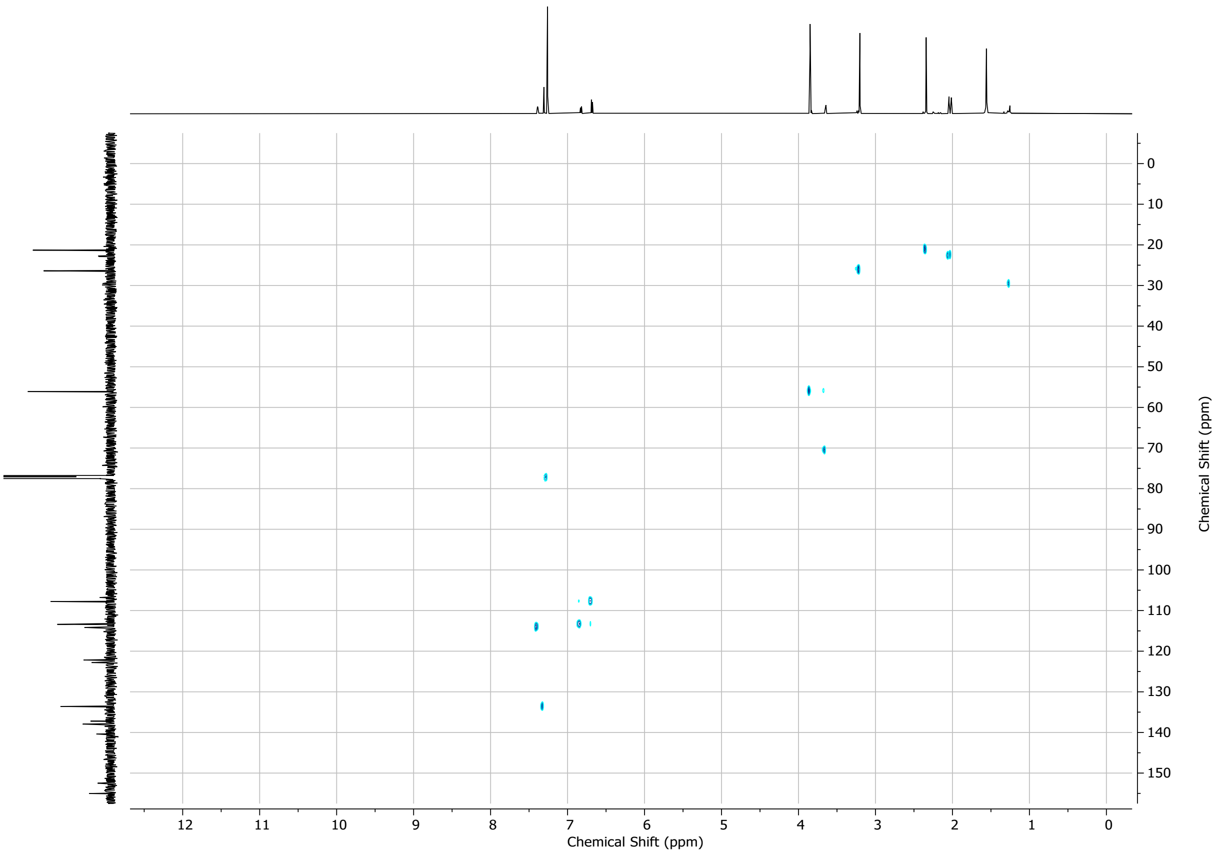

Figure S140. *HSQC NMR spectrum of (E_SE_S)-**5** ($CDCl_3$, 20 °C).*

Motor 6

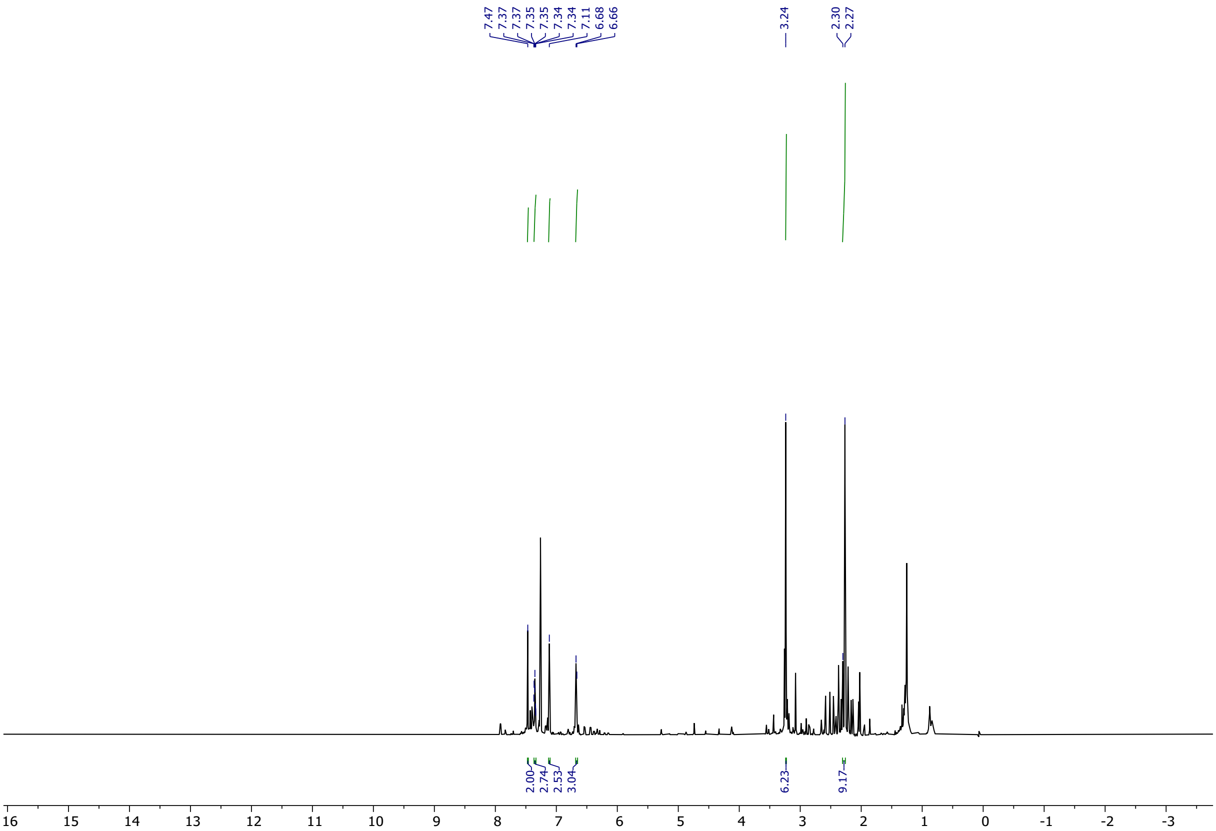

Figure S141. *^{1}H NMR spectrum of a mixture of (Z_SZ_S)-**6** (64%), (E_SZ_S)-**6** (29%) and (E_SE_S)-**6** (7%) ($CDCl_3$, 20 °C).*

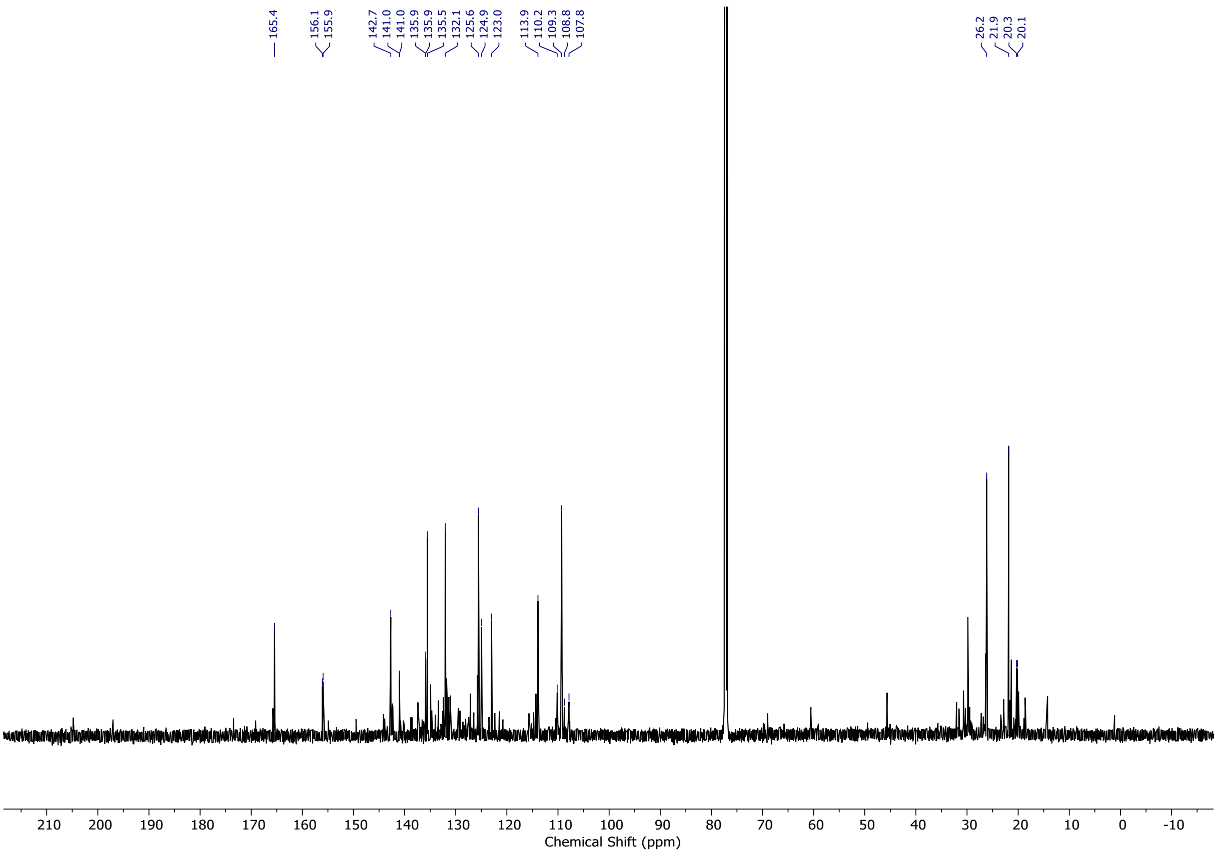

Figure S142. *^{13}C NMR spectrum of a mixture of (Z_SZ_S)-**6** (64%), (E_SZ_S)-**6** (29%) and (E_SE_S)-**6** (7%) ($CDCl_3$, 20 °C).*

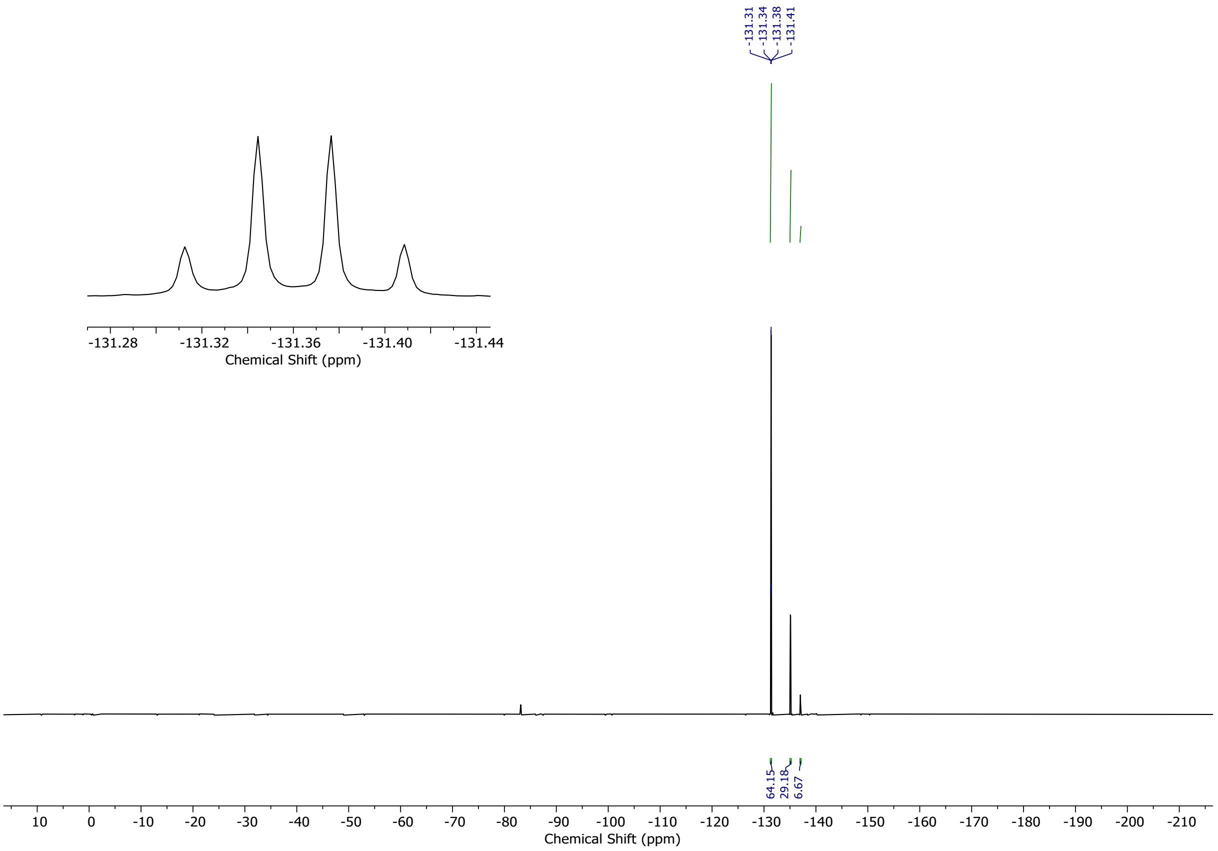

Figure S143. *^{19}F NMR spectrum of a mixture of (Z_SZ_S)-**6** (64%), (E_SZ_S)-**6** (29%) and (E_SE_S)-**6** (7%) ($CDCl_3$, 20 °C).*

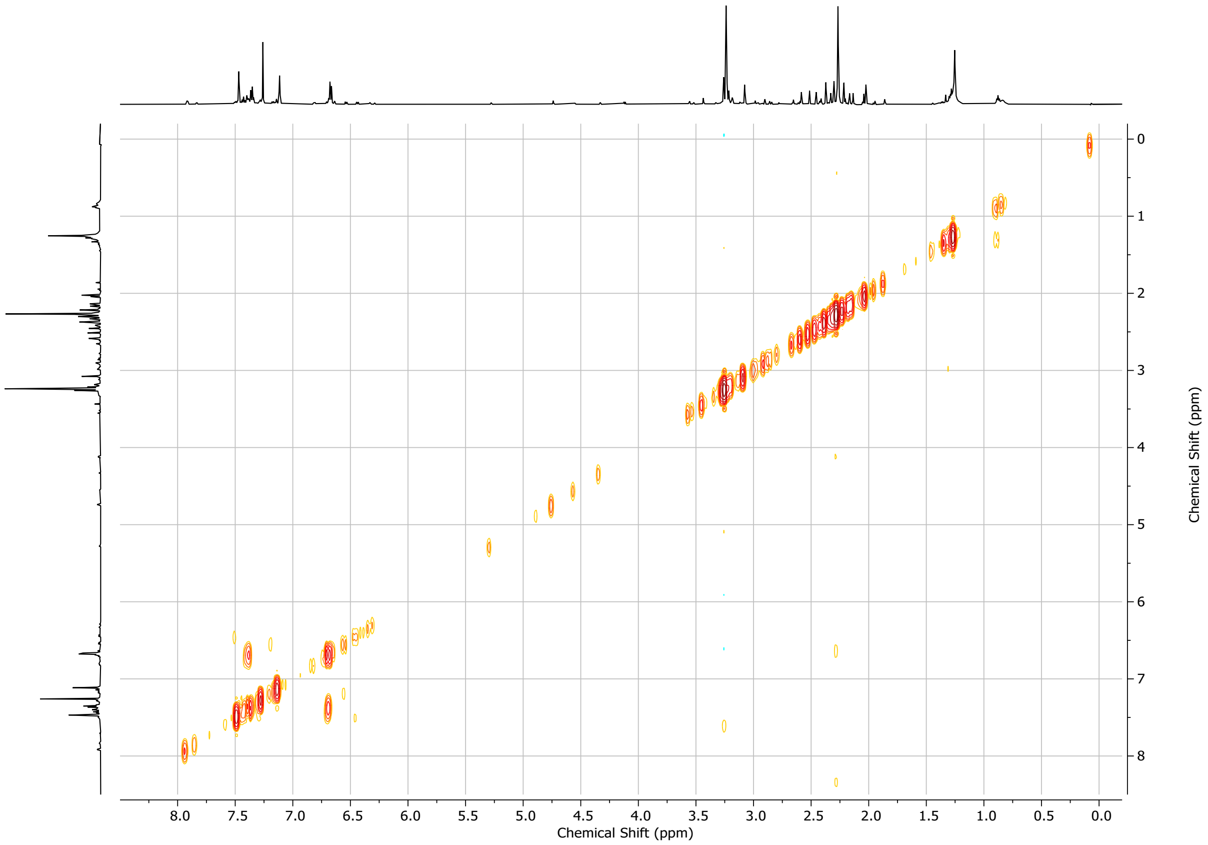

Figure S144. *COSY NMR spectrum of a mixture of (Z_SZ_S)-**6** (64%), (E_SZ_S)-**6** (29%) and (E_SE_S)-**6** (7%) ($CDCl_3$, 20 °C).*

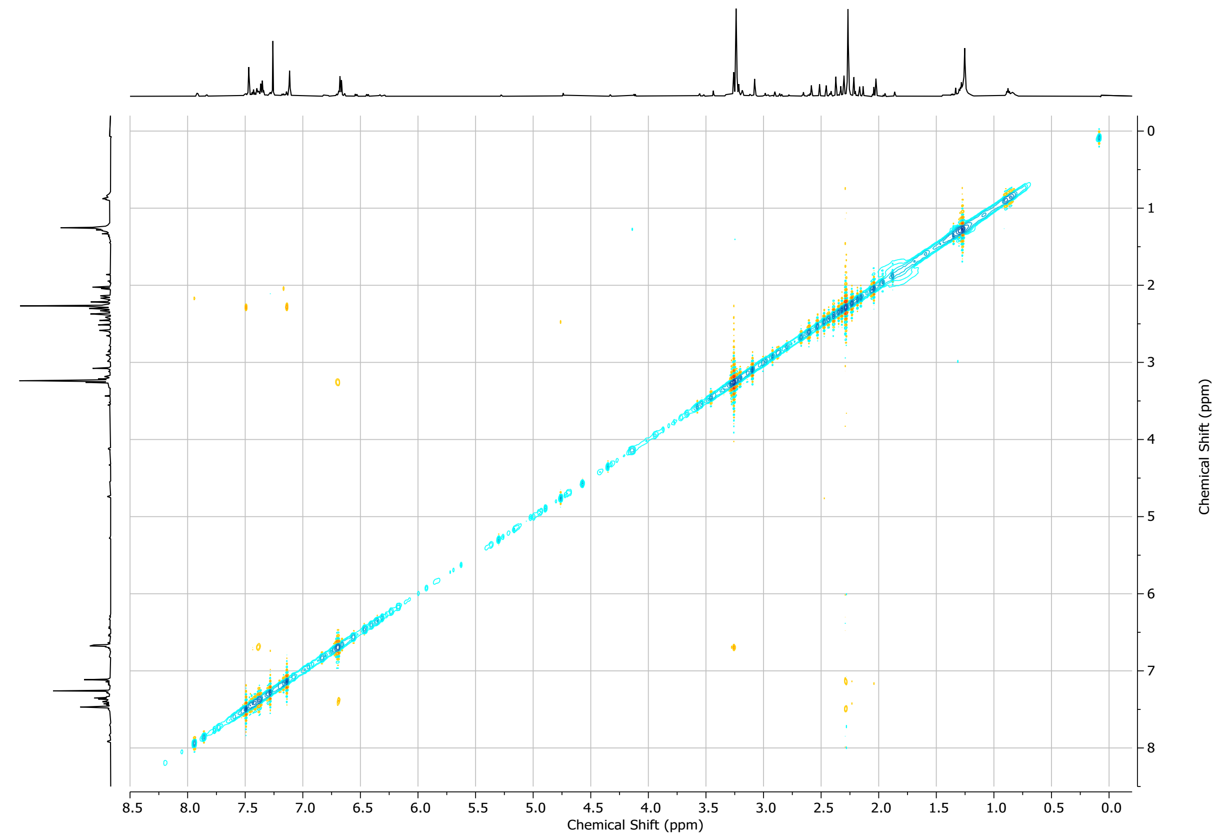

Figure S145. *NOESY NMR spectrum of a mixture of (Z_SZ_S)-**6** (64%), (E_SZ_S)-**6** (29%) and (E_SE_S)-**6** (7%) ($CDCl_3$, 20 °C).*

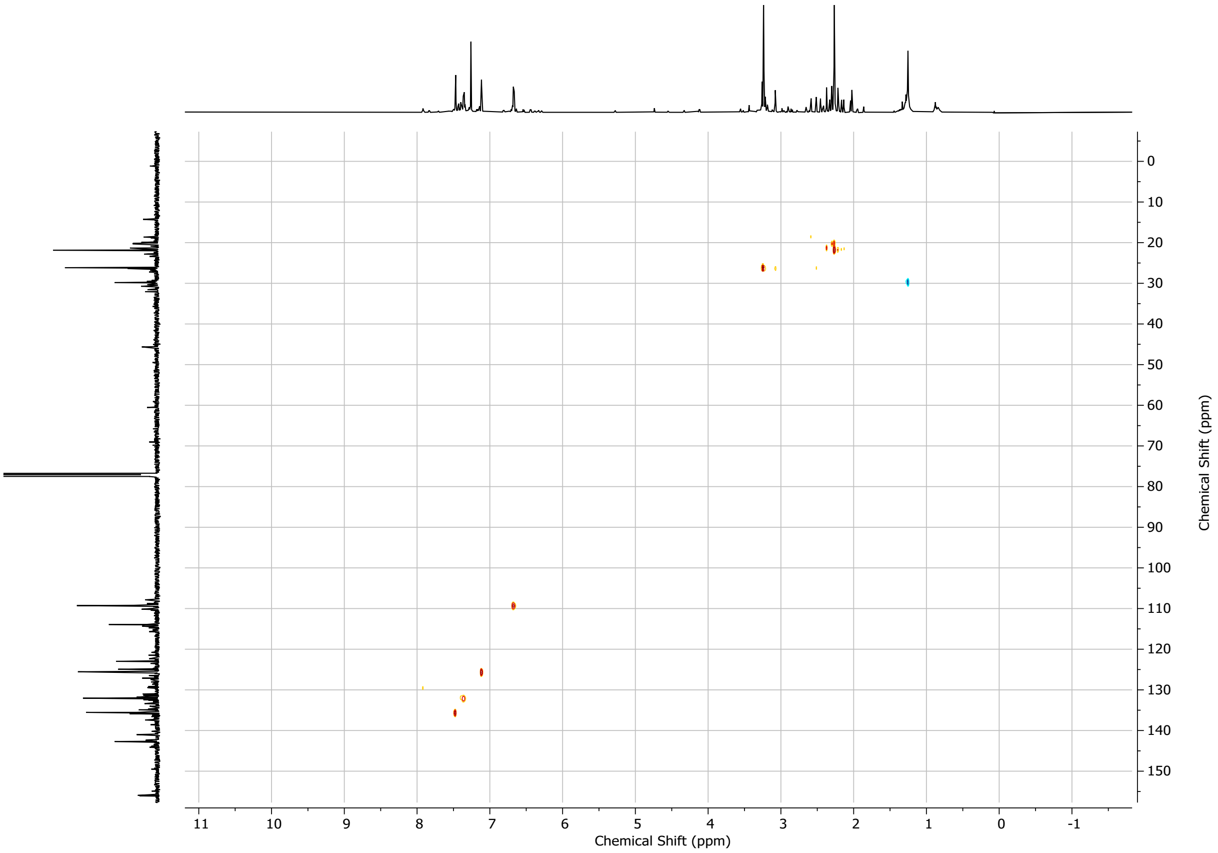

Figure S146. *HSQC NMR spectrum of a mixture of (Z_SZ_S)-**6** (64%), (E_SZ_S)-**6** (29%) and (E_SE_S)-**6** (7%) ($CDCl_3$, 20 °C).*

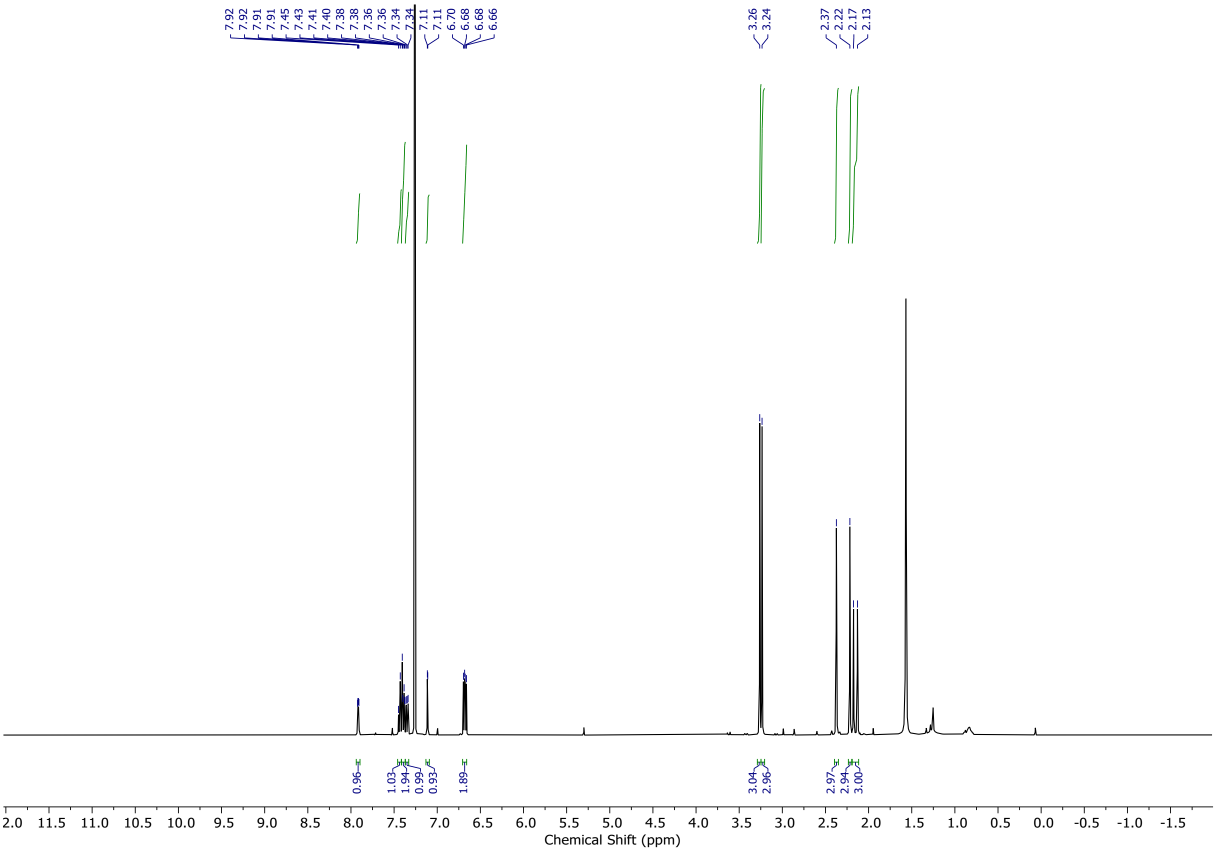

Figure S147. *^{1}H NMR spectrum of (E_SZ_S)-**6** ($CDCl_3$, 20 °C).*

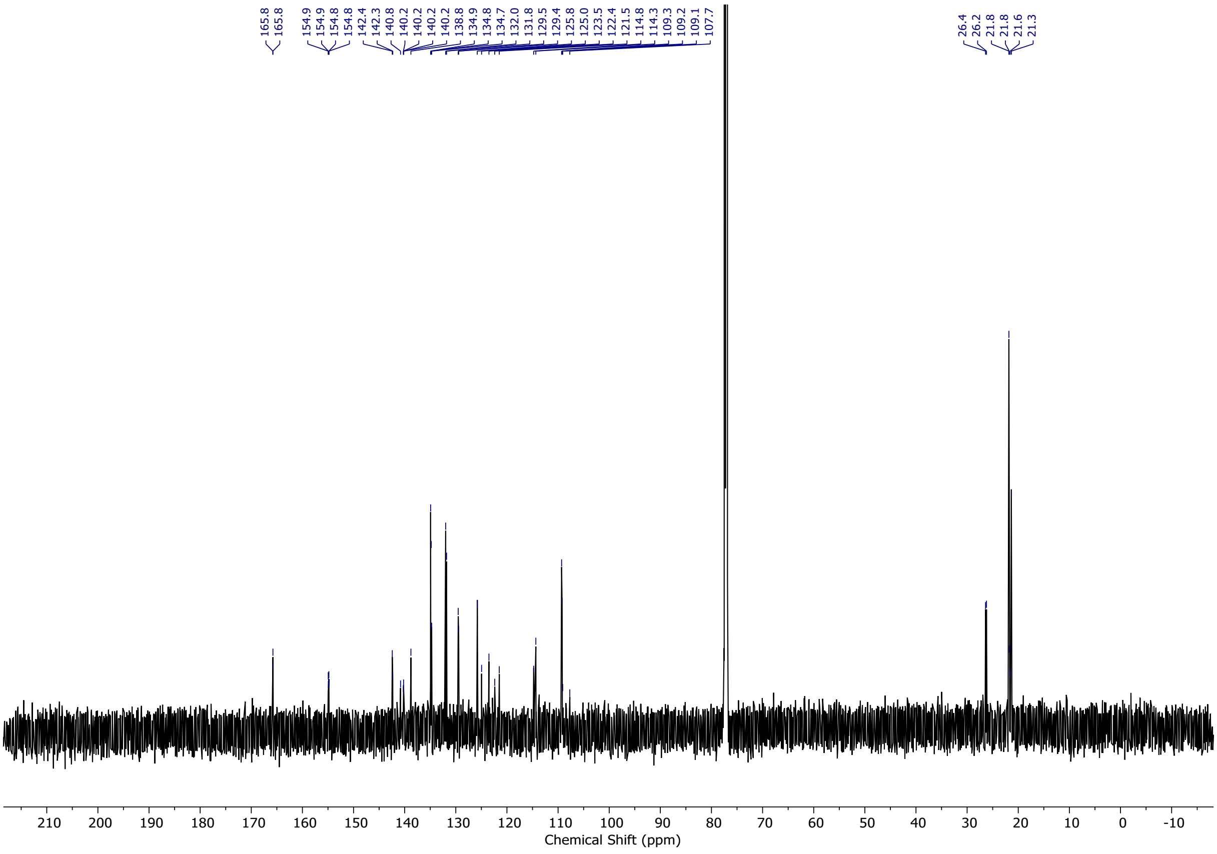

Figure S148. *^{13}C NMR spectrum of (E_SZ_S)-**6** ($CDCl_3$, 20 °C).*

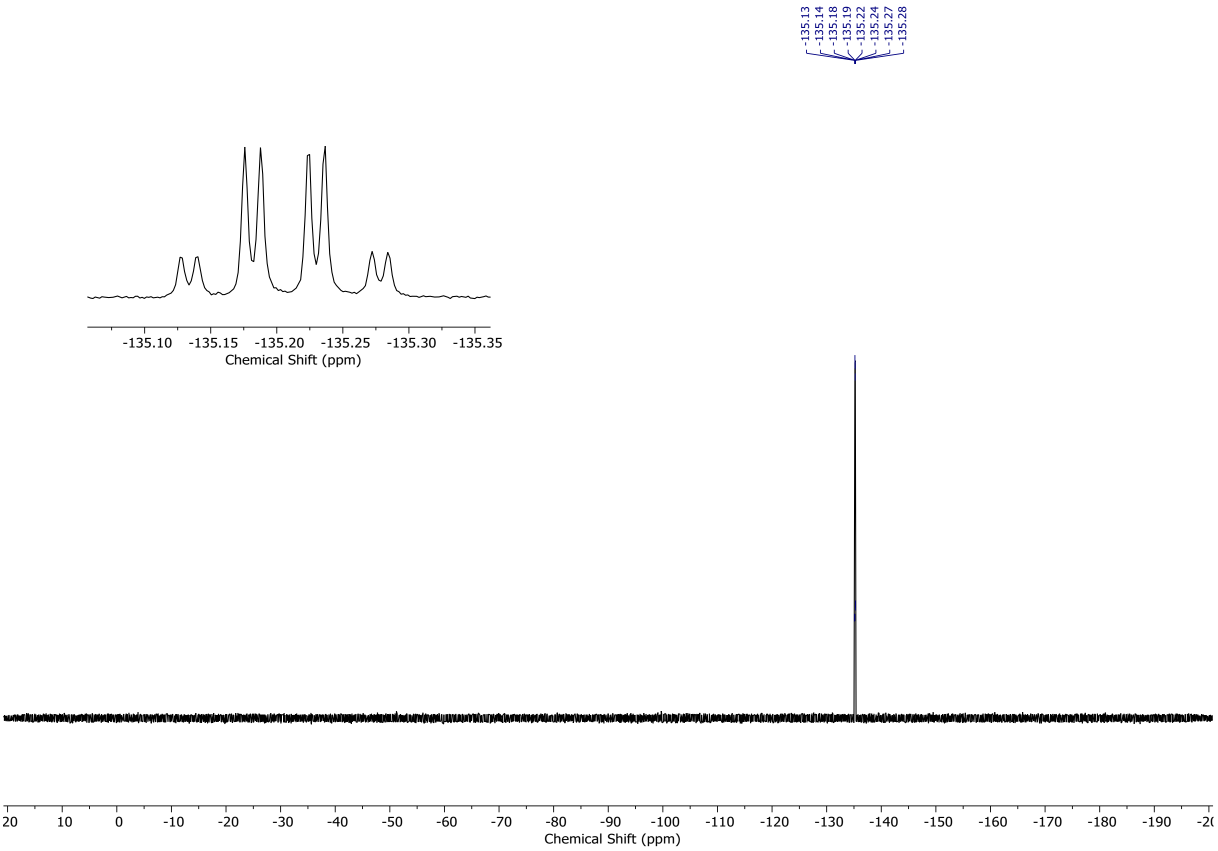

Figure S149. *^{19}F NMR spectrum of (E_SZ_S)-**6** ($CDCl_3$, 20 °C).*

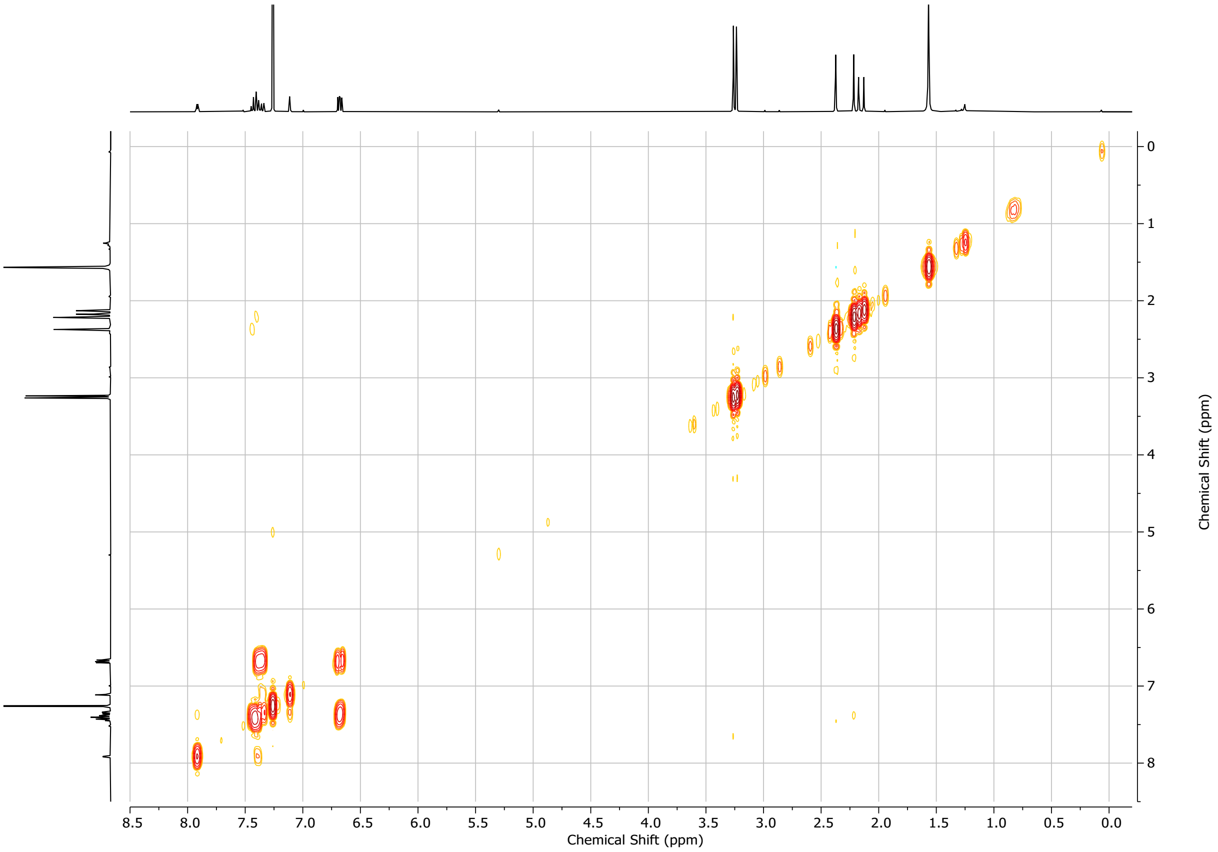

Figure S150. *COSY NMR spectrum of (E_SZ_S)-**6** ($CDCl_3$, 20 °C).*

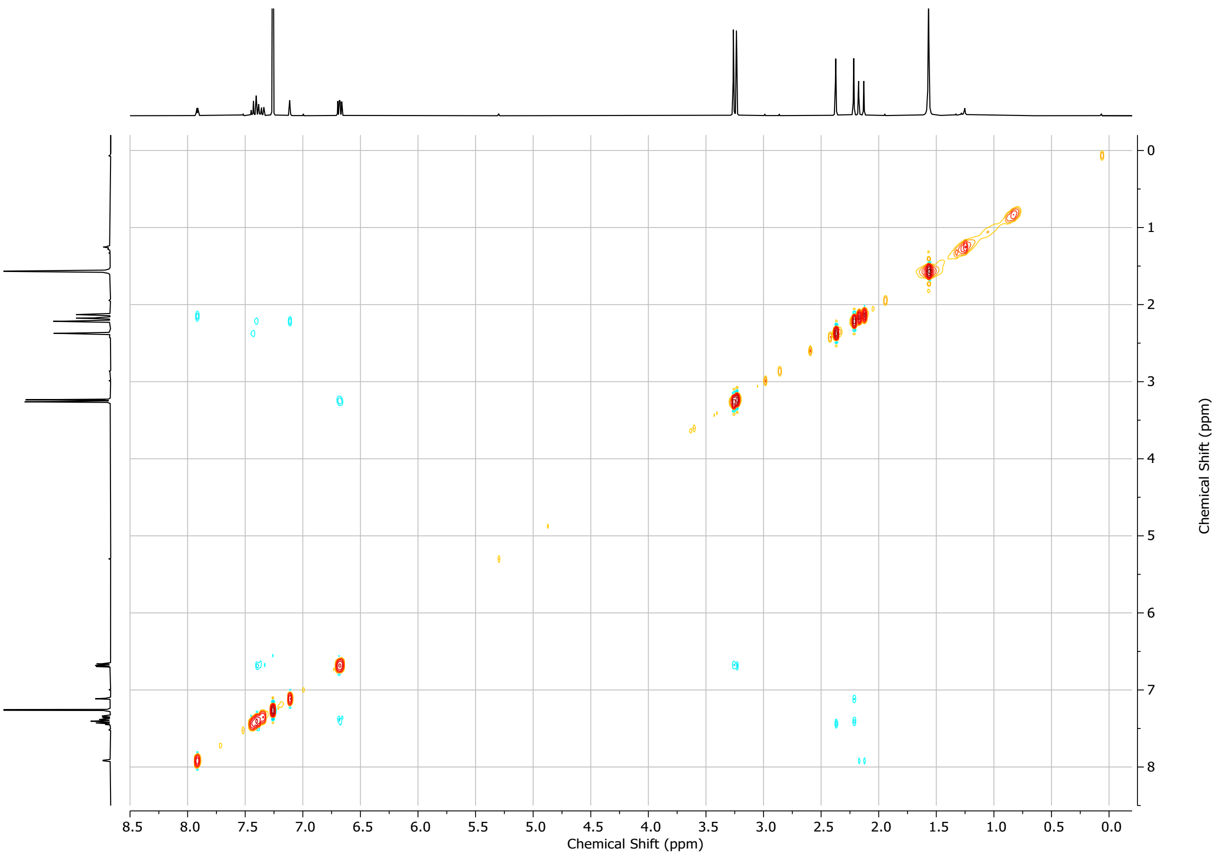

***Figure S151**. NOESY NMR spectrum of (E_SZ_S)-**6** ($CDCl_3$, 20 °C).*

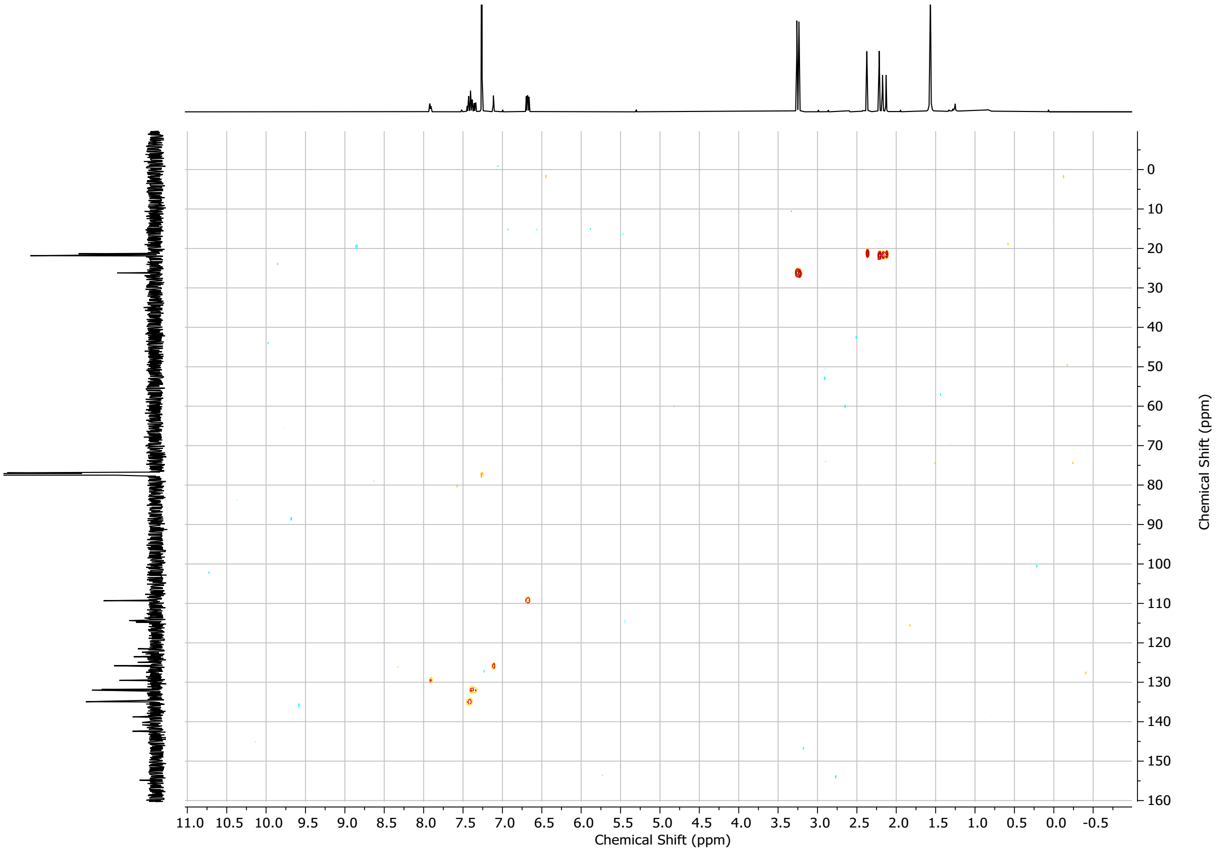

***Figure S152**. HSQC NMR spectrum of (E_SZ_S)-**6** ($CDCl_3$, 20 °C).*

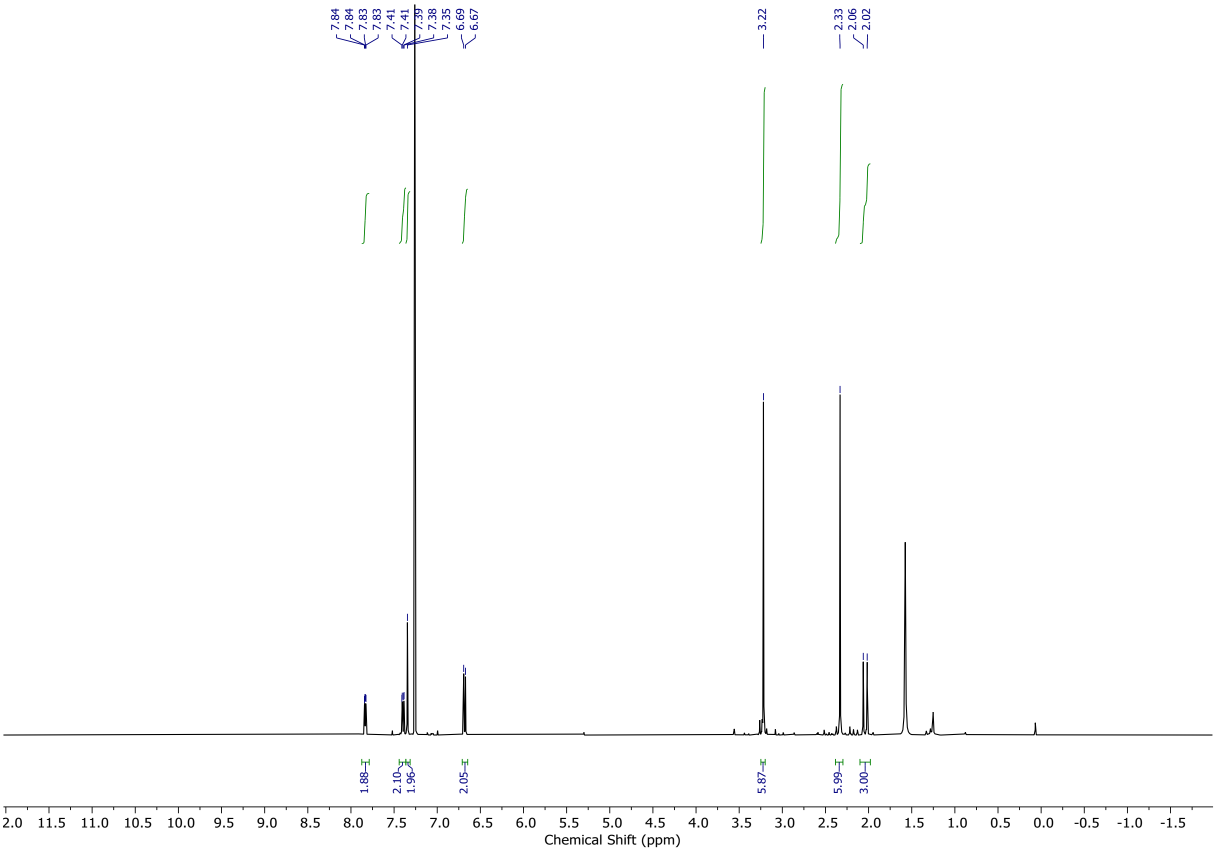

Figure S153. *^{1}H NMR spectrum of (E_SE_S)-**6** ($CDCl_3$, 20 °C).*

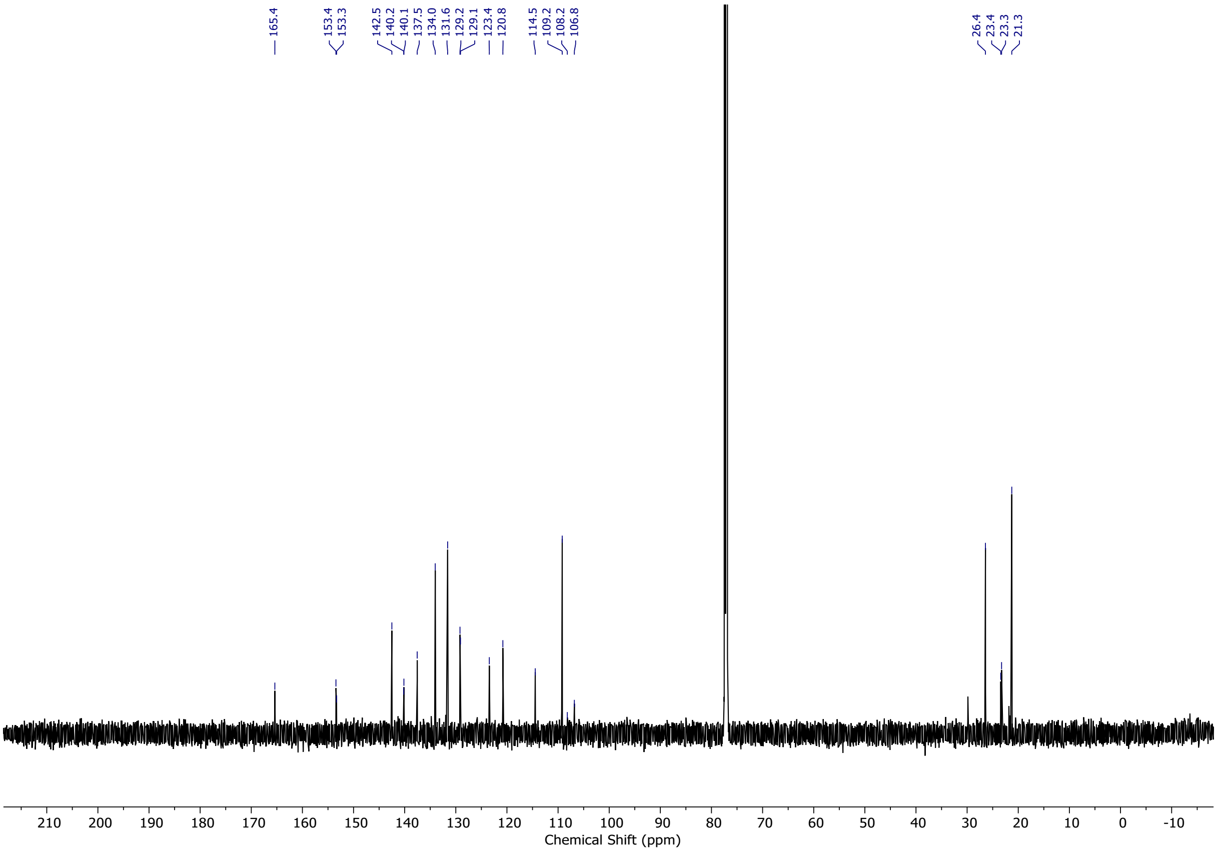

Figure S154. *^{13}C NMR spectrum of (E_SE_S)-**6** ($CDCl_3$, 20 °C).*

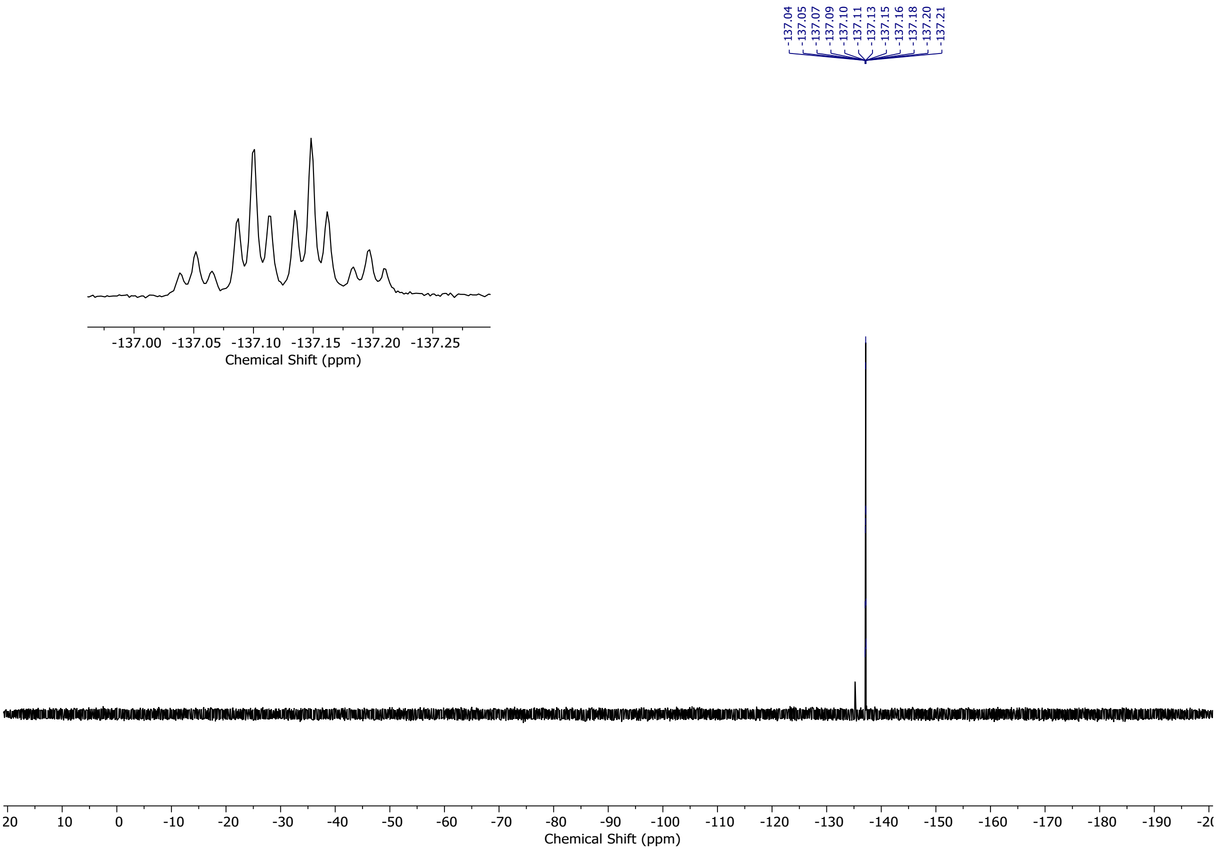

Figure S155. *^{19}F NMR spectrum of (E_SE_S)-**6** ($CDCl_3$, 20 °C).*

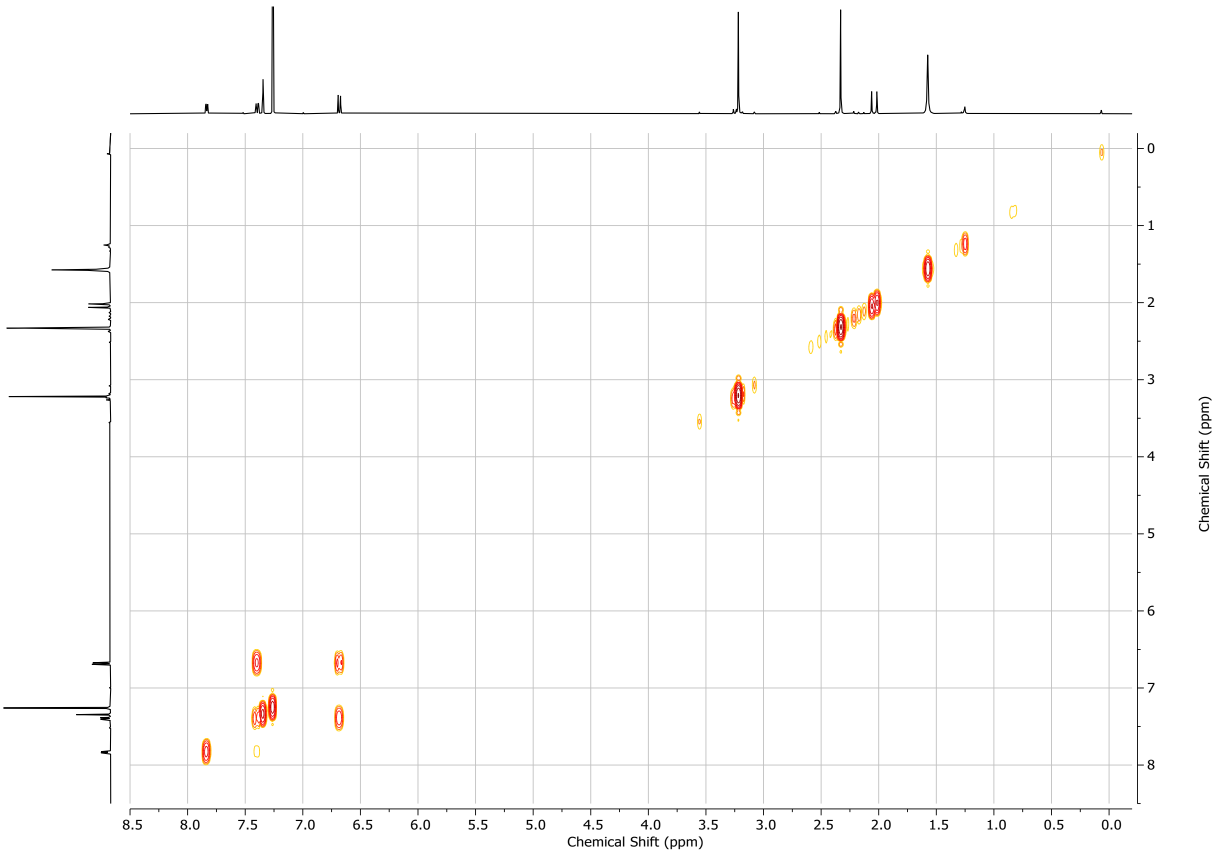

Figure S156. *COSY NMR spectrum of (E_SE_S)-**6** ($CDCl_3$, 20 °C).*

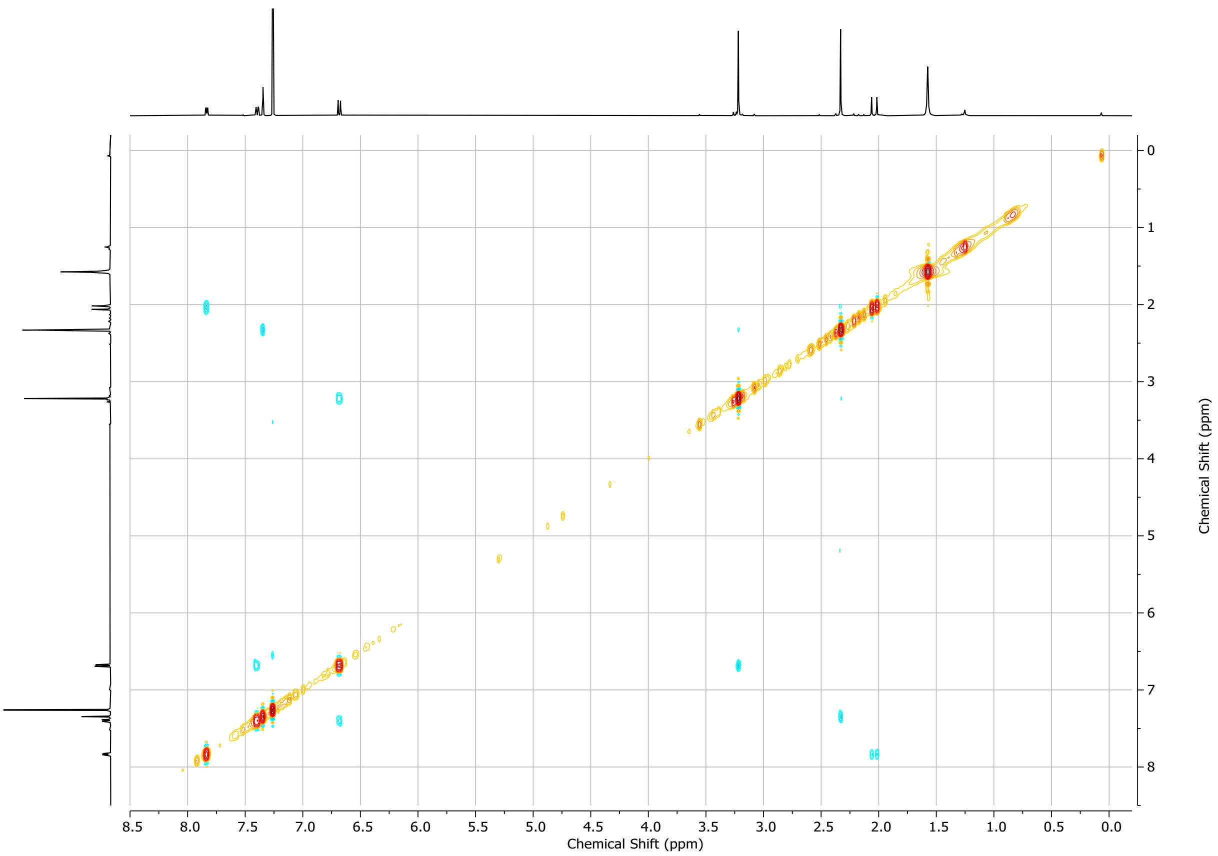

Figure S157. *NOESY NMR spectrum of (E_SE_S)-**6** ($CDCl_3$, 20 °C).*

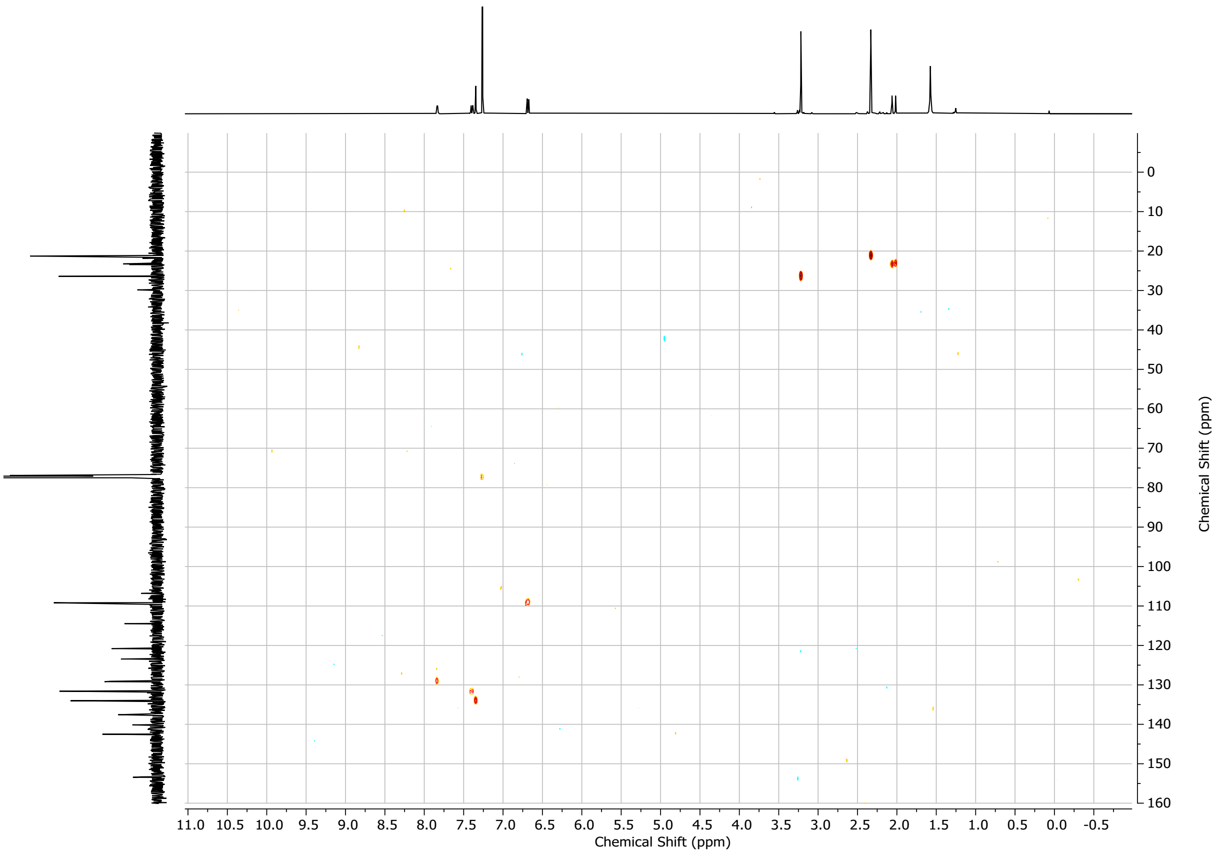

Figure S158. *HSQC NMR spectrum of (E_SE_S)-**6** ($CDCl_3$, 20 °C).*

Motor 7

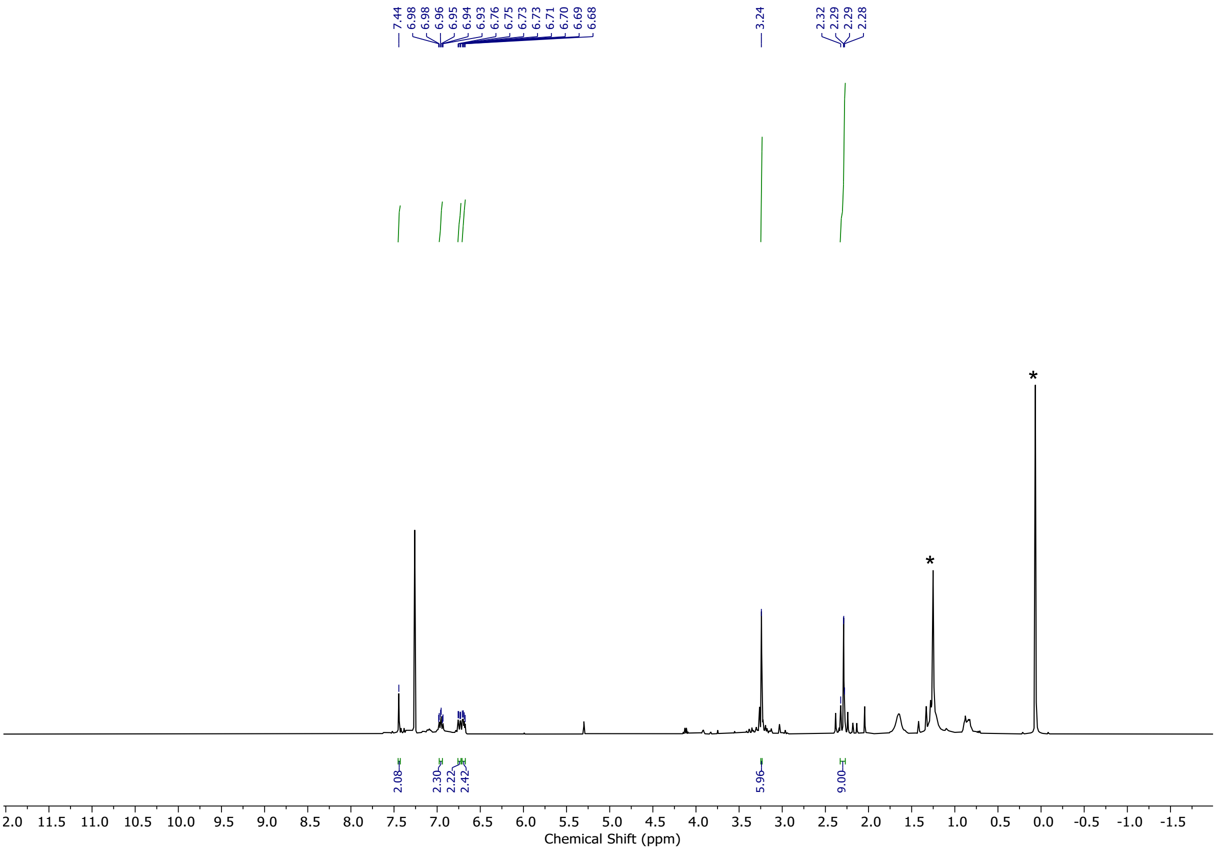

Figure S159. ^{1}H NMR spectrum of a mixture of (Z_SZ_S)-**7** (72%) and (E_SZ_S)-**7** (28%) ($CDCl_3$, 20 °C). The peaks marked with an asterisk arise from grease.

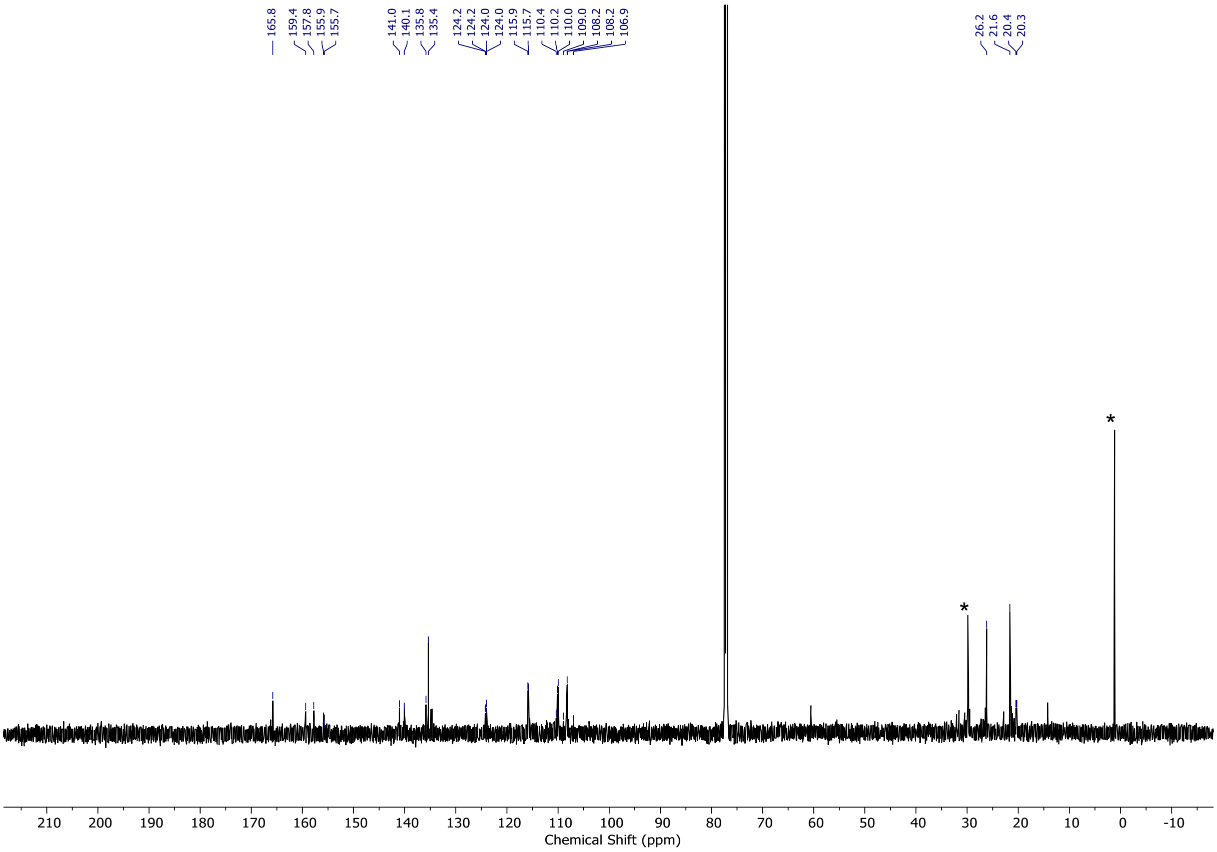

Figure S160. ^{13}C NMR spectrum of (Z_SZ_S)-**7** (72%) and (E_SZ_S)-**7** (28%) ($CDCl_3$, 20 °C). The peaks marked with an asterisk arise from grease.

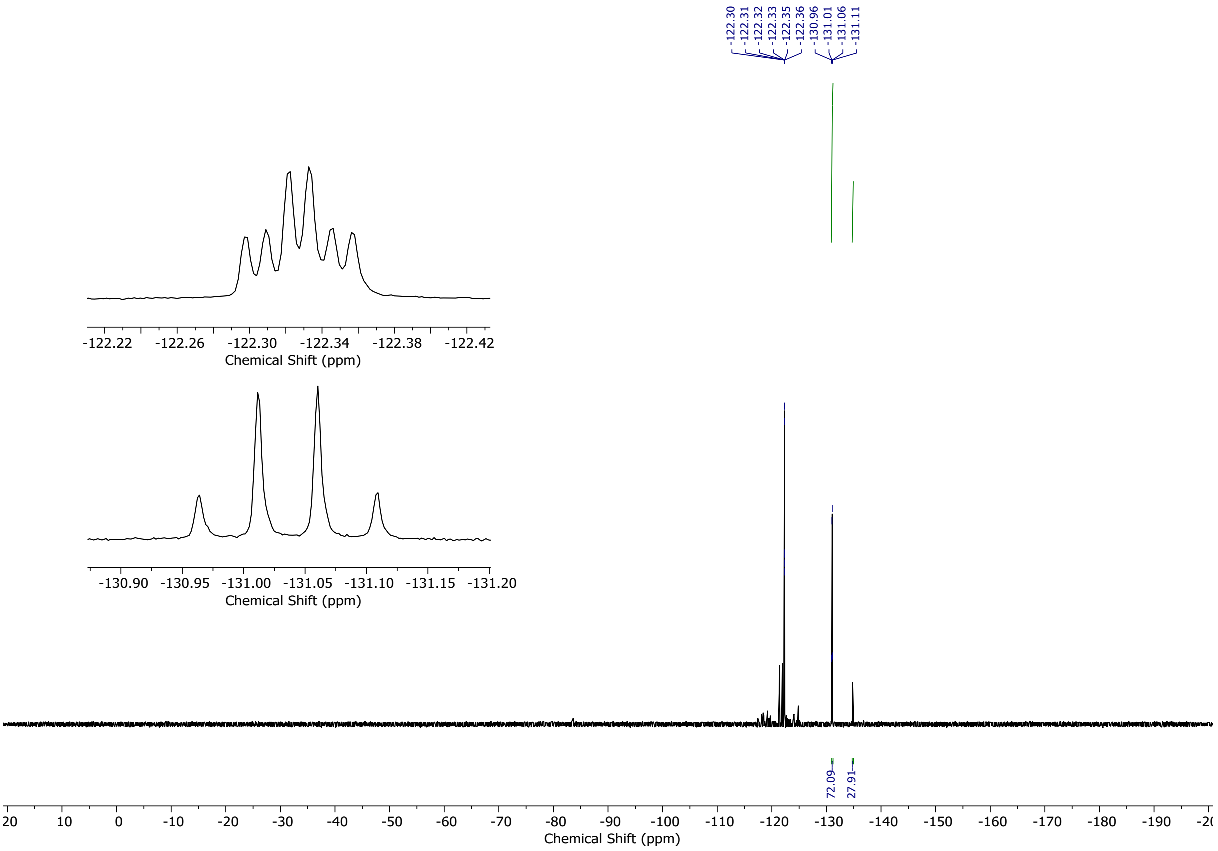

Figure S161. *^{19}F NMR spectrum of (Z_SZ_S)-**7** (72%) and (E_SZ_S)-**7** (28%) ($CDCl_3$, 20 °C).*

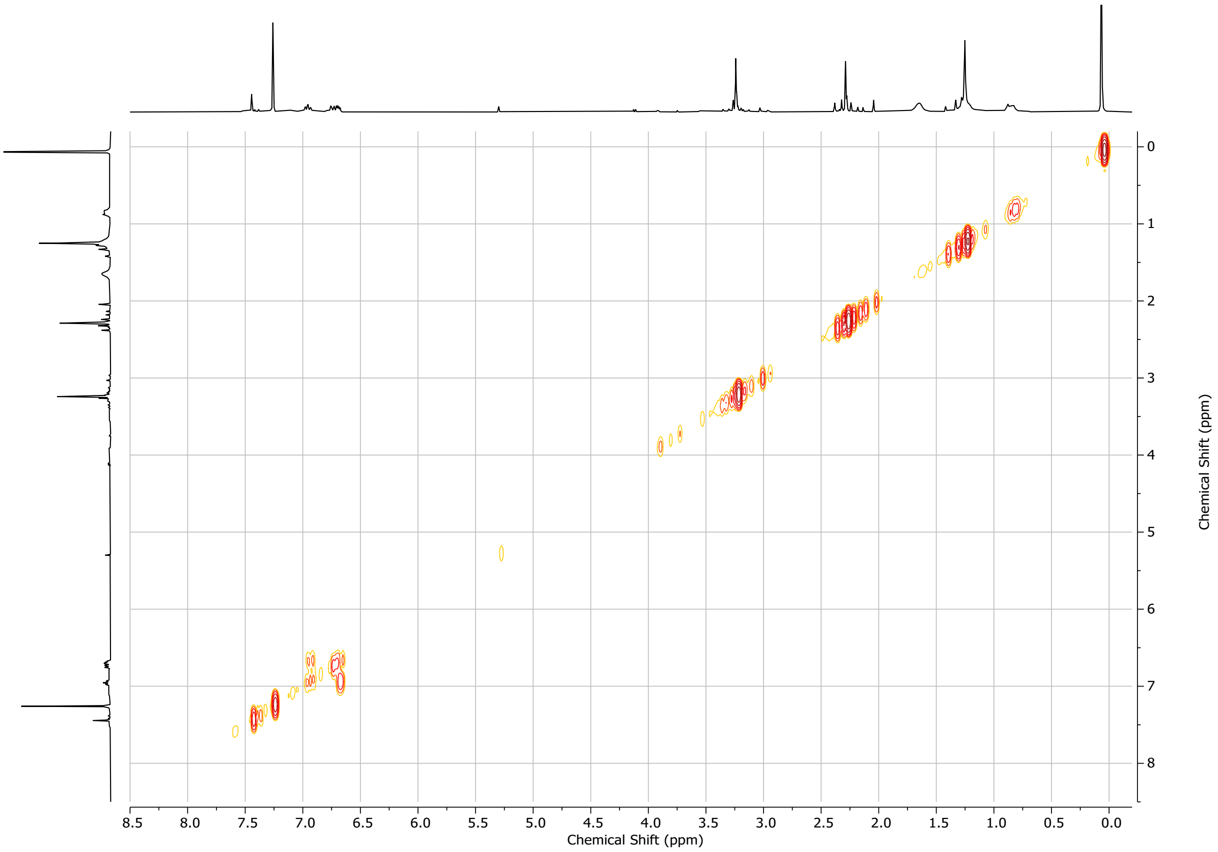

Figure S162. *COSY NMR spectrum of (Z_SZ_S)-**7** (72%) and (E_SZ_S)-**7** (28%) ($CDCl_3$, 20 °C).*

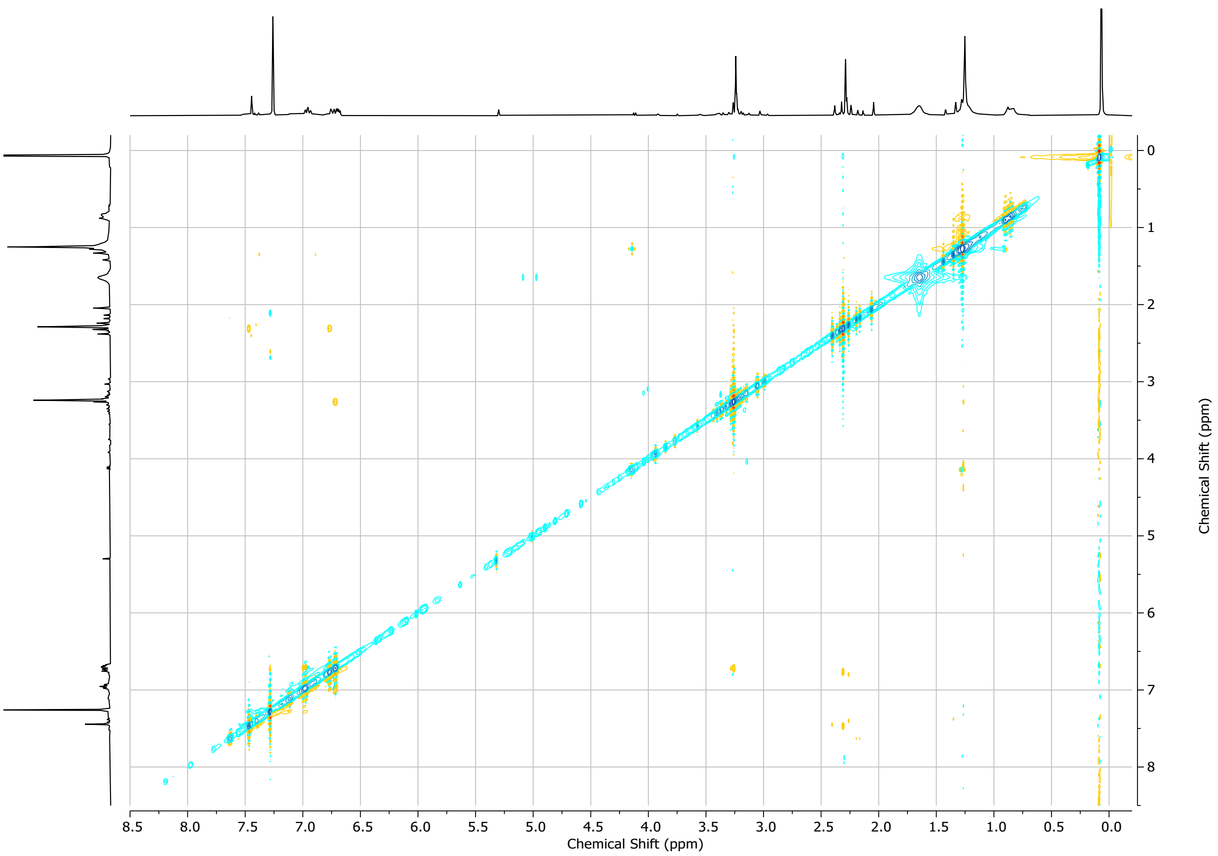

Figure S163. *NOESY NMR spectrum of (Z_SZ_S)-**7** (72%) and (E_SZ_S)-**7** (28%) ($CDCl_3$, 20 °C).*

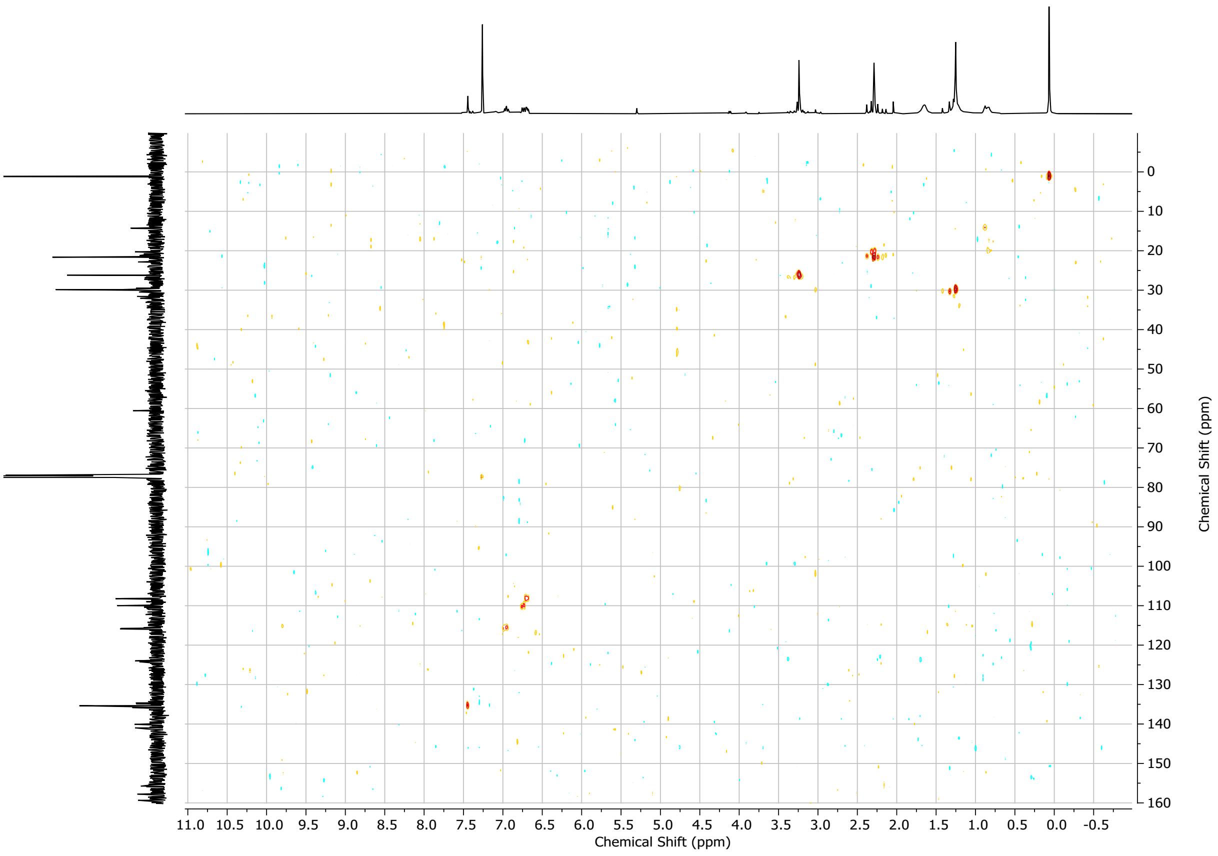

Figure S164. *HSQC NMR spectrum of (Z_SZ_S)-**7** (72%) and (E_SZ_S)-**7** (28%) ($CDCl_3$, 20 °C).*

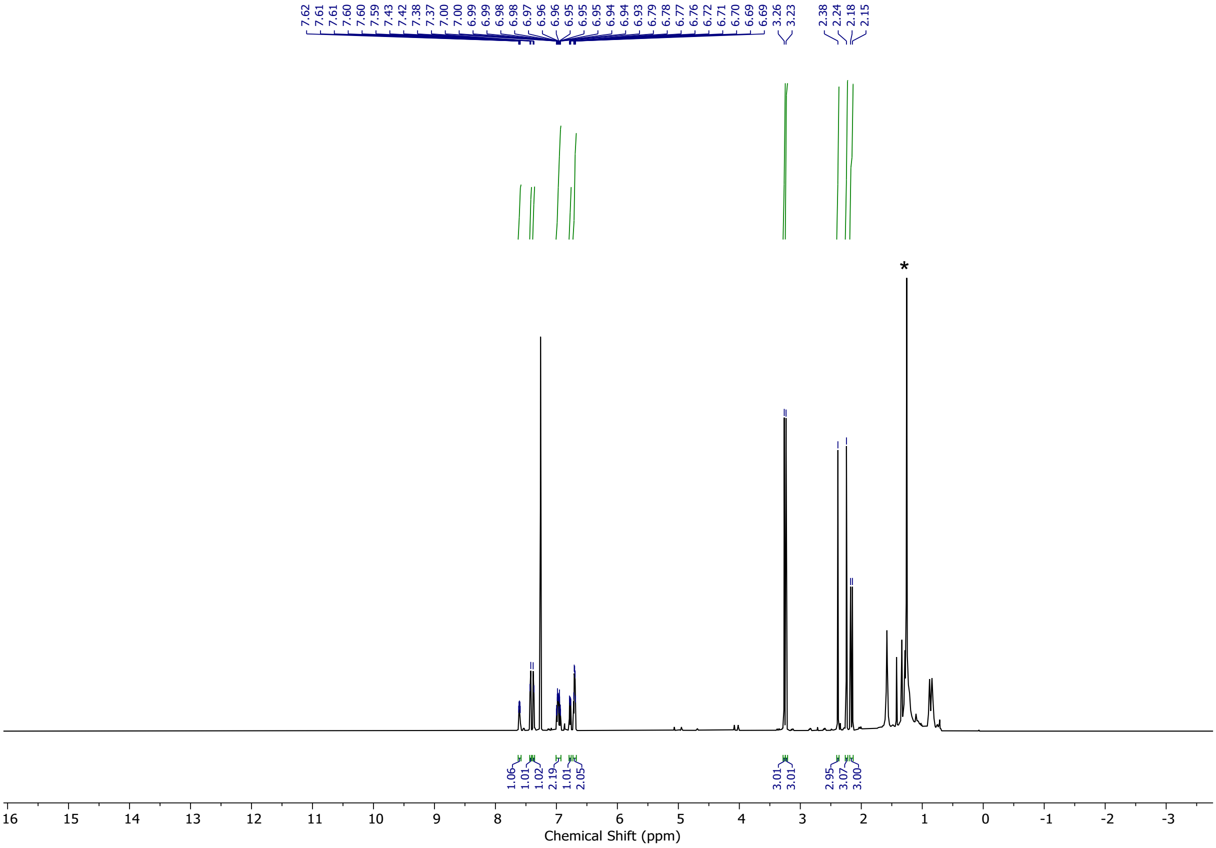

Figure S165. *^{1}H NMR spectrum of (E_SZ_S)-**7** ($CDCl_3$, 20 °C). The peak marked with an asterisk arises from grease.*

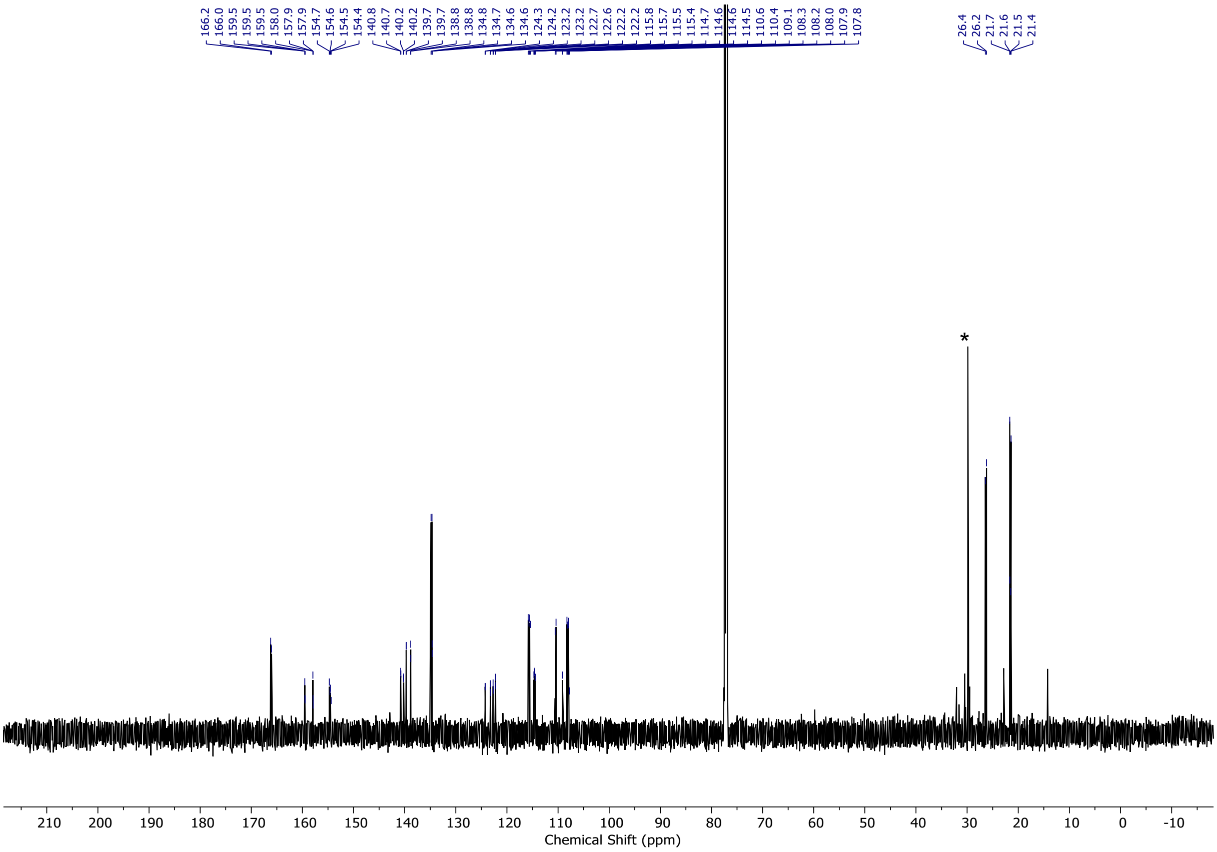

Figure S166. *^{13}C NMR spectrum of (E_SZ_S)-**7** ($CDCl_3$, 20 °C). The peak marked with an asterisk arises from grease.*

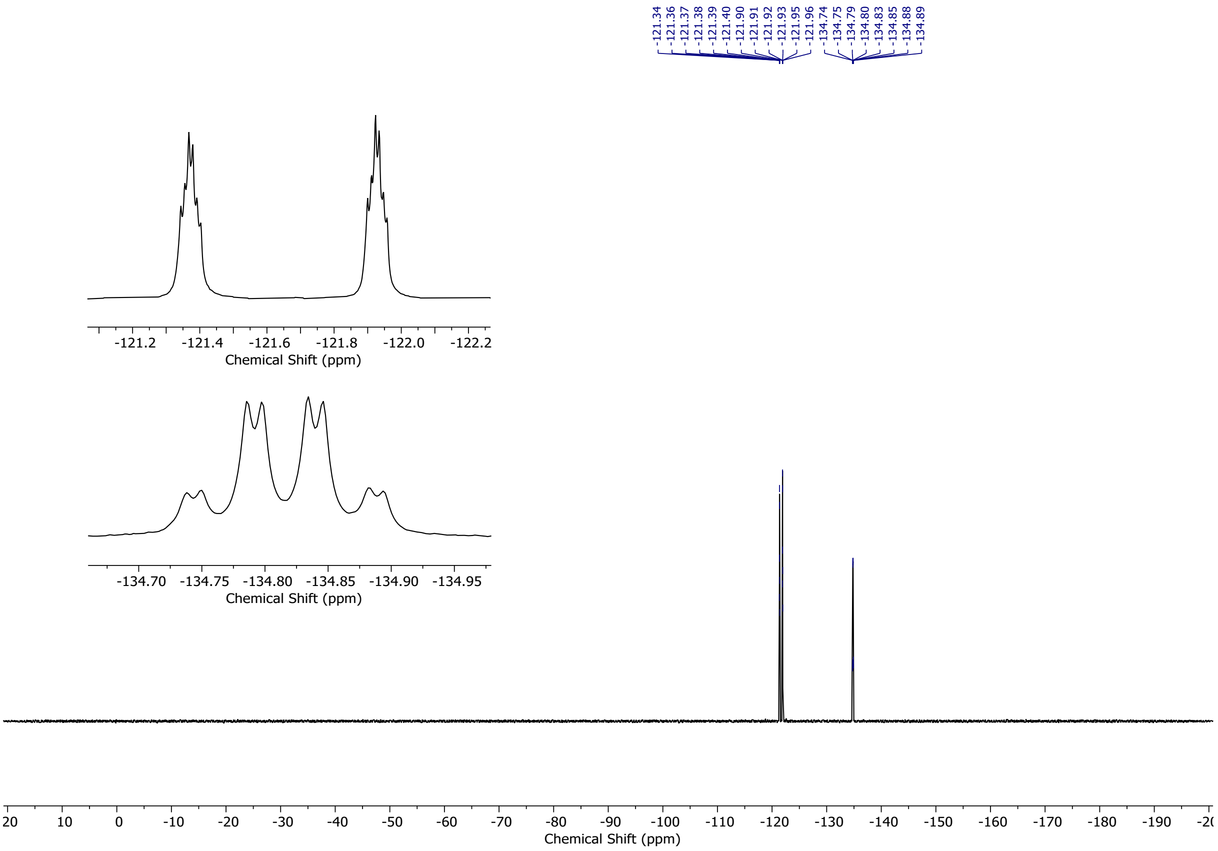

Figure S167. ^{19}F NMR spectrum of (E_SZ_S)-**7** ($CDCl_3$, 20 °C).

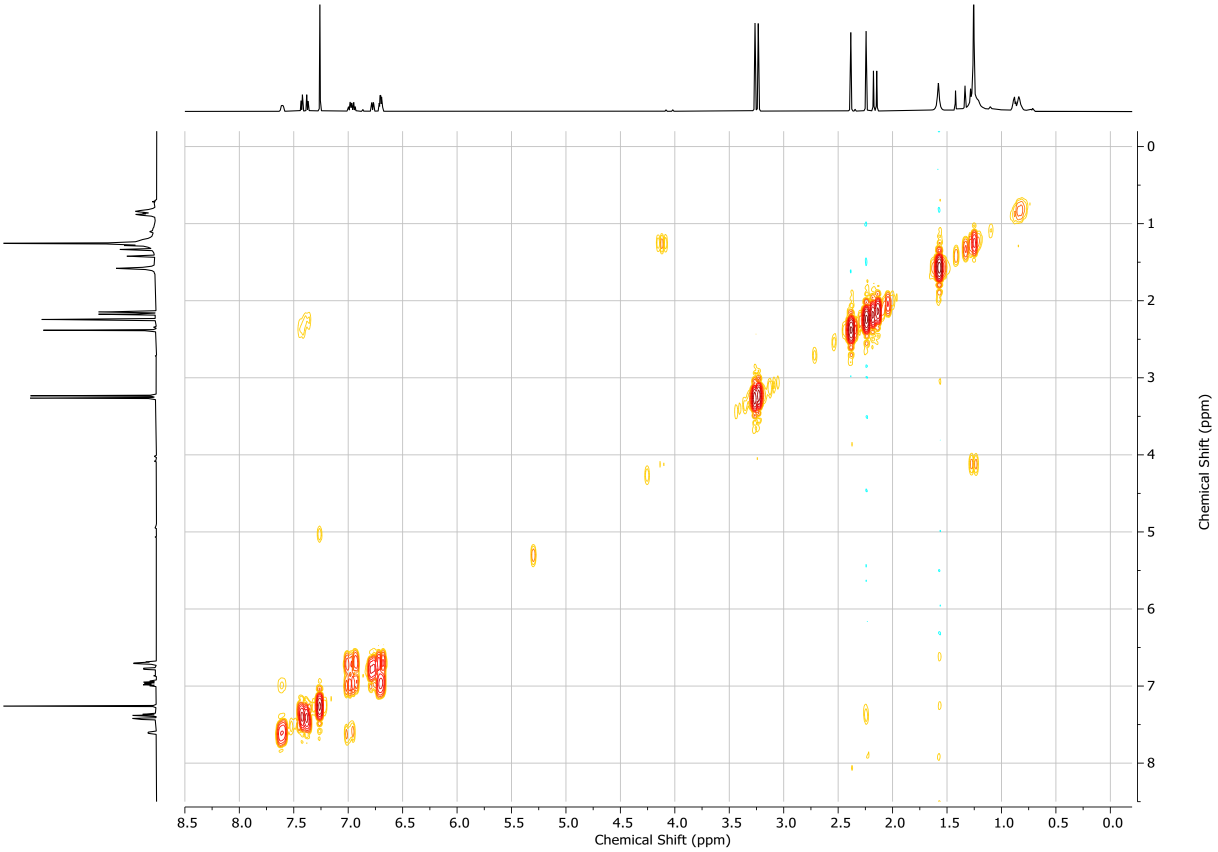

Figure S168. COSY NMR spectrum of (E_SZ_S)-**7** ($CDCl_3$, 20 °C).

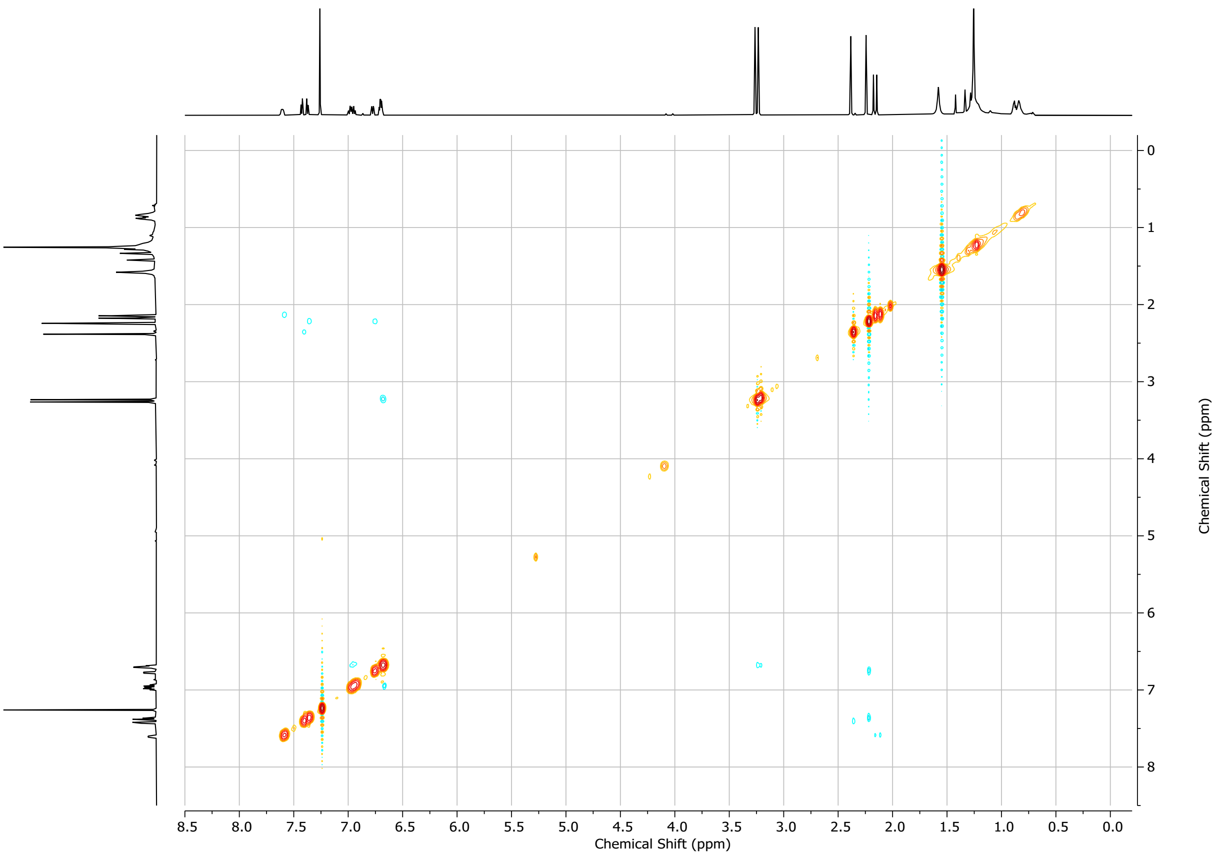

Figure S169. *NOESY NMR spectrum of (E_SZ_S)-**7** ($CDCl_3$, 20 °C).*

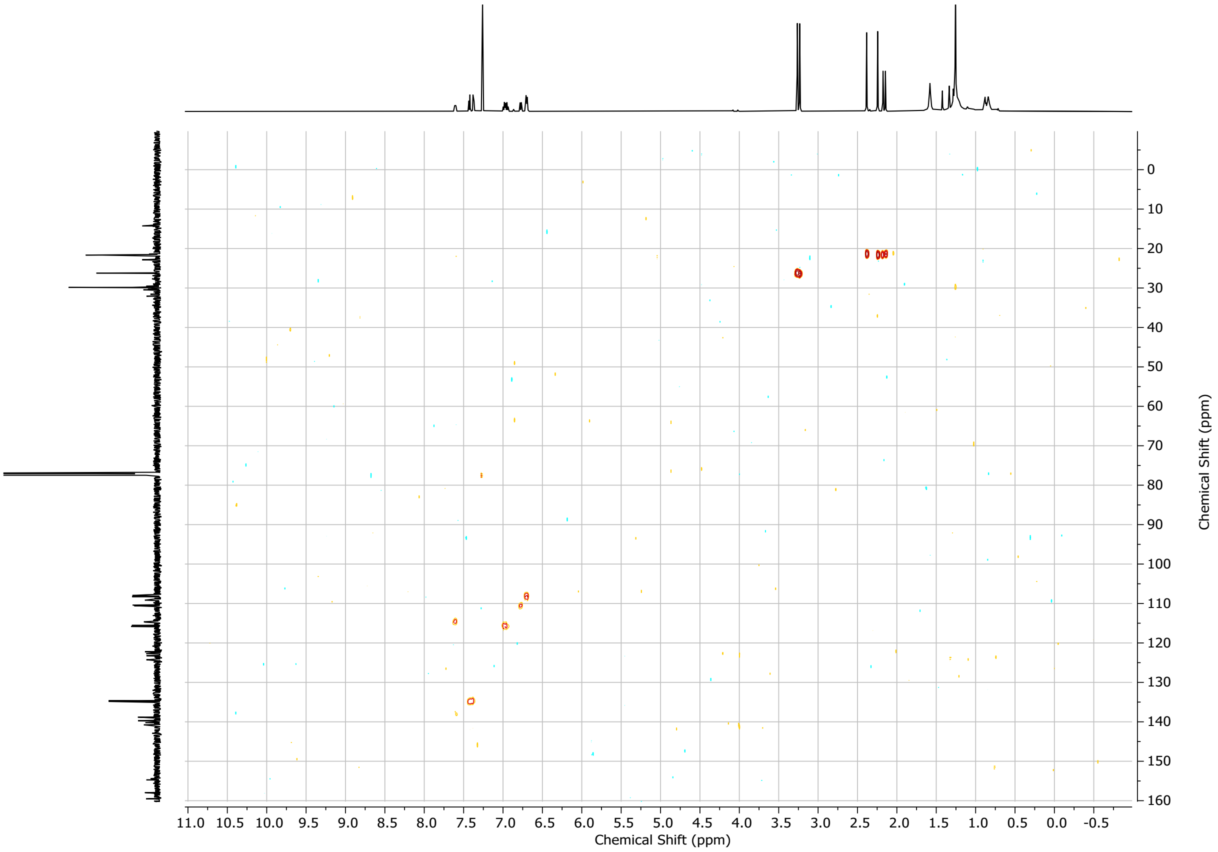

Figure S170. *HSQC NMR spectrum of (E_SZ_S)-**7** ($CDCl_3$, 20 °C).*

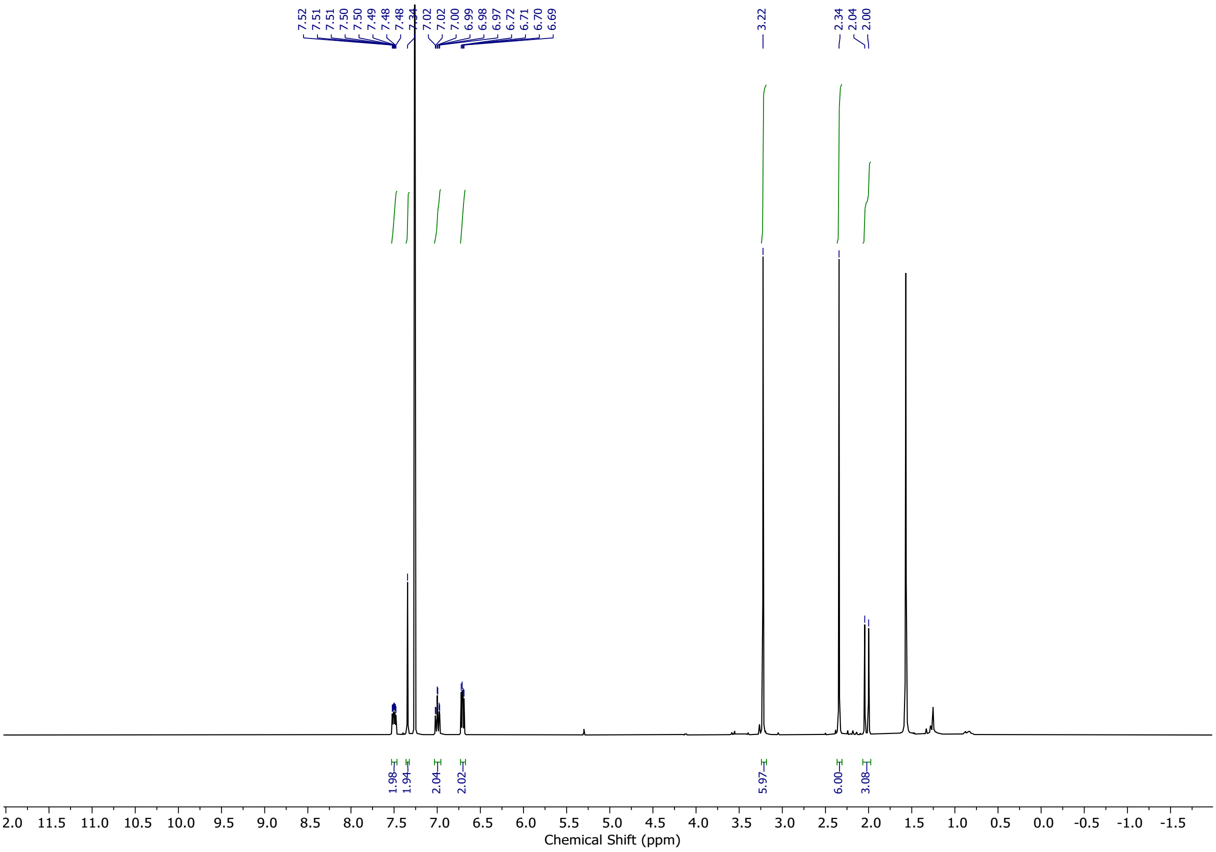

Figure S171. *^{1}H NMR spectrum of (E$_S$E$_S$)-**7** (CDCl$_3$, 20 °C).*

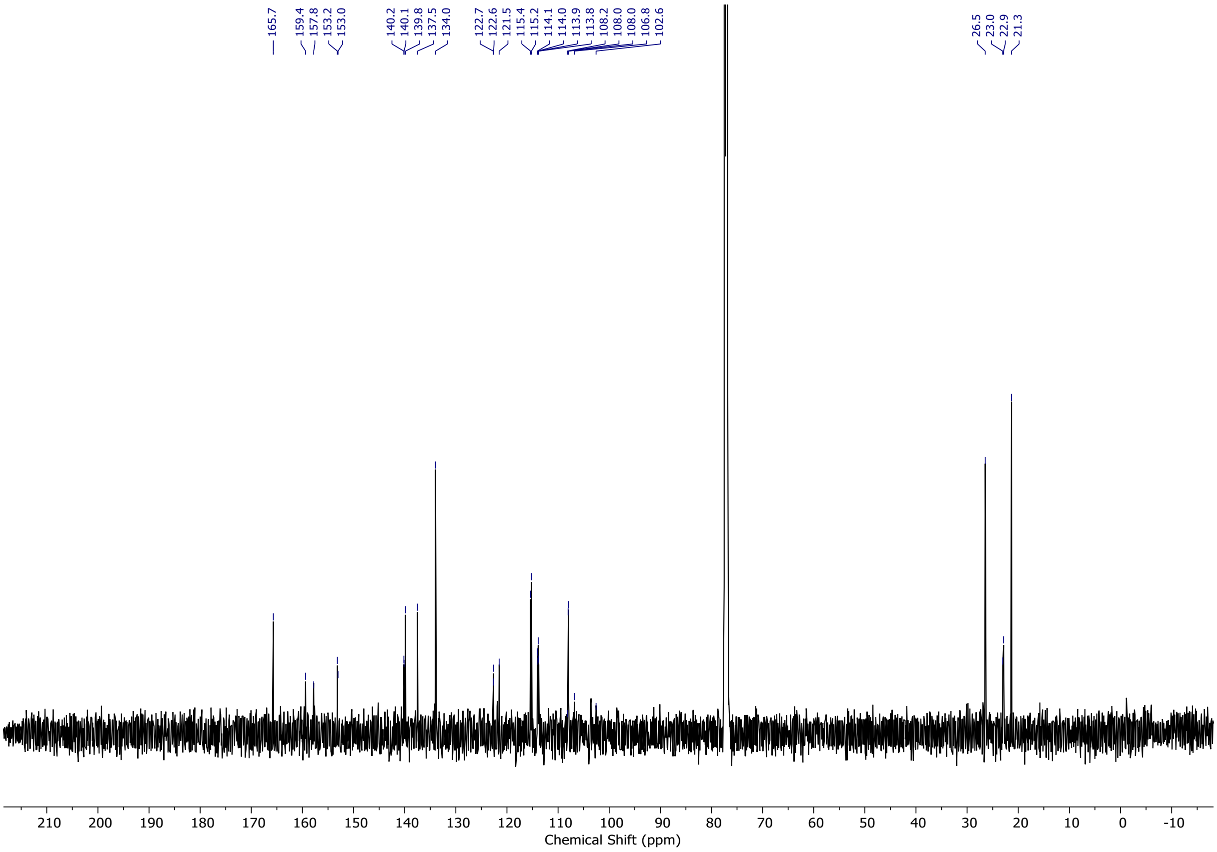

Figure S172. *^{13}C NMR spectrum of (E$_S$E$_S$)-**7** (CDCl$_3$, 20 °C).*

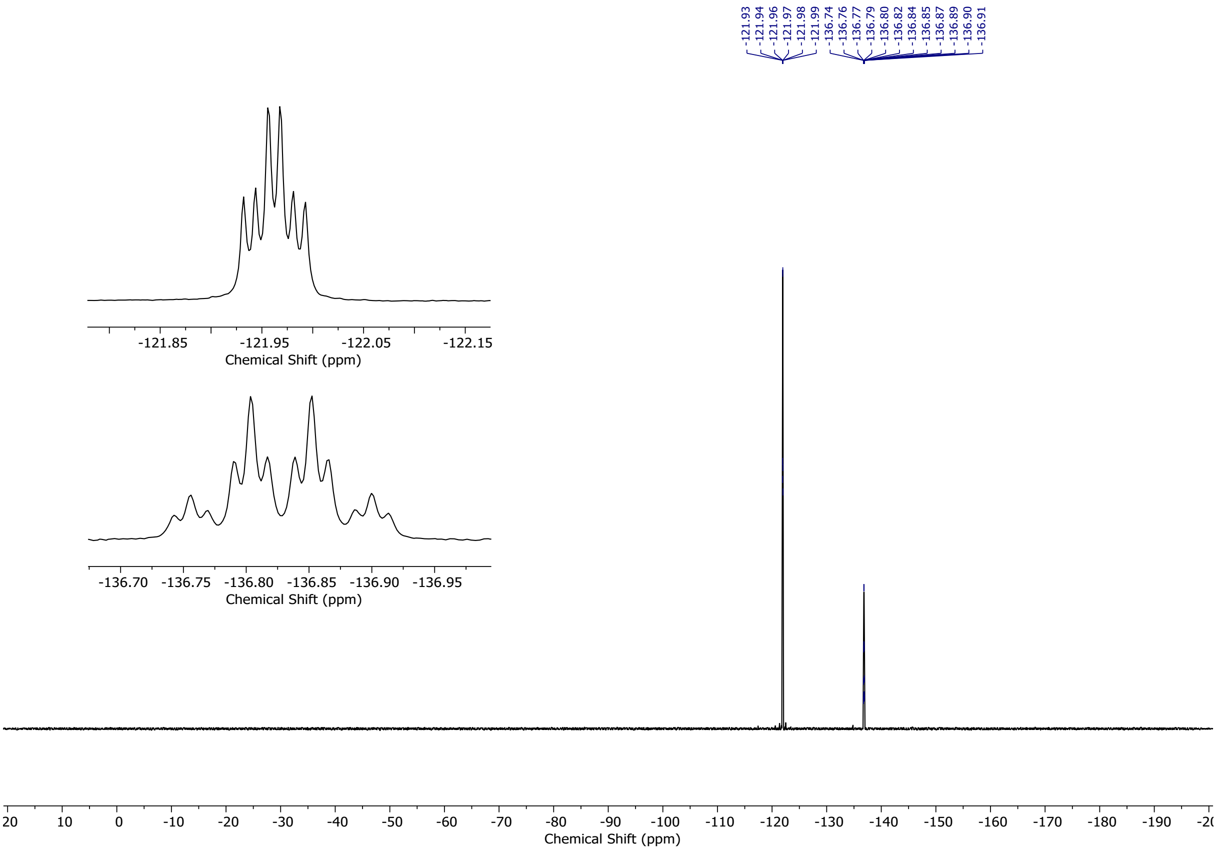

Figure S173. *^{19}F NMR spectrum of (E_SE_S)-**7** ($CDCl_3$, 20 °C).*

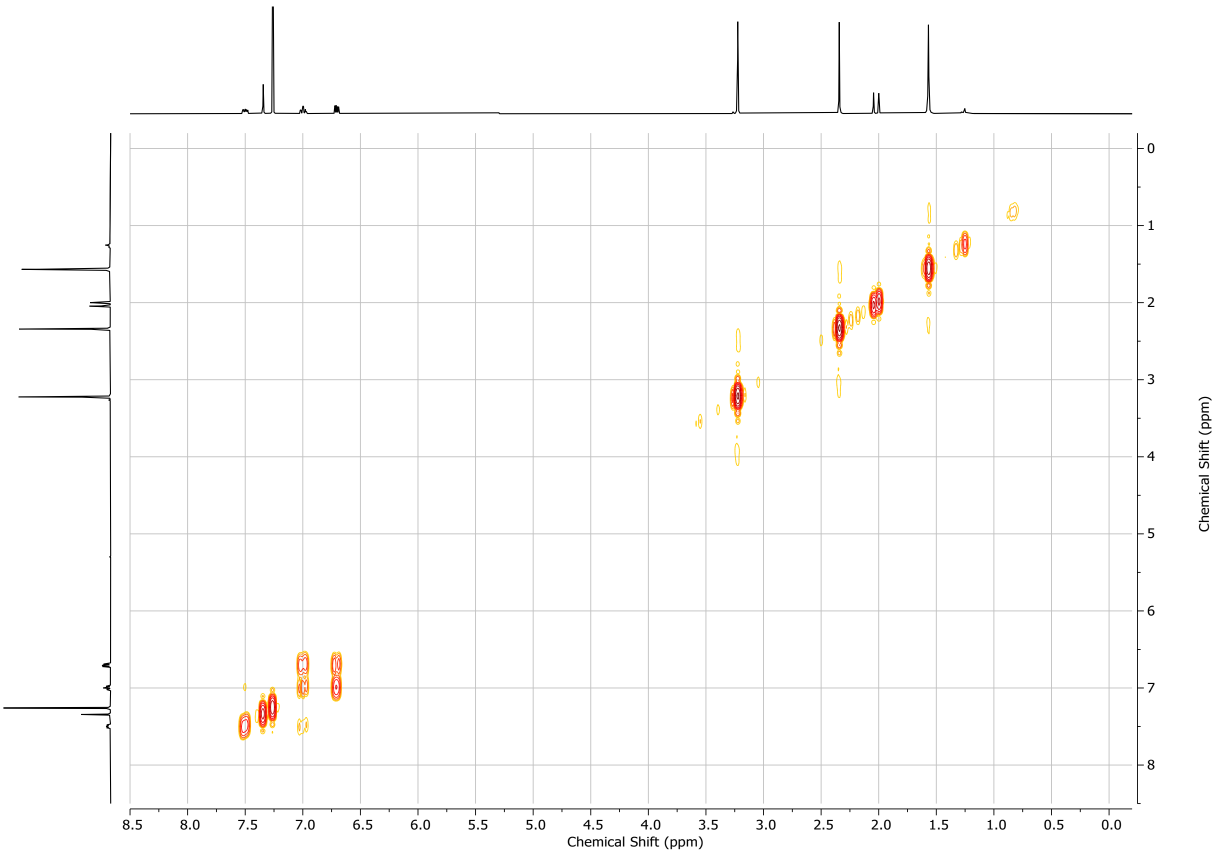

Figure S174. *COSY NMR spectrum of (E_SE_S)-**7** ($CDCl_3$, 20 °C).*

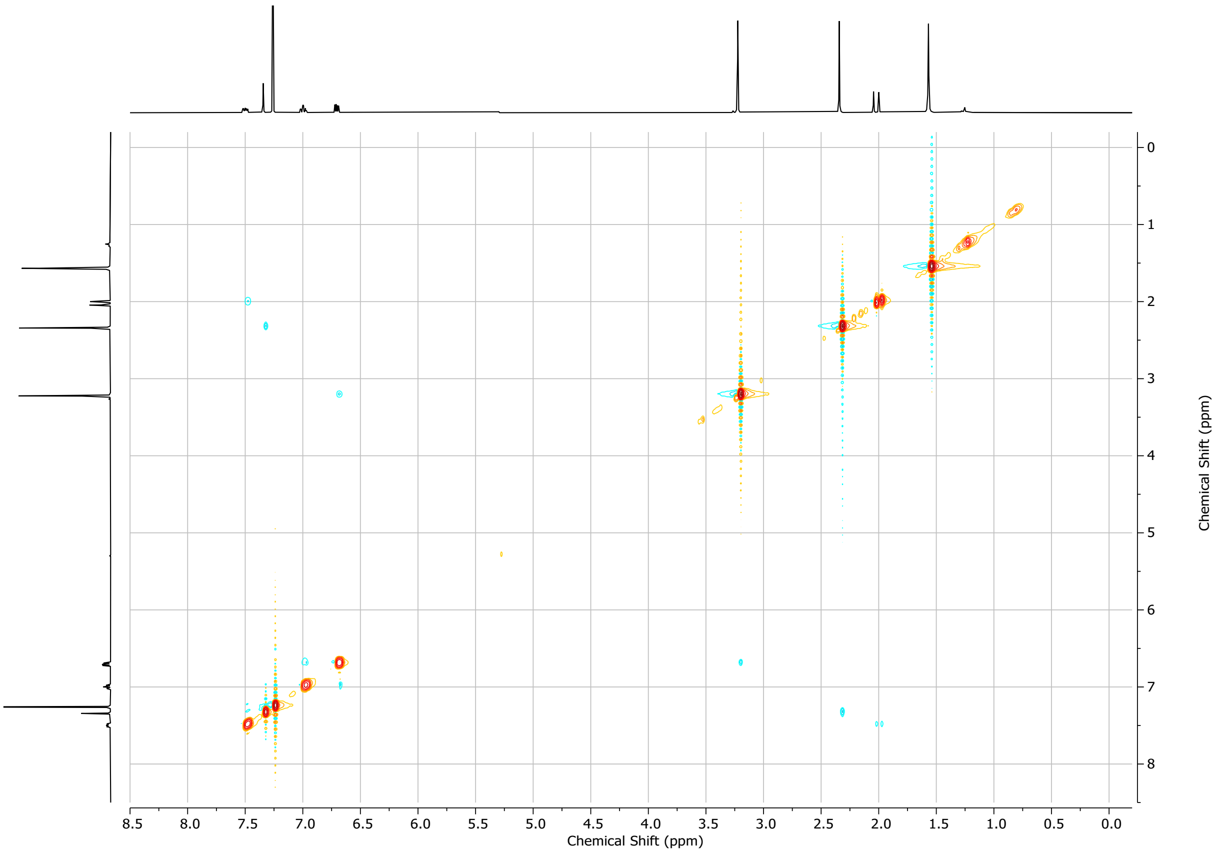

Figure S175. *NOESY NMR spectrum of (E_SE_S)-**7** ($CDCl_3$, 20 °C).*

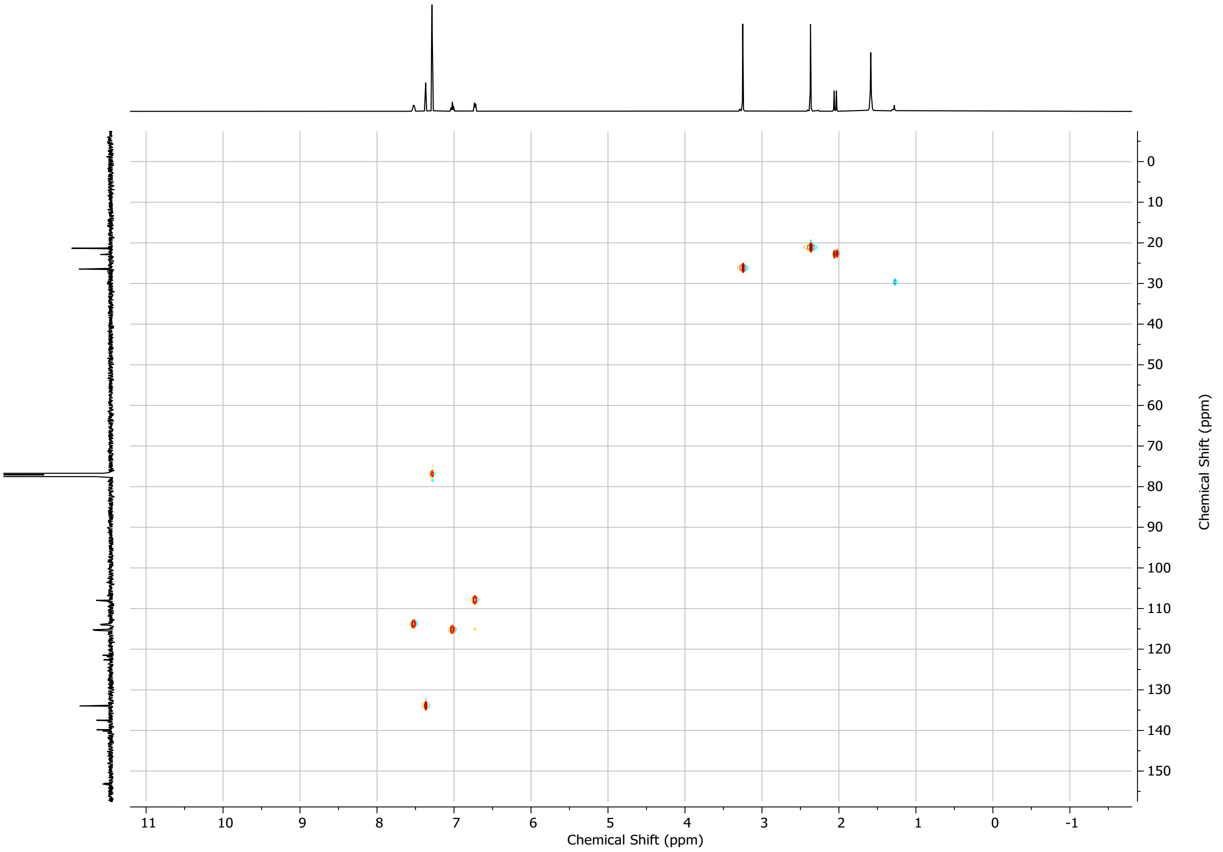

Figure S176. *HSQC NMR spectrum of (E_SE_S)-**7** ($CDCl_3$, 20 °C).*

Motor 8

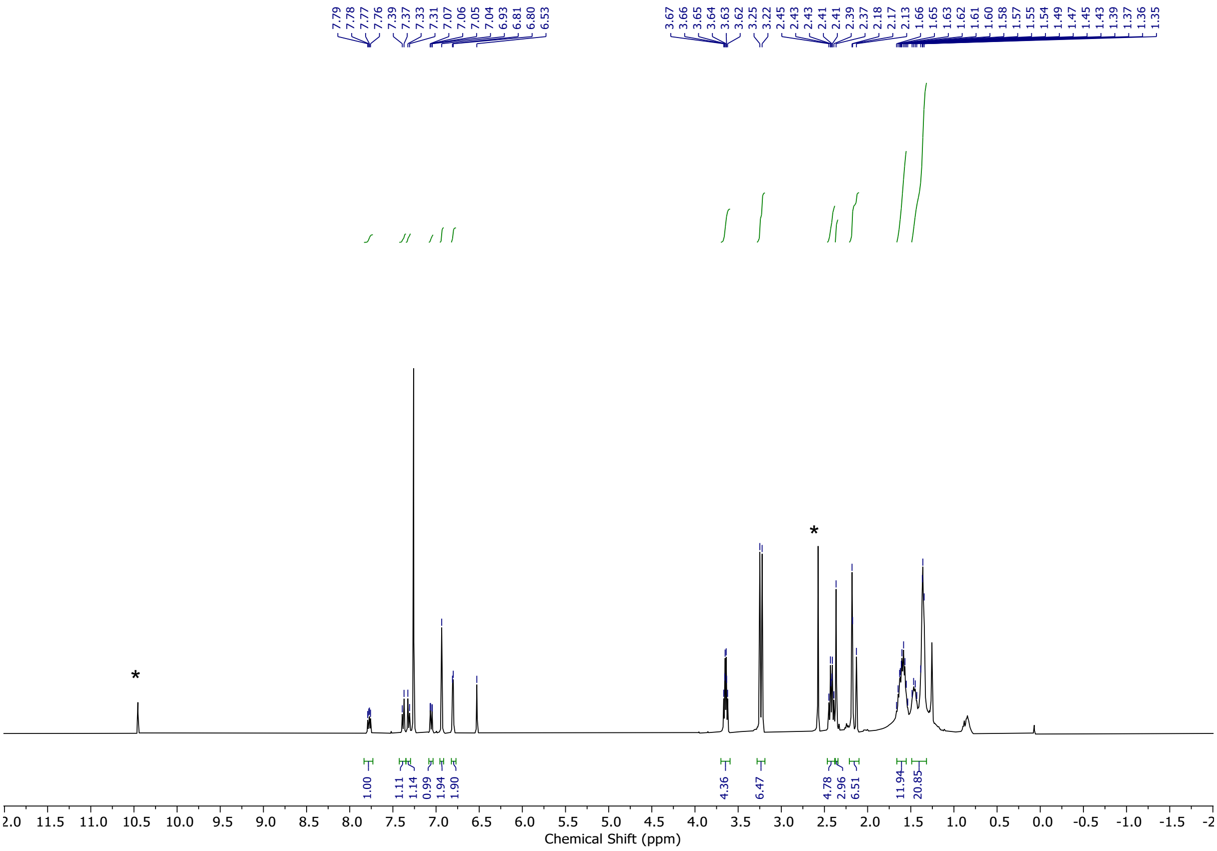

Figure S177. *^{1}H NMR spectrum of (E$_S$Z$_S$)-**8** (CDCl$_3$, 20 °C). The peaks marked with an asterisk arise from an aliphatic impurity.*

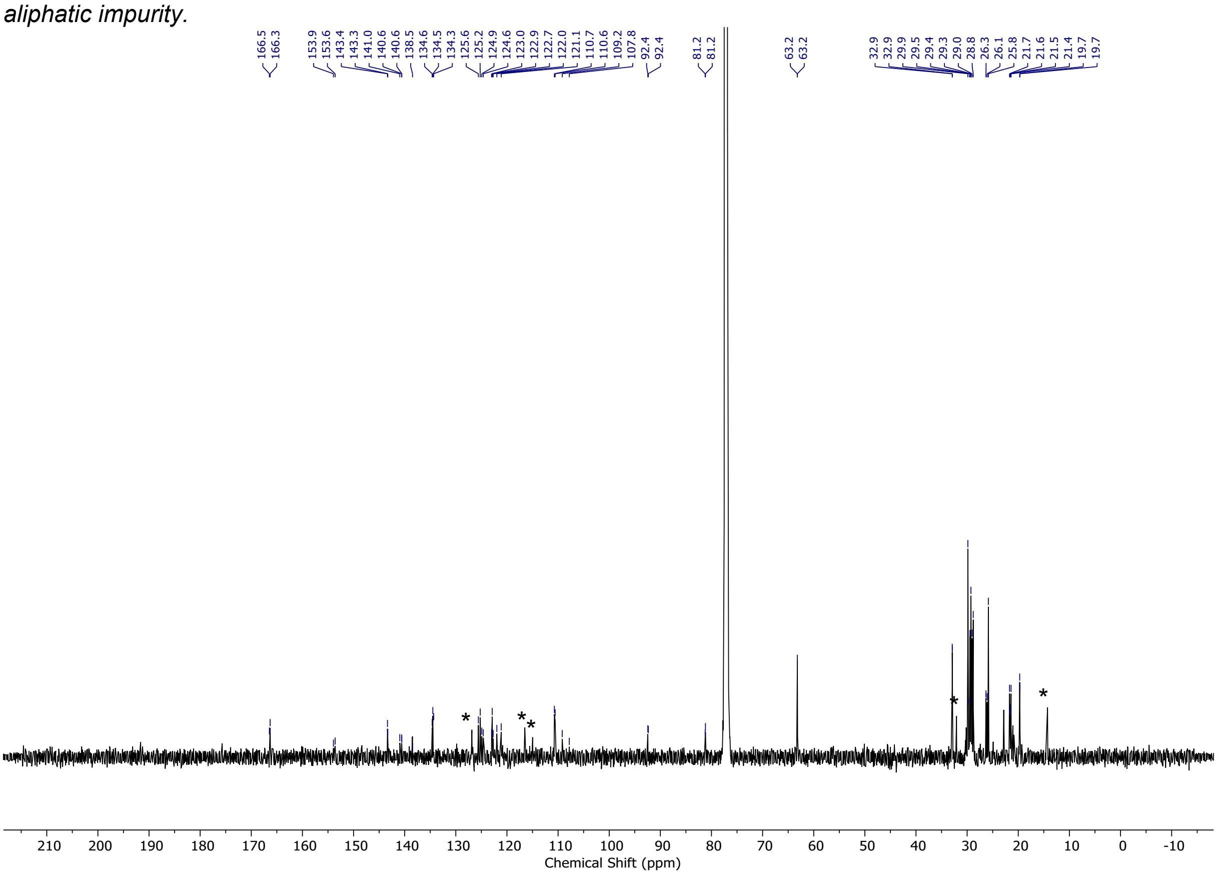

Figure S178. *^{13}C NMR spectrum of (E$_S$Z$_S$)-**8** (CDCl$_3$, 20 °C). The peaks marked with an asterisk arise from an aliphatic impurity.*

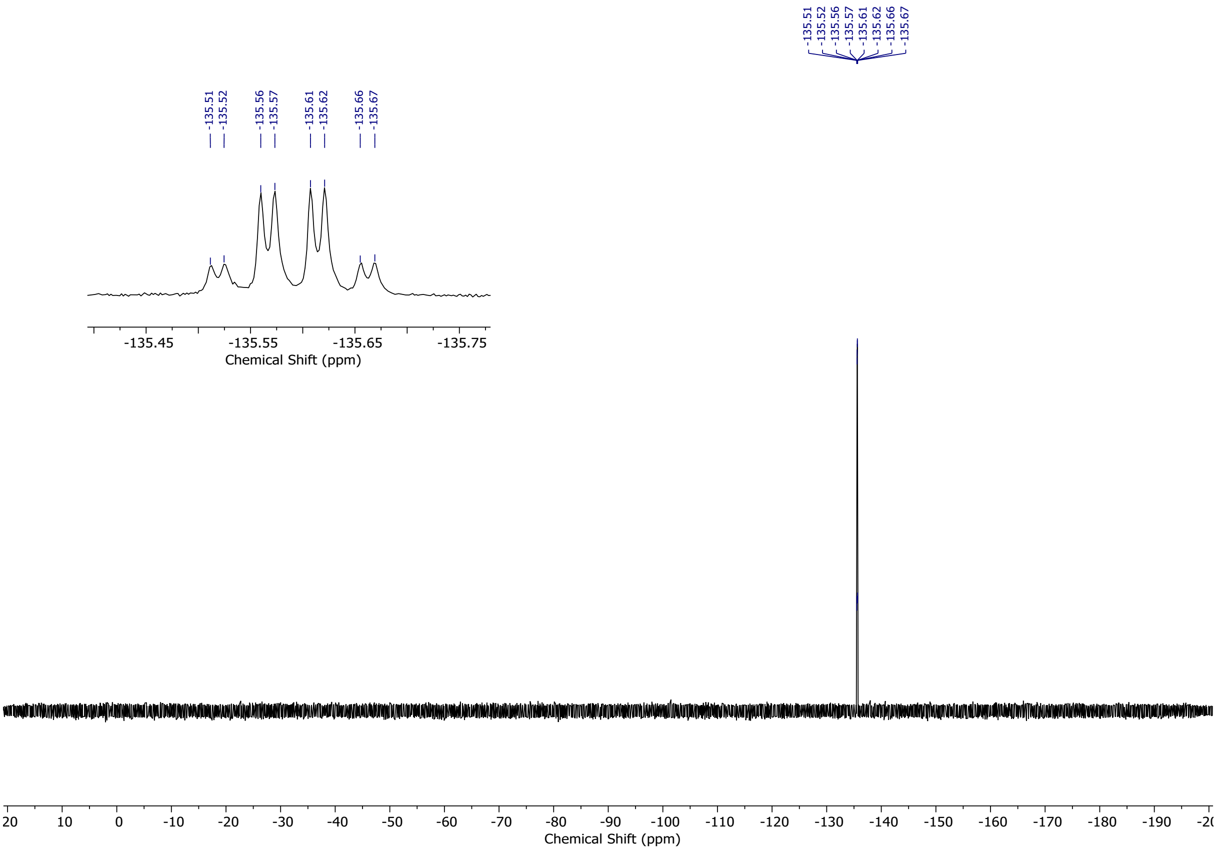

***Figure S179**. ^{19}F NMR spectrum of (E_SZ_S)-**8** ($CDCl_3$, 20 °C).*

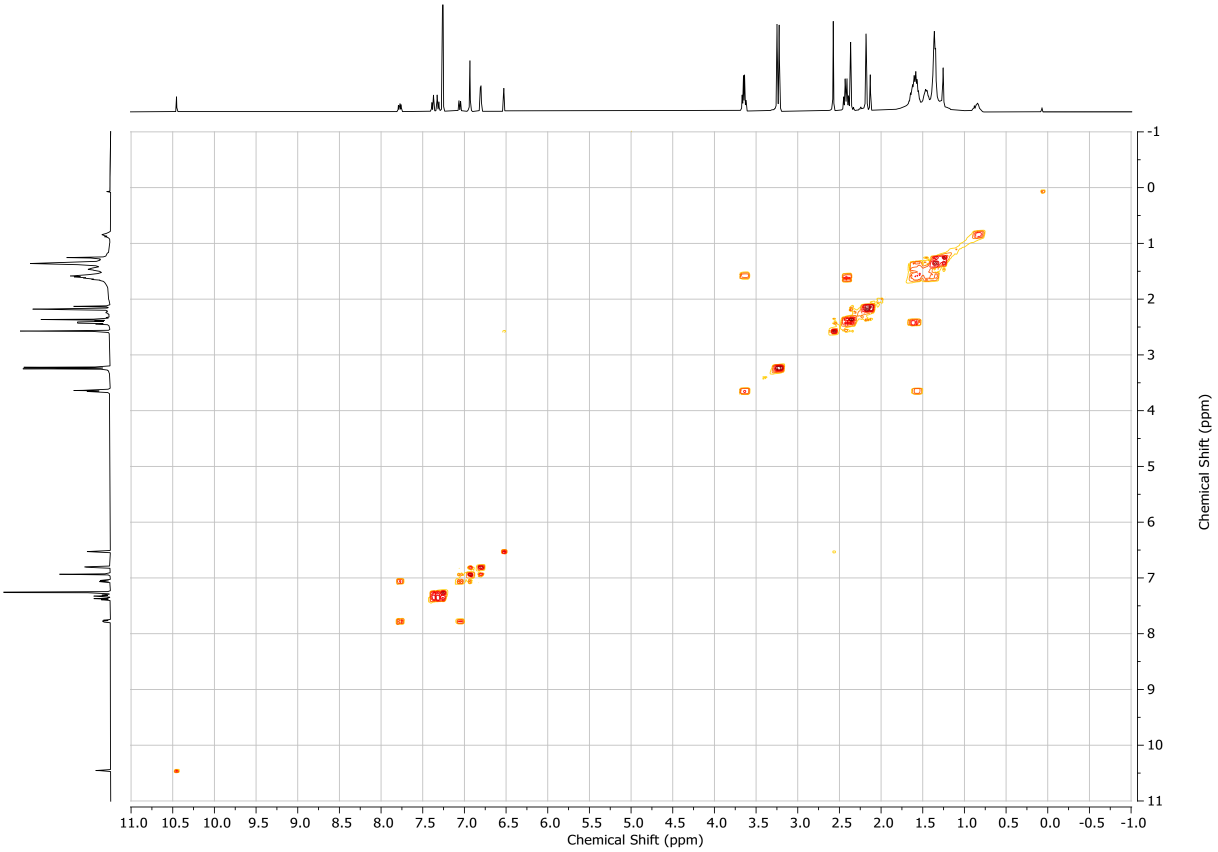

***Figure S180**. COSY NMR spectrum of (E_SZ_S)-**8** ($CDCl_3$, 20 °C).*

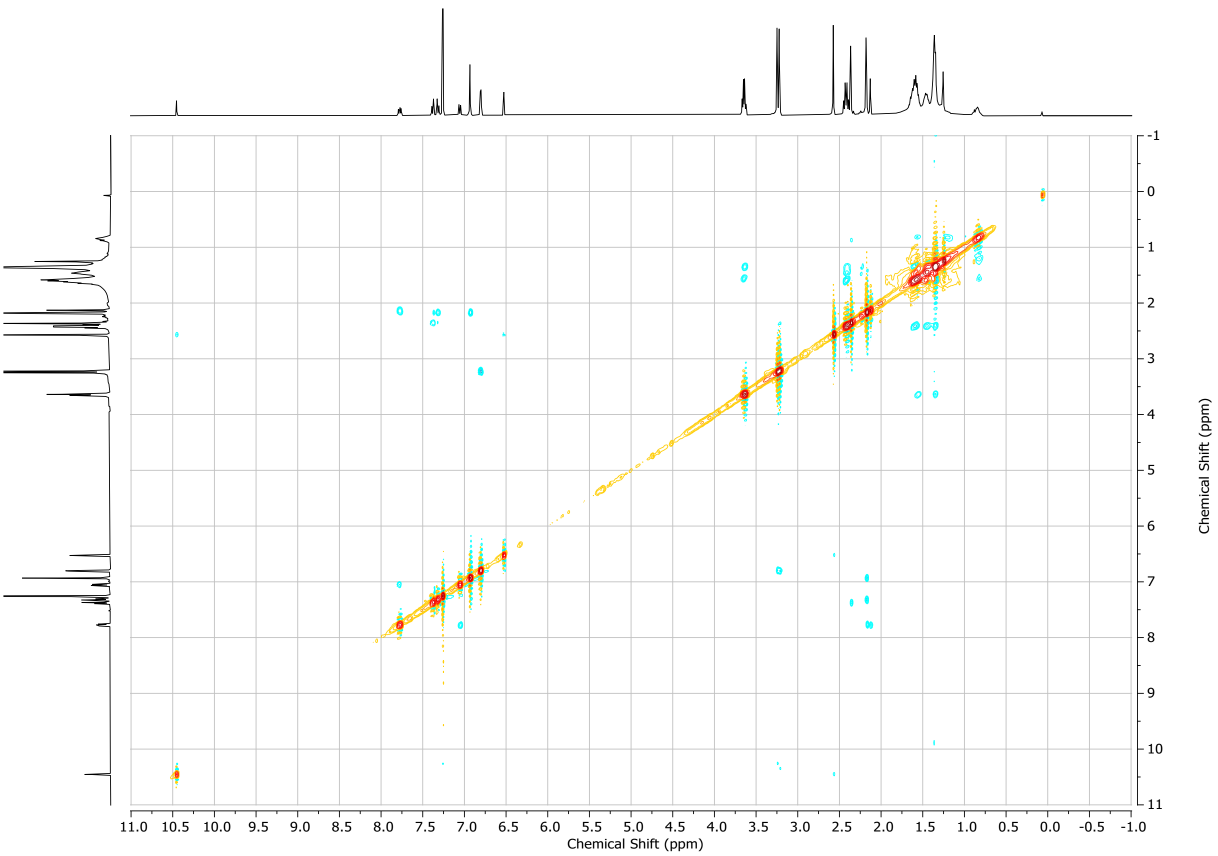

Figure S181. *NOESY NMR spectrum of (E_SZ_S)-**8** ($CDCl_3$, 20 °C).*

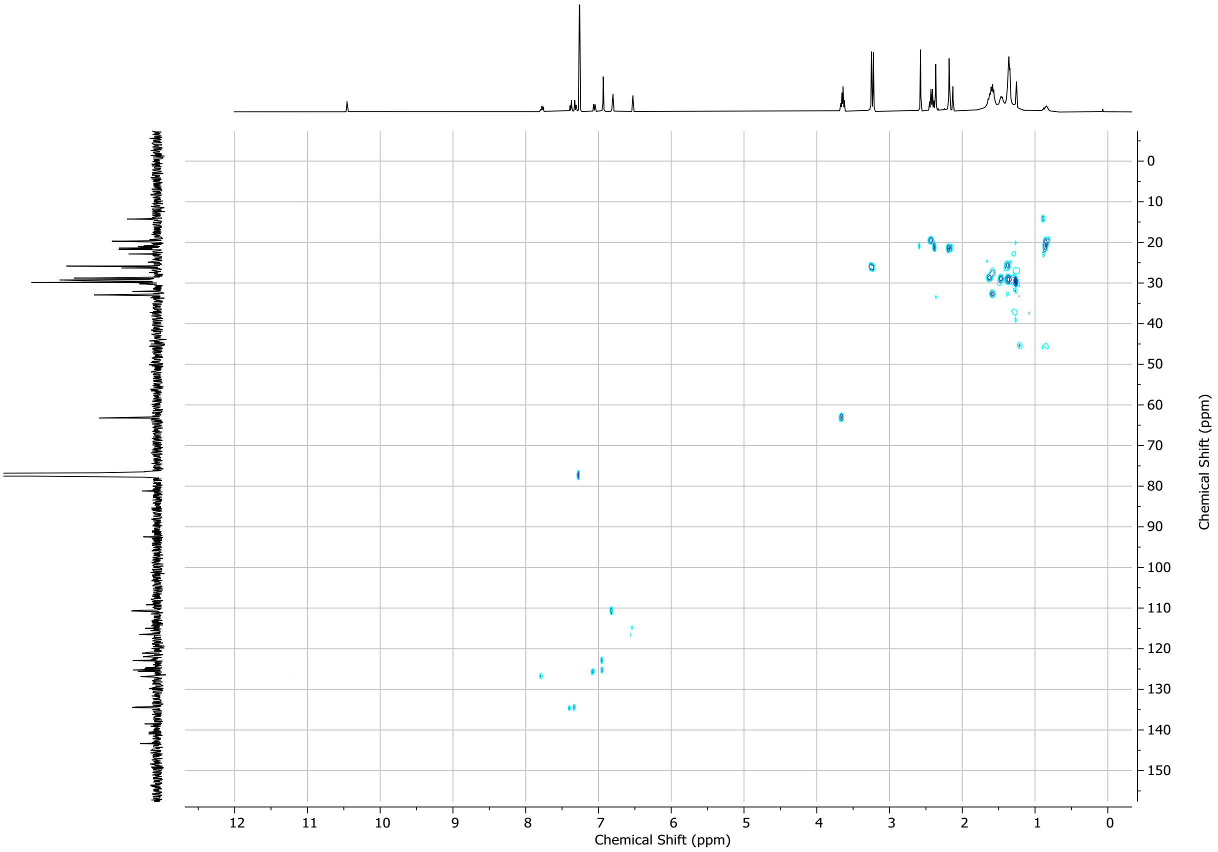

Figure S182. *HSQC NMR spectrum of (E_SZ_S)-**8** ($CDCl_3$, 20 °C).*

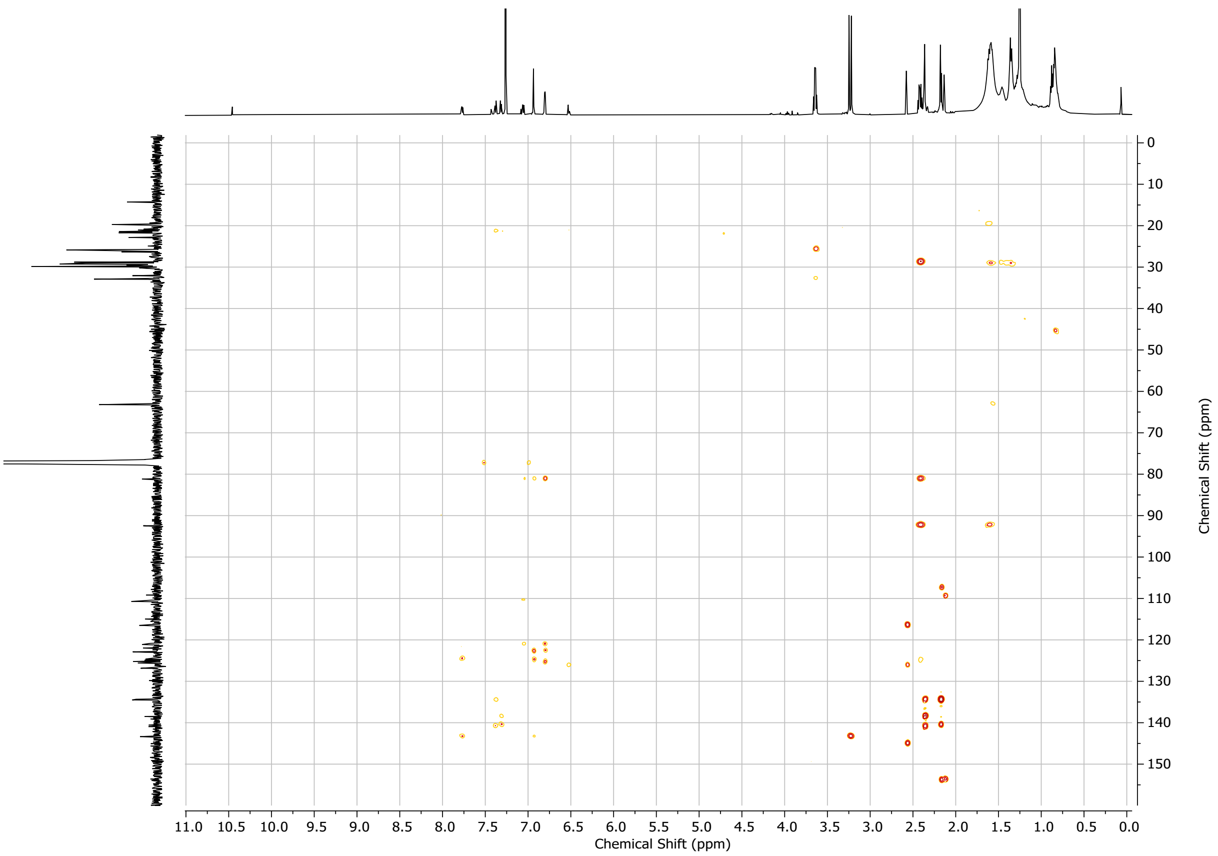

Figure S183. *HMBC NMR spectrum of (E_SZ_S)-**8** ($CDCl_3$, 20 °C).*

8. HRMS Spectra

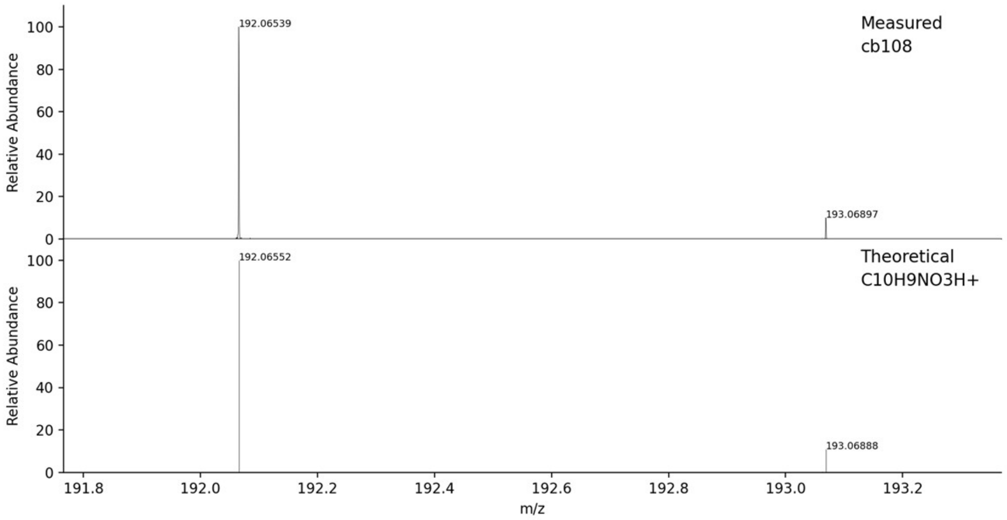

Figure S184. *HRMS (ESI$^+$) of **S5a** (top: measured, bottom: calcd.).*

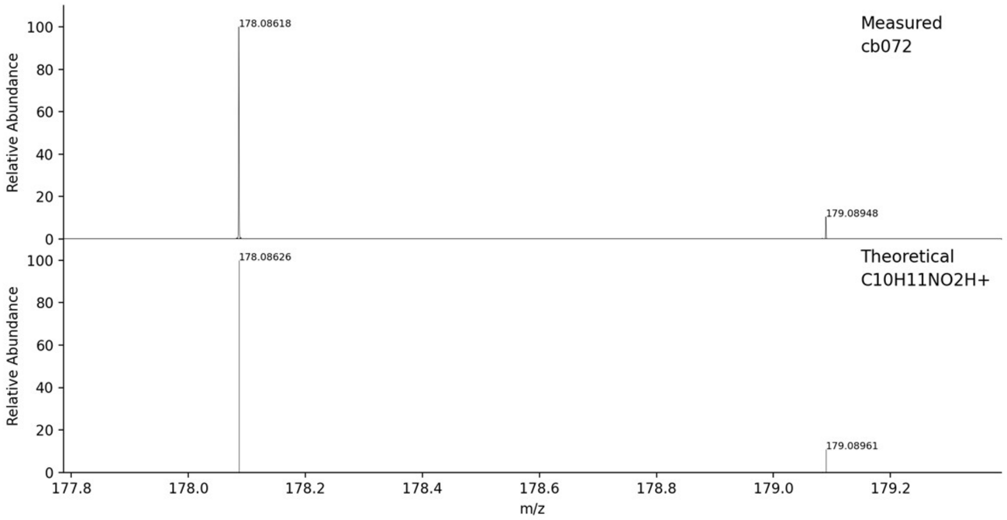

Figure S185. *HRMS (ESI$^+$) of **S2** (top: measured, bottom: calcd.).*

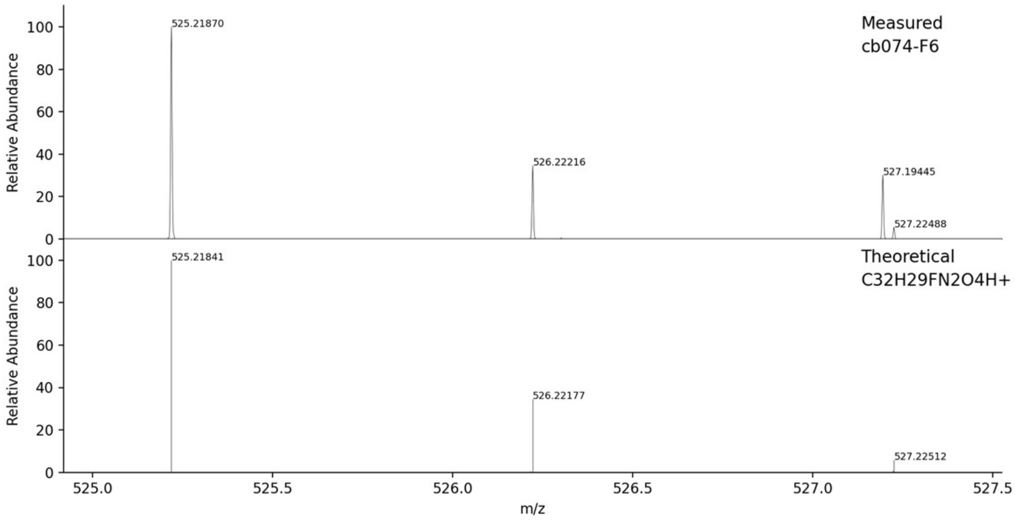

Figure S186. *HRMS (ESI*$^{+}$*) of (*Z_SZ_S*)-**2** (top: measured, bottom: calcd.).*

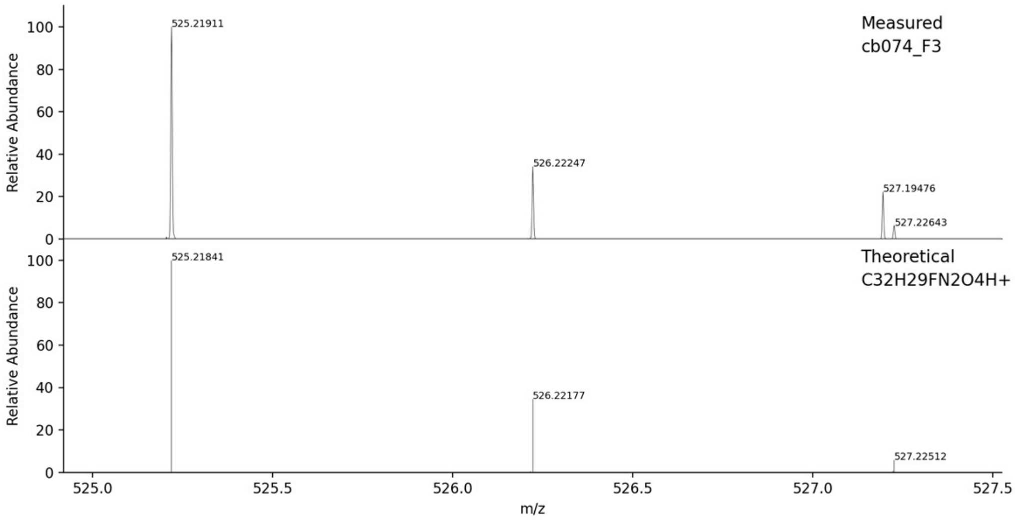

Figure S187. *HRMS (ESI*$^{+}$*) of (*E_SZ_S*)-**2** (top: measured, bottom: calcd.).*

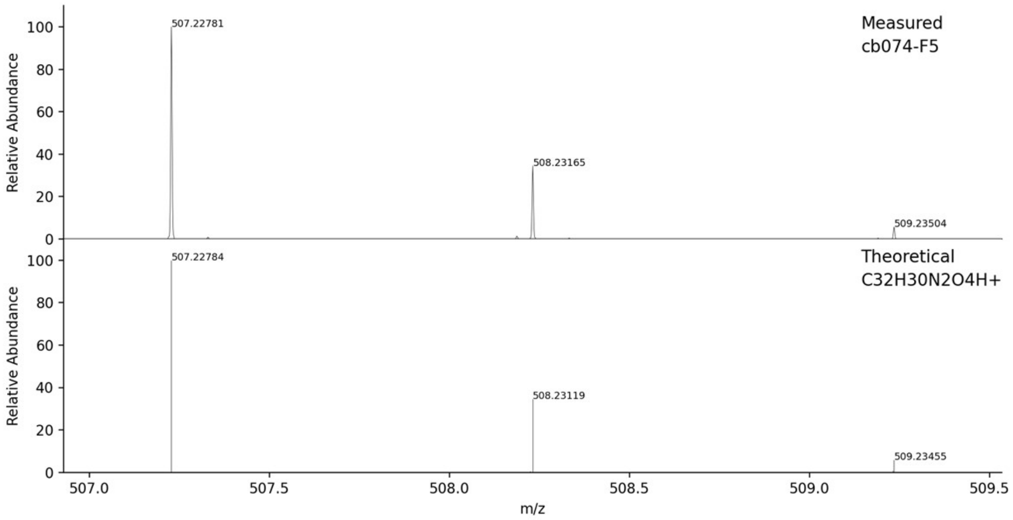

Figure S188. *HRMS (ESI⁺) of (Z_SZ_S)-**2DeF** (top: measured, bottom: calcd.).*

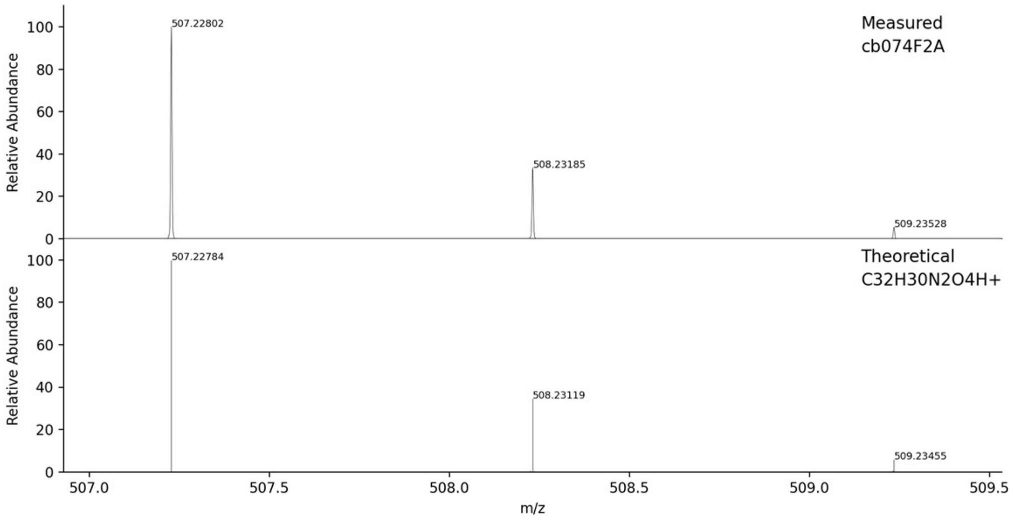

Figure S189. *HRMS (ESI⁺) of (E_SZ_S)-**2DeF** (top: measured, bottom: calcd.).*

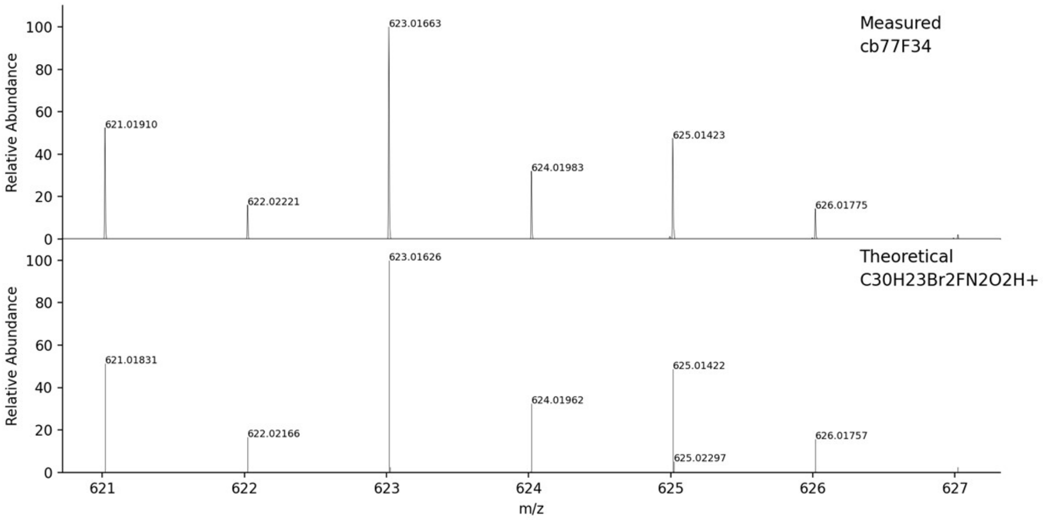

Figure S190. *HRMS (ESI⁺) of (Z_SZ_S)-**3** (top: measured, bottom: calcd.).*

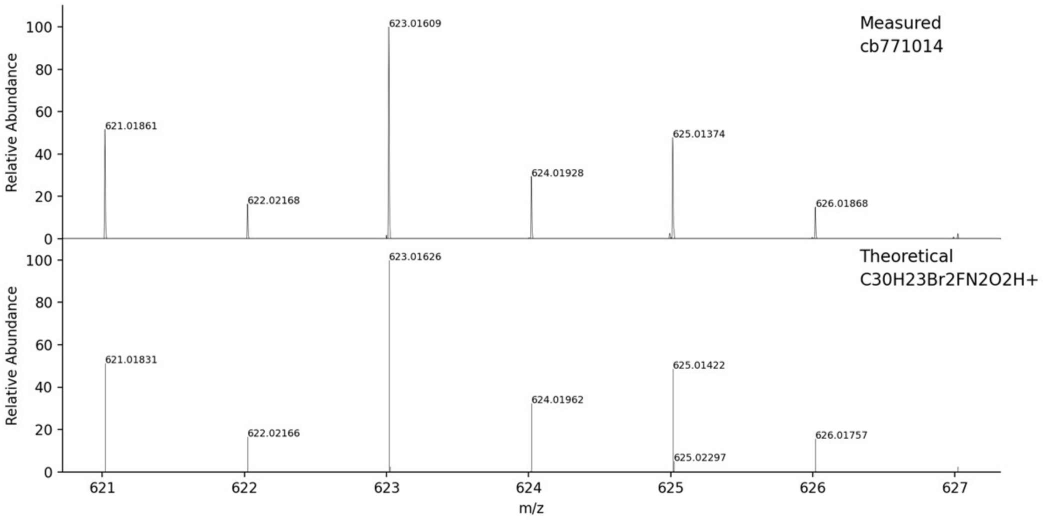

Figure S191. *HRMS (ESI$^+$) of (E_SZ_S)-**3** (top: measured, bottom: calcd.).*

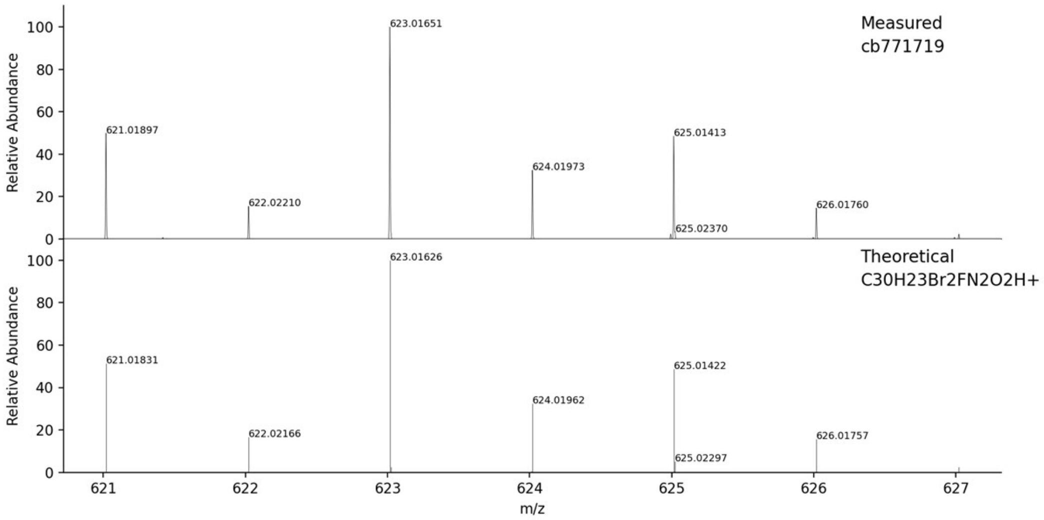

Figure S192. *HRMS (ESI⁺) of (E_SE_S)-**3** (top: measured, bottom: calcd.).*

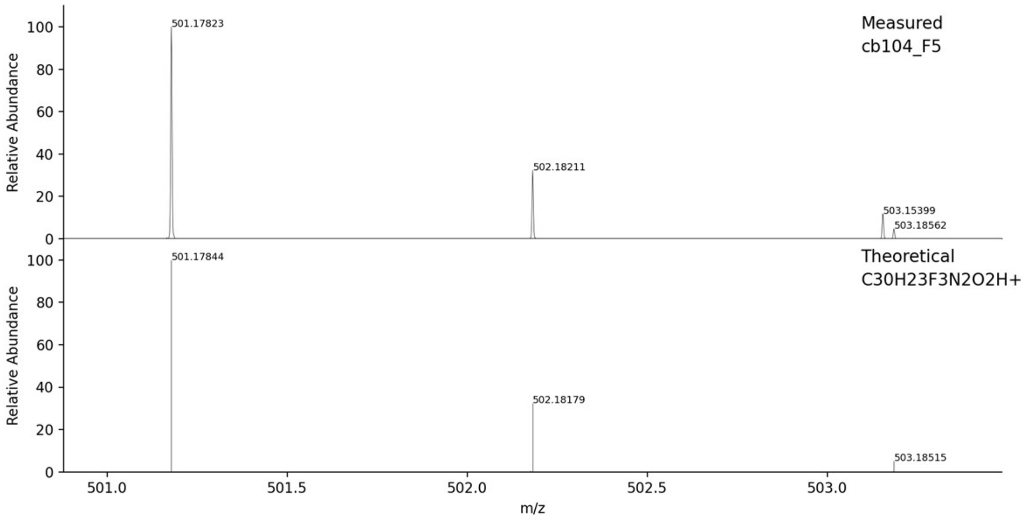

Figure S193. *HRMS (ESI⁺) of (Z_SZ_S)-**4** (top: measured, bottom: calcd.).*

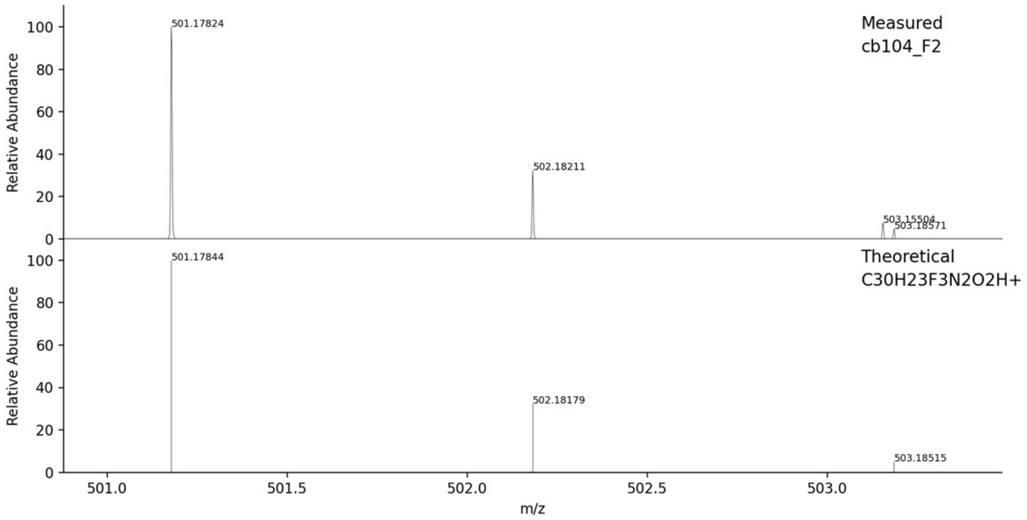

Figure S194. *HRMS (ESI⁺) of (E_SZ_S)-**4** (top: measured, bottom: calcd.).*

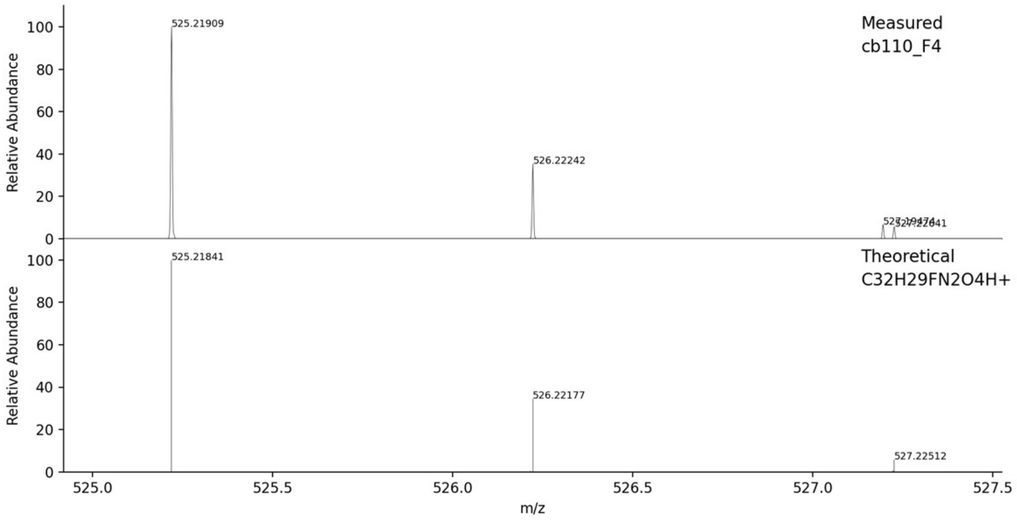

Figure S195. *HRMS (ESI⁺) of (Z_SZ_S)-**5** (top: measured, bottom: calcd.).*

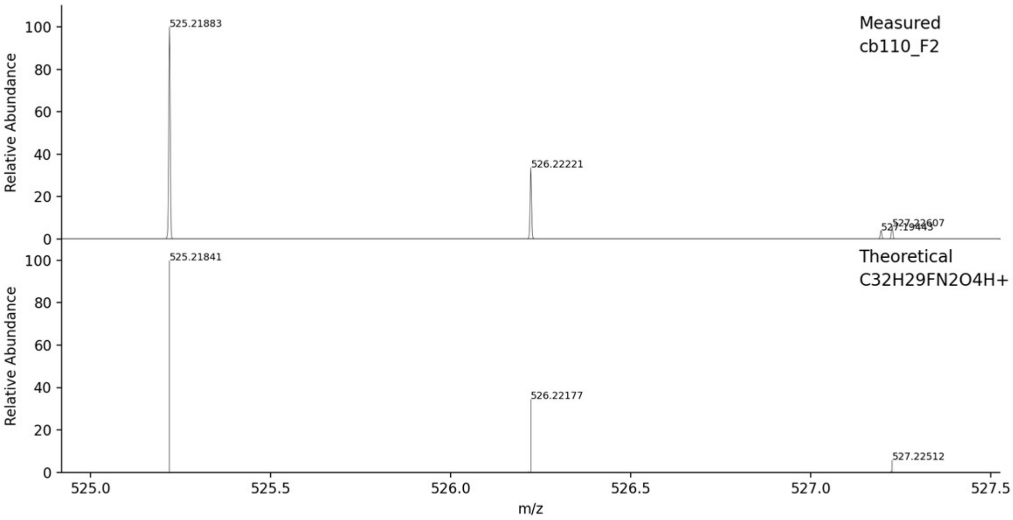

Figure S196. *HRMS (ESI⁺) of* (E_SZ_S)-***5*** *(top: measured, bottom: calcd.).*

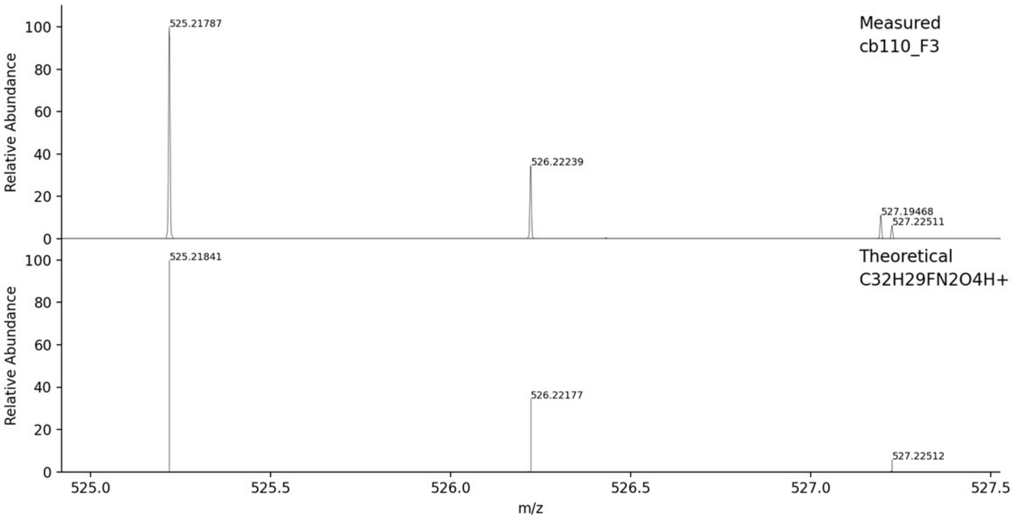

Figure S197. *HRMS (ESI⁺) of* (E_SE_S)-***5*** *(top: measured, bottom: calcd.).*

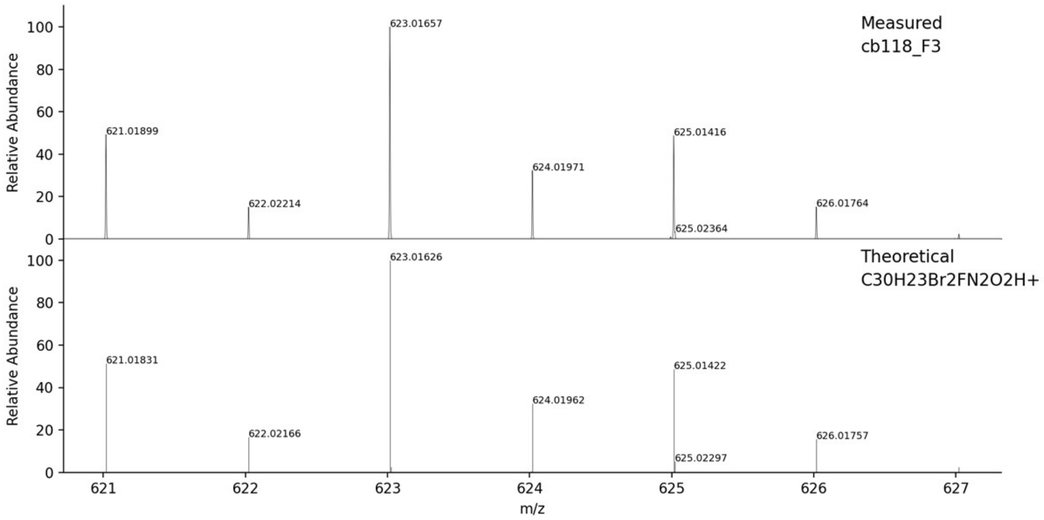

Figure S198. *HRMS (ESI⁺) of (Z_SZ_S)-**6** (top: measured, bottom: calcd.).*

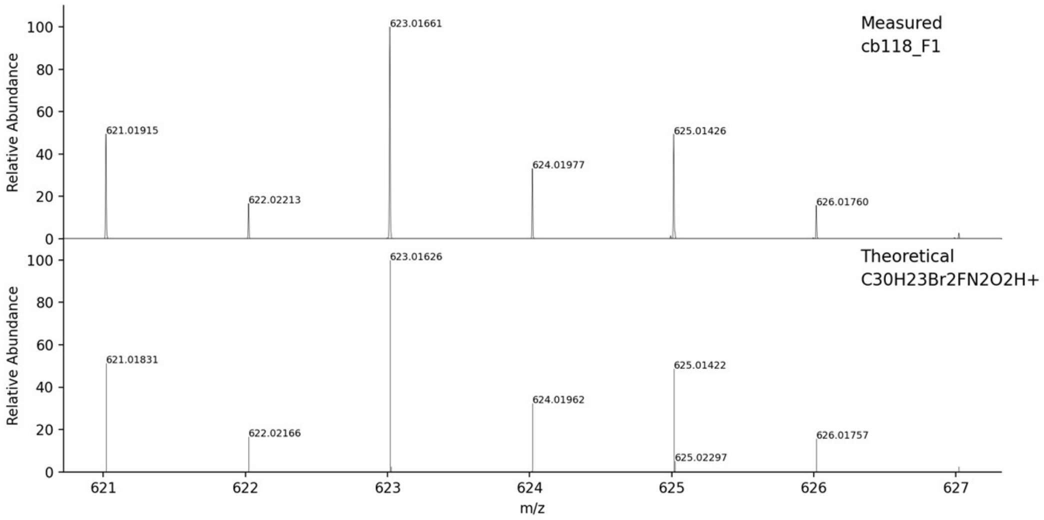

Figure S199. *HRMS (ESI⁺) of (E_SZ_S)-**6** (top: measured, bottom: calcd.).*

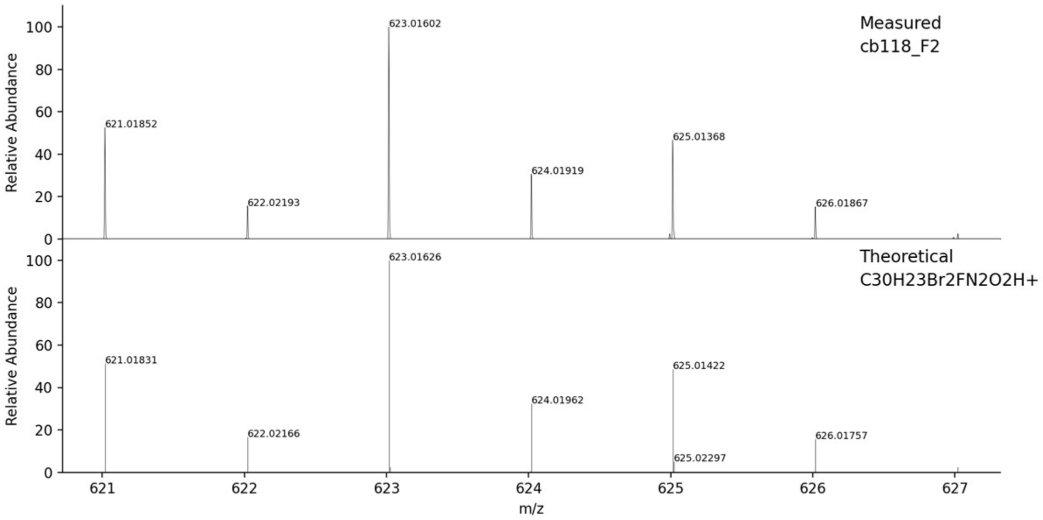

Figure S200. *HRMS (ESI⁺) of (E_SE_S)-**6** (top: measured, bottom: calcd.).*

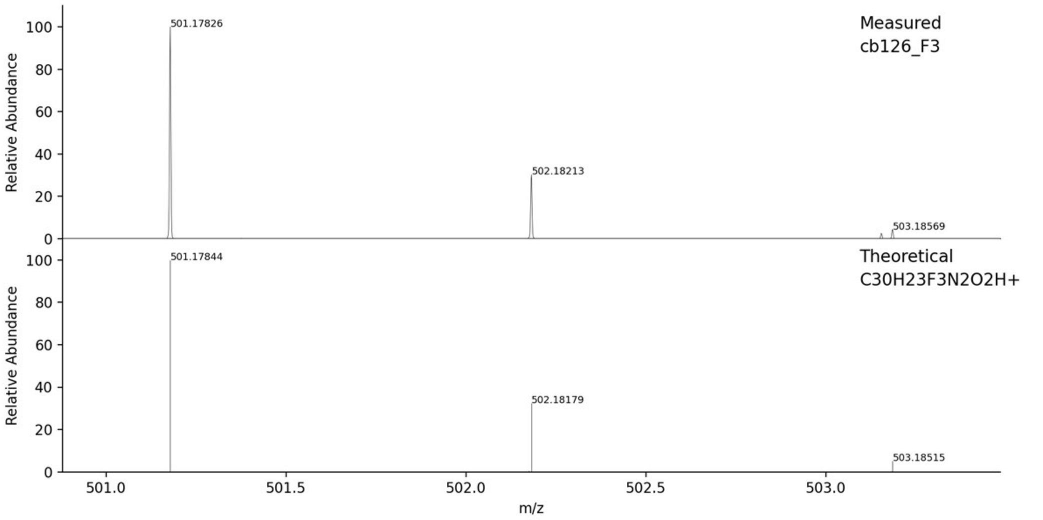

Figure S201. *HRMS (ESI⁺) of (Z_SZ_S)-**7** (top: measured, bottom: calcd.).*

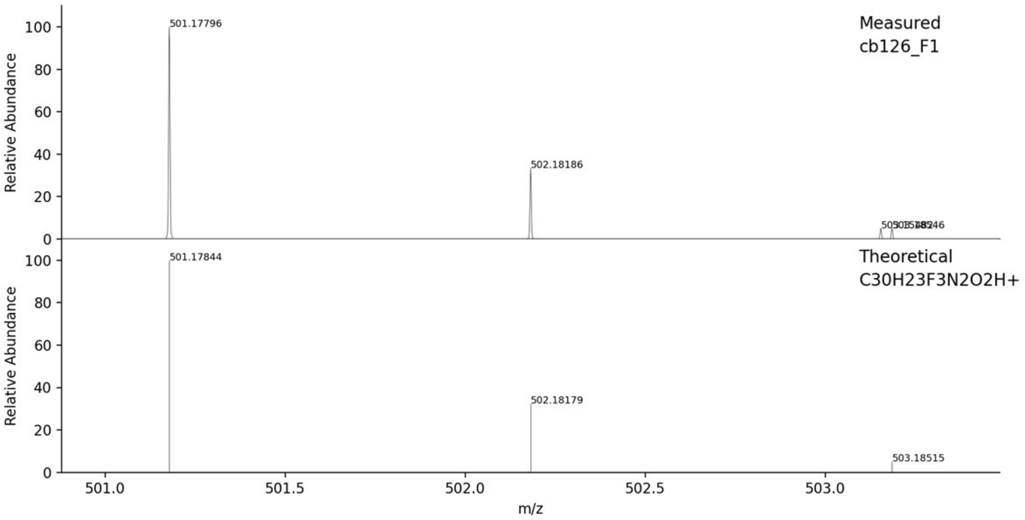

Figure S202. *HRMS (ESI$^+$) of (E_SZ_S)-**7** (top: measured, bottom: calcd.).*

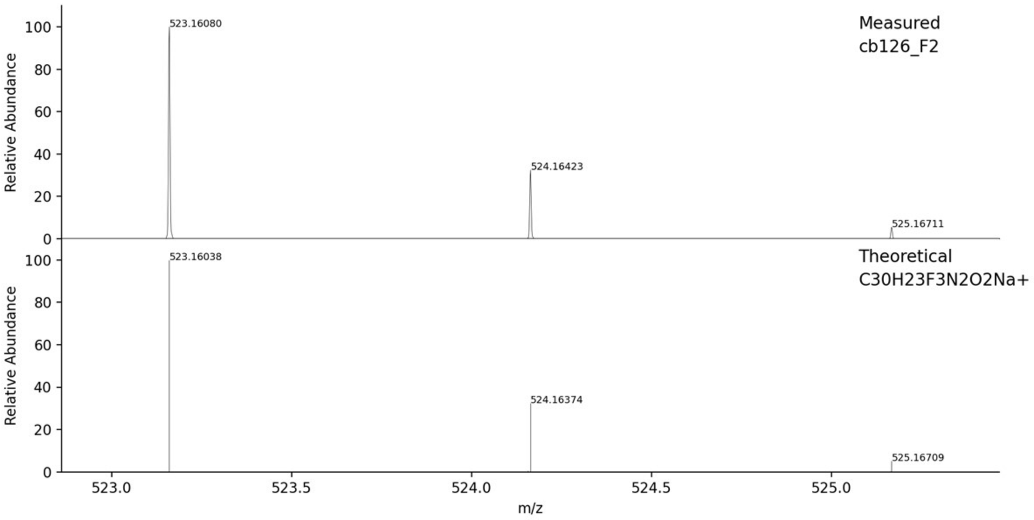

Figure S203. *HRMS (ESI$^+$) of (E_SE_S)-**7** (top: measured, bottom: calcd.).*

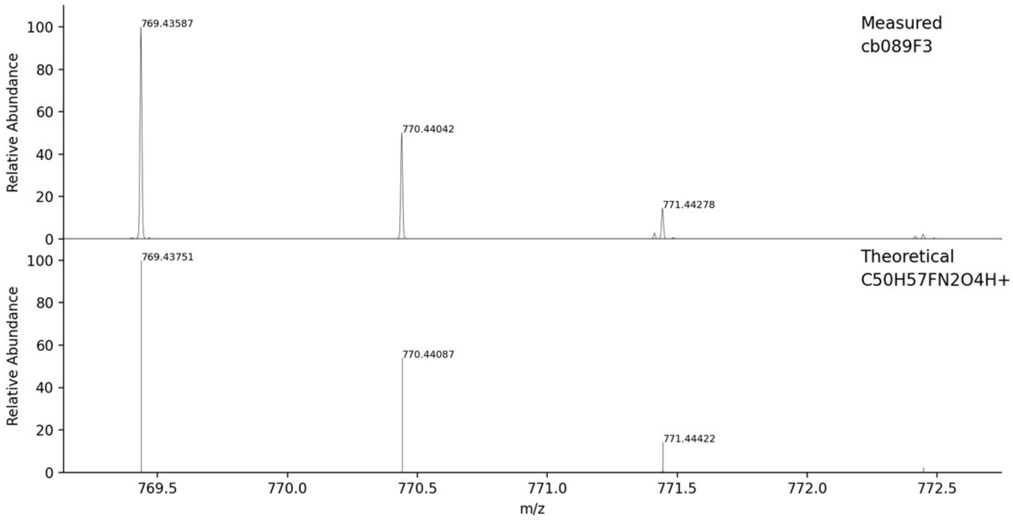

Figure S204. *HRMS (ESI$^+$) of (E_SZ_S)-**8** (top: measured, bottom: calcd.).*

9. References

1 C. L. F. Van Beek and B. L. Feringa, *J. Am. Chem. Soc.*, 2024, **146**, 5634–5642.
2 J.-Q. Zhang, S.-M. Li, C.-F. Wu, X.-L. Wang, T.-T. Wu, Y. Du, Y.-Y. Yang, L.-L. Fan, Y.-X. Dong, J.-T. Wang and L. Tang, *Catal. Commun.*, 2020, **138**, 105838.
3 H. Ji, Y. Zhu, Y. Shao, J. Liu, Y. Yuan and X. Jia, *J. Org. Chem.*, 2017, **82**, 9859–9865.
4 Y. Liao, Y. Gao, W. Zheng and R. Tang, *Adv. Synth. Catal.*, 2018, **360**, 3391–3400.
5 X. K. Wee, T. Yang and M. L. Go, *ChemMedChem*, 2012, **7**, 777–791.
6 Z. Lian, S. D. Friis and T. Skrydstrup, *Angew. Chem. Int. Ed.*, 2014, **53**, 9582–9586.
7 R. Le Goff, M. Sanselme, A. M. Lawson, A. Daïch and S. Comesse, *Eur. J. Org. Chem.*, 2015, **2015**, 7244–7248.
8 F.-C. Jia, C. Xu, Z.-W. Zhou, Q. Cai, Y.-D. Wu and A.-X. Wu, *Org. Lett.*, 2016, **18**, 5232–5235.
9 G. Zhao, L. Liang, E. Wang, S. Lou, R. Qi and R. Tong, *Green Chem.*, 2021, **23**, 2300–2307.
10 C. Prandi, E. G. Occhiato, S. Tabasso, P. Bonfante, M. Novero, D. Scarpi, M. E. Bova and I. Miletto, *Eur. J. Org. Chem.*, 2011, **2011**, 3781–3793.
11 H. L. Schmitt, D. Martymianov, O. Green, T. Delcaillau, Y. S. Park Kim and B. Morandi, *J. Am. Chem. Soc.*, 2024, **146**, 4301–4308.
12 H.-G. Cheng, M. Pu, G. Kundu and F. Schoenebeck, *Org. Lett.*, 2020, **22**, 331–334.
13 Y. Qutbuddin, A. Guinart, S. Gavrilović, K. Al Nahas, B. L. Feringa and P. Schwille, *Adv. Mater.*, 2024, **36**, 2311176.
14 A. Guinart, Y. Qutbuddin, A. Ryabchun, J.-H. Krohn, P. Schwille and B. L. Feringa, *Chem*, 2025, 102574.
15 J. Schindelin, I. Arganda-Carreras, E. Frise, V. Kaynig, M. Longair, T. Pietzsch, S. Preibisch, C. Rueden, S. Saalfeld, B. Schmid, J.-Y. Tinevez, D. J. White, V. Hartenstein, K. Eliceiri, P. Tomancak and A. Cardona, *Nat. Methods*, 2012, **9**, 676–682.
16 F. Neese, F. Wennmohs, U. Becker and C. Riplinger, *J. Chem. Phys.*, 2020, **152**, 224108.
17 S. Grimme, A. Hansen, S. Ehlert and J.-M. Mewes, *J. Chem. Phys.*, 2021, **154**, 064103.
18 V. Barone and M. Cossi, *J. Phys. Chem. A*, 1998, **102**, 1995–2001.
19 F. Weigend and R. Ahlrichs, *Phys. Chem. Chem. Phys.*, 2005, **7**, 3297.
20 J.-D. Chai and M. Head-Gordon, *Phys. Chem. Chem. Phys.*, 2008, **10**, 6615.
21 S. Grimme, J. Antony, S. Ehrlich and H. Krieg, *J. Chem. Phys.*, DOI:10.1063/1.3382344.
